# Impact of Living Environment on Attachment Behaviour in Domestic Cats from Private Homes and Shelters

**DOI:** 10.3390/ani15243521

**Published:** 2025-12-05

**Authors:** Isabelle Kappel, Bianca Materne, Udo Gansloßer

**Affiliations:** 1 Institute of Zoology and Evolutionary Research, Friedrich Schiller University Jena, Erbertstr. 1, 07743 Jena, Germany; udo@ganslosser.de; 2 Institute of Biometry and Clinical Epidemiology, Charité-Berlin University of Medicine, Charitéplatz 1, 10117 Berlin, Germany

**Keywords:** strange situation test, domestic cats, attachment behaviour

## Abstract

Since cats often struggle to acclimate to laboratory settings, a key requirement for effective testing, we adapted Ainsworth’s Strange Situation Test (SST) for use in environments familiar to the cats. We examined 82 cat–caregiver dyads from private households and animal shelters using a standardised five-phase video procedure comprising distinct phases of presence, separation, and reunion between the cats and their human caregivers. Seven attachment-related behaviours were recorded via focal animal sampling and analysed using mixed models. Significant differences were observed in exploratory behaviour, physical contact, play (social, object, and locomotor), passive behaviour, and vocalisation. Cats from private homes exhibited more exploratory and play behaviour, particularly after reuniting with their caregiver, indicating a positive influence of the familiar person’s presence. Cats from animal shelters exhibited reduced play behaviour, more passive behaviour, and more frequent purring, particularly following reunion. Physical contact increased following separations, indicating attachment. Meowing was most frequent during absences and reunions, possibly reflecting stress and renewed social contact. Outdoor access emerged as a potential influencing factor for exploratory behaviour. In summary, attachment behaviours in cats were significantly influenced by the living environment, access to the outdoors, and the presence of familiar or unfamiliar individuals. Certain test phases elicited particularly strong behavioural responses.

## Simple Summary

Cats often struggle to acclimate to new environments such as laboratories, making behavioural studies challenging. To better understand how cats bond with their caregivers, we changed a common test so it could be performed in the cats’ own homes or shelters, where they feel comfortable. We observed 82 cats living in private homes or animal shelters during different moments of being with, separated from, and reunited with their caregivers. We looked at behaviours such as exploration, play, making contact, and vocalisation. Cats living in private homes showed higher levels of exploration and play, particularly following reunion with their caregivers, suggesting a higher level of security and caregiver-associated comfort. Shelter cats played less, acted more quietly, and purred more after being reunited with their caregiver, which may show stress or comfort-seeking. Cats from both groups showed more physical contact after being apart, which is a sign of attachment. Meowing was common when cats were left alone or reunited, likely because they felt stressed or wanted attention. Access to outdoor spaces also seemed to help cats explore more. Understanding these attachment behaviours helps improve cat welfare and strengthen the human–animal relationship.

## 1. Introduction

The Strange Situation Test (SST), developed by Ainsworth [1], shows that infants exhibit different attachment behaviours in relation to their primary caregiver. It has been widely used as a valid instrument for investigating attachment behaviour. Bowlby’s criteria for secure attachment in children are measured based on four behavioural dimensions.

In unfamiliar environments, a securely attached child views the caregiver as a source of physical closeness, a secure base for exploration, a safe haven during distress, responding to separation with noticeable stress followed by rapid calming upon reunion. Deviations from these patterns indicate insecure attachment, classified into distinct subtypes based on behavioural responses [2]. In recent decades, the SST has been adapted for assessing attachment behaviour in non-human animals like dogs or chimpanzees. Using an SST, Bard was able to demonstrate that the attachment classifications in nursery-reared chimpanzees (*Pan troglodytes*) were similar to those of human infants [3]. Topál et al. observed fifty-one dog–caregiver pairs in a modified version of the SST [4]. The study was able to show that adult dogs (*Canis familiaris*) exhibit patterns of attachment behaviour towards their caregivers. There were considerable differences in the attachment behaviour of dogs, although the authors found no effects of sex, age, housing conditions, or breed of the dog on most behavioural variables. In the following years, further studies detected various signs of attachment of dogs to their humans [5,6,7,8,9]. Notable here is the work of Mariti et al., who also tested dogs and their canine companions alongside the humans for separation and reunion [10]. The results suggest that the intraspecific bond could be stronger than the bond with humans. In this context, it was also discussed that dogs that live together with other dogs are rarely completely alone and therefore might show a stronger effect in relation to their conspecifics. Although this question could not be definitively resolved, it remains an interesting aspect to consider the broader context and the specific manifestations of attachment behaviour in non-human animals. In summary, many leading authors agree that non-human animals also exhibit signs of attachment behaviour toward humans.

Ultimately, the SST has also been adapted for cats (*Felis silvestris catus*) by Potter & Mills. In a cross-over test, 20 cat–caregiver pairs went through two versions of the SST in laboratory conditions. The duration of cat attachment behaviours was recorded [11]. Although the cats vocalised more in the presence of the caregiver, rather than the stranger, there were no other signs of attachment between the cat and its human. The authors eventually had to exclude several cats that hid for the entire test period and had not acclimatised to the new space by the end of the test setting. Further work with cats and the SST found evidence that cats show signs of attachment behaviour [12,13]. Due to the limited sample sizes in many studies, the results should be regarded as preliminary findings that require further validation. Furthermore, laboratory conditions appear to be consistently more challenging for cats than for dogs, potentially influencing their behaviour and the validity of experimental findings. In contrast, Eriksson et al. observed the behaviour of cats that were left alone at home and then reunited to their caregivers [14]. Although this is not an SST in the strict sense, cats initiated more social contact towards their caregiver after separation in a familiar environment.

The SST requires a ‘strange situation’, i.e., an unfamiliar room. This is essential not only for the ‘strange situation’ paradigm but also due to the standardised conditions of the laboratory. Studies also show that dogs and cats acclimatise very differently to unfamiliar environments. A study by Uccheddu et al. demonstrated that, unlike dogs—of which 100% passed a habituation test on the first attempt—only 40% of cats initially met the criteria, with the remaining 60% refusing food and interaction, and although repeated exposure increased habituation rates, approximately 40% still failed or withdrew by the third attempt, leading the authors to conclude that direct comparisons between cats and dogs under identical experimental conditions are problematic due to the cats’ need for prior acclimatisation to unfamiliar laboratory settings [15]. Another study compared the interaction of cats and dogs with a moving object, again within a laboratory room. The dogs showed a fundamentally greater interest in exploration and interaction. The authors of the study argued that, despite a one-time habituation of the cats in advance, the stress factor should not be underestimated and recommended that future studies be conducted in a familiar environment [16]. The stress-relieving effect of a familiar environment was also documented by Nibblett and her co-authors, who found that cats that underwent veterinary procedures at home showed less stress-related behaviour than cats in a clinic [17].

In summary, cats exhibit signs of attachment behaviour towards their caregiver, but they are more challenging to acclimate to unfamiliar environments, necessitating careful consideration of the testing conditions. This study aims to adapt the SST for cats, based on Ainsworth and Topál et al. [1,4], to examine human–cat relationship dynamics in a domestic setting and to assess whether a familiar environment reduces stress and provides clearer insights. In addition, we examined two groups of cats from animal shelters and from private homes. We have employed the SST to investigate the following questions: Do domestic cats show different attachment behaviour towards their caregiver compared to a stranger? Do domestic cats from private homes, in contrast to cats from animal shelters, show different attachment behaviour in general?

## 2. Materials and Methods

### 2.1. Participants

A total of 82 cats along with their caregivers were recruited through the laboratory’s network in the Jena area in Thuringia, Germany and in the Vechta and Osnabrück in Lower Saxony, Germany. Of these, 15 cats were sourced from animal shelters and 67 from private homes. The prerequisites for participation were that all cats were healthy and had no chronic illnesses or acute complaints. Participation required also that the cats had lived under the same conditions in the shelter or private household for a minimum of three months, allowing for the establishment of a stable attachment system to the primary caregiver.

### 2.2. Test Environment, Data Collection, Materials and Methods

A pilot study using a shortened SST setting, with 19 cats in an animal shelter, was conducted [18]. Following the development of the final SST protocol, the data collection took place from June 2022 until July 2024. The tests were conducted in the living rooms of private homes and in the main rooms of animal shelters where the cats were typically housed, ensuring a familiar environment in each setting. In private homes with multiple cats, the respective cat undergoing the test remained alone in the familiar room until the first focus person entered. In shelter rooms with multiple cats, separation of one cat was not possible. Thus, one cat was selected beforehand and focused during testing. The observation method used was focal animal sampling in both settings, where a continuous stream of behaviour is recorded for a defined period of time [19,20]. To ensure objectivity and interrater reliability, four observers were trained and validated prior to data coding. All observers scored the same set of test videos, and their results were compared. If the deviation between individual ratings exceeded 5%, the differences were discussed until a consensus was reached on how to objectively score the respective behaviours. This process was repeated until consistent scoring across observers was achieved.

### 2.3. Procedure Protocol

The SST which was used in this trail was developed based on Topál et al. [4]. The behavioural categories for dogs were transferred to the behaviour of cats. Based on the findings from the pilot study, the protocol was adjusted to five video sequences, each three minutes long [18]. A phase in which the cat is alone in the room, comparable to video sequence 5 in Topál’s test, was omitted, as it was not possible in most of the rooms to install a camera in such a way that the cat remained in the picture as it wandered around the room. Interactions with the cat could include verbal contact and physical approach (for example, by stepping closer or stretching out an arm). The focal person (FP) was the cat’s interaction partner in the respective video sequence; observed behaviour was attributed to the focal person in the evaluation.

Video sequence 1–Familiar person and cat: The familiar person (FP) enters the room. They take over the camera and interact with the cat.

Video sequence 2–Familiar person, stranger, and cat: The stranger (FP) enters the room and interacts with the cat. The familiar person stays in the background and continues to direct the camera.

Video sequence 3–Stranger and cat: The familiar person hands over the camera and leaves the room. The stranger (FP) films and interacts with the cat.

Video sequence 4–Familiar person, stranger, and cat: The familiar person (FP) enters the room and interacts with the cat. The stranger stays in the background and continues the camera work.

Video sequence 5–Familiar person and cat: The stranger hands over the camera and leaves the room. The familiar person (FP) films and interacts with the cat.

### 2.4. Behavioural Categories

To classify observed behaviour, seven categories were defined—six based on Topál et al., with Category 7, ‘Vocalisation’, added due to pilot study findings indicating its relevance to attachment behaviour [4,18].

*Category 1:* Exploratory behaviour (state)—The cat orients itself in its environment—the cat sniffs at objects or inspects them tactilely or visually from close or at a distance. This category also included: observation of the partner animal or present person as well as observation of a moving toy from a distance, without interaction *.

*Category 2:* Play behaviour (state)—The cat interacts in a lively manner with a toy (object play) or an interaction partner (social play) as well as locomotor play and physical contact with the toy. Observations of the toy from a distance were not rated as play unless the cat fixated the object with sustained attention, actively imitated its movements with its head, or assumed a lurking position *.

*Category 3:* Behaviour by the door (state)—The time the cat has spent in close (distance less than one body length of the cat) proximity to the door; the face should be oriented towards the exit. Looking or staring towards the door also fell into this category, provided that the cat’s attention was focused on the door for longer than one second **.

*Category 4:* Passive behaviour (state)—The cat sits, stands, or lies down; the focus is not specifically on the environment. Food intake and grooming were also classified as passive behaviour, as the cat turns its attention away from its environment and potential interaction partners **.

*Category 5:* Physical contact (state)—Body contact with the interaction partner. Furthermore, sniffing at close range, provided the nose (of the cat) was less than one cat’s nose length from the human’s body **.

*Category 6:* Initiating contact (event).

*Category 6.1:* Approach—The cat establishes or intensifies closeness. This also includes tilting the head and cuddling or pressing into the caressing hand. Approaches motivated by toys or food were not counted, as the cat was not interested in the human. However, if the cat played directly with the human without being mediated by an object and made physical contact, this was also counted as an approach *.

*Category 6.2:* Avoidance—The cat signals discomfort by turning its head or body away, avoids (eye) contact, makes itself small when approached by the human. This includes, for example, hissing, ducking away, flinching, or fleeing. Strolling away due to a change in interest and jumping or any kind of play do not fall into this category *.

*Category 7:* Vocalisation (event).

*Category 7.1:* Meow—A guttural meow sound, which may be of varying length and depth *.

*Category 7.2:* Purr—A deep, rhythmic vibrating sound emanating from the throat and chest. It is heard both on exhalation and inhalation *.

*Category 7.3:* Hiss—A long, soft hissing sound made by rapidly expelling air from the cat’s mouth, usually when exhaling *.

*Category 7.4:* Growl—A low-pitched, throaty, rumbling noise produced while the mouth is closed *.

* For more details, cf. ethogram of Kappel et al. [21].

** For more details, cf. definition of Topál et al. [4].

For the categories exploratory behaviour, play behaviour, behaviour by the door, passive behaviour, and physical contact, the total duration in seconds was determined. These categories were evaluated as states in terms of their duration, which mainly characterise longer-lasting activities [19,20]. For Initiating contact and vocalisation with all subcategories, which can be classified as events due to their very limited duration, the occurrence per video sequence was determined. One exception was purring, which can occur for a longer duration. For the sake of comparability, we counted all vocalisations, regardless of whether purring lasted for an extended period.

### 2.5. Statistical Analysis

A total of 82 cats were included in this analysis. We continuously recorded behavioural data during the five video sequences. Depending on the behavioural category, the data was measured as duration in seconds or counted as events. The variables were analysed descriptively. Absolute and relative frequencies for categorical data are reported, as well as average duration for metric data, and median and quartiles for ordinal and metric data.

Given the hierarchical nature of the data with repeated measures, linear mixed models (LMMs) were chosen to examine the relationship between the dependent variable of attachment behaviour within the seven categories and the independent variables of living environment (private homes/animal shelters) and the video sequence representing interaction with different person types (familiar/stranger/combination) and outdoor access (outdoor/indoor). In the first step, we analysed the relationship between five categories of behaviour (exploratory behaviour, play behaviour, behaviour by the door, passive behaviour, physical contact) and the variable ‘living environment’, as well as the video sequence using an LMM to account for continuous data. The model included fixed effects for living environment and video sequence, as well as random intercepts for individual differences. Residual plots were inspected to verify that the assumptions of normality and homoscedasticity were met. Since the assumptions were violated for three behaviours (play behaviour, behaviour by the door, physical contact) the dependent variable was log-transformed to ensure normality and improve model fit. Additionally, a robust linear mixed model (RLMM) was computed, and t-values were compared to ensure the robustness and fit of the model. In the second step, a separate LMM was computed for the three behavioural categories (exploratory behaviour, play behaviour, physical contact) which had been identified by the laboratory’s preliminary tests as being influenced by the cat having outdoor access. This second series of models included the variable ‘outdoor access’ as an additional fixed effect as well as random intercept, as described before following the same approach regarding residuals and robustness again. In the third step, we analysed the relationship between four categories of behaviour (making contact—approach and avoiding, vocalisation—meow and purr) and the variable ‘living environment’ and the video sequence using a generalised linear mixed model (GLMM) with a negative binomial distribution to account for count data with repeated measures. The model included fixed effects for living environment and video sequence and random intercepts for individual differences. As is typical for count data, the data structure of all four categories exhibited the characteristic right skew. For three categories (approach, avoiding, purr), the negative binomial model provided the best representation of the complexity. For one category (meow), there were high numbers of zero values, which is why the previous model was not applicable. The zero-inflated model demonstrated the best fit, incorporating a negative binomial distribution for count data and a logistic regression for modelling extensive zero values. To determine the best-fitting model for the data, Akaike Information Criterion (AIC) and Bayesian Information Criterion (BIC) values were compared across several negative binomial models, and likelihood ratio tests were conducted. The model demonstrating the lowest AIC and BIC values was selected as the best-fitting model. According to Pek & Flora, it is advisable to report unstandardized effect sizes for models of this type [22]. In this case, we calculated and reported the estimates per category, along with their corresponding *p*-values. Due to the primarily exploratory character of this study, no correction for multiple testing was applied, and *p*-values should be interpreted as descriptive indicators. R Studio Version 4.4.2 was used for all statistical analysis, published 31st of October 2024. All R packages used are cited in the Supplementary Material.

### 2.6. Ethical Approval

Ethical review and approval were waived for this study because it involved non-invasive observations and video analyses of cats in their natural environment and routine behaviour. Particular care was taken to avoid interrupting the cats’ natural behaviour, both in private homes as well as the cats living in the shelter. The test was conducted with consideration for minimising stress for the animal. In German animal welfare regulations, no permission is needed to observe undisturbed animals (except for threatened wildlife) in their everyday life. We have also compared our procedure with the strict animal testing regulations in Switzerland [23]. In case of our Strange Situation Test on cats, the severity level would be 0: no pain, suffering, injury, or fear is inflicted on an animal during an experiment. In addition, informed consent (informal written confirmation) was obtained from the cat caregivers—both from private households and the animal shelter—allowing the behavioural observations to be conducted on private property and agreeing to participate in the study with their cats. Information on the sex of the human participants was provided on a voluntary basis. No human participants were minors at the time of testing. No further data were collected from the human participants. All humans and animals are treated anonymously.

## 3. Results

### 3.1. Descriptive Statistics

A total of 82 cats and their primary caregivers participated in the SST study, comprising 15 cats from animal shelters and 67 from private homes. Detailed sociodemographic information is provided in Table 1. The median age of all cats was 4.25 years; shelter cats had a lower median age of 2.08 years, whereas privately owned cats had a median age of 4.50 years. Among all cats, three were unneutered (two females and one male); the majority (56 out of 82) were neutered across both sexes, and 23 owners did not provide information on their cats’ neuter status. Information regarding the human participants indicates that six primary caregivers identified as male, 54 as female, and 22 did not specify their gender. The sample size, median, mean, and interquartile ranges, as well as the standard deviation for the assessed behavioural categories, are presented in Table 2 and Table 3.

### 3.2. LMM1 Results for Video Sequence and Living Environment

Exploratory behaviour: Regarding the fixed effects, we found a significant difference in the cats’ exploratory behaviour between video sequences 1 and 3 (b = −12.39, *p* = 0.022), as well as between sequences 1 (Familiar person and cat) and 4 (Familiar person, stranger, and cat) (b = −13.88, *p* = 0.010), independent of the environment. The random intercept for individual differences accounted for 63.90% of the variance (standard deviation 41.35), indicating high variability in duration baseline of behaviour across the group of cats. The pairwise comparisons of the post hoc tests revealed a significant difference between private homes and animal shelters for video sequence 3 (Stranger and cat) (b = −34.77, *p* = 0.020), 4 (Familiar person, stranger, and cat) (b = −29.53, *p* = 0.047) and 5 (Familiar person and cat) (b = −31.40, *p* = 0.035), as well as a significant difference between video sequence 2 and 3 (b = 14.82, *p* = 0.048) and 2 and 4 (b = 16.31, *p* = 0.022) for the group of cats from private homes.

Play behaviour: Regarding the fixed effects, we found a significant difference between the cats’ play behaviour in video sequences 1 and 4 (b = 0.49, *p* = 0.015), regardless of the environment. The random intercept for individual differences accounted for 61.70% of the variance (standard deviation 1.50), indicating high variability in duration baseline of play behaviour across the group of cats. The pairwise comparisons of the post hoc tests revealed a significant difference between private homes and animal shelters for video sequence 4 (b = 1.38, *p* = 0.018), as well as a significant difference between 2 and 4 (b = −0.76, *p* = 0.002) for the group of cats from private homes. Results were derived from log-transformed data—estimates were subsequently back-transformed for presentation, as seen in Table 4.

Passive behaviour: Regarding the fixed effects, we found a significant difference between the cats’ passive behaviour in video sequences 1 and 3 (b = 10.91, *p* = 0.050), regardless of the environment, as well as a significant difference between the groups of cats from shelters and private homes (b = −38.74, *p* = 0.006). The random intercept for individual differences accounted for 57.30% of the variance (standard deviation 37.14), indicating high variability in duration baseline of passive behaviour across the group of cats. The pairwise comparisons of the post hoc tests revealed a significant difference between private homes and animal shelters for video sequence 1 (b = 38.70, *p* = 0.006), 2 (b = 37.60, *p* = 0.008), 3 (b = 30.40, *p* = 0.032), and 5 (b = 30.20, *p* = 0.033).

Behaviour by the door: Regarding the fixed effects, we found a significant difference between the cats’ behaviour by the door in video sequences 1 and 3 (b = 0.66, *p* = 0.002) and in 1 and 4 (b = −0.49, *p* = 0.022), regardless of the environment. We also found a significant effect for cats from shelters for video sequence 1 and 5 (b = 1.00, *p* = 0.05). The random intercept for individual differences accounted for 45.70% of the variance (standard deviation 1.13), indicating high variability in duration baseline of behaviour across the group of cats. The pairwise comparisons of the post hoc tests revealed a significant difference between private homes and animal shelters for video sequence 3 (b = 0.95, *p* = 0.049), as well as for video sequence 2 and 3 (b = −0.90, *p* < 0.001), 3 and 4 (b = 1.15, *p* < 0.001), and 3 and 5 (b = 0.79, *p* = 0.002) for the group of cats from private homes. Results were derived from log-transformed data—estimates were subsequently back-transformed for presentation, as seen in Table 4.

Physical contact: Regarding the fixed effects, we found a significant difference in physical contact for video sequences 1 and 3 (b = −0.58, *p* = 0.010), 1 and 4 (b = −0.76, *p* < 0.001), and 1 and 5 (b = −0.70, *p* = 0.002), regardless of the environment. The random intercept for individual differences accounted for 34.90% of the variance (standard deviation 0.95), indicating moderate variability in duration baseline of physical contact across the group of cats. The pairwise comparisons of the post hoc tests revealed a significant difference between cats from private homes and animal shelters for video sequence 5 (b = −0.98, *p* = 0.033), as well as a significant difference between video sequence 1 and 4 (b = 0.76, *p* = 0.007) for the group of cats from private homes. Table 4 illustrates the results of this analysis. Results were derived from log-transformed data—estimates were subsequently back-transformed for presentation. The graphical representations of behaviours across the video sequences, grouped by living environment, are provided in the Supplementary Material.

### 3.3. LMM2 Results for Video Sequence, Living Environment and Outdoor Access

Exploratory behaviour: Regarding the fixed effects, a significant difference was observed in the cats’ exploratory behaviour for the group of cats from shelters and private homes, regardless of the video sequence (b = 45.18, *p* = 0.008), as well as for the group of cats with outdoor access to those without (b = 37.16, *p* = 0.003). We found a significant difference between the group of cats from shelters and those from private homes with no outdoor access (b = −92.78, *p* = 0.023). We also found significant differences between video sequences 1 (Familiar person and cat) and 3 (Stranger and cat) (b = −21.23, *p* = 0.046) and 1 and 4 (Familiar person, stranger and cat) (b = −26.58, *p* = 0.013), as well as between sequences 1 and 5 (Familiar person and cat) (b = −27.51, *p* = 0.010) for cats with access to the outdoors and those without. Lastly, we found a significant effect for cats from shelters with no outdoor access for video sequence 1 and 5 (b = 89.63, *p* = 0.010). The random intercept for individual cats accounted for 63.80% of the variance (standard deviation of 40.56), indicating high variability in duration baseline of exploratory behaviour across the group of cats. The pairwise comparisons of the post hoc tests revealed a significant difference between video sequence 2 (Familiar person, stranger, and cat) and 4 (b = 15.07, *p* = 0.040) for the group of cats from private homes.

Play behaviour: We found no significant effect for play behaviour for the fixed effects. The random intercept for individual cats accounted for 61.90% of the variance (standard deviation of 1.50) indicating high variability in duration baseline of play behaviour across the group of cats.

Physical contact: Regarding the fixed effects, a significant difference was observed for video sequence 1 and 4 (b = −0.75, *p* = 0.027) and 1 and 5 (b = −0.78, *p* = 0.021) regardless of the environment. We also found significant difference between the group of cats with outdoor access and those without (b = −0.77, *p* = 0.049). The random intercept for individual cats accounted for 31.40% of the variance (standard deviation of 0.88), indicating moderate variability in duration baseline of physical contact across the group of cats. The pairwise comparisons of the post hoc tests revealed a significant difference between cats from private homes and animal shelters for video sequence 5 (b = −1.55, *p* = 0.015), as well as a significant difference between video sequence 1 and 4 (b = 0.76, *p* = 0.008) and 1 and 5 (b = 0.71, *p* = 0.017) for the group of cats from private homes. Table 5 illustrates the results of the linear mixed models. Results were derived from log-transformed data—estimates were subsequently back-transformed for presentation. The graphical representations of behaviours across the video sequences, grouped by outdoor access are provided in the Supplementary Material.

### 3.4. Results GLMM for Video Sequence and Living Environment

Initiating contact—Approach: Regarding the fixed effects, a significant difference was observed in the cats’ initiating behaviour for video sequences 1 and 3 (b = −0.32, *p* = 0.014) and 1 and 4 (b = −0.71, *p* < 0.001), as well as 1 and 5 (b = −0.56, *p* < 0.001), regardless of the environment.

The random intercept accounted for 53.73% of the variance (standard deviation of 0.69), indicating low variability in duration baseline of approach behaviour across the group of cats. The pairwise comparisons of the post hoc tests revealed a significant difference between video sequence 1 and 4 (b = 0.71, *p* < 0.001), 1 and 5 (b = 0.56, *p* = 0.001) for the group of cats from private homes, and 1 and 4 (b = 1.12, *p* = 0.002) and 2 and 4 (b = 0.99, *p* = 0.011) for the group of shelter cats.

Initiating contact—Avoidance: We found no significant effect on play behaviour for the fixed effects. The random intercept for individual cats accounted for 50.04% of the variance (standard deviation of 1.01), indicating high variability in duration baseline of avoidance behaviour across the group of cats.

Vocalisation—Meow: Regarding the fixed effects, a significant difference was observed in the cats’ meow behaviour for video sequences 1 and 4 (b = −0.66, *p* = 0.044), regardless of the environment. The random intercept accounted for 92.77% of the variance (standard deviation of 2.10), indicating high variability in duration baseline of avoiding behaviour across the group of cats. The pairwise comparisons of the post hoc tests revealed no further significant effect.

Vocalisation—Purr: Regarding the fixed effects, a significant difference was observed in the cats’ purr behaviour between for the group of cats from shelters and private homes (b = 1.21, *p* = 0.011). We also found significant differences for video sequence 1 and 4 (b = −0.71, *p* = 0.002). The random intercept accounted for 57.87% of the variance (standard deviation of 1.39), indicating moderate variability in duration baseline of purr behaviour across the group of cats. The pairwise comparisons of the post hoc tests revealed a significant difference between private homes and animal shelters for video sequence 1 (b = −1.21, *p* = 0.011) and 2 (b = −1.30, *p* = 0.006), as well as a significant difference between video sequence 1 and 4 (b = 0.71, *p* = 0.018) for the group of cats from private homes and 1 and 3 (b = 0.81, *p* = 0.023), 1 and 4 (b = 0.98, *p* = 0.006), and 2 and 4 (b = 0.89, *p* = 0.020) for the group of shelter cats. Table 6 illustrates the results of the generalised linear mixed models. The graphical representations of behaviours across the video sequences, grouped by living environment, are provided in the Supplementary Material.

## 4. Discussion

### 4.1. Test Setting, Data Collection, and Methods

We used a structured five-phase video protocol to assess cat–caregiver pairs in private homes and animal shelters, recording seven attachment-related behaviours and analysing them using mixed models. The behavioural categories used to model attachment were developed and adapted from the framework established by Topál et al. on dogs in a laboratory [4]. One of Topál’s behavioural categories, ‘delay of contact seeking’, was excluded. In private homes, non-standardised spaces and transparent doors often allowed cats to see the familiar person or stranger before the test began. Due to many missing values, this measure was discarded. In contrast, the addition of the category ‘vocalisation’ appears to be useful; in particular, the sub-category ‘meow’ revealed several insights. The category ‘purr’ turned out to have the largest measurement error. Due to the low volume, the purring was often poorly captured by the camera, especially in video sequences 2 and 4, when the person filming does not interact with the cat and may be filming from a greater distance. After the first indications of this emerged from the pilot, this category was only counted.

A key objective of this study was to implement a comprehensive SST in a familiar environment, enabling the assessment of cats’ attachment to their caregiver versus a stranger while minimising environmental stress. Building on previous studies that employed either abbreviated protocols such as the Secure Base Test [13] or full SSTs with limited sample sizes [11,12], the present study applied the full SST protocol with a substantially larger sample of 82 cat–caregiver dyads. Based on earlier findings that cats often remained hidden under standard laboratory conditions [12,13], the assessment was conducted in familiar environments to reduce stress. This approach inevitably involved a trade-off, resulting in a reduced level of environmental standardisation. Given that no animals were hiding during the present study, we infer that a low-stress setting is ideal for assessing attachment behaviour. In a well-known living environment, not only is it likely that staying in familiar surroundings helps to alleviate stress, but also that the cat does not have to be transported, either after the test or for the journey there, so that its stress level is not increased prematurely. The relationship between stress and transport, as well as mechanisms for possible alleviation, are the subject of numerous studies [24,25,26,27]. Possible stressors include the camera, the stranger, and the general setting, e.g., the closed door and unusual behaviour on the part of the caregivers.

The relative distribution of behaviours also offers insights into the overall well-being of the animals: Behaviours such as playing, exploring, and physical contact are typically associated with positive mood, whereas hiding, staying by the door, growling, hissing, and avoidance indicate discomfort [28,29,30,31]. In our sample, exploratory behaviour was most common, followed by passive behaviour (mainly hiding and dozing), physical contact, play, and behaviour by the door. Approach behaviour occurred most frequently among the discrete behaviours, followed by meowing, purring, and avoidance. Hissing and growling were displayed by only one individual and were therefore not further analysed. Overall, the distribution of behaviours suggests that the animals did not experience substantial or prolonged stress. The presentation of the relative distribution of behaviours is provided in the Supplementary Material.

Conducting the study in private homes and shelter rooms introduced practical challenges due to the variability of room layouts. Manual filming limited the focus person’s ability to interact freely with the cat and reduced the precision of timing during transitions. Future studies could benefit from pre-inspecting test environments and installing fixed cameras to ensure continuous visibility of the animal throughout the procedure.

Smaller effects may also be underrepresented due to the still limited sample size. For example, there were only weak effects on play behaviour in the group of cats from the animal shelter, although it is noticeable that the cats in the shelters played very little or almost not at all throughout the entire course of the trial. With a group size of 15 cats, the power to show weak effects is limited. In the future, more emphasis should be placed on achieving a better balance of groups. Lastly, the distribution of age, sex, breed, and neutering status in cats may influence the attachment system towards their human caregiver.

Eligibility for participation required a minimum housing duration of three months in private homes and animal shelters. Most privately kept cats had been adopted as kittens and had lived in the same household ever since. Future research could examine housing duration more systematically as a potential moderating factor of attachment-related behaviour. Long-term studies on the development and flexibility of cat–human relationships are scarce; evidence from dogs suggests that attachment evolves over time, with stronger proximity-seeking and reduced stress in animals with longer-established bonds [32]. While Wedl et al. considered caregiving duration, it did not emerge in their results. Future work could therefore investigate how caregiving duration affects attachment in cats, particularly in those adopted as adults [33].

The continuous decline in initial approaches across video sequences may indicate fatigue and suggests potential order effects, meaning that behaviour may have depended on the sequence of the five-phase protocol. Future studies could address this by counterbalancing sequences, for example, by exposing half of the animals to an alternative order, as conducted in previous research [6,11,34]. Unlike previous trials [4,6,11,12], the cats in this study were familiar with the test room, which may explain the high level of exploratory behaviour. To enhance future designs, incorporating a novel stimulus, such as a toy or box, after a phase of separation could allow for an investigation of whether cats explore more in the presence of a familiar person versus a stranger (cf. Palmer & Custance [6]).

Finally, we aimed to incorporate the modelling of both individual differences and fixed effects that may influence attachment behaviour. Building upon the application of various statistical parametric and non-parametric methods in the studies by Topál et al. and Potter & Mills, we deliberately focused on modelling the independent variables for living environment and the constellation of persons in the room in relation to attachment behaviour in cats [4,11]. Additionally, we accounted for another independent variable of outdoor access, addressing variability associated with non-standardised living conditions in both environments.

### 4.2. LMM1 Effects of Video Sequence and Living Environment

Regarding the observed effects, the group of cats across the five video sequences exhibited distinct behavioural differences toward the familiar person and the stranger, depending on their respective living environment.

A comparison of the video sequences showed significant behavioural changes: in sequence 3 (Stranger and cat), cats exhibited more door-oriented and passive behaviour, were less exploratory, and had more physical contact with the stranger than in sequence 1 (Familiar person and cat). The effects here were in the low-to-moderate range when comparing the expected mean of the dependent variable for the reference group (estimates) to the intercept. In reunion in sequence 4 (Familiar person, stranger, and cat), cats played more, showed increased physical contact with the caregiver, and displayed reduced exploratory behaviour, though door-related behaviour remained elevated. In particular, the effect of physical contact in the context of reunion indicates that the cats, after a period of separation distress (increased behaviour by the door), sought physical closeness to the caregiver, both of which can be considered as criteria for secure attachment [1,2]. This interpretation is supported by the comparison between video sequences 1 (Familiar person and cat) and 5 (Familiar person and cat), where cats also spent significantly more time in physical contact with the familiar person.

At first, it seems surprising that the cats also show more physical contact with the stranger in video sequence 3. One interpretation could be that the cats might have felt insecure when the familiar person left the room and approached the door with increased searching behaviour. At the same time, the cats explored the stranger to find out whether she or he is an interesting interaction partner or a trustworthy source of protection in the absence of the attachment figure.

Similarly, Podberscek et al. found that cats initially showed more direct contact with a stranger than with their caregiver, an effect that diminished with repeated exposure [35]. In contrast, Potter & Mills found no differences in cats’ affiliative behaviour towards a stranger when the caregiver was absent, aside from increased vocalisation towards the caregiver [11]. Edwards et al., using a modified SST in a laboratory setting, reported longer physical contact with the caregiver, though their use of simple mean comparisons limited contextual interpretation [12]. Given the small sample sizes in several of these studies, further research is needed to clarify whether cats reliably seek physical contact with a stranger in the caregiver’s absence. The relatively low ICC of 0.35 for physical contact suggests moderate individual variability, with most variance explained by situational factors. This supports the view that physical contact reflects a general attachment response, influenced by context and interaction partner. Increases in exploratory and contact in the presence of the caregiver further suggest the use of the familiar person as a secure base, consistent with the core criteria of secure attachment [1,2].

Two other noteworthy observations should be discussed here: firstly, there was a significant effect on the group of cats from the animal shelter for video sequence 5 for the behaviour by the door. This group shows increased behaviour by the door in the test compared to cats in private homes and at other test times. Since cats in the shelter mostly have a monotonous daily routine, the events around the door seem to exert a strong stimulus on the cats in video sequence 5 as well, after the known person enters and the stranger leaves. The reason for this could be that the entry of familiar persons is usually associated with food or toys, or that the quality of new stimuli, all of which come through one door, is highly valued by the animals.

Secondly, shelter cats exhibited significantly less passive behaviour than privately kept cats, with a mean difference of 38.74 s—representing the strongest effect in the analysis and lending substantial support to the initial hypothesis. Increased passivity or excessive sleep may function as an adaptive response to overstimulation, with sleep helping to conserve energy and reduce exposure to external stimuli. As Leyhausen observed, cats tend to withdraw and sleep when coping options are limited, a pattern likely more pronounced in privately housed cats due to their richer and more dynamic environments [36]. In contrast, the relative monotony and predictability of the shelter setting, characterised by limited human interaction and lower environmental complexity, may reduce passivity and increase responsiveness to novel stimuli. Specifically, it suggests that the characteristics of the shelter environment, particularly the lack of stimuli and monotony, play a significant role in shaping the observed behaviour. Numerous studies have shown that environmental enrichment in shelters improves welfare in both cats and other animals [37,38,39,40,41,42,43,44,45,46,47,48]. In this context, it has also been shown that general enrichment, but especially human interaction with shelter cats, can be a particular lever for increasing their well-being.

Although not statistically significant, the reduced play behaviour observed in shelter cats is noteworthy and may indicate a lack of the ‘relaxed field’ typically associated with play [49]. Previous studies similarly found that cats prefer to play with familiar individuals and avoid unfamiliar ones, highlighting a potential welfare disadvantage for shelter cats with inconsistent human contact [12,50]. Volunteers could be an important addition, provided they come back consistently and can establish themselves as a permanent point of contact for cats in the shelter. Play behaviour, though complex, is widely recognised as an indicator of animal welfare, with a noted reduction in play in response to stress [28,50,51,52,53,54]. In conjunction with the present findings, this suggests that interaction with familiar individuals, as well as focused play, enhances the well-being of cats.

### 4.3. LMM2 Effects of Video Sequence, Living Environment, and Outdoor Access

In exploring potential confounders or other independent variables that may influence feline behaviour in the SST, the pilot study provided a valuable insight: only cats from the animal shelter, all of which had no access to outdoor environments, were included. This prompted consideration of outdoor enclosures as a potential influencing factor. Previous research supports the fact that outdoor access can substantially affect feline behaviour [55,56,57,58,59]. Therefore, we recruited animals from an animal shelter with outdoor access and analysed the variable of outdoor access in relation to three behaviours for which we anticipated an influence: exploratory behaviour, play behaviour, and physical contact. The results indicate that there are indeed effects within this sample, particularly for exploratory behaviour and physical contact. The group of indoor cats showed significantly higher physical contact independent of the focal person, with a median effect of 58.41% compared to outdoor cats (cf. similar effects described by various authors [60,61,62,63,64]). Similarly, the significant effects observed for video sequence 4 (Reunion. Familiar person, stranger, and cat) and 5 (Familiar person and cat) when compared to video sequence 1 (Familiar person and cat) for cats, regardless of outdoor access, were confirmed, as seen in the LLM1 analysis. In contrast, no significant result was found for video sequence 3 (Stranger and cat) when compared to video sequence 1. However, the trend identified in the LLM1 analysis is also apparent here. The slightly lower ICC for physical contact suggests that the LMM2 analysis, which includes the variable outdoor access, offers a more precise explanation of physical contact behaviour. The most distinct pattern emerges in the context of exploratory behaviour, where the variable outdoor access provides the clearest effects based on the available results. This highlights the two primary differences observed: first, cats from animal shelters, and second, cats without access to outdoor environments both show significantly longer exploratory behaviour to the reference group (private homes and outdoor cats), with 45.18 and 37.16 s. These effects can be considered strong to very strong. Given the clear nature of these connections, it is not surprising that, in combination with indoor cats, the significant results for video sequences 3 and 4 and the trend for sequence 5 from the LMM1 analysis are achieved here in a similar way. We found a significantly shorter exploratory behaviour between video sequences 3, 4, and 5 compared to 1 for the group of indoor cats. This indicates that, overall, the group of indoor cats tends to exhibit longer exploratory behaviour; the exact behaviour is significantly lower in these phases of the test. From the authors’ point of view, this confirms two working assumptions: Firstly, exploratory behaviour can only be performed safely from a secure base. It seems that, with the familiar person’s leaving, this base is no longer present, so the cats significantly restrict the behaviour. The fact that physical contact increases at the same time as the familiar person reappears in sequences 4 and 5 supports the hypothesis that the familiar person is used to re-establish the safe base after the separation.

Secondly, living environment influences exploratory behaviour. Indoor cats conduct all exploration within the home, whereas cats with outdoor access engage mostly in roaming and hunting outside in combination with exploratory behaviour, focusing on rest and human interaction indoors. Parker et al. found that indoor cats exhibit more exploratory behaviour during the night within the home environment compared to outdoor cats [65]. Similarly, Scandurra et al. observed that indoor cats display more exploratory behaviour and experience lower stress levels than their outdoor counterparts when faced with an impossible task [66]. In contrast, Smit et al. (2024) reported no significant difference in exploratory behaviour between indoor and outdoor cats [67]. Our findings suggest that outdoor access does indeed influence exploratory behaviour in this study, and potentially other behavioural aspects as well. Future research will be required to further elucidate these effects.

Two further significant results must be considered in this context: we also found significant effects for the group of animal shelter cats in combination with indoor cats with a strongly negative correlation, i.e., greatly reduced exploratory behaviour compared to the reference group (private home and outdoor), as well as a strongly positive correlation, i.e., increased exploratory behaviour for the combination of cats from animal shelters, indoor cats, and the video sequence 5. In the pilot study, we found that shelter cats with outdoor enclosures could provide valuable SST data. We therefore selected ‘Tierheim Jena’, where most cats have controlled access to 25 m^2^ outdoor enclosures with varied areas and climbing structures. Only two cats were in quarantine without outdoor access. All tested cats were healthy. Due to the limited and unique sample, results are not representative. These findings should be considered in context. Individually housed cats may have had limited opportunities to form secure attachments, reducing exploratory behaviour. With less overall interaction, the quality of human interaction—especially when the familiar person returned—likely played a dominant role, explaining the observed behaviours.

### 4.4. GLMM Effects of Video Sequence and Living Environment

Consistent with LMM1 and LMM2, the GLMM revealed behavioural differences across video sequences and between shelter and home cats. The most pronounced behavioural differences emerged between sequence 1 (alone with the familiar person) and sequence 4 (reunion with the familiar person), with cats showing significantly more initiating behaviours, increased vocalisation, and purring in sequence 4—responses commonly associated with feline well-being [68,69,70,71,72]. In fact, previous work shows that meowing is a behaviour that cats express primarily towards their caregivers [11,13,70]. Significant increases in approaching behaviour were also observed in sequences 3 (alone with stranger) and 5 (alone with familiar person) compared to sequence 1, suggesting that exposure to the stranger may have enhanced the cats’ appreciation of proximity to their caregiver, which supports the attachment criteria of ‘separation distress’ as well as the cats using their caregivers as a ‘safe haven’ to return to [1,2]. Finally, the analysis of the dependence of behaviour on the living environment revealed a significant difference between the general purring behaviour of cats from animal shelters compared to those from private homes over the entire test. According to the model, the expected number of purring events for cats in private homes is 1.73 times per video sequence ($e^{0.55}\approx 1.73$). Cats in animal shelters show 5.78 purring events, a 3.34-fold increase compared to those in private homes. This effect may be classified as strong. Purring in cats is variably interpreted in the literature, often seen as an indicator of well-being, suggesting that the test provided a stimulating diversion for shelter cats [69,71]. Human interaction appears particularly important in shelters, a hypothesis supported by Gourkow et al., who found that routines including petting, play, and grooming increased levels of immune markers (S-IgA) and reduced upper respiratory disease [73]. Additional evidence shows that cats prefer social interaction, spending more time near attentive humans, and that interactions promoting choice, control, and play enhance cat–guardian relationships [74]. Alternatively, purring may serve as self-soothing; for example, labouring cat mothers purr despite pain. Thus, increased purring in shelter cats during the test could indicate stress, helping them calm themselves [68,75].

Finally, it is noteworthy that the Intraclass Correlation Coefficients (ICCs) for all four behavioural categories were found to be high to very high. In particular, the ICC of 0.93 for meowing behaviour is exceptionally high. This indicates that 93% of the variance in meowing behaviour observed in this test can be attributed to stable individual differences between cats, with only 7% attributable to residual variation. These findings align with previous research showing that vocalisation in cats and dogs is influenced by personality, arousal, and the human interaction partner [70,76,77,78]. Although the concept of personality—defined as stable, trait-like patterns of behaviour that are consistent across time and contexts—differs markedly from the broader notion of individuality, which refers to consistent behavioural or physiological differences between individuals, it is noteworthy that Kalenta et al. identified a feline personality dimension termed ‘quietness’ and emphasised that vocalisation patterns in cats appear to be strongly influenced by personality [79]. Similarly, the behaviours of approaching and avoiding contact, as well as purring, exhibited relatively high Intraclass Correlation Coefficients (ICCs), with values greater than or equal to 0.50. These results suggest that both the way cats seek social interaction and their vocalisation patterns are strongly influenced by individual differences. It appears that some cats consistently use vocalisation as a strategy to attract attention across various contexts, whereas others remain characteristically quiet. These patterns provide evidence for stable personality traits that shape cats’ responses to their environment, with research identifying several distinct feline personality dimensions. Bennett et al., Gartner et al., and Litchfield et al. [80,81,82] show that cats can be categorised into four or five core personality dimensions, similar to canine research [83]. ‘Shyness’ is one such dimension and may moderate responses in the SST. Future studies combining SST with a personality inventory could clarify the influence of personality on behavioural variability and high ICC values.

However, feline personality does not operate in isolation; the caregiver’s own characteristics can influence the expression of these traits and thus must also be taken into account. Growing evidence highlights that caregiver attributes constitute a significant factor influencing feline behaviour and the quality of the cat–human relationship. Research demonstrates that caregiver personality traits, emotional dispositions, and interaction styles can shape cats’ stress responses, exploratory tendencies, social engagement, and the perceived strength of the bond [57,61,84]. Moreover, psychological characteristics and the mental well-being of caregivers have been shown to predict attachment styles towards companion animals, with human and animal traits interacting to produce distinct relational patterns [85]. Evidence from dog-owner research further underscores that the caregiver’s attachment profile itself can modulate the animal’s behavioural and emotional responses, suggesting that human-side variables may systematically influence attachment outcomes across species [86]. Taken together, these findings indicate that the attachment behaviours observed in assessments such as the adapted SST are likely shaped not only by feline traits and environmental context but also by caregiver-specific psychological and relational factors. Incorporating such human-associated variables in future studies would enable a more comprehensive understanding of the mechanisms underpinning attachment in cat–caregiver dyads and support a more accurate interpretation of SST-derived behavioural patterns.

## 5. Conclusions

The results of this study indicate that living environment and established bond to a person influence feline attachment behaviour, particularly regarding cats exhibiting context-dependent patterns consistent with core criteria of secure attachment—such as increased physical contact, vocalisation, exploratory behaviour during reunions, and increased passive and door-oriented behaviour during periods of separation. Environmental variables, particularly the distinction between private home and animal shelter, as well as access to outdoor areas, were found to moderate behavioural responses: Animal shelter cats exhibited reduced exploratory and play behaviour; indoor cats demonstrated significantly higher levels of exploration, likely due to the restricted nature of their environment.

Moreover, the consistent manifestation of behaviours such as meowing and contact initiation across different test conditions suggests the influence of stable individual differences, likely underpinned by enduring individuality or personality traits, in shaping cats’ responsiveness to social stimuli and human interaction.

### 5.1. Limitations and Future Research

There was no systematic investigation into the impact of caregiving duration on attachment behaviours. Future research could investigate the influence of caregiving duration on attachment behaviours in cats. Furthermore, while the standardisation of test environments was deliberately suspended to reduce stress and improve ecological validity, this decision inherently implies a limitation due to the variability introduced by the different room layouts in private homes and animal shelters. Additionally, future studies should explore the impact of environmental standardisation, potentially using fixed cameras and pre-inspected test environments, to further minimise variability and enhance the reliability of attachment assessments. A potential order effect may have influenced the results, as the proportion of cats that initially approached the focus person decreased steadily across the video sequences, suggesting possible fatigue or habituation. Future studies should therefore counterbalance the sequence of phases. Moreover, incorporating a feline personality inventory in future data collection could yield valuable insights into behavioural differences among the cats. At the same time, caregiver influences should be acknowledged as a potential limitation, emphasising the need for future studies to account more rigorously for human-derived variability, for instance, by including a prior personality assessment such as the Big Five Inventory. Lastly, it would be advisable to aim for more balanced sample sizes across groups. In the present study, for example, differences in age between shelter and privately owned cats may have influenced the results—particularly if variables such as age were not included as potential confounders in the statistical models. Other potential confounding variables that may have influenced the observed effects include the animals’ sex, neuter status, and breed, as well as the sex of the caregiver. Following the findings of Topál et al. [4], who reported no significant effects of sex, age, housing conditions, or breed on most behavioural variables in dogs, we refrained from including these factors directly into the model. Future research should aim to control for these factors or investigate their individual and combined impact more systematically.

### 5.2. Implications for the Keeping of Cats in Animal Shelters and Private Homes

The multifaceted findings of this study allow us to draw specific implications for the management of cats in living environments, private homes, and shelter environments:

1. Focus on human interaction: Human interaction can serve as a compensatory factor for shelter cats lacking outdoor access or facing monotonous routines. However, it cannot fully replace the benefits of free-roaming environments. Attachment theory suggests that long-term human godparentships, such as defined cat sitters or volunteers in shelters, could be beneficial for attachment-hungry cats. These interactions, supported by the biophilia effect, can strengthen bonds and benefit both cats and humans [33,62,63,64,87,88,89].
2. Improving shelter environments: Cats in shelters may especially benefit from engaging in play behaviour. By creating a relaxed field with open, stimulating surroundings is crucial for promoting natural play behaviour in cats [49,53,54,90,91]. While shelter layouts are often constrained by limited resources, the low play levels observed in this study suggest the need for more space and opportunities for playful activities. A lack of suitable play areas can reduce behavioural diversity and increase stress [38,40,41,42,43,47,48,92,93,94,95]. Such changes could improve both the emotional well-being and adoption prospects of cats, with spatial enrichment being a practical and effective solution.
3. Living environment matters: The debate over outdoor access for cats involves balancing concerns about wildlife protection, parasite prevention, and behavioural needs. Both indoor and outdoor environments significantly impact cat well-being [58,65,96]. This study suggests that indoor cats should be encouraged to explore, while outdoor cats should receive focused physical contact and affection, tailored to their individual needs and temperament.

## Figures and Tables

**Table 1:** Cat sample socio-demographics.

Characteristic	*n*	Characteristic	*n*	Characteristic	*n*
Breed		Sex		Multi-cat home	
European Shorthair (ESH)	46	Female	47	Single-cat	19
Siamese	2	Male	35	Multi-cat	63
British Shorthair (BSH)	5	Neuter status			
Maine Coon	4	Intact	3		
Persian	5	Neutered	56		
Mixed breed	20	Not available	23		
Age (years)		Environment			
<1 year	7	Private household	67		
1–3	32	Shelter	15		
4–6	13	Outdoor access			
7–9	18	Outdoor	43		
>10	12	Indoor	39		

**Table 2:** Behaviour categories measured in duration(s).

			Video Sequence 1		Video Sequence 2		Video Sequence 3		Video Sequence 4		Video Sequence 5	
**Living Environment**	**Behaviour**	***n***	**Mean ± SD**	**Median (Q1–Q3)**	**Mean ± SD**	**Median (Q1–Q3)**	**Mean ± SD**	**Median (Q1–Q3)**	**Mean ± SD**	**Median (Q1–Q3)**	**Mean ± SD**	**Median (Q1–Q3)**
Private Home	physical contact	67	43.9 ± 45.2	33 (9–63)	35.1 ± 33.9	25 (8–58)	26.6 ± 28.2	16 (3–44)	28.1 ± 34.9	11 (1–40.5)	28.1 ± 31.6	15 (0–48.5)
	exploratory behaviour	67	70 ± 50.4	70 (29.5–109.5)	72.4 ± 55.1	65 (25.5–114)	57.6 ± 46.7	43 (21–92.5)	56.1 ± 44	60 (12.5–81.5)	60.5 ± 49.1	49 (17–99.5)
	passive behaviour	67	43.7 ± 48.7	27 (5–63.5)	52.4 ± 51.4	38 (9.5–86.5)	54.6 ± 50.7	44 (13–86)	51.6 ± 52.3	41 (8–72)	52.9 ± 55.8	31 (7–89.5)
	play behaviour	67	21.6 ± 37.9	0 (0–34.5)	18.1 ± 32.9	0 (0–29)	26 ± 39.4	0 (0–53)	38.3 ± 50.7	0 (0–75)	27.8 ± 47.1	0 (0–48.5)
	door behaviour	67	17.6 ± 30.9	0 (0–23.5)	14.9 ± 29.9	0 (0–21)	28.1 ± 37.1	13 (0–40)	11.9 ± 23.5	0 (0–8)	15.8 ± 28.2	0 (0–17.5)
Animal Shelter	physical contact	15	85.1 ± 62.1	93 (23.5–146.5)	67.8 ± 59.7	66 (5–116)	59.9 ± 70.4	18 (4–109.5)	36.8 ± 41.6	20 (5.5–54)	58 ± 55.6	37 (14–90.5)
	exploratory behaviour	15	87.2 ± 54.9	73 (61.5–125)	80.9 ± 73.5	53 (15.5–160)	92.3 ± 59.4	107 (31–123)	85.6 ± 64.9	71 (26.5–136.5)	91.9 ± 55.8	94 (59–121.5)
	passive behaviour	15	4.9 ± 12.3	0 (0–5)	14.9 ± 39.6	0 (0–5.5)	24.2 ± 36.8	0 (0–50)	27.7 ± 34.6	12 (0–48.5)	22.7 ± 35.1	10 (0–37)
	play behaviour	15	6.7 ± 21.7	0 (0–0)	7.7 ± 19.5	0 (0–1.5)	13.8 ± 40.1	0 (0–3.5)	0.3 ± 0.9 *	0 (0–0) *	5.3 ± 14.9	0 (0–0.5)
	door behaviour	15	8.1 ± 21.1	0 (0–4)	9.8 ± 30.1	0 (0–0)	11.3 ± 20	0 (0–14)	10 ± 34.7	0 (0–1)	18.5 ± 32.3	10 (0–17)

* for play behaviour in video sequence 4, the actual sample size is *n* = 11.

**Table 3:** Behaviour categories measured in frequency (number).

			Video Sequence 1		Video Sequence 2		Video Sequence 3		Video Sequence 4		Video Sequence 5	
Living Environment	Behaviour	*n*	Mean ± SD	Median (Q1–Q3)	Mean ± SD	Median (Q1–Q3)	Mean ± SD	Median (Q1–Q3)	Mean ± SD	Median (Q1–Q3)	Mean ± SD	Median (Q1–Q3)
Private Home	hiss	67	0 ± 0	0 (0–0)	0 ± 0.1	0 (0–0)	0 ± 0	0 (0–0)	0 ± 0.2	0 (0–0)	0 ± 0	0 (0–0)
	growl	67	0 ± 0.1	0 (0–0)	0 ± 0.1	0 (0–0)	0 ± 0.4	0 (0–0)	0 ± 0.4	0 (0–0)	0 ± 0.4	0 (0–0)
	approach	15	7.2 ± 6	6 (4–10)	6.4 ± 5.3	6 (4–8)	5 ± 4.7	4 (2–6)	3.6 ± 4.1	2 (0–6)	4.4 ± 5.5	4 (0–6)
	avoidance	67	1 ± 1.6	0 (0–2)	1.1 ± 1.5	1 (0–2)	1 ± 1.3	1 (0–2)	0.9 ± 1.2	0 (0–2)	0.7 ± 1.1	0 (0–1)
	meow	67	2.5 ± 7.3	0 (0–2)	2.2 ± 5.8	0 (0–2)	2.8 ± 9.7	0 (0–0)	1.3 ± 4.7	0 (0–0)	1.9 ± 5	0 (0–0)
	purr	67	1.2 ± 1.6	0 (0–2)	1 ± 1.4	0 (0–2)	0.8 ± 1.4	0 (0–2)	0.6 ± 1.2	0 (0–1)	1.1 ± 1.6	0 (0–2)
Animal Shelter	hiss	15	0.2 ± 0.8	0 (0–0)	0.1 ± 0.3	0 (0–0)	0 ± 0	0 (0–0)	0.1 ± 0.3	0 (0–0)	0 ± 0	0 (0–0)
	growl	15	0 ± 0	0 (0–0)	0.1 ± 0.3	0 (0–0)	0.1 ± 0.3	0 (0–0)	0 ± 0	0 (0–0)	0 ± 0	0 (0–0)
	approach	15	13.3 ± 11.2	10 (4–24)	11.9 ± 12	12 (0–21)	7.2 ± 8.9	2 (0–13)	4.4 ± 7.1 *	1 (0–3.5) *	7.5 ± 7.9	6 (1–11)
	avoidance	15	2.7 ± 4.3	0 (0–4)	1.6 ± 3.6	0 (0–1)	2 ± 3.1	0 (0–2.5)	1.1 ± 3.4	0 (0–0.8)	1.5 ± 2.2	1 (0–2.5)
	meow	15	1.5 ± 3.6	0 (0–0)	1.3 ± 4.6	0 (0–0)	2.9 ± 11.4	0 (0–0)	1.3 ± 4.1	0 (0–0)	0.5 ± 1.6	0 (0–0)
	purr	15	4.1 ± 3.7	4 (1–6)	3.9 ± 4.5	2 (0–7)	1.9 ± 2.7	2 (0–2)	1.6 ± 2.5	0 (0–2)	2.3 ± 2.5	2 (0–4)

* for approach in video sequence 4, the actual sample size is *n* = 14.

**Table 4:** LMM1 results for video sequence and living environment.

	**Play** **Behaviour**	**Behaviour** **by the Door**	**Physical Contact**	**Exploratory** **Behaviour**	**Passive** **Behaviour**
**Predictors**	**Estimates**	**Estimates**	**Estimates**	**Estimates**	**Estimates**
(Intercept)	4.41 *** (1.02)	4.86 *** (1.00)	22.14 *** (4.34)	69.96 *** (6.32)	43.67 *** (6.00)
Environment [Animal Shelter]	0.40 (0.22)	0.50 (0.24)	1.73 (0.79)	17.24 (14.77)	−38.74 ** (14.02)
Video Sequence [2]	0.76 (0.15)	0.79 (0.17)	0.84 (0.19)	2.43 (5.36)	8.76 (5.54)
Video Sequence [3]	1.24 (0.25)	1.93 ** (0.41)	0.56 ** (0.12)	−12.39 * (5.36)	10.91 * (5.54)
Video Sequence [4]	1.64 * (0.33)	0.61 * (0.13)	0.47 *** (0.10)	−13.88 * (5.36)	7.93 (5.54)
Video Sequence [5]	0.97 (0.20)	0.87 (0.19)	0.50 ** (0.11)	−9.49 (5.36)	9.27 (5.54)
Environment [Animal Shelter] × Video Sequence [2]	1.55 (0.73)	0.90 (0.45)	0.80 (0.42)	−8.70 (12.53)	1.17 (12.96)
Environment [Animal Shelter] × Video Sequence [3]	1.24 (0.59)	0.77 (0.39)	0.95 (0.50)	17.52 (12.53)	8.36 (12.96)
Environment [Animal Shelter] × Video Sequence [4]	0.63 (0.32)	1.30 (0.65)	0.87 (0.46)	12.28 (12.53)	14.87 (12.96)
Environment [Animal Shelter] × Video Sequence [5]	1.06 (0.50)	2.73 * (1.36)	1.54 (0.81)	14.16 (12.53)	8.53 (12.96)
**Random Effects**					
σ^2^	1.38	1.52	1.68	962.07	1029.09
τ_00_	2.21	1.28	0.90	1710.05	1379.57
ICC	0.62	0.46	0.35	0.64	0.57
N	82	82	82	82	82
**Observations**	406	410	410	410	410

* *p* < 0.05 ** *p* < 0.01 *** *p* < 0.001.

**Table 5:** LMM2 results for video sequence, living environment and outdoor access.

	Play Behaviour	Physical Contact	Exploration Behaviour
Predictors	Estimates	Estimates	Estimates
(Intercept)	3.65 *** (1.27)	33.83 *** (9.75)	49.43 *** (9.27)
Environment [Animal Shelter]	0.53 (0.33)	0.93 (0.49)	45.18 ** (16.85)
Video Sequence [2]	0.87 (0.26)	0.71 (0.24)	4.00 (7.88)
Video Sequence [3]	1.16 (0.35)	0.59 (0.20)	−0.67 (7.88)
Video Sequence [4]	1.75 (0.53)	0.47 * (0.16)	0.80 (7.88)
Video Sequence [5]	0.68 (0.21)	0.46 * (0.15)	5.70 (7.88)
Access [Indoor]	1.41 (0.66)	0.46 * (0.18)	37.16 ** (12.47)
Environment [Animal Shelter] × Video Sequence [2]	1.39 (0.76)	1.10 (0.68)	−16.38 (14.33)
Environment [Animal Shelter] × Video Sequence [3]	1.26 (0.70)	0.86 (0.53)	3.13 (14.33)
Environment [Animal Shelter] × Video Sequence [4]	0.55 (0.32)	0.85 (0.52)	−1.65 (14.33)
Environment [Animal Shelter] × Video Sequence [5]	1.20 (0.66)	1.64 (1.01)	−9.32 (14.33)
Environment [Animal Shelter] × Access [Indoor]	0.37 (0.56)	9.34 (11.77)	−92.78 * (40.52)
Video Sequence [2] × Access [Indoor]	0.78 (0.32)	1.35 (0.61)	−2.84 (10.60)
Video Sequence [3] × Access [Indoor]	1.14 (0.46)	0.89 (0.41)	−21.23 * (10.60)
Video Sequence [4] × Access [Indoor]	0.89 (0.36)	0.98 (0.45)	−26.58 * (10.60)
Video Sequence [5] × Access [Indoor]	1.91 (0.78)	1.16 (0.53)	−27.51 ** (10.60)
(Environment [Animal Shelter] × Video Sequence [2]) × Access [Indoor]	1.05 (1.39)	0.23 (0.33)	48.72 (34.44)
(Environment [Animal Shelter] × Video Sequence [3]) × Access [Indoor]	1.34 (1.78)	1.41 (2.09)	41.26 (34.44)
(Environment [Animal Shelter] × Video Sequence [4]) × Access [Indoor]	1.94 (3.14)	1.18 (1.74)	20.93 (34.44)
(Environment [Animal Shelter] × Video Sequence [5]) × Access [Indoor]	2.93 (3.88)	1.01 (1.50)	89.63 ** (34.44)
**Random Effects**			
σ^2^	1.37	1.71	930.65
τ_00_	2.24	0.78	1645.11
ICC	0.62	0.31	0.64
N	82	82	82
Observations	406	410	410

* *p* < 0.05 ** *p* < 0.01 *** *p* < 0.001.

**Table 6:** GLMM results for video sequence and living environment.

	Approach	Avoidance	Meow	Purr
Predictors	Incidence Rate Ratios	Incidence Rate Ratios	Incidence Rate Ratios	Incidence Rate Ratios
(Intercept)	6.10 *** (0.76)	0.63 * (0.14)	0.57 (0.24)	0.55 * (0.14)
**Zero-Inflated Model**				
(Intercept)			0.17 ** (0.11)	
Environment [Animal Shelter]	1.39 (0.37)	1.98 (0.87)	0.20 (0.19)	3.34 * (1.58)
Video Sequence [2]	0.94 (0.11)	1.21 (0.26)	0.72 (0.20)	0.83 (0.16)
Video Sequence [3]	0.73 * (0.09)	1.13 (0.24)	0.78 (0.21)	0.68 (0.14)
Video Sequence [4]	0.49 *** (0.07)	0.88 (0.20)	0.52 * (0.17)	0.49 ** (0.11)
Video Sequence [5]	0.57 *** (0.08)	0.81 (0.19)	0.61 (0.17)	0.92 (0.18)
Environment [Animal Shelter] × Video Sequence [2]	0.94 (0.23)	0.47 (0.20)	1.40 (1.02)	1.10 (0.32)
Environment [Animal Shelter] × Video Sequence [3]	0.72 (0.20)	0.65 (0.27)	2.45 (1.88)	0.66 (0.22)
Environment [Animal Shelter] × Video Sequence [4]	0.67 (0.22)	0.39 (0.20)	2.23 (1.59)	0.76 (0.28)
Environment [Animal Shelter] × Video Sequence [5]	1.03 (0.28)	0.77 (0.33)	0.90 (0.72)	0.60 (0.19)
**Random Effects**				
σ^2^	0.41	1.15	0.34	1.41
τ_00_	0.48	1.16	4.36	1.93
ICC	0.54	0.50	0.93	0.58
N	82	82	82	82
Observations	409	409	410	410

* *p* < 0.05 ** *p* < 0.01 *** *p* < 0.001.

## Data Availability

All data supporting the findings of this study, as well as the R code used for statistical analyses, are available in the Supplementary Material.

## Supplementary Materials

The following supporting information can be downloaded at https://www.mdpi.com/article/10.3390/ani15243521/s1, File S1. Supplementary Material: Additional graphics, equipment data, used R packages. File S2. R Code. File S3. Data set.
