# Supplementary material for: Impact of Living Environment on Attachment Behaviour in Domestic Cats from Private Homes and Shelters
Source: Animals (Basel). 2025 Dec 5;15(24):3521. doi: 10.3390/ani15243521 (PMC12729804; doi:10.3390/ani15243521)
Supplement: Supplementary file 1 [file animals-15-03521-s001.zip › S1. Supplememtary Material.pdf]

S1. Supplementary Material

1. Camera Data

The videos were recorded using mobile phone cameras and digital cameras.

Mobile phone cameras: Apple iPhone 6, Apple iPhone X, Apple iPhone 15, Samsung Galaxy A5

Digital cameras: Canon EOS 1100D, Panasonic Lumix HD AVCHD Lite

2. Relative proportions of behaviours in total

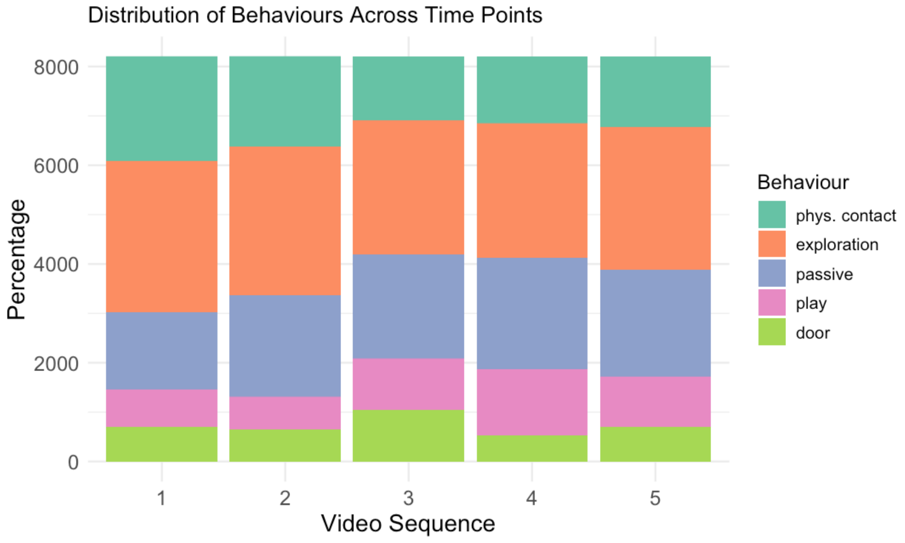

Figure S1. distribution of relative proportions of behaviours

3. Behaviours across the video sequences, separated by living environment/ outdoor access

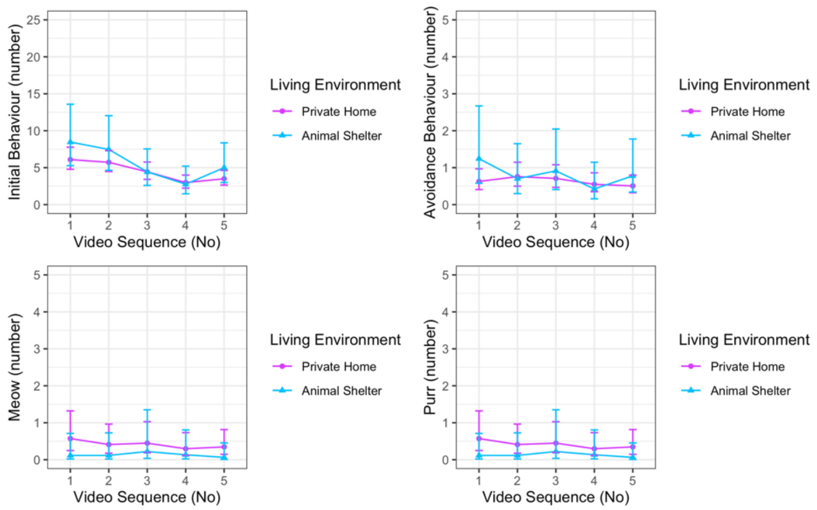

Figure S2. Graphs of the behaviours across the video sequences, separated by living environment for GLMM.

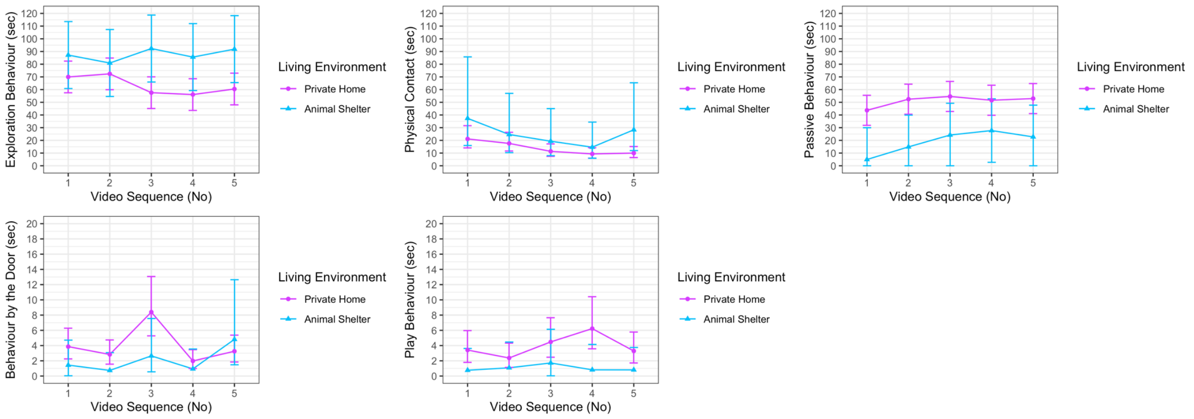

Figure S3 Graphs of the behaviours across the video sequences, separated by living environment for LMM1.

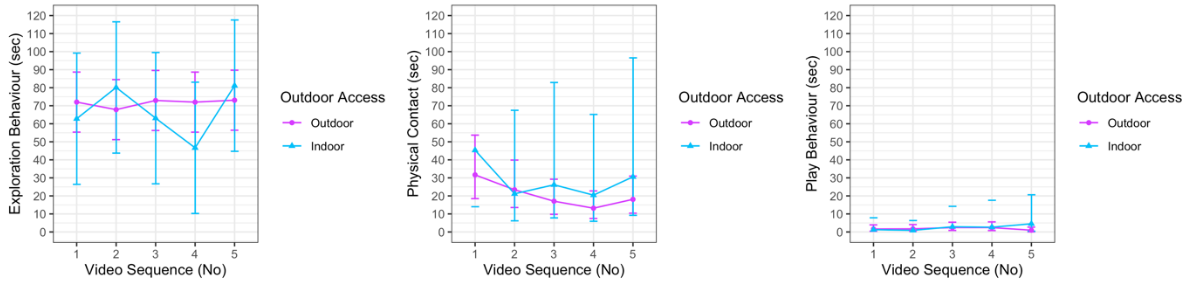

Figure S4. Graphs of the behaviours across the video sequences, separated by outdoor access for LMM2.

4. Statistics

4.1 Log-level calculated according to Rittmann et al.

Rittmann, O., Neunhoeffer, M., & Gschwend, T. (2023). How to improve the substantive interpretation of regression results when the dependent variable is logged. Political Science Research and Methods. https://doi.org/10.1017/psrm.2023.29

4.2 Unstandardised effect sizes reported according to Pek et al.

Pek, J., & Flora, D. B. (2018). Reporting effect sizes in original psychological research: A discussion and tutorial. Psychological Methods, 23(2), 208–225. https://doi.org/10.1037/met0000126

4.3 Citation of used R packages

Bates, D., Maechler, M., Bolker, B., & Walker, S. (2015). Fitting linear mixed-effects models using lme4. *Journal of Statistical Software, 67*(1), 1–48. https://doi.org/10.18637/jss.v067.i01

Ben-Shachar, M., Lüdecke, D., & Makowski, D. (2020). effectsize: Estimation of effect size indices and standardized parameters. *Journal of Open Source Software, 5*(56), 2815. https://doi.org/10.21105/joss.02815

Bolker, B., & Robinson, D. (2024). *broom.mixed: Tidying Methods for Mixed Models* (Version 0.2.9.6) \[R package]. https://CRAN.R-project.org/package=broom.mixed

Brunson, J. C. (2020). ggalluvial: Layered grammar for alluvial plots. *Journal of Open Source Software, 5*(49), 2017. https://doi.org/10.21105/joss.02017

Brunson, J. C., & Read, Q. D. (2023). *ggalluvial: Alluvial Plots in 'ggplot2'* (Version 0.12.5) \[R package]. http://corybrunson.github.io/ggalluvial/

Buuren, S. van, & Groothuis-Oudshoorn, K. (2011). mice: Multivariate imputation by chained equations in R. *Journal of Statistical Software, 45*(3), 1–67. https://doi.org/10.18637/jss.v045.i03

Chongsuvivatwong, V. (2022). *epiDisplay: Epidemiological Data Display Package* (Version 3.5.0.2) \[R package]. https://CRAN.R-project.org/package=epiDisplay

Delignette-Muller, M. L., & Dutang, C. (2015). fitdistrplus: An R package for fitting distributions. *Journal of Statistical Software, 64*(4), 1–34. https://doi.org/10.18637/jss.v064.i04

Firke, S. (2023). *janitor: Simple Tools for Examining and Cleaning Dirty Data* (Version 2.2.0) \[R package]. https://CRAN.R-project.org/package=janitor

Fox, J., & Weisberg, S. (2019). *An R companion to applied regression* (3rd ed.). Sage. https://socialsciences.mcmaster.ca/jfox/Books/Companion/

Gohel, D., & Skintzos, P. (2024). *flextable: Functions for Tabular Reporting* (Version 0.9.7) \[R package]. https://CRAN.R-project.org/package=flextable

Haman, J., & Avery, M. (2020). *ciTools: Confidence or Prediction Intervals, Quantiles, and Probabilities for Statistical Models* (Version 0.6.1) \[R package]. https://CRAN.R-project.org/package=ciTools

Hartig, F. (2022). *DHARMa: Residual Diagnostics for Hierarchical (Multi-Level / Mixed) Regression Models* (Version 0.4.6) \[R package]. https://CRAN.R-project.org/package=DHARMa

Hothorn, T., Hornik, K., van de Wiel, M. A., & Zeileis, A. (2006). A Lego system for conditional inference. *The American Statistician, 60*(3), 257–263. https://doi.org/10.1198/000313006X118430

Kassambara, A. (2023). *rstatix: Pipe-Friendly Framework for Basic Statistical Tests* (Version 0.7.2) \[R package]. https://CRAN.R-project.org/package=rstatix

Koller, M. (2016). robustlmm: An R package for robust estimation of linear mixed-effects models. *Journal of Statistical Software, 75*(6), 1–24. https://doi.org/10.18637/jss.v075.i06

Lenth, R. (2024). *emmeans: Estimated Marginal Means, aka Least-Squares Means* (Version 1.10.3) \[R package]. https://CRAN.R-project.org/package=emmeans

Lüdecke, D. (2024). *sjPlot: Data Visualization for Statistics in Social Science* (Version 2.8.16) \[R package]. https://CRAN.R-project.org/package=sjPlot

Lüdecke, D., Ben-Shachar, M., Patil, I., & Makowski, D. (2020). Extracting, computing and exploring the parameters of statistical models using R. *Journal of Open Source Software, 5*(53), 2445. https://doi.org/10.21105/joss.02445

Mollie, E. B., Kristensen, K., van Benthem, K. J., Magnusson, A., Berg, C. W., Nielsen, A., Skaug, H. J., Maechler, M., & Bolker, B. M. (2017). glmmTMB balances speed and flexibility among packages for zero-inflated generalized linear mixed modeling. *The R Journal, 9*(2), 378–400. https://doi.org/10.32614/RJ-2017-066

Müller, K., & Wickham, H. (2023). *tibble: Simple Data Frames* (Version 3.2.1) \[R package]. https://CRAN.R-project.org/package=tibble

Neuwirth, E. (2022). *RColorBrewer: ColorBrewer Palettes* (Version 1.1-3) \[R package]. https://CRAN.R-project.org/package=RColorBrewer

Pedersen, T. (2024). *patchwork: The Composer of Plots* (Version 1.3.0) \[R package]. https://CRAN.R-project.org/package=patchwork

Pruim, R., Kaplan, D. T., & Horton, N. J. (2017). The mosaic package: Helping students to 'think with data' using R. *The R Journal, 9*(1), 77–102.

Rigby, R. A., & Stasinopoulos, D. M. (2005). Generalized additive models for location, scale and shape (with discussion). *Applied Statistics, 54*(3), 507–554.

Robinson, D., Hayes, A., & Couch, S. (2024). *broom: Convert Statistical Objects into Tidy Tibbles* (Version 1.0.6) \[R package]. https://CRAN.R-project.org/package=broom

Rosseel, Y. (2012). lavaan: An R package for structural equation modeling. *Journal of Statistical Software, 48*(2), 1–36. https://doi.org/10.18637/jss.v048.i02

Singmann, H., Bolker, B., Westfall, J., Aust, F., & Ben-Shachar, M. (2024). *afex: Analysis of Factorial Experiments* (Version 1.4-1) \[R package]. https://CRAN.R-project.org/package=afex

Tierney, N., & Cook, D. (2023). Expanding tidy data principles to facilitate missing data exploration, visualization and assessment of imputations. *Journal of Statistical Software, 105*(7), 1–31. https://doi.org/10.18637/jss.v105.i07

Wickham, H. (2016). *ggplot2: Elegant graphics for data analysis*. Springer.

Wickham, H., Bryan, J. (2023). *readxl: Read Excel Files* (Version 1.4.3) \[R package]. https://CRAN.R-project.org/package=readxl

Wickham, H., François, R., Henry, L., Müller, K., & Vaughan, D. (2023). *dplyr: A Grammar of Data Manipulation* (Version 1.1.4) \[R package]. https://CRAN.R-project.org/package=dplyr

Wickham, H., Vaughan, D., & Girlich, M. (2024). *tidyr: Tidy Messy Data* (Version 1.3.1) \[R package]. https://CRAN.R-project.org/package=tidyr
