# Supplementary material for: Impact of Living Environment on Attachment Behaviour in Domestic Cats from Private Homes and Shelters
Source: Animals (Basel). 2025 Dec 5;15(24):3521. doi: 10.3390/ani15243521 (PMC12729804; doi:10.3390/ani15243521)
Supplement: Supplementary file 1 [file S2. R Code.pdf › S2. R Code.pdf]

KatzenSST

Libraries

```
library(readxl)
library(lavaan)
library(dplyr)
library(psych)
library(car)
library(rstatix)
library(coin)
library(tidyr)
library(psych)
library(epiDisplay)
library(mosaic)
library(afex)
library(effectsize)
library(emmeans)
library(broom)
library(broom.mixed)
library(DHARMa)
library(ggplot2)
library(janitor)
library(RColorBrewer)
library(patchwork)
library(mice)
library(naniar)
library(lme4)
library(sjPlot)
library(lme4)
library(robustlmm)
library(fitdistrplus)
library(ciTools)
library(gamlss)
library(glmmTMB)
library(parameters)
library(tidyr)
library(flextable)
library(ggplot2)
library(ggalluvial)
library(dplyr)
library(tidyr)
library(flextable)
library(tibble)
```

Zitieren

```
citation("mice")
```

```
To cite mice in publications use:

  Stef van Buuren, Karin Groothuis-Oudshoorn (2011). mice: Multivariate
  Imputation by Chained Equations in R. Journal of Statistical
  Software, 45(3), 1-67. DOI 10.18637/jss.v045.i03.

A BibTeX entry for LaTeX users is

  @Article{,
    title = {{mice}: Multivariate Imputation by Chained Equations in R},
    author = {Stef {van Buuren} and Karin Groothuis-Oudshoorn},
    journal = {Journal of Statistical Software},
    year = {2011},
    volume = {45},
    number = {3},
    pages = {1-67},
    doi = {10.18637/jss.v045.i03},
  }
```

```
citation("readxl")
```

```
To cite package 'readxl' in publications use:

  Wickham H, Bryan J (2023). _readxl: Read Excel Files_. R package
  version 1.4.3, <https://CRAN.R-project.org/package=readxl>.

A BibTeX entry for LaTeX users is

  @Manual{,
    title = {readxl: Read Excel Files},
    author = {Hadley Wickham and Jennifer Bryan},
    year = {2023},
    note = {R package version 1.4.3},
    url = {https://CRAN.R-project.org/package=readxl},
  }
```

```
citation("lavaan")
```

```
To cite lavaan in publications use:

  Yves Rosseel (2012). lavaan: An R Package for Structural Equation
  Modeling. Journal of Statistical Software, 48(2), 1-36.
  https://doi.org/10.18637/jss.v048.i02

A BibTeX entry for LaTeX users is

  @Article{,
    title = {{lavaan}: An {R} Package for Structural Equation Modeling},
    author = {Yves Rosseel},
    journal = {Journal of Statistical Software},
    year = {2012},
    volume = {48},
    number = {2},
    pages = {1--36},
    doi = {10.18637/jss.v048.i02},
  }
```

```
citation("dplyr")
```

```
To cite package 'dplyr' in publications use:

  Wickham H, François R, Henry L, Müller K, Vaughan D (2023). _dplyr: A
  Grammar of Data Manipulation_. R package version 1.1.4,
  <https://CRAN.R-project.org/package=dplyr>.

A BibTeX entry for LaTeX users is

  @Manual{,
    title = {dplyr: A Grammar of Data Manipulation},
    author = {Hadley Wickham and Romain François and Lionel Henry and Kirill Müller and Davis Vaughan},
    year = {2023},
    note = {R package version 1.1.4},
    url = {https://CRAN.R-project.org/package=dplyr},
  }
```

```
citation("psych")
```

```
To cite package 'psych' in publications use:

  William Revelle (2024). _psych: Procedures for Psychological,
  Psychometric, and Personality Research_. Northwestern University,
  Evanston, Illinois. R package version 2.4.6,
  <https://CRAN.R-project.org/package=psych>.

A BibTeX entry for LaTeX users is

  @Manual{,
    title = {psych: Procedures for Psychological, Psychometric, and Personality Research},
    author = {{William Revelle}},
    organization = {Northwestern University},
    address = {Evanston, Illinois},
    year = {2024},
    note = {R package version 2.4.6},
    url = {https://CRAN.R-project.org/package=psych},
  }
```

```
citation("car")
```

```
To cite the car package in publications use:

  Fox J, Weisberg S (2019). _An R Companion to Applied Regression_,
  Third edition. Sage, Thousand Oaks CA.
  <https://socialsciences.mcmaster.ca/jfox/Books/Companion/>.

A BibTeX entry for LaTeX users is

  @Book{,
    title = {An {R} Companion to Applied Regression},
    edition = {Third},
    author = {John Fox and Sanford Weisberg},
    year = {2019},
    publisher = {Sage},
    address = {Thousand Oaks {CA}},
    url = {https://socialsciences.mcmaster.ca/jfox/Books/Companion/},
  }
```

```
citation("rstatix")
```

```
To cite package 'rstatix' in publications use:

  Kassambara A (2023). _rstatix: Pipe-Friendly Framework for Basic
  Statistical Tests_. R package version 0.7.2,
  <https://CRAN.R-project.org/package=rstatix>.

A BibTeX entry for LaTeX users is

  @Manual{,
    title = {rstatix: Pipe-Friendly Framework for Basic Statistical Tests},
    author = {Alboukadel Kassambara},
    year = {2023},
    note = {R package version 0.7.2},
    url = {https://CRAN.R-project.org/package=rstatix},
  }
```

```
citation("coin")
```

```
Use the following references when citing the 'coin' package in
publications:

  Hothorn T, Hornik K, van de Wiel MA, Zeileis A (2006). "A Lego system
  for conditional inference." _The American Statistician_, *60*(3),
  257-263. doi:10.1198/000313006X118430
  <https://doi.org/10.1198/000313006X118430>.

for the implemented theoretical framework and/or

Hothorn T, Hornik K, van de Wiel MA, Zeileis A (2008). "Implementing a
class of permutation tests: The coin package." _Journal of Statistical
Software_, *28*(8), 1-23. doi:10.18637/jss.v028.i08
<https://doi.org/10.18637/jss.v028.i08>.

for the implementation approach.

To see these entries in BibTeX format, use 'print(<citation>,
bibtex=TRUE)', 'toBibtex(.)', or set
'options(citation.bibtex.max=999)'.
```

```
citation("tidyr")
```

```
To cite package 'tidyr' in publications use:

  Wickham H, Vaughan D, Girlich M (2024). _tidyr: Tidy Messy Data_. R
  package version 1.3.1, <https://CRAN.R-project.org/package=tidyr>.

A BibTeX entry for LaTeX users is

  @Manual{,
    title = {tidyr: Tidy Messy Data},
    author = {Hadley Wickham and Davis Vaughan and Maximilian Girlich},
    year = {2024},
    note = {R package version 1.3.1},
    url = {https://CRAN.R-project.org/package=tidyr},
  }
```

```
citation("epiDisplay")
```

```
To cite package 'epiDisplay' in publications use:

  Chongsuvivatwong V (2022). _epiDisplay: Epidemiological Data Display
  Package_. R package version 3.5.0.2,
  <https://CRAN.R-project.org/package=epiDisplay>.

A BibTeX entry for LaTeX users is

  @Manual{,
    title = {epiDisplay: Epidemiological Data Display Package},
    author = {Virasakdi Chongsuvivatwong},
    year = {2022},
    note = {R package version 3.5.0.2},
    url = {https://CRAN.R-project.org/package=epiDisplay},
  }

ATTENTION: This citation information has been auto-generated from the
package DESCRIPTION file and may need manual editing, see
'help("citation")'.
```

```
citation("mosaic")
```

```
To cite mosaic in publications, please use:

  R. Pruim, D. T. Kaplan and N. J. Horton. The mosaic Package: Helping
  Students to 'Think with Data' Using R (2017). The R Journal,
  9(1):77-102.

A BibTeX entry for LaTeX users is

  @Article{,
    author = {Randall Pruim and Daniel T Kaplan and Nicholas J Horton},
    title = {The mosaic Package: Helping Students to 'Think with Data' Using R},
    journal = {The R Journal},
    volume = {9},
    number = {1},
    pages = {77--102},
    year = {2017},
    url = {https://journal.r-project.org/archive/2017/RJ-2017-024/index.html},
  }
```

```
citation("afex")
```

```
To cite package 'afex' in publications use:

  Singmann H, Bolker B, Westfall J, Aust F, Ben-Shachar M (2024).
  _afex: Analysis of Factorial Experiments_. R package version 1.4-1,
  <https://CRAN.R-project.org/package=afex>.

A BibTeX entry for LaTeX users is

  @Manual{,
    title = {afex: Analysis of Factorial Experiments},
    author = {Henrik Singmann and Ben Bolker and Jake Westfall and Frederik Aust and Mattan S. Ben-Shachar},
    year = {2024},
    note = {R package version 1.4-1},
    url = {https://CRAN.R-project.org/package=afex},
  }
```

```
citation("effectsize")
```

```
To cite effectsize in publications use:

  Ben-Shachar M, Lüdecke D, Makowski D (2020). effectsize: Estimation
  of Effect Size Indices and Standardized Parameters. Journal of Open
  Source Software, 5(56), 2815. doi: 10.21105/joss.02815

A BibTeX entry for LaTeX users is

  @Article{,
    title = {{e}ffectsize: Estimation of Effect Size Indices and Standardized Parameters},
    author = {Mattan S. Ben-Shachar and Daniel Lüdecke and Dominique Makowski},
    year = {2020},
    journal = {Journal of Open Source Software},
    volume = {5},
    number = {56},
    pages = {2815},
    publisher = {The Open Journal},
    doi = {10.21105/joss.02815},
    url = {https://doi.org/10.21105/joss.02815},
  }
```

```
citation("emmeans")
```

```
To cite package 'emmeans' in publications use:

  Lenth R (2024). _emmeans: Estimated Marginal Means, aka Least-Squares
  Means_. R package version 1.10.3,
  <https://CRAN.R-project.org/package=emmeans>.

A BibTeX entry for LaTeX users is

  @Manual{,
    title = {emmeans: Estimated Marginal Means, aka Least-Squares Means},
    author = {Russell V. Lenth},
    year = {2024},
    note = {R package version 1.10.3},
    url = {https://CRAN.R-project.org/package=emmeans},
  }
```

```
citation("broom")
```

```
To cite package 'broom' in publications use:

  Robinson D, Hayes A, Couch S (2024). _broom: Convert Statistical
  Objects into Tidy Tibbles_. R package version 1.0.6,
  <https://CRAN.R-project.org/package=broom>.

A BibTeX entry for LaTeX users is

  @Manual{,
    title = {broom: Convert Statistical Objects into Tidy Tibbles},
    author = {David Robinson and Alex Hayes and Simon Couch},
    year = {2024},
    note = {R package version 1.0.6},
    url = {https://CRAN.R-project.org/package=broom},
  }
```

```
citation("DHARMa")
```

```
To cite package 'DHARMa' in publications use:

  Hartig F (2022). _DHARMa: Residual Diagnostics for Hierarchical
  (Multi-Level / Mixed) Regression Models_. R package version 0.4.6,
  <https://CRAN.R-project.org/package=DHARMa>.

A BibTeX entry for LaTeX users is

  @Manual{,
    title = {DHARMa: Residual Diagnostics for Hierarchical (Multi-Level / Mixed)
Regression Models},
    author = {Florian Hartig},
    year = {2022},
    note = {R package version 0.4.6},
    url = {https://CRAN.R-project.org/package=DHARMa},
  }
```

```
citation("ggplot2")
```

```
To cite ggplot2 in publications, please use

  H. Wickham. ggplot2: Elegant Graphics for Data Analysis.
  Springer-Verlag New York, 2016.

A BibTeX entry for LaTeX users is

  @Book{,
    author = {Hadley Wickham},
    title = {ggplot2: Elegant Graphics for Data Analysis},
    publisher = {Springer-Verlag New York},
    year = {2016},
    isbn = {978-3-319-24277-4},
    url = {https://ggplot2.tidyverse.org},
  }
```

```
citation("broom.mixed")
```

```
To cite package 'broom.mixed' in publications use:

  Bolker B, Robinson D (2024). _broom.mixed: Tidying Methods for Mixed
  Models_. R package version 0.2.9.6,
  <https://CRAN.R-project.org/package=broom.mixed>.

A BibTeX entry for LaTeX users is

  @Manual{,
    title = {broom.mixed: Tidying Methods for Mixed Models},
    author = {Ben Bolker and David Robinson},
    year = {2024},
    note = {R package version 0.2.9.6},
    url = {https://CRAN.R-project.org/package=broom.mixed},
  }
```

```
citation("janitor")
```

```
To cite package 'janitor' in publications use:

  Firke S (2023). _janitor: Simple Tools for Examining and Cleaning
  Dirty Data_. R package version 2.2.0,
  <https://CRAN.R-project.org/package=janitor>.

A BibTeX entry for LaTeX users is

  @Manual{,
    title = {janitor: Simple Tools for Examining and Cleaning Dirty Data},
    author = {Sam Firke},
    year = {2023},
    note = {R package version 2.2.0},
    url = {https://CRAN.R-project.org/package=janitor},
  }
```

```
citation("RColorBrewer")
```

```
To cite package 'RColorBrewer' in publications use:

  Neuwirth E (2022). _RColorBrewer: ColorBrewer Palettes_. R package
  version 1.1-3, <https://CRAN.R-project.org/package=RColorBrewer>.

A BibTeX entry for LaTeX users is

  @Manual{,
    title = {RColorBrewer: ColorBrewer Palettes},
    author = {Erich Neuwirth},
    year = {2022},
    note = {R package version 1.1-3},
    url = {https://CRAN.R-project.org/package=RColorBrewer},
  }
```

```
citation("patchwork")
```

```
To cite package 'patchwork' in publications use:

  Pedersen T (2024). _patchwork: The Composer of Plots_. R package
  version 1.3.0, <https://CRAN.R-project.org/package=patchwork>.

A BibTeX entry for LaTeX users is

  @Manual{,
    title = {patchwork: The Composer of Plots},
    author = {Thomas Lin Pedersen},
    year = {2024},
    note = {R package version 1.3.0},
    url = {https://CRAN.R-project.org/package=patchwork},
  }
```

```
citation("naniar")
```

```
To cite naniar in publications use:

  Tierney N, Cook D (2023). "Expanding Tidy Data Principles to
  Facilitate Missing Data Exploration, Visualization and Assessment of
  Imputations." _Journal of Statistical Software_, *105*(7), 1-31.
  doi:10.18637/jss.v105.i07 <https://doi.org/10.18637/jss.v105.i07>.

A BibTeX entry for LaTeX users is

  @Article{,
    title = {Expanding Tidy Data Principles to Facilitate Missing Data Exploration, Visualization and Assessment of Imputations},
    author = {Nicholas Tierney and Dianne Cook},
    journal = {Journal of Statistical Software},
    year = {2023},
    volume = {105},
    number = {7},
    pages = {1--31},
    doi = {10.18637/jss.v105.i07},
  }
```

```
citation("lme4")
```

```
To cite lme4 in publications use:

  Douglas Bates, Martin Maechler, Ben Bolker, Steve Walker (2015).
  Fitting Linear Mixed-Effects Models Using lme4. Journal of
  Statistical Software, 67(1), 1-48. doi:10.18637/jss.v067.i01.

A BibTeX entry for LaTeX users is

  @Article{,
    title = {Fitting Linear Mixed-Effects Models Using {lme4}},
    author = {Douglas Bates and Martin M{\"a}chler and Ben Bolker and Steve Walker},
    journal = {Journal of Statistical Software},
    year = {2015},
    volume = {67},
    number = {1},
    pages = {1--48},
    doi = {10.18637/jss.v067.i01},
  }
```

```
citation("sjPlot")
```

```
To cite package 'sjPlot' in publications use:

  Lüdecke D (2024). _sjPlot: Data Visualization for Statistics in
  Social Science_. R package version 2.8.16,
  <https://CRAN.R-project.org/package=sjPlot>.

A BibTeX entry for LaTeX users is

  @Manual{,
    title = {sjPlot: Data Visualization for Statistics in Social Science},
    author = {Daniel Lüdecke},
    year = {2024},
    note = {R package version 2.8.16},
    url = {https://CRAN.R-project.org/package=sjPlot},
  }
```

```
citation("robustlmm")
```

```
To cite robustlmm in publications use:

  Manuel Koller (2016). robustlmm: An R Package for Robust Estimation
  of Linear Mixed-Effects Models. Journal of Statistical Software,
  75(6), 1-24. doi:10.18637/jss.v075.i06

A BibTeX entry for LaTeX users is

  @Article{,
    title = {{robustlmm}: An {R} Package for Robust Estimation of Linear Mixed-Effects Models},
    author = {Manuel Koller},
    journal = {Journal of Statistical Software},
    year = {2016},
    volume = {75},
    number = {6},
    pages = {1--24},
    doi = {10.18637/jss.v075.i06},
  }
```

```
citation("fitdistrplus")
```

```
To cite fitdistrplus in publications use:

  Marie Laure Delignette-Muller, Christophe Dutang (2015).
  fitdistrplus: An R Package for Fitting Distributions. Journal of
  Statistical Software, 64(4), 1-34. DOI 10.18637/jss.v064.i04.

A BibTeX entry for LaTeX users is

  @Article{,
    title = {{fitdistrplus}: An {R} Package for Fitting Distributions},
    author = {Marie Laure Delignette-Muller and Christophe Dutang},
    journal = {Journal of Statistical Software},
    year = {2015},
    volume = {64},
    number = {4},
    pages = {1--34},
    doi = {10.18637/jss.v064.i04},
  }

Please cite both the package and R when using them for data analysis.
See also 'citation()' for citing R.
```

```
citation("ciTools")
```

```
To cite package 'ciTools' in publications use:

  Haman J, Avery M (2020). _ciTools: Confidence or Prediction
  Intervals, Quantiles, and Probabilities for Statistical Models_. R
  package version 0.6.1, <https://CRAN.R-project.org/package=ciTools>.

A BibTeX entry for LaTeX users is

  @Manual{,
    title = {ciTools: Confidence or Prediction Intervals, Quantiles, and Probabilities
for Statistical Models},
    author = {John Haman and Matthew Avery},
    year = {2020},
    note = {R package version 0.6.1},
    url = {https://CRAN.R-project.org/package=ciTools},
  }
```

```
citation("gamlss")
```

```
To cite GAMLSS in publications use:

  Rigby R.A. and Stasinopoulos D.M. (2005). Generalized additive models
  for location, scale and shape,(with discussion), Appl. Statist., 54,
  part 3, pp 507-554.

A BibTeX entry for LaTeX users is

  @Article{,
    title = {Generalized additive models for location, scale and shape,(with discussion)},
    journal = {Applied Statistics},
    volume = {54},
    part = {3},
    pages = {507-554},
    year = {2005},
    author = {{R. A. Rigby} and {D. M. Stasinopoulos}},
  }

We have invested a lot of time and effort in creating GAMLSS, please
cite it when using it for data analysis.
```

```
citation("glmmTMB")
```

```
To cite glmmTMB in publications use:

  Mollie E. Brooks, Kasper Kristensen, Koen J. van Benthem, Arni
  Magnusson, Casper W. Berg, Anders Nielsen, Hans J. Skaug, Martin
  Maechler and Benjamin M. Bolker (2017). glmmTMB Balances Speed and
  Flexibility Among Packages for Zero-inflated Generalized Linear Mixed
  Modeling. The R Journal, 9(2), 378-400. doi: 10.32614/RJ-2017-066.

A BibTeX entry for LaTeX users is

  @Article{,
    author = {Mollie E. Brooks and Kasper Kristensen and Koen J. {van Benthem} and Arni Magnusson and Casper W. Berg and Anders Nielsen and Hans J. Skaug and Martin Maechler and
Benjamin M. Bolker},
    title = {{glmmTMB} Balances Speed and Flexibility Among Packages for Zero-inflated Generalized Linear Mixed Modeling},
    year = {2017},
    journal = {The R Journal},
    doi = {10.32614/RJ-2017-066},
    pages = {378--400},
    volume = {9},
    number = {2},
  }
```

```
citation("parameters")
```

```
To cite package 'parameters' in publications use:

  Lüdecke D, Ben-Shachar M, Patil I, Makowski D (2020). "Extracting,
  Computing and Exploring the Parameters of Statistical Models using
  R." _Journal of Open Source Software_, *5*(53), 2445.
  doi:10.21105/joss.02445 <https://doi.org/10.21105/joss.02445>.

A BibTeX entry for LaTeX users is

  @Article{,
    title = {Extracting, Computing and Exploring the Parameters of Statistical Models using {R}.},
    volume = {5},
    doi = {10.21105/joss.02445},
    number = {53},
    journal = {Journal of Open Source Software},
    author = {Daniel Lüdecke and Mattan S. Ben-Shachar and Indrajeet Patil and Dominique Makowski},
    year = {2020},
    pages = {2445},
  }
```

```
citation("tidyr")
```

```
To cite package 'tidyr' in publications use:

  Wickham H, Vaughan D, Girlich M (2024). _tidyr: Tidy Messy Data_. R
  package version 1.3.1, <https://CRAN.R-project.org/package=tidyr>.

A BibTeX entry for LaTeX users is

  @Manual{,
    title = {tidyr: Tidy Messy Data},
    author = {Hadley Wickham and Davis Vaughan and Maximilian Girlich},
    year = {2024},
    note = {R package version 1.3.1},
    url = {https://CRAN.R-project.org/package=tidyr},
  }
```

```
citation("flextable")
```

```
To cite package 'flextable' in publications use:

  Gohel D, Skintzos P (2024). _flextable: Functions for Tabular
  Reporting_. R package version 0.9.7,
  <https://CRAN.R-project.org/package=flextable>.

A BibTeX entry for LaTeX users is

  @Manual{,
    title = {flextable: Functions for Tabular Reporting},
    author = {David Gohel and Panagiotis Skintzos},
    year = {2024},
    note = {R package version 0.9.7},
    url = {https://CRAN.R-project.org/package=flextable},
  }
```

```
citation("ggalluvial")
```

```
To cite package 'ggalluvial' in publications, use:

  Jason Cory Brunson and Quentin D. Read (2023). ggalluvial: Alluvial
  Plots in 'ggplot2'. R package version 0.12.5.
  http://corybrunson.github.io/ggalluvial/

To cite layered alluvial plots as implemented in 'ggalluvial':

  Brunson JC (2020). "ggalluvial: Layered Grammar for Alluvial Plots."
  _Journal of Open Source Software_, *5*(49), 2017.
  doi:10.21105/joss.02017 <https://doi.org/10.21105/joss.02017>.

To see these entries in BibTeX format, use 'print(<citation>,
bibtex=TRUE)', 'toBibtex(.)', or set
'options(citation.bibtex.max=999)'.
```

```
citation("dplyr")
```

```
To cite package 'dplyr' in publications use:

  Wickham H, François R, Henry L, Müller K, Vaughan D (2023). _dplyr: A
  Grammar of Data Manipulation_. R package version 1.1.4,
  <https://CRAN.R-project.org/package=dplyr>.

A BibTeX entry for LaTeX users is

  @Manual{,
    title = {dplyr: A Grammar of Data Manipulation},
    author = {Hadley Wickham and Romain François and Lionel Henry and Kirill Müller and Davis Vaughan},
    year = {2023},
    note = {R package version 1.1.4},
    url = {https://CRAN.R-project.org/package=dplyr},
  }
```

```
citation("tibble")
```

```
To cite package 'tibble' in publications use:

  Müller K, Wickham H (2023). _tibble: Simple Data Frames_. R package
  version 3.2.1, <https://CRAN.R-project.org/package=tibble>.

A BibTeX entry for LaTeX users is

  @Manual{,
    title = {tibble: Simple Data Frames},
    author = {Kirill Müller and Hadley Wickham},
    year = {2023},
    note = {R package version 3.2.1},
    url = {https://CRAN.R-project.org/package=tibble},
  }
```

Daten aufbereiten

Daten einlesen

```
cat <- read_excel("~/Desktop/DISS/01 DISS/00 STATISTICS/DATA/Katzen SST/KatzenSST.xlsx",
                        na = "NA")

#cat <- read_excel("KatzenSST.xlsx", na = "NA")
summary(cat)
```

```
    Versuch     Name_Tester       Testjahr     Name_Katze
 Min.   :  1   Min.   :1.000   Min.   :2020   Length:109
 1st Qu.: 26   1st Qu.:3.000   1st Qu.:2022   Class :character
 Median : 51   Median :4.000   Median :2022   Mode  :character
 Mean   : 51   Mean   :4.089   Mean   :2022
 3rd Qu.: 76   3rd Qu.:5.000   3rd Qu.:2023
 Max.   :101   Max.   :6.000   Max.   :2024
 NA's   :8     NA's   :8       NA's   :8
 Geschlecht_Katze     Rasse           Umfeld      Aufenthaltsgrund
 Min.   :1.000    Min.   :1.000   Min.   :1.000   Min.   :1.000
 1st Qu.:1.000    1st Qu.:1.000   1st Qu.:1.000   1st Qu.:1.000
 Median :1.000    Median :3.000   Median :1.000   Median :1.000
 Mean   :1.396    Mean   :3.612   Mean   :1.337   Mean   :1.244
 3rd Qu.:2.000    3rd Qu.:6.000   3rd Qu.:2.000   3rd Qu.:1.000
 Max.   :2.000    Max.   :7.000   Max.   :2.000   Max.   :4.000
 NA's   :8        NA's   :11      NA's   :8       NA's   :27
 Aufenthaltsdauer    Freigang     Kastrationsstatus Mehrkatzenhaushalt
 Min.   :  0.00   Min.   :1.000   Min.   :1.000     Min.   :1.000
 1st Qu.: 12.00   1st Qu.:1.000   1st Qu.:2.000     1st Qu.:1.000
 Median : 24.00   Median :1.000   Median :2.000     Median :1.000
 Mean   : 48.84   Mean   :1.476   Mean   :1.949     Mean   :1.208
 3rd Qu.: 81.00   3rd Qu.:2.000   3rd Qu.:2.000     3rd Qu.:1.000
 Max.   :228.00   Max.   :2.000   Max.   :2.000     Max.   :2.000
 NA's   :26       NA's   :27      NA's   :31        NA's   :8
    Hunde             Alter_Katze      Geschlecht Halter    Episode
 Length:109         Min.   :  2.083   Min.   :1.0       Min.   :1
 Class :character   1st Qu.: 16.000   1st Qu.:1.0       1st Qu.:1
 Mode  :character   Median : 36.000   Median :1.0       Median :1
                    Mean   : 54.560   Mean   :1.1       Mean   :1
                    3rd Qu.: 84.000   3rd Qu.:1.0       3rd Qu.:1
                    Max.   :270.000   Max.   :2.0       Max.   :1
                    NA's   :5         NA's   :49        NA's   :8
  Videodauer_1   Personentyp_1    Exp_n_1         Exp_t_1
 Min.   : 63.0   Min.   :1     Min.   :0.000   Min.   :  0.00
 1st Qu.:180.0   1st Qu.:1     1st Qu.:2.000   1st Qu.: 25.00
 Median :183.0   Median :1     Median :3.000   Median : 56.00
 Mean   :184.5   Mean   :1     Mean   :3.249   Mean   : 64.59
 3rd Qu.:192.5   3rd Qu.:1     3rd Qu.:5.000   3rd Qu.:102.00
 Max.   :484.0   Max.   :1     Max.   :9.000   Max.   :196.00
 NA's   :10      NA's   :11    NA's   :10      NA's   :10
    Ply_n_1           Ply_t_1          Passiv_n_1      Passiv_t_1
 Min.   :0.0000   Min.   :  0.00   Min.   :0.000   Min.   :  0.00
 1st Qu.:0.0000   1st Qu.:  0.00   1st Qu.:0.250   1st Qu.:  3.75
 Median :0.0000   Median :  0.00   Median :1.667   Median : 27.00
 Mean   :0.6736   Mean   : 16.53   Mean   :2.062   Mean   : 40.56
 3rd Qu.:1.0000   3rd Qu.: 14.00   3rd Qu.:3.000   3rd Qu.: 56.00
 Max.   :5.0000   Max.   :176.00   Max.   :8.000   Max.   :199.00
 NA's   :10       NA's   :10       NA's   :10      NA's   :10
    Tuer_n_1        Tuer_t_1          Con_n_1          Con_t_1
 Min.   : 0.00   Min.   :  0.00   Min.   : 0.000   Min.   :  0.00
 1st Qu.: 0.00   1st Qu.:  0.00   1st Qu.: 1.607   1st Qu.:  7.50
 Median : 0.00   Median :  0.00   Median : 3.600   Median : 33.00
 Mean   : 1.39   Mean   : 15.84   Mean   : 3.720   Mean   : 45.82
 3rd Qu.: 2.00   3rd Qu.: 19.75   3rd Qu.: 5.000   3rd Qu.: 68.50
 Max.   :11.00   Max.   :189.00   Max.   :13.000   Max.   :184.00
 NA's   :27      NA's   :27       NA's   :10       NA's   :10
 Kontakt_pro_1    Kontakt_con_1    Kontakt_sum_1     Verz_Kont_1
 Min.   : 0.000   Min.   : 0.000   Min.   :-11.00   Min.   :  0.00
 1st Qu.: 4.000   1st Qu.: 0.000   1st Qu.:  1.25   1st Qu.:  4.00
 Median : 6.000   Median : 0.000   Median :  5.75   Median : 13.00
 Mean   : 7.801   Mean   : 1.331   Mean   :  6.47   Mean   : 43.73
 3rd Qu.:10.000   3rd Qu.: 2.000   3rd Qu.: 10.00   3rd Qu.: 57.75
 Max.   :32.000   Max.   :15.000   Max.   : 32.00   Max.   :180.00
 NA's   :10       NA's   :10       NA's   :10       NA's   :10
 Kont_t_start_1    Lau_pro_miau_1    Lau_pro_schnu_1   Lau_con_fau_1
 Min.   :  0.000   Min.   : 0.0000   Min.   : 0.0000   Min.   :0.00000
 1st Qu.:  0.125   1st Qu.: 0.0000   1st Qu.: 0.0000   1st Qu.:0.00000
 Median :  3.000   Median : 0.0000   Median : 0.8571   Median :0.00000
 Mean   : 14.737   Mean   : 2.0107   Mean   : 1.5287   Mean   :0.03535
 3rd Qu.: 10.000   3rd Qu.: 0.9286   3rd Qu.: 2.0000   3rd Qu.:0.00000
 Max.   :175.000   Max.   :46.0000   Max.   :12.0000   Max.   :3.00000
 NA's   :10        NA's   :10        NA's   :10        NA's   :10
 Lau_con_knu_1       Lau_sum_1         Episode2  Videodauer_2   Personentyp_2
 Min.   :0.0000   Min.   :-3.000   Min.   :2   Min.   : 74.5   Min.   :2.000
 1st Qu.:0.0000   1st Qu.: 0.000   1st Qu.:2   1st Qu.:180.0   1st Qu.:3.000
 Median :0.0000   Median : 2.000   Median :2   Median :186.0   Median :3.000
 Mean   :0.0202   Mean   : 4.088   Mean   :2   Mean   :189.2   Mean   :2.837
 3rd Qu.:0.0000   3rd Qu.: 6.000   3rd Qu.:2   3rd Qu.:196.0   3rd Qu.:3.000
 Max.   :1.0000   Max.   :46.000   Max.   :2   Max.   :389.0   Max.   :3.000
 NA's   :10       NA's   :10       NA's   :8   NA's   :11      NA's   :11
    Exp_n_2          Exp_t_2          Ply_n_f_2         Ply_t_f_2
 Min.   :0.000   Min.   :  0.00   Min.   :0.0000   Min.   :  0.000
 1st Qu.:1.229   1st Qu.: 12.75   1st Qu.:0.0000   1st Qu.:  0.000
 Median :3.000   Median : 52.50   Median :0.0000   Median :  0.000
 Mean   :2.871   Mean   : 64.82   Mean   :0.6139   Mean   : 14.835
 3rd Qu.:4.000   3rd Qu.:111.00   3rd Qu.:1.0000   3rd Qu.:  9.091
 Max.   :7.000   Max.   :193.00   Max.   :5.0000   Max.   :138.000
 NA's   :11      NA's   :11       NA's   :11       NA's   :11
   Ply_n_b_2          Ply_t_b_2          Passiv_n_2     Passiv_t_2
 Min.   :0.00000   Min.   : 0.0000   Min.   :0.00   Min.   :  0.00
 1st Qu.:0.00000   1st Qu.: 0.0000   1st Qu.:1.00   1st Qu.:  4.75
 Median :0.00000   Median : 0.0000   Median :2.00   Median : 33.50
 Mean   :0.09091   Mean   : 0.5151   Mean   :2.13   Mean   : 49.12
 3rd Qu.:0.00000   3rd Qu.: 0.0000   3rd Qu.:3.00   3rd Qu.: 71.50
 Max.   :2.00000   Max.   :13.0000   Max.   :8.00   Max.   :213.33
 NA's   :76        NA's   :76        NA's   :11     NA's   :11
    Tuer_n_2          Tuer_t_2          Con_n_f_2        Con_t_f_2
 Min.   :0.0000   Min.   :  0.00   Min.   :0.000   Min.   :  0.00
 1st Qu.:0.0000   1st Qu.:  0.00   1st Qu.:1.000   1st Qu.:  6.10
 Median :0.0000   Median :  0.00   Median :3.000   Median : 24.50
 Mean   :0.9756   Mean   : 13.96   Mean   :2.991   Mean   : 38.87
 3rd Qu.:2.0000   3rd Qu.: 15.75   3rd Qu.:4.000   3rd Qu.: 65.50
 Max.   :7.0000   Max.   :196.00   Max.   :9.000   Max.   :155.00
 NA's   :27       NA's   :27       NA's   :11      NA's   :11
 Kontakt_pro_f_2  Kontakt_con_f_2  Kontakt_sum_f_2   Verz_Kont_f_2
 Min.   : 0.000   Min.   : 0.000   Min.   :-14.000   Min.   :  0.00
 1st Qu.: 3.050   1st Qu.: 0.000   1st Qu.:  2.000   1st Qu.:  8.25
 Median : 5.750   Median : 1.000   Median :  3.611   Median : 17.50
 Mean   : 7.017   Mean   : 1.318   Mean   :  5.699   Mean   : 42.51
 3rd Qu.: 8.000   3rd Qu.: 2.000   3rd Qu.:  6.000   3rd Qu.: 59.48
 Max.   :38.000   Max.   :14.000   Max.   : 38.000   Max.   :180.00
 NA's   :11       NA's   :11       NA's   :11        NA's   :11
 Kont_t_start_f_2    Con_n_b_2         Con_t_b_2       Kontakt_pro_b_2
 Min.   : 0.0000   Min.   :0.000   Min.   :  0.00   Min.   : 0.000
 1st Qu.: 0.3125   1st Qu.:0.000   1st Qu.:  0.00   1st Qu.: 0.000
 Median : 5.0000   Median :1.000   Median :  6.00   Median : 2.000
 Mean   :10.6645   Mean   :1.865   Mean   : 16.14   Mean   : 3.568
 3rd Qu.:13.0000   3rd Qu.:3.000   3rd Qu.: 15.00   3rd Qu.: 6.000
 Max.   :82.0000   Max.   :8.000   Max.   :124.00   Max.   :14.000
 NA's   :11        NA's   :72      NA's   :72       NA's   :72
 Kontakt_con_b_2    Kontakt_sum_b_2  Verz_Kont_b_2 Kont_t_start_b_2
 Min.   :0.00000   Min.   :-1.000   Min.   :  0   Min.   : 0.000
 1st Qu.:0.00000   1st Qu.: 0.000   1st Qu.: 29   1st Qu.: 0.000
 Median :0.00000   Median : 2.000   Median :111   Median : 2.000
 Mean   :0.05405   Mean   : 3.514   Mean   :107   Mean   : 4.351
 3rd Qu.:0.00000   3rd Qu.: 6.000   3rd Qu.:180   3rd Qu.: 6.000
 Max.   :1.00000   Max.   :14.000   Max.   :180   Max.   :29.000
 NA's   :72        NA's   :72       NA's   :72    NA's   :72
 Lau_pro_miau_2   Lau_pro_schnu_2  Lau_con_fau_2     Lau_con_knu_2
 Min.   : 0.000   Min.   : 0.000   Min.   :0.00000   Min.   :0.00000
 1st Qu.: 0.000   1st Qu.: 0.000   1st Qu.:0.00000   1st Qu.:0.00000
 Median : 0.000   Median : 0.000   Median :0.00000   Median :0.00000
 Mean   : 1.861   Mean   : 1.342   Mean   :0.02296   Mean   :0.02041
 3rd Qu.: 1.056   3rd Qu.: 2.000   3rd Qu.:0.00000   3rd Qu.:0.00000
 Max.   :36.000   Max.   :12.000   Max.   :1.00000   Max.   :1.00000
 NA's   :11       NA's   :11       NA's   :11        NA's   :11
   Lau_sum_2         Episode3   Videodauer_3   Personentyp_3    Exp_n_3
 Min.   :-1.000   Min.   :3    Min.   :155.0   Min.   :2     Min.   :0.000
 1st Qu.: 0.000   1st Qu.:3    1st Qu.:180.0   1st Qu.:2     1st Qu.:2.000
 Median : 2.000   Median :3    Median :183.5   Median :2     Median :3.000
 Mean   : 4.286   Mean   :3    Mean   :186.0   Mean   :2     Mean   :2.976
 3rd Qu.: 5.500   3rd Qu.:3    3rd Qu.:190.0   3rd Qu.:2     3rd Qu.:4.000
 Max.   :36.000   Max.   :3    Max.   :240.0   Max.   :2     Max.   :9.000
 NA's   :11       NA's   :27   NA's   :27      NA's   :27    NA's   :27
    Exp_t_3          Ply_n_3          Ply_t_3          Passiv_n_3
 Min.   :  0.00   Min.   :0.000   Min.   :  0.00   Min.   :0.000
 1st Qu.: 22.00   1st Qu.:0.000   1st Qu.:  0.00   1st Qu.:1.000
 Median : 53.00   Median :0.000   Median :  0.00   Median :2.000
 Mean   : 63.93   Mean   :1.012   Mean   : 23.80   Mean   :2.439
 3rd Qu.:102.75   3rd Qu.:2.000   3rd Qu.: 33.25   3rd Qu.:4.000
 Max.   :180.00   Max.   :8.000   Max.   :159.00   Max.   :8.000
 NA's   :27       NA's   :27      NA's   :27       NA's   :27
   Passiv_t_3        Tuer_n_3         Tuer_t_3          Con_n_3
 Min.   :  0.00   Min.   : 0.000   Min.   :  0.00   Min.   : 0.000
 1st Qu.:  3.00   1st Qu.: 0.000   1st Qu.:  0.00   1st Qu.: 1.000
 Median : 37.00   Median : 1.000   Median : 11.00   Median : 2.000
 Mean   : 49.02   Mean   : 1.695   Mean   : 24.99   Mean   : 2.561
 3rd Qu.: 78.50   3rd Qu.: 3.000   3rd Qu.: 37.25   3rd Qu.: 3.000
 Max.   :199.00   Max.   :10.000   Max.   :181.00   Max.   :10.000
 NA's   :27       NA's   :27       NA's   :27       NA's   :27
    Con_t_3        Kontakt_pro_3    Kontakt_con_3  Kontakt_sum_3
 Min.   :  0.00   Min.   : 0.000   Min.   :0.00   Min.   :-7.00
 1st Qu.:  3.00   1st Qu.: 2.000   1st Qu.:0.00   1st Qu.: 0.00
 Median : 17.00   Median : 4.000   Median :0.50   Median : 2.00
 Mean   : 32.70   Mean   : 5.439   Mean   :1.22   Mean   : 4.22
 3rd Qu.: 50.75   3rd Qu.: 8.000   3rd Qu.:2.00   3rd Qu.: 6.75
 Max.   :180.00   Max.   :28.000   Max.   :9.00   Max.   :28.00
 NA's   :27       NA's   :27       NA's   :27     NA's   :27
  Verz_Kont_3      Kont_t_start_3   Lau_pro_miau_3   Lau_pro_schnu_3
 Min.   :  0.00   Min.   :  0.00   Min.   : 0.000   Min.   : 0
 1st Qu.: 13.00   1st Qu.:  1.00   1st Qu.: 0.000   1st Qu.: 0
 Median : 25.00   Median :  3.00   Median : 0.000   Median : 0
 Mean   : 57.27   Mean   : 19.16   Mean   : 2.805   Mean   : 1
 3rd Qu.: 91.00   3rd Qu.: 15.00   3rd Qu.: 0.000   3rd Qu.: 2
 Max.   :180.00   Max.   :180.00   Max.   :68.000   Max.   :10
 NA's   :72       NA's   :72       NA's   :27       NA's   :27
 Lau_con_fau_3 Lau_con_knu_3       Lau_sum_3          Episode4   Videodauer_4
 Min.   :0     Min.   :0.00000   Min.   :-1.000   Min.   :4    Min.   :122.0
 1st Qu.:0     1st Qu.:0.00000   1st Qu.: 0.000   1st Qu.:4    1st Qu.:180.0
 Median :0     Median :0.00000   Median : 0.000   Median :4    Median :182.5
 Mean   :0     Mean   :0.04878   Mean   : 3.756   Mean   :4    Mean   :184.3
 3rd Qu.:0     3rd Qu.:0.00000   3rd Qu.: 4.000   3rd Qu.:4    3rd Qu.:191.0
 Max.   :0     Max.   :3.00000   Max.   :68.000   Max.   :4    Max.   :219.0
 NA's   :27    NA's   :27        NA's   :27       NA's   :27   NA's   :27
 Personentyp_4    Exp_n_4         Exp_t_4          Ply_n_f_4
 Min.   :3      Min.   :0.000   Min.   :  0.00   Min.   :0.0000
 1st Qu.:3      1st Qu.:2.000   1st Qu.: 16.25   1st Qu.:0.0000
 Median :3      Median :3.000   Median : 60.50   Median :0.0000
 Mean   :3      Mean   :3.159   Mean   : 61.48   Mean   :0.4324
 3rd Qu.:3      3rd Qu.:4.000   3rd Qu.: 87.25   3rd Qu.:0.0000
 Max.   :3      Max.   :9.000   Max.   :180.00   Max.   :6.0000
```

```
Max.   :3      Max.   :9.000   Max.   :180.00   Max.   :6.0000
NA's   :27     NA's   :27      NA's   :27       NA's   :72
  Ply_t_f_42       Ply_n_b_4        Ply_t_b_4        Passiv_n_4
 Min.   :  0.000   Min.   : 0.000   Min.   :  0.00   Min.   :0.000
 1st Qu.:  0.000   1st Qu.: 0.000   1st Qu.:  0.00   1st Qu.:1.000
 Median :  0.000   Median : 0.000   Median :  0.00   Median :2.000
 Mean   :  7.568   Mean   : 1.205   Mean   : 32.92   Mean   :2.183
 3rd Qu.:  0.000   3rd Qu.: 2.000   3rd Qu.: 69.00   3rd Qu.:3.000
 Max.   :157.000   Max.   :10.000   Max.   :165.00   Max.   :8.000
 NA's   :72        NA's   :31       NA's   :31       NA's   :27
  Passiv_t_4       Tuer_n_4         Tuer_t_4         Con_n_f_4
 Min.   :  0.00   Min.   :0.0000   Min.   :  0.00   Min.   :0.000
 1st Qu.:  6.50   1st Qu.:0.0000   1st Qu.:  0.00   1st Qu.:0.000
 Median : 37.50   Median :0.0000   Median :  0.00   Median :2.000
 Mean   : 47.23   Mean   :0.6829   Mean   : 11.51   Mean   :1.973
 3rd Qu.: 68.00   3rd Qu.:1.0000   3rd Qu.:  4.75   3rd Qu.:3.000
 Max.   :187.00   Max.   :7.0000   Max.   :135.00   Max.   :7.000
 NA's   :27       NA's   :27       NA's   :27       NA's   :72
  Con_t_f_4      Kontakt_pro_f_4  Kontakt_con_f_4  Kontakt_sum_f_4
 Min.   : 0.00   Min.   : 0.000   Min.   :0.0000   Min.   :-2.000
 1st Qu.: 0.00   1st Qu.: 0.000   1st Qu.:0.0000   1st Qu.: 0.000
 Median : 8.00   Median : 2.000   Median :0.0000   Median : 2.000
 Mean   :13.59   Mean   : 4.486   Mean   :0.4324   Mean   : 4.054
 3rd Qu.:20.00   3rd Qu.: 6.000   3rd Qu.:0.0000   3rd Qu.: 6.000
 Max.   :82.00   Max.   :26.000   Max.   :5.0000   Max.   :26.000
 NA's   :72      NA's   :72       NA's   :72       NA's   :72
 Verz_Kont_f_4   Kont_t_start_f_4  Con_n_b_4        Con_t_b_4
 Min.   :  0.0   Min.   : 0.000   Min.   : 0.000   Min.   :  0.00
 1st Qu.: 21.0   1st Qu.: 0.000   1st Qu.: 1.000   1st Qu.:  1.25
 Median :114.0   Median : 2.000   Median : 2.000   Median : 13.50
 Mean   :103.1   Mean   : 3.486   Mean   : 2.561   Mean   : 29.70
 3rd Qu.:180.0   3rd Qu.: 5.000   3rd Qu.: 3.750   3rd Qu.: 44.75
 Max.   :180.0   Max.   :21.000   Max.   :13.000   Max.   :130.00
 NA's   :72      NA's   :72       NA's   :27       NA's   :27
 Kontakt_pro_b_4  Kontakt_con_b_4   Kontakt_sum_b_4  Verz_Kont_b_4
 Min.   : 0.000   Min.   : 0.0000   Min.   :-13.00   Min.   :  0.00
 1st Qu.: 0.000   1st Qu.: 0.0000   1st Qu.:  0.00   1st Qu.: 12.00
 Median : 2.000   Median : 0.0000   Median :  2.00   Median : 36.00
 Mean   : 3.704   Mean   : 0.9136   Mean   :  2.79   Mean   : 75.71
 3rd Qu.: 6.000   3rd Qu.: 1.0000   3rd Qu.:  4.00   3rd Qu.:180.00
 Max.   :18.000   Max.   :13.0000   Max.   : 18.00   Max.   :180.00
 NA's   :28       NA's   :28        NA's   :28       NA's   :58
 Kont_t_start_b_4 Lau_pro_miau_4   Lau_pro_schnu_4  Lau_con_fau_4
 Min.   : 0.000   Min.   : 0.000   Min.   :0.0000   Min.   :0.00000
 1st Qu.: 0.000   1st Qu.: 0.000   1st Qu.:0.0000   1st Qu.:0.00000
 Median : 2.000   Median : 0.000   Median :0.0000   Median :0.00000
 Mean   : 8.333   Mean   : 1.341   Mean   :0.7805   Mean   :0.03658
 3rd Qu.: 8.000   3rd Qu.: 0.000   3rd Qu.:2.0000   3rd Qu.:0.00000
 Max.   :99.000   Max.   :34.000   Max.   :8.0000   Max.   :1.00000
 NA's   :58       NA's   :27       NA's   :27       NA's   :27
 Lau_con_knur_4      Lau_sum_4          Episode5   Videodauer_5   Personentyp_5
 Min.   :0.00000   Min.   :-1.000   Min.   :5    Min.   : 66.0   Min.   :1
 1st Qu.:0.00000   1st Qu.: 0.000   1st Qu.:5    1st Qu.:180.0   1st Qu.:1
 Median :0.00000   Median : 0.000   Median :5    Median :181.5   Median :1
 Mean   :0.03658   Mean   : 2.049   Mean   :5    Mean   :180.5   Mean   :1
 3rd Qu.:0.00000   3rd Qu.: 2.000   3rd Qu.:5    3rd Qu.:188.0   3rd Qu.:1
 Max.   :3.00000   Max.   :34.000   Max.   :5    Max.   :250.0   Max.   :1
 NA's   :27        NA's   :27       NA's   :27   NA's   :27      NA's   :27
    Exp_n_5          Exp_t_5          Ply_n_5           Ply_t_5
 Min.   : 0.000   Min.   :  0.00   Min.   :0.0000   Min.   :  0.00
 1st Qu.: 1.000   1st Qu.: 21.25   1st Qu.:0.0000   1st Qu.:  0.00
 Median : 3.000   Median : 59.50   Median :0.0000   Median :  0.00
 Mean   : 3.024   Mean   : 66.21   Mean   :0.8659   Mean   : 23.68
 3rd Qu.: 4.000   3rd Qu.:103.50   3rd Qu.:2.0000   3rd Qu.: 20.00
 Max.   :10.000   Max.   :187.00   Max.   :5.0000   Max.   :169.00
 NA's   :27       NA's   :27       NA's   :27       NA's   :27
  Passiv_n_5      Passiv_t_5        Tuer_n_5        Tuer_t_5
 Min.   :0.000   Min.   :  0.00   Min.   :0.000   Min.   :  0.00
 1st Qu.:1.000   1st Qu.:  4.00   1st Qu.:0.000   1st Qu.:  0.00
 Median :2.000   Median : 26.50   Median :1.000   Median :  2.00
 Mean   :2.122   Mean   : 47.41   Mean   :1.122   Mean   : 16.27
 3rd Qu.:3.000   3rd Qu.: 83.75   3rd Qu.:2.000   3rd Qu.: 17.00
 Max.   :8.000   Max.   :184.00   Max.   :7.000   Max.   :125.00
 NA's   :27      NA's   :27       NA's   :27      NA's   :27
    Con_n_5          Con_t_5        Kontakt_pro_5 Kontakt_con_5
 Min.   : 0.000   Min.   :  0.00   Min.   : 0    Min.   :0.000
 1st Qu.: 1.000   1st Qu.:  2.25   1st Qu.: 0    1st Qu.:0.000
 Median : 2.000   Median : 19.00   Median : 4    Median :0.000
 Mean   : 2.878   Mean   : 33.59   Mean   : 5    Mean   :0.878
 3rd Qu.: 4.000   3rd Qu.: 54.00   3rd Qu.: 6    3rd Qu.:1.000
 Max.   :11.000   Max.   :180.00   Max.   :28    Max.   :8.000
 NA's   :27       NA's   :27       NA's   :27    NA's   :27
 Kontakt_sum_5     Verz_Kont_5      Kont_t_start_5   Lau_pro_miau_5
 Min.   :-4.000   Min.   :  0.00   Min.   :  0.00   Min.   : 0.000
 1st Qu.: 0.000   1st Qu.:  8.00   1st Qu.:  0.00   1st Qu.: 0.000
 Median : 2.000   Median : 22.00   Median :  1.00   Median : 0.000
 Mean   : 4.122   Mean   : 57.82   Mean   : 11.02   Mean   : 1.683
 3rd Qu.: 5.000   3rd Qu.:105.50   3rd Qu.:  7.50   3rd Qu.: 0.000
 Max.   :28.000   Max.   :180.00   Max.   :180.00   Max.   :26.000
 NA's   :27       NA's   :58       NA's   :58       NA's   :27
 Lau_pro_schnu_5 Lau_con_fau_5 Lau_con_knur_5      Lau_sum_5
 Min.   :0.000   Min.   :0     Min.   :0.00000   Min.   : 0.000
 1st Qu.:0.000   1st Qu.:0     1st Qu.:0.00000   1st Qu.: 0.000
 Median :0.000   Median :0     Median :0.00000   Median : 2.000
 Mean   :1.317   Mean   :0     Mean   :0.03658   Mean   : 2.963
 3rd Qu.:2.000   3rd Qu.:0     3rd Qu.:0.00000   3rd Qu.: 4.000
 Max.   :8.000   Max.   :0     Max.   :3.00000   Max.   :26.000
 NA's   :27      NA's   :27    NA's   :27        NA's   :27
     Pilot
 Min.   :0.0000
 1st Qu.:1.0000
 Median :1.0000
 Mean   :0.8119
 3rd Qu.:1.0000
 Max.   :1.0000
 NA's   :8
```

```
cat <- janitor::clean_names(cat)

#Katzen ausschließen aus Pilotversuch
pilot <- subset(cat, versuch>=83)
cat <- subset(cat, versuch<83)
```

Faktoren definieren

```
cat$umfeld <- factor(cat$umfeld,levels = c(1,2), labels = c("Private Home","Animal Shelter"))

cat$geschlecht_katze <- factor(cat$geschlecht_katze,levels = c(1,2), labels = c("Female","Male"))

cat$rasse <- factor(cat$rasse,levels = c(1,2,3,4,5,6,7), labels = c("EKH","Siam", "BKH","Maincoon", "Perser", "Mix", "GHK"))

cat$freigang <- factor(cat$freigang,levels = c(1,2), labels = c("Outdoor", "Indoor"))

cat$kastrationsstatus <- factor(cat$kastrationsstatus, levels = c(1, 2), labels = c("intact", "Neutered"))

cat$mehrkatzenhaushalt <- factor(cat$mehrkatzenhaushalt, levels = c(1, 2), labels = c("Single-Cat", "Multi-Cat"))

cat$hunde <- factor(cat$hunde, levels = c(1, 2), labels = c("With Dog", "No Dog"))

cat$geschlecht_halter <- factor(cat$geschlecht_halter, levels = c(1, 2), labels = c("Female", "Male"))

cat$alter_katze <- factor(cat$alter_katze, levels = c(1,2,3,4,5,6,7,8), labels = c("<3 Months", "4-6 Months","7-9 Months", "10-11 Months", "1-3 Years", "4-6 Years","7-9 Years", ">10 Year


levels(cat$freigang) <- c("Outdoor", "Indoor")
```

Subset 1 erstellen

7 Verhaltensweisen:

Exploration (exp_t_x), Spielverhalten (ply_t_x), Passives Verhalten (passiv_t_start_x), Verhalten an der Tür (tuer_t_x), physischer Kontakt (con_t_x), Verzögerung der Kontaktaufnahme (verz_kont_x), Dauer Kontaktaufnahme (kont_t_start_x)

- Verzögerung der Kontaktaufnahme (verz_kont_x), Dauer Kontaktaufnahme (kont_t_start_x) werden beide ausgeschlossen - 19 incomplete Cases

wide in long Format umwandeln

```
cat_sub <- cat %>% dplyr::select(versuch, geschlecht_katze:geschlecht_halter,

                                  exp_t_1, ply_t_1, passiv_t_1, tuer_t_1, con_t_1,

                                  exp_t_2, ply_t_f_2, passiv_t_2, tuer_t_2, con_t_f_2,

                                  exp_t_3, ply_t_3, passiv_t_3, tuer_t_3, con_t_3,

                                  exp_t_4, ply_t_b_4, passiv_t_4, tuer_t_4, con_t_b_4,

                                  exp_t_5, ply_t_5, passiv_t_5, tuer_t_5, con_t_5,
                                   )


cat_sub <- cat_sub %>% dplyr::rename(
    exp_1 = exp_t_1,
    ply_1 = ply_t_1,
    passiv_1 = passiv_t_1,
    tuer_1 = tuer_t_1,
    con_1 = con_t_1,

    exp_2 = exp_t_2,
    ply_2 = ply_t_f_2,
    passiv_2 = passiv_t_2,
    tuer_2 = tuer_t_2,
    con_2 = con_t_f_2,

    exp_3 = exp_t_3,
    ply_3 = ply_t_3,
    passiv_3 = passiv_t_3,
    tuer_3 = tuer_t_3,
    con_3 = con_t_3,

    exp_4 = exp_t_4,
    ply_4 = ply_t_b_4,
    passiv_4 = passiv_t_4,
    tuer_4 = tuer_t_4,
    con_4 = con_t_b_4,

    exp_5 = exp_t_5,
    ply_5 = ply_t_5,
    passiv_5 = passiv_t_5,
    tuer_5 = tuer_t_5,
    con_5 = con_t_5,

  )

#kont soll verworfen werden
cat_long <- cat_sub %>% tidyr::pivot_longer(cols= exp_1:con_5,
                                            names_sep = "_",
                                            names_to = c("verhalten","zeit"),
                                            values_to = "sec")

cat_long$verhalten <- factor(cat_long$verhalten)
cat_long$zeit <- factor(cat_long$zeit)

# für Darstellung der Verhalten sonst wird neuer Df erstellt

#result <- cat_long %>%
  # Convert zeit to numeric if it's a factor
  #mutate(zeit = as.numeric(as.character(zeit))) %>%
  # Pivot the data wider so each behavior has its own column
  # pivot_wider(
  #   id_cols = c(versuch, zeit), # Keep identifying columns
  #   names_from = verhalten,
  #   values_from = sec,
  #   values_fill = 0  # Replace NA with 0
  # ) %>%
  # # Calculate "sonst" as 180 minus sum of other behaviors
  # mutate(
  #   sonst = 180 - (exp + ply + passiv + tuer + con)
  # ) %>%
  # # Convert back to long format
  # pivot_longer(
  #   cols = c(exp, ply, passiv, tuer, con, sonst),
  #   names_to = "verhalten",
  #   values_to = "sec"
  # )


cat_l <- cat_long %>%
  pivot_wider(names_from = verhalten, values_from = sec)
```

Subset 2 erstellen

2 Verhaltensweisen:

Ergebnis der Kontaktaufnahme (kontakt_sum_x), Ergebnis der Lautäußerung (lau_sum_x)

ACHTUNG: Ordinaldaten

- hier werden die ursprünglich gemessenen Variablen VOR der Summierung verwendet und die Summen verworfen

Lautäußerung Fauchen (lau_con_fau), Lautäußerung Knurren (lau_con_knu), Lautäußerung Miauen (lau_pro_miau), Lautäußerung Schnuren (lau_pro_schnu)

Initiierndes Kontaktverhalten (kontakt_pro), Vermeidendes Kontaktverhalten (kontakt_con)

wide in long Format umwandeln

```
cat_sub2 <- cat %>% dplyr::select(versuch, geschlecht_katze:geschlecht_halter,
                                  kontakt_pro_1, kontakt_con_1, lau_pro_miau_1, lau_pro_schnu_1,
                                  lau_con_fau_1, lau_con_knu_1,

                                  kontakt_pro_f_2, kontakt_con_f_2, lau_pro_miau_2,
                                  lau_pro_schnu_2, lau_con_fau_2, lau_con_knu_2,

                                  kontakt_pro_3, kontakt_con_3, lau_pro_miau_3,
                                  lau_pro_schnu_3, lau_con_fau_3, lau_con_knu_3,

                                  kontakt_pro_b_4, kontakt_con_b_4, lau_pro_miau_4,
                                  lau_pro_schnu_4, lau_con_fau_4, lau_con_knur_4,

                                  kontakt_pro_5, kontakt_con_5, lau_pro_miau_5,
                                  lau_pro_schnu_5, lau_con_fau_5, lau_con_knur_5)

cat_sub2 <- cat_sub2 %>% dplyr::rename(
    kontaktini_1 = kontakt_pro_1,
    kontaktver_1 = kontakt_con_1,
    miauen_1 = lau_pro_miau_1,
    schnurren_1 = lau_pro_schnu_1,
    fauchen_1 = lau_con_fau_1,
    knurren_1 = lau_con_knu_1,

    kontaktini_2 = kontakt_pro_f_2,
    kontaktver_2 = kontakt_con_f_2,
    miauen_2 = lau_pro_miau_2,
    schnurren_2 = lau_pro_schnu_2,
    fauchen_2 = lau_con_fau_2,
    knurren_2 = lau_con_knu_2,

    kontaktini_3 = kontakt_pro_3,
    kontaktver_3 = kontakt_con_3,
    miauen_3 = lau_pro_miau_3,
    schnurren_3 = lau_pro_schnu_3,
    fauchen_3 = lau_con_fau_3,
    knurren_3 = lau_con_knu_3,

    kontaktini_4 = kontakt_pro_b_4,
    kontaktver_4 = kontakt_con_b_4,
    miauen_4 = lau_pro_miau_4,
    schnurren_4 = lau_pro_schnu_4,
    fauchen_4 = lau_con_fau_4,
    knurren_4 = lau_con_knur_4,

    kontaktini_5 = kontakt_pro_5,
    kontaktver_5 = kontakt_con_5,
    miauen_5 = lau_pro_miau_5,
    schnurren_5 = lau_pro_schnu_5,
    fauchen_5 = lau_con_fau_5,
    knurren_5 = lau_con_knur_5,
  )


cat_long2 <- cat_sub2 %>% tidyr::pivot_longer(cols= kontaktini_1:knurren_5,
                                            names_sep = "_",
                                            names_to = c("verhalten","zeit"),
                                            values_to = "anzahl")

cat_long2$verhalten <- factor(cat_long2$verhalten)
cat_long2$zeit <- factor(cat_long2$zeit)

cat_l2 <- cat_long2 %>%
  pivot_wider(names_from = verhalten, values_from = anzahl)
```

Deskriptive Analyse

Fehlende Werte

```
md.pattern(cat)
```

```
   versuch name_tester testjahr name_katze geschlecht_katze rasse umfeld
14       1           1        1          1                1     1      1
30       1           1        1          1                1     1      1
1        1           1        1          1                1     1      1
22       1           1        1          1                1     1      1
9        1           1        1          1                1     1      1
1        1           1        1          1                1     1      1
1        1           1        1          1                1     1      1
2        1           1        1          1                1     1      1
1        1           1        1          1                1     1      1
1        1           1        1          1                1     1      1
         0           0        0          0                0     0      0
   aufenthaltsgrund freigang mehrkatzenhaushalt episode videodauer_1
14                1        1                  1       1            1
30                1        1                  1       1            1
1                 1        1                  1       1            1
22                1        1                  1       1            1
9                 1        1                  1       1            1
1                 1        1                  1       1            1
1                 1        1                  1       1            1
2                 1        1                  1       1            1
1                 1        1                  1       1            1
1                 1        1                  1       1            1
                  0        0                  0       0            0
   personentyp_1 exp_n_1 exp_t_1 ply_n_1 ply_t_1 passiv_n_1 passiv_t_1 tuer_n_1
14             1       1       1       1       1          1          1        1
30             1       1       1       1       1          1          1        1
1              1       1       1       1       1          1          1        1
22             1       1       1       1       1          1          1        1
9              1       1       1       1       1          1          1        1
1              1       1       1       1       1          1          1        1
1              1       1       1       1       1          1          1        1
2              1       1       1       1       1          1          1        1
1              1       1       1       1       1          1          1        1
1              1       1       1       1       1          1          1        1
               0       0       0       0       0          0          0        0
   tuer_t_1 con_n_1 con_t_1 kontakt_pro_1 kontakt_con_1 kontakt_sum_1
14        1       1       1             1             1             1
30        1       1       1             1             1             1
1         1       1       1             1             1             1
22        1       1       1             1             1             1
9         1       1       1             1             1             1
1         1       1       1             1             1             1
1         1       1       1             1             1             1
2         1       1       1             1             1             1
1         1       1       1             1             1             1
1         1       1       1             1             1             1
          0       0       0             0             0             0
   verz_kont_1 kont_t_start_1 lau_pro_miau_1 lau_pro_schnu_1 lau_con_fau_1
14           1              1              1               1             1
30           1              1              1               1             1
1            1              1              1               1             1
22           1              1              1               1             1
9            1              1              1               1             1
1            1              1              1               1             1
1            1              1              1               1             1
2            1              1              1               1             1
1            1              1              1               1             1
1            1              1              1               1             1
             0              0              0               0             0
   lau_con_knu_1 lau_sum_1 episode2 videodauer_2 personentyp_2 exp_n_2 exp_t_2
14             1         1        1            1             1       1       1
30             1         1        1            1             1       1       1
1              1         1        1            1             1       1       1
22             1         1        1            1             1       1       1
9              1         1        1            1             1       1       1
1              1         1        1            1             1       1       1
1              1         1        1            1             1       1       1
2              1         1        1            1             1       1       1
1              1         1        1            1             1       1       1
1              1         1        1            1             1       1       1
               0         0        0            0             0       0       0
   ply_n_f_2 ply_t_f_2 passiv_n_2 passiv_t_2 tuer_n_2 tuer_t_2 con_n_f_2
14         1         1          1          1        1        1         1
30         1         1          1          1        1        1         1
1          1         1          1          1        1        1         1
22         1         1          1          1        1        1         1
9          1         1          1          1        1        1         1
1          1         1          1          1        1        1         1
1          1         1          1          1        1        1         1
2          1         1          1          1        1        1         1
1          1         1          1          1        1        1         1
1          1         1          1          1        1        1         1
           0         0          0          0        0        0         0
   con_t_f_2 kontakt_pro_f_2 kontakt_con_f_2 kontakt_sum_f_2 verz_kont_f_2
14         1               1               1               1             1
30         1               1               1               1             1
1          1               1               1               1             1
22         1               1               1               1             1
9          1               1               1               1             1
1          1               1               1               1             1
1          1               1               1               1             1
2          1               1               1               1             1
1          1               1               1               1             1
1          1               1               1               1             1
           0               0               0               0             0
   kont_t_start_f_2 lau_pro_miau_2 lau_pro_schnu_2 lau_con_fau_2 lau_con_knu_2
14                1              1               1             1             1
30                1              1               1             1             1
1                 1              1               1             1             1
22                1              1               1             1             1
9                 1              1               1             1             1
1                 1              1               1             1             1
1                 1              1               1             1             1
2                 1              1               1             1             1
1                 1              1               1             1             1
1                 1              1               1             1             1
                  0              0               0             0             0
   lau_sum_2 episode3 videodauer_3 personentyp_3 exp_n_3 exp_t_3 ply_n_3
14         1        1            1             1       1       1       1
30         1        1            1             1       1       1       1
1          1        1            1             1       1       1       1
22         1        1            1             1       1       1       1
9          1        1            1             1       1       1       1
1          1        1            1             1       1       1       1
1          1        1            1             1       1       1       1
2          1        1            1             1       1       1       1
1          1        1            1             1       1       1       1
1          1        1            1             1       1       1       1
           0        0            0             0       0       0       0
   ply_t_3 passiv_n_3 passiv_t_3 tuer_n_3 tuer_t_3 con_n_3 con_t_3
14       1          1          1        1        1       1       1
30       1          1          1        1        1       1       1
1        1          1          1        1        1       1       1
22       1          1          1        1        1       1       1
9        1          1          1        1        1       1       1
1        1          1          1        1        1       1       1
1        1          1          1        1        1       1       1
2        1          1          1        1        1       1       1
1        1          1          1        1        1       1       1
1        1          1          1        1        1       1       1
         0          0          0        0        0       0       0
   kontakt_pro_3 kontakt_con_3 kontakt_sum_3 lau_pro_miau_3 lau_pro_schnu_3
14             1             1             1              1               1
30             1             1             1              1               1
1              1             1             1              1               1
22             1             1             1              1               1
9              1             1             1              1               1
1              1             1             1              1               1
1              1             1             1              1               1
2              1             1             1              1               1
1              1             1             1              1               1
1              1             1             1              1               1
               0             0             0              0               0
   lau_con_fau_3 lau_con_knu_3 lau_sum_3 episode4 videodauer_4 personentyp_4
14             1             1         1        1            1             1
30             1             1         1        1            1             1
1              1             1         1        1            1             1
22             1             1         1        1            1             1
9              1             1         1        1            1             1
1              1             1         1        1            1             1
1              1             1         1        1            1             1
2              1             1         1        1            1             1
1              1             1         1        1            1             1
1              1             1         1        1            1             1
               0             0         0        0            0             0
   exp_n_4 exp_t_4 passiv_n_4 passiv_t_4 tuer_n_4 tuer_t_4 con_n_b_4 con_t_b_4
14       1       1          1          1        1        1         1         1
30       1       1          1          1        1        1         1         1
1        1       1          1          1        1        1         1         1
22       1       1          1          1        1        1         1         1
9        1       1          1          1        1        1         1         1
1        1       1          1          1        1        1         1         1
1        1       1          1          1        1        1         1         1
2        1       1          1          1        1        1         1         1
1        1       1          1          1        1        1         1         1
1        1       1          1          1        1        1         1         1
         0       0          0          0        0        0         0         0
   lau_pro_miau_4 lau_pro_schnu_4 lau_con_fau_4 lau_con_knur_4 lau_sum_4
14              1               1             1              1         1
30              1               1             1              1         1
1               1               1             1              1         1
22              1               1             1              1         1
9               1               1             1              1         1
1               1               1             1              1         1
1               1               1             1              1         1
2               1               1             1              1         1
1               1               1             1              1         1
1               1               1             1              1         1
                0               0             0              0         0
   episode5 videodauer_5 personentyp_5 exp_n_5 exp_t_5 ply_n_5 ply_t_5
14        1            1             1       1       1       1       1
30        1            1             1       1       1       1       1
1         1            1             1       1       1       1       1
22        1            1             1       1       1       1       1
9         1            1             1       1       1       1       1
1         1            1             1       1       1       1       1
1         1            1             1       1       1       1       1
2         1            1             1       1       1       1       1
1         1            1             1       1       1       1       1
1         1            1             1       1       1       1       1
          0            0             0       0       0       0       0
   passiv_n_5 passiv_t_5 tuer_n_5 tuer_t_5 con_n_5 con_t_5 kontakt_pro_5
14          1          1        1        1       1       1             1
30          1          1        1        1       1       1             1
1           1          1        1        1       1       1             1
22          1          1        1        1       1       1             1
9           1          1        1        1       1       1             1
1           1          1        1        1       1       1             1
1           1          1        1        1       1       1             1
2           1          1        1        1       1       1             1
1           1          1        1        1       1       1             1
1           1          1        1        1       1       1             1
            0          0        0        0       0       0             0
   kontakt_con_5 kontakt_sum_5 lau_pro_miau_5 lau_pro_schnu_5 lau_con_fau_5
14             1             1              1               1             1
30             1             1              1               1             1
1              1             1              1               1             1
22             1             1              1               1             1
9              1             1              1               1             1
1              1             1              1               1             1
1              1             1              1               1             1
2              1             1              1               1             1
1              1             1              1               1             1
1              1             1              1               1             1
               0             0              0               0             0
   lau_con_knur_5 lau_sum_5 pilot kontakt_pro_b_4 kontakt_con_b_4
14              1         1     1               1               1
30              1         1     1               1               1
1               1         1     1               1               1
22              1         1     1               1               1
9               1         1     1               1               1
1               1         1     1               1               1
1               1         1     1               1               1
2               1         1     1               1               1
1               1         1     1               1               1
1               1         1     1               0               0
                0         0     0               1               1
   kontakt_sum_b_4 ply_n_b_4 ply_t_b_4 aufenthaltsdauer geschlecht_halter
14               1         1         1                1                 1
30               1         1         1                1                 1
1                1         1         1                1                 1
22               1         1         1                1                 0
9                1         1         1                0                 1
1                1         1         1                0                 1
1                1         0         0                0                 1
2                1         0         0                0                 1
1                1         0         0                0                 1
1                0         1         1                0                 1
                 1         4         4               15                22
   kastrationsstatus verz_kont_b_4 kont_t_start_b_4 verz_kont_5 kont_t_start_5
14                 1             1                1           1              1
30                 1             0                0           0              0
1                  1             0                0           0              0
22                 0             1                1           1              1
9                  1             1                1           1              1
1                  1             1                1           1              1
1                  1             1                1           1              1
2                  1             1                1           1              1
1                  1             1                1           1              1
1                  0             1                1           1              1
                  23            31               31          31             31
   hunde con_n_b_2 con_t_b_2 kontakt_pro_b_2 kontakt_con_b_2 kontakt_sum_b_2
14     0         0         0               0               0               0
30     1         0         0               0               0               0
1      0         0         0               0               0               0
22     0         1         1               1               1               1
9      1         1         1               1               1               1
1      1         1         1               1               1               1
1      1         1         1               1               1               1
2      1         1         1               1               1               1
1      1         1         1               1               1               1
1      1         1         1               1               1               1
      37        45        45              45              45              45
   verz_kont_b_2 kont_t_start_b_2 verz_kont_3 kont_t_start_3 ply_n_f_4
14             0                0           0              0         0
30             0                0           0              0         0
1              0                0           0              0         0
22             1                1           1              1         1
9              1                1           1              1         1
1              1                1           1              1         1
1              1                1           1              1         1
2              1                1           1              1         1
1              1                1           1              1         1
1              1                1           1              1         1
              45               45          45             45        45
   ply_t_f_42 con_n_f_4 con_t_f_4 kontakt_pro_f_4 kontakt_con_f_4
14          0         0         0               0               0
30          0         0         0               0               0
1           0         0         0               0               0
22          1         1         1               1               1
9           1         1         1               1               1
1           1         1         1               1               1
1           1         1         1               1               1
2           1         1         1               1               1
1           1         1         1               1               1
1           1         1         1               1               1
           45        45        45              45              45
   kontakt_sum_f_4 verz_kont_f_4 kont_t_start_f_4 ply_n_b_2 ply_t_b_2
14               0             0                0         0         0
30               0             0                0         0         0
1                0             0                0         0         0
22               1             1                1         1         1
9                1             1                1         1         1
1                1             1                1         0         0
1                1             1                1         1         1
2                1             1                1         0         0
1                1             1                1         0         0
1                1             1                1         1         1
                45            45               45        49        49
   alter_katze
14           0   22
30           0   25
1            0   26
22           0    4
9            0    2
1            0    4
1            0    4
2            1    5
1            0    6
1            0    6
            80 1220
```

```
vis_miss(cat)
```

```
colSums(is.na(cat)) / nrow(cat)
```

```
          versuch        name_tester           testjahr         name_katze
       0.00000000         0.00000000         0.00000000         0.00000000
 geschlecht_katze              rasse             umfeld   aufenthaltsgrund
       0.00000000         0.00000000         0.00000000         0.00000000
 aufenthaltsdauer           freigang  kastrationsstatus mehrkatzenhaushalt
       0.18292683         0.00000000         0.28048780         0.00000000
            hunde        alter_katze  geschlecht_halter            episode
       0.45121951         0.97560976         0.26829268         0.00000000
     videodauer_1      personentyp_1            exp_n_1            exp_t_1
       0.00000000         0.00000000         0.00000000         0.00000000
          ply_n_1            ply_t_1         passiv_n_1         passiv_t_1
       0.00000000         0.00000000         0.00000000         0.00000000
         tuer_n_1           tuer_t_1            con_n_1            con_t_1
       0.00000000         0.00000000         0.00000000         0.00000000
    kontakt_pro_1      kontakt_con_1      kontakt_sum_1        verz_kont_1
       0.00000000         0.00000000         0.00000000         0.00000000
   kont_t_start_1     lau_pro_miau_1    lau_pro_schnu_1      lau_con_fau_1
       0.00000000         0.00000000         0.00000000         0.00000000
    lau_con_knu_1          lau_sum_1           episode2       videodauer_2
       0.00000000         0.00000000         0.00000000         0.00000000
    personentyp_2            exp_n_2            exp_t_2          ply_n_f_2
       0.00000000         0.00000000         0.00000000         0.00000000
        ply_t_f_2          ply_n_b_2          ply_t_b_2         passiv_n_2
       0.00000000         0.59756098         0.59756098         0.00000000
       passiv_t_2           tuer_n_2           tuer_t_2          con_n_f_2
       0.00000000         0.00000000         0.00000000         0.00000000
        con_t_f_2    kontakt_pro_f_2    kontakt_con_f_2    kontakt_sum_f_2
       0.00000000         0.00000000         0.00000000         0.00000000
    verz_kont_f_2   kont_t_start_f_2          con_n_b_2          con_t_b_2
       0.00000000         0.00000000         0.54878049         0.54878049
  kontakt_pro_b_2    kontakt_con_b_2    kontakt_sum_b_2      verz_kont_b_2
       0.54878049         0.54878049         0.54878049         0.54878049
 kont_t_start_b_2     lau_pro_miau_2    lau_pro_schnu_2      lau_con_fau_2
       0.54878049         0.00000000         0.00000000         0.00000000
    lau_con_knu_2          lau_sum_2           episode3       videodauer_3
       0.00000000         0.00000000         0.00000000         0.00000000
    personentyp_3            exp_n_3            exp_t_3            ply_n_3
       0.00000000         0.00000000         0.00000000         0.00000000
          ply_t_3         passiv_n_3         passiv_t_3           tuer_n_3
       0.00000000         0.00000000         0.00000000         0.00000000
         tuer_t_3            con_n_3            con_t_3      kontakt_pro_3
       0.00000000         0.00000000         0.00000000         0.00000000
    kontakt_con_3      kontakt_sum_3        verz_kont_3     kont_t_start_3
       0.00000000         0.00000000         0.54878049         0.54878049
   lau_pro_miau_3    lau_pro_schnu_3      lau_con_fau_3      lau_con_knu_3
       0.00000000         0.00000000         0.00000000         0.00000000
        lau_sum_3           episode4       videodauer_4      personentyp_4
       0.00000000         0.00000000         0.00000000         0.00000000
          exp_n_4            exp_t_4          ply_n_f_4         ply_t_f_42
       0.00000000         0.00000000         0.54878049         0.54878049
        ply_n_b_4          ply_t_b_4         passiv_n_4         passiv_t_4
       0.04878049         0.04878049         0.00000000         0.00000000
         tuer_n_4           tuer_t_4          con_n_f_4          con_t_f_4
       0.00000000         0.00000000         0.54878049         0.54878049
  kontakt_pro_f_4    kontakt_con_f_4    kontakt_sum_f_4      verz_kont_f_4
       0.54878049         0.54878049         0.54878049         0.54878049
 kont_t_start_f_4          con_n_b_4          con_t_b_4    kontakt_pro_b_4
       0.54878049         0.00000000         0.00000000         0.01219512
  kontakt_con_b_4    kontakt_sum_b_4      verz_kont_b_4   kont_t_start_b_4
       0.01219512         0.01219512         0.37804878         0.37804878
   lau_pro_miau_4    lau_pro_schnu_4      lau_con_fau_4     lau_con_knur_4
       0.00000000         0.00000000         0.00000000         0.00000000
        lau_sum_4           episode5       videodauer_5      personentyp_5
       0.00000000         0.00000000         0.00000000         0.00000000
          exp_n_5            exp_t_5            ply_n_5            ply_t_5
       0.00000000         0.00000000         0.00000000         0.00000000
       passiv_n_5         passiv_t_5           tuer_n_5           tuer_t_5
       0.00000000         0.00000000         0.00000000         0.00000000
          con_n_5            con_t_5      kontakt_pro_5      kontakt_con_5
       0.00000000         0.00000000         0.00000000         0.00000000
    kontakt_sum_5        verz_kont_5     kont_t_start_5     lau_pro_miau_5
       0.00000000         0.37804878         0.37804878         0.00000000
  lau_pro_schnu_5      lau_con_fau_5     lau_con_knur_5          lau_sum_5
       0.00000000         0.00000000         0.00000000         0.00000000
            pilot
       0.00000000
```

```
gg_miss_upset(cat)
```

```
naniar::prop_miss_row(cat)
```

```
 [1] 0.02684564 0.04026846 0.02684564 0.01342282 0.01342282 0.03355705
 [7] 0.01342282 0.04026846 0.01342282 0.01342282 0.01342282 0.03355705
[13] 0.01342282 0.01342282 0.01342282 0.14765101 0.14765101 0.14765101
[19] 0.14765101 0.14765101 0.14765101 0.14765101 0.17449664 0.14765101
[25] 0.14765101 0.14765101 0.14765101 0.14765101 0.14765101 0.14765101
[31] 0.16778523 0.16778523 0.16778523 0.16778523 0.16778523 0.16778523
[37] 0.16778523 0.16778523 0.16778523 0.16778523 0.16778523 0.16778523
[43] 0.16778523 0.16778523 0.16778523 0.16778523 0.16778523 0.16778523
[49] 0.16778523 0.16778523 0.16778523 0.16778523 0.16778523 0.16778523
[55] 0.16778523 0.16778523 0.16778523 0.16778523 0.16778523 0.16778523
[61] 0.02684564 0.02684564 0.02684564 0.02684564 0.02684564 0.02684564
[67] 0.02684564 0.02684564 0.02684564 0.02684564 0.02684564 0.02684564
[73] 0.02684564 0.02684564 0.02684564 0.02684564 0.02684564 0.02684564
[79] 0.02684564 0.02684564 0.02684564 0.02684564
```

```
md.pattern(cat_sub)
```

```
   versuch geschlecht_katze rasse umfeld aufenthaltsgrund freigang
30       1                1     1      1                1        1
15       1                1     1      1                1        1
22       1                1     1      1                1        1
10       1                1     1      1                1        1
1        1                1     1      1                1        1
2        1                1     1      1                1        1
2        1                1     1      1                1        1
         0                0     0      0                0        0
   mehrkatzenhaushalt exp_1 ply_1 passiv_1 tuer_1 con_1 exp_2 ply_2 passiv_2
30                  1     1     1        1      1     1     1     1        1
15                  1     1     1        1      1     1     1     1        1
22                  1     1     1        1      1     1     1     1        1
10                  1     1     1        1      1     1     1     1        1
1                   1     1     1        1      1     1     1     1        1
2                   1     1     1        1      1     1     1     1        1
2                   1     1     1        1      1     1     1     1        1
                    0     0     0        0      0     0     0     0        0
   tuer_2 con_2 exp_3 ply_3 passiv_3 tuer_3 con_3 exp_4 passiv_4 tuer_4 con_4
30      1     1     1     1        1      1     1     1        1      1     1
15      1     1     1     1        1      1     1     1        1      1     1
22      1     1     1     1        1      1     1     1        1      1     1
10      1     1     1     1        1      1     1     1        1      1     1
1       1     1     1     1        1      1     1     1        1      1     1
2       1     1     1     1        1      1     1     1        1      1     1
2       1     1     1     1        1      1     1     1        1      1     1
        0     0     0     0        0      0     0     0        0      0     0
   exp_5 ply_5 passiv_5 tuer_5 con_5 ply_4 aufenthaltsdauer geschlecht_halter
30     1     1        1      1     1     1                1                 1
15     1     1        1      1     1     1                1                 1
22     1     1        1      1     1     1                1                 0
10     1     1        1      1     1     1                0                 1
1      1     1        1      1     1     1                0                 1
2      1     1        1      1     1     0                0                 1
2      1     1        1      1     1     0                0                 1
       0     0        0      0     0     4               15                22
   kastrationsstatus hunde alter_katze
30                 1     1           0   1
15                 1     0           0   2
22                 0     0           0   4
10                 1     1           0   2
1                  0     1           0   3
2                  1     1           1   2
2                  1     1           0   3
                  23    37          80 181
```

```
vis_miss(cat_sub)
```

```
colSums(is.na(cat_sub)) / nrow(cat_sub)
```

```
           versuch   geschlecht_katze              rasse             umfeld
        0.00000000         0.00000000         0.00000000         0.00000000
  aufenthaltsgrund   aufenthaltsdauer           freigang  kastrationsstatus
        0.00000000         0.18292683         0.00000000         0.28048780
mehrkatzenhaushalt              hunde        alter_katze  geschlecht_halter
        0.00000000         0.45121951         0.97560976         0.26829268
             exp_1              ply_1           passiv_1             tuer_1
        0.00000000         0.00000000         0.00000000         0.00000000
             con_1              exp_2              ply_2           passiv_2
        0.00000000         0.00000000         0.00000000         0.00000000
            tuer_2              con_2              exp_3              ply_3
        0.00000000         0.00000000         0.00000000         0.00000000
          passiv_3             tuer_3              con_3              exp_4
        0.00000000         0.00000000         0.00000000         0.00000000
             ply_4           passiv_4             tuer_4              con_4
        0.04878049         0.00000000         0.00000000         0.00000000
             exp_5              ply_5           passiv_5             tuer_5
        0.00000000         0.00000000         0.00000000         0.00000000
             con_5
        0.00000000
```

```
gg_miss_upset(cat_sub)
```

```
naniar::prop_miss_row(cat_sub)
```

```
 [1] 0.05405405 0.08108108 0.08108108 0.05405405 0.05405405 0.05405405
 [7] 0.05405405 0.08108108 0.05405405 0.05405405 0.05405405 0.05405405
[13] 0.05405405 0.05405405 0.05405405 0.05405405 0.05405405 0.05405405
[19] 0.05405405 0.05405405 0.05405405 0.05405405 0.05405405 0.05405405
[25] 0.05405405 0.05405405 0.05405405 0.05405405 0.05405405 0.05405405
[31] 0.02702703 0.02702703 0.02702703 0.02702703 0.02702703 0.02702703
[37] 0.02702703 0.02702703 0.02702703 0.02702703 0.02702703 0.02702703
[43] 0.02702703 0.02702703 0.02702703 0.02702703 0.02702703 0.02702703
[49] 0.02702703 0.02702703 0.02702703 0.02702703 0.02702703 0.02702703
[55] 0.02702703 0.02702703 0.02702703 0.02702703 0.02702703 0.02702703
[61] 0.10810811 0.10810811 0.10810811 0.10810811 0.10810811 0.10810811
[67] 0.10810811 0.10810811 0.10810811 0.10810811 0.10810811 0.10810811
[73] 0.10810811 0.10810811 0.10810811 0.10810811 0.10810811 0.10810811
[79] 0.10810811 0.10810811 0.10810811 0.10810811
```

```
md.pattern(cat_sub2)
```

```
   versuch geschlecht_katze rasse umfeld aufenthaltsgrund freigang
30       1                1     1      1                1        1
15       1                1     1      1                1        1
22       1                1     1      1                1        1
2        1                1     1      1                1        1
12       1                1     1      1                1        1
1        1                1     1      1                1        1
         0                0     0      0                0        0
   mehrkatzenhaushalt kontaktini_1 kontaktver_1 miauen_1 schnurren_1 fauchen_1
30                  1            1            1        1           1         1
15                  1            1            1        1           1         1
22                  1            1            1        1           1         1
2                   1            1            1        1           1         1
12                  1            1            1        1           1         1
1                   1            1            1        1           1         1
                    0            0            0        0           0         0
   knurren_1 kontaktini_2 kontaktver_2 miauen_2 schnurren_2 fauchen_2 knurren_2
30         1            1            1        1           1         1         1
15         1            1            1        1           1         1         1
22         1            1            1        1           1         1         1
2          1            1            1        1           1         1         1
12         1            1            1        1           1         1         1
1          1            1            1        1           1         1         1
           0            0            0        0           0         0         0
   kontaktini_3 kontaktver_3 miauen_3 schnurren_3 fauchen_3 knurren_3 miauen_4
30            1            1        1           1         1         1        1
15            1            1        1           1         1         1        1
22            1            1        1           1         1         1        1
2             1            1        1           1         1         1        1
12            1            1        1           1         1         1        1
1             1            1        1           1         1         1        1
              0            0        0           0         0         0        0
   schnurren_4 fauchen_4 knurren_4 kontaktini_5 kontaktver_5 miauen_5
30           1         1         1            1            1        1
15           1         1         1            1            1        1
22           1         1         1            1            1        1
2            1         1         1            1            1        1
12           1         1         1            1            1        1
1            1         1         1            1            1        1
             0         0         0            0            0        0
   schnurren_5 fauchen_5 knurren_5 kontaktini_4 kontaktver_4 aufenthaltsdauer
30           1         1         1            1            1                1
15           1         1         1            1            1                1
22           1         1         1            1            1                1
2            1         1         1            1            1                0
12           1         1         1            1            1                0
1            1         1         1            0            0                0
             0         0         0            1            1               15
   geschlecht_halter kastrationsstatus hunde alter_katze
30                 1                 1     1           0   1
15                 1                 1     0           0   2
22                 0                 0     0           0   4
2                  1                 1     1           1   1
12                 1                 1     1           0   2
1                  1                 0     1           0   5
                  22                23    37          80 179
```

```
vis_miss(cat_sub2)
```

```
colSums(is.na(cat_sub2)) / nrow(cat_sub2)
```

```
           versuch   geschlecht_katze              rasse             umfeld
        0.00000000         0.00000000         0.00000000         0.00000000
  aufenthaltsgrund   aufenthaltsdauer           freigang  kastrationsstatus
        0.00000000         0.18292683         0.00000000         0.28048780
mehrkatzenhaushalt              hunde        alter_katze  geschlecht_halter
        0.00000000         0.45121951         0.97560976         0.26829268
      kontaktini_1       kontaktver_1           miauen_1        schnurren_1
        0.00000000         0.00000000         0.00000000         0.00000000
         fauchen_1          knurren_1       kontaktini_2       kontaktver_2
        0.00000000         0.00000000         0.00000000         0.00000000
          miauen_2        schnurren_2          fauchen_2          knurren_2
        0.00000000         0.00000000         0.00000000         0.00000000
      kontaktini_3       kontaktver_3           miauen_3        schnurren_3
        0.00000000         0.00000000         0.00000000         0.00000000
         fauchen_3          knurren_3       kontaktini_4       kontaktver_4
        0.00000000         0.00000000         0.01219512         0.01219512
          miauen_4        schnurren_4          fauchen_4          knurren_4
        0.00000000         0.00000000         0.00000000         0.00000000
      kontaktini_5       kontaktver_5           miauen_5        schnurren_5
        0.00000000         0.00000000         0.00000000         0.00000000
         fauchen_5          knurren_5
        0.00000000         0.00000000
```

```
gg_miss_upset(cat_sub2)
```

```
naniar::prop_miss_row(cat_sub2)
```

[illegible]

```
 [1] 0.04761905 0.04761905 0.04761905 0.04761905 0.04761905 0.02380952
 [7] 0.04761905 0.11904762 0.04761905 0.04761905 0.04761905 0.02380952
[13] 0.04761905 0.04761905 0.04761905 0.04761905 0.04761905 0.04761905
[19] 0.04761905 0.04761905 0.04761905 0.04761905 0.04761905 0.04761905
[25] 0.04761905 0.04761905 0.04761905 0.04761905 0.04761905 0.04761905
[31] 0.02380952 0.02380952 0.02380952 0.02380952 0.02380952 0.02380952
[37] 0.02380952 0.02380952 0.02380952 0.02380952 0.02380952 0.02380952
[43] 0.02380952 0.02380952 0.02380952 0.02380952 0.02380952 0.02380952
[49] 0.02380952 0.02380952 0.02380952 0.02380952 0.02380952 0.02380952
[55] 0.02380952 0.02380952 0.02380952 0.02380952 0.02380952 0.02380952
[61] 0.09523810 0.09523810 0.09523810 0.09523810 0.09523810 0.09523810
[67] 0.09523810 0.09523810 0.09523810 0.09523810 0.09523810 0.09523810
[73] 0.09523810 0.09523810 0.09523810 0.09523810 0.09523810 0.09523810
[79] 0.09523810 0.09523810 0.09523810 0.09523810
```

Variablen

```
column_missing_percentage <- colMeans(is.na(cat)) * 100
column_missing_percentage_ordered <- sort(column_missing_percentage, decreasing = TRUE)

# Show the result
column_missing_percentage_ordered
```

```
       alter_katze          ply_n_b_2          ply_t_b_2          con_n_b_2
         97.560976          59.756098          59.756098          54.878049
          con_t_b_2     kontakt_pro_b_2    kontakt_con_b_2    kontakt_sum_b_2
          54.878049          54.878049          54.878049          54.878049
       verz_kont_b_2    kont_t_start_b_2        verz_kont_3     kont_t_start_3
          54.878049          54.878049          54.878049          54.878049
          ply_n_f_4           ply_t_f_42          con_n_f_4          con_t_f_4
          54.878049          54.878049          54.878049          54.878049
     kontakt_pro_f_4     kontakt_con_f_4    kontakt_sum_f_4        verz_kont_f_4
          54.878049          54.878049          54.878049          54.878049
    kont_t_start_f_4               hunde        verz_kont_b_4   kont_t_start_b_4
          54.878049          45.121951          37.804878          37.804878
         verz_kont_5      kont_t_start_5   kastrationsstatus   geschlecht_halter
          37.804878          37.804878          28.048780          26.829268
     aufenthaltsdauer           ply_n_b_4          ply_t_b_4     kontakt_pro_b_4
          18.292683           4.878049           4.878049           1.219512
     kontakt_con_b_4     kontakt_sum_b_4             versuch         name_tester
           1.219512           1.219512           0.000000           0.000000
            testjahr          name_katze    geschlecht_katze               rasse
           0.000000           0.000000           0.000000           0.000000
              umfeld   aufenthaltsgrund            freigang  mehrkatzenhaushalt
           0.000000           0.000000           0.000000           0.000000
             episode         videodauer_1       personentyp_1             exp_n_1
           0.000000           0.000000           0.000000           0.000000
             exp_t_1             ply_n_1             ply_t_1          passiv_n_1
           0.000000           0.000000           0.000000           0.000000
          passiv_t_1            tuer_n_1            tuer_t_1             con_n_1
           0.000000           0.000000           0.000000           0.000000
             con_t_1       kontakt_pro_1       kontakt_con_1       kontakt_sum_1
           0.000000           0.000000           0.000000           0.000000
         verz_kont_1      kont_t_start_1       lau_pro_miau_1     lau_pro_schnu_1
           0.000000           0.000000           0.000000           0.000000
        lau_con_fau_1        lau_con_knu_1           lau_sum_1            episode2
           0.000000           0.000000           0.000000           0.000000
        videodauer_2       personentyp_2             exp_n_2             exp_t_2
           0.000000           0.000000           0.000000           0.000000
           ply_n_f_2           ply_t_f_2          passiv_n_2          passiv_t_2
           0.000000           0.000000           0.000000           0.000000
            tuer_n_2            tuer_t_2           con_n_f_2           con_t_f_2
           0.000000           0.000000           0.000000           0.000000
     kontakt_pro_f_2     kontakt_con_f_2     kontakt_sum_f_2       verz_kont_f_2
           0.000000           0.000000           0.000000           0.000000
    kont_t_start_f_2      lau_pro_miau_2     lau_pro_schnu_2       lau_con_fau_2
           0.000000           0.000000           0.000000           0.000000
        lau_con_knu_2           lau_sum_2            episode3        videodauer_3
           0.000000           0.000000           0.000000           0.000000
       personentyp_3             exp_n_3             exp_t_3             ply_n_3
           0.000000           0.000000           0.000000           0.000000
             ply_t_3          passiv_n_3          passiv_t_3            tuer_n_3
           0.000000           0.000000           0.000000           0.000000
            tuer_t_3             con_n_3             con_t_3       kontakt_pro_3
           0.000000           0.000000           0.000000           0.000000
       kontakt_con_3       kontakt_sum_3      lau_pro_miau_3     lau_pro_schnu_3
           0.000000           0.000000           0.000000           0.000000
       lau_con_fau_3       lau_con_knu_3           lau_sum_3            episode4
           0.000000           0.000000           0.000000           0.000000
        videodauer_4       personentyp_4             exp_n_4             exp_t_4
           0.000000           0.000000           0.000000           0.000000
          passiv_n_4          passiv_t_4            tuer_n_4            tuer_t_4
           0.000000           0.000000           0.000000           0.000000
           con_n_b_4           con_t_b_4      lau_pro_miau_4     lau_pro_schnu_4
           0.000000           0.000000           0.000000           0.000000
       lau_con_fau_4      lau_con_knur_4           lau_sum_4            episode5
           0.000000           0.000000           0.000000           0.000000
        videodauer_5       personentyp_5             exp_n_5             exp_t_5
           0.000000           0.000000           0.000000           0.000000
             ply_n_5             ply_t_5          passiv_n_5          passiv_t_5
           0.000000           0.000000           0.000000           0.000000
            tuer_n_5            tuer_t_5             con_n_5             con_t_5
           0.000000           0.000000           0.000000           0.000000
       kontakt_pro_5       kontakt_con_5       kontakt_sum_5      lau_pro_miau_5
           0.000000           0.000000           0.000000           0.000000
     lau_pro_schnu_5       lau_con_fau_5      lau_con_knur_5           lau_sum_5
           0.000000           0.000000           0.000000           0.000000
               pilot
           0.000000
```

Katzen

```
# Calculate and order the percentage of missing data for each row
row_missing_percentage <- rowMeans(is.na(cat)) * 100

# Create a dataframe with `vpcode` and the percentage of missing data
vpcode_with_missing_percentage <- data.frame(
  vpcode = cat$versuch,
  missing_percentage = row_missing_percentage
)

# Order the dataframe by the missing percentage in descending order
vpcode_with_missing_percentage_ordered <- vpcode_with_missing_percentage[order(-vpcode_with_missing_percentage$missing_percentage), ]

# Show the result
vpcode_with_missing_percentage_ordered
```

```
   vpcode missing_percentage
23     23          17.449664
31     31          16.778523
32     32          16.778523
33     33          16.778523
34     34          16.778523
35     35          16.778523
36     36          16.778523
37     37          16.778523
38     38          16.778523
39     39          16.778523
40     40          16.778523
41     41          16.778523
42     42          16.778523
43     43          16.778523
44     44          16.778523
45     45          16.778523
46     46          16.778523
47     47          16.778523
48     48          16.778523
49     49          16.778523
50     50          16.778523
51     51          16.778523
52     52          16.778523
53     53          16.778523
54     54          16.778523
55     55          16.778523
56     56          16.778523
57     57          16.778523
58     58          16.778523
59     59          16.778523
60     60          16.778523
16     16          14.765101
17     17          14.765101
18     18          14.765101
19     19          14.765101
20     20          14.765101
21     21          14.765101
22     22          14.765101
24     24          14.765101
25     25          14.765101
26     26          14.765101
27     27          14.765101
28     28          14.765101
29     29          14.765101
30     30          14.765101
2       2           4.026846
8       8           4.026846
6       6           3.355705
12     12           3.355705
1       1           2.684564
3       3           2.684564
61     61           2.684564
62     62           2.684564
63     63           2.684564
64     64           2.684564
65     65           2.684564
66     66           2.684564
67     67           2.684564
68     68           2.684564
69     69           2.684564
70     70           2.684564
71     71           2.684564
72     72           2.684564
73     73           2.684564
74     74           2.684564
75     75           2.684564
76     76           2.684564
77     77           2.684564
78     78           2.684564
79     79           2.684564
80     80           2.684564
81     81           2.684564
82     82           2.684564
4       4           1.342282
5       5           1.342282
7       7           1.342282
9       9           1.342282
10     10           1.342282
11     11           1.342282
13     13           1.342282
14     14           1.342282
15     15           1.342282
```

Häufigkeitstabellen

```
table(cat$umfeld)
```

```
  Private Home Animal Shelter 
            67             15 
```

```
table(cat$rasse)
```

```
     EKH     Siam      BKH Maincoon   Perser      Mix      GHK 
      28        2        5        4        1       24       18 
```

```
table(cat$geschlecht_katze)
```

```
Female   Male 
    47     35 
```

```
table(cat$kastrationsstatus)
```

```
  intact Neutered 
       3       56 
```

```
table(cat$freigang)
```

```
Outdoor  Indoor 
     43      39 
```

```
table(cat$mehrkatzenhaushalt)
```

```
Single-Cat  Multi-Cat 
        63         19 
```

```
table(cat$geschlecht_halter)
```

```
Female   Male 
    54      6 
```

```
table(cat$hunde)
```

```
With Dog   No Dog 
       6       39 
```

```
table(cat$alter_katze)
```

```
   <3 Months   4-6 Months   7-9 Months 10-11 Months    1-3 Years    4-6 Years 
           0            0            0            0            0            0 
   7-9 Years    >10 Years 
           1            1 
```

```
df1 <- data.frame(Kastrationsstatus = c("kastriert", "intakt", "NA"))
df2 <- data.frame(Alter = c(56, 3, 23))

# Spaltenweise zusammenfügen
table2 <- cbind(df1, df2)
print(table2)
```

```
  Kastrationsstatus Alter
1         kastriert    56
2            intakt     3
3                NA    23
```

```
cat_demo_table <- tribble(
  ~Characteristic, ~N,

  # Sex
  "Sex", "",
  "  Female", "47",
  "  Male", "35",

  # Neuter status
  "Neuter status", "",
  "  Intact", "3",
  "  Neutered", "56",
  "  NA", "23",

  # Environment
  "Environment", "",
  "  Private household", "67",
  "  Shelter", "15",

  # Access outdoor
  "Outdoor access", "",
  "  Outdoor", "43",
  "  Indoor", "39",

  # Multicat home
  "Multicat home", "",
  "  Single-cat", "19",
  "  Multi-cat", "63",

  # Socialised with dog
  "Socialised with dog", "",
  "  With dog", "6",
  "  Without dog", "39",
  "  NA", "37",

)




# Formatieren mit flextable
ft <- flextable(cat_demo_table) %>%
  theme_booktabs() %>%
  autofit() %>%
  bold(i = ~Characteristic != "", bold = TRUE, part = "body") %>%
  align(align = "left", part = "all") %>%
  set_header_labels(Characteristic = "Characteristic", N = "n")



ft <- flextable(cat_demo_table) %>%
  theme_booktabs() %>%
  autofit() %>%
  # Nur Hauptkategorien fett machen (nicht eingerückt)
  bold(i = ~!startsWith(Characteristic, "  "), bold = TRUE, part = "body") %>%
  align(align = "left", part = "all")



ft <- flextable(cat_demo_table) %>%
  theme_booktabs() %>%
  autofit() %>%
  bold(i = ~!startsWith(Characteristic, "  "), bold = TRUE) %>%
  align(align = "left", part = "all") %>%
  set_header_labels(Characteristic = "Characteristic", Wert = "n") %>%
  line_spacing(space = 0.6)  # 🔽 reduziert Zeilenhöhe (Standard ist 1.0)

ft
```

Characteristic	N
**Sex**	
Female	47
Male	35
**Neuter status**	
Intact	3
Neutered	56
NA	23
**Environment**	
Private household	67
Shelter	15
**Outdoor access**	
Outdoor	43
Indoor	39
**Multicat home**	
Single-cat	19
Multi-cat	63
**Socialised with dog**	
With dog	6
Without dog	39
NA	37

```
library(tibble)
library(flextable)

# Breed distribution table
breed_table <- tribble(
  ~Characteristic, ~n,

  "Breed", "",
  "  European Shorthair (ESH)", "46",
  "  Siamese", "2",
  "  British Shorthair (BSH)", "5",
  "  Maine Coon", "4",
  "  Persian", "5",
  "  Mixed breed", "20"
)

# Formatieren mit flextable
ft_breed <- flextable(breed_table) %>%
  theme_booktabs() %>%
  autofit() %>%
  bold(i = ~!startsWith(Characteristic, "  "), bold = TRUE) %>%
  align(align = "left", part = "all") %>%
  set_header_labels(Characteristic = "Characteristic", n = "n") %>%
  line_spacing(space = 0.6)

ft_breed
```

Characteristic	n
**Breed**	
European Shorthair (ESH)	46
Siamese	2
British Shorthair (BSH)	5
Maine Coon	4
Persian	5
Mixed breed	20

```
cat_age_table <- tribble(
  ~Characteristic, ~N,
  "Age (years)", "",
  "  <1 year", "7",
  "  1-3", "32",
  "  4-6", "13",
  "  7-9", "18",
  "  >10", "12"
)

ft_age <- flextable(cat_age_table) %>%
  theme_booktabs() %>%
  autofit() %>%
  bold(i = ~!startsWith(Characteristic, "  "), bold = TRUE) %>%
  align(align = "left", part = "all") %>%
  set_header_labels(Characteristic = "Characteristic", N = "n") %>%
  line_spacing(space = 0.6)

ft_age
```

Characteristic	n
**Age (years)**	
<1 year	7
1–3	32
4–6	13
7–9	18
>10	12

Mittelwerte Verhalten

Subset 1

```
mosaic::favstats(sec ~ verhalten + zeit, data=cat_long)
```

```
   verhalten.zeit min    Q1 median     Q3 max     mean       sd  n missing
1           con.1   0 10.25   33.5  74.75 184 51.40244 50.86799 82       0
2           exp.1   0 30.25   70.0 112.25 196 73.10976 51.37949 82       0
3        passiv.1   0  0.00   15.0  52.00 199 36.58537 46.78502 82       0
4           ply.1   0  0.00    0.0  24.25 176 18.82927 35.86732 82       0
5          tuer.1   0  0.00    0.0  19.75 189 15.84146 29.50517 82       0
6           con.2   0  6.25   26.0  68.25 155 41.09756 41.39052 82       0
7           exp.2   0 21.25   62.0 118.75 193 73.95122 58.46575 82       0
8        passiv.2   0  3.00   28.5  71.50 191 45.56098 51.34418 82       0
9           ply.2   0  0.00    0.0  12.75 138 16.18293 31.07582 82       0
10         tuer.2   0  0.00    0.0  15.75 196 13.96341 29.81070 82       0
11          con.3   0  3.00   17.0  50.75 180 32.69512 40.89966 82       0
12          exp.3   0 22.00   53.0 102.75 180 63.92683 50.70632 82       0
13       passiv.3   0  3.00   37.0  78.50 199 49.02439 49.63720 82       0
14          ply.3   0  0.00    0.0  33.25 159 23.80488 39.55788 82       0
15         tuer.3   0  0.00   11.0  37.25 181 24.98780 35.14256 82       0
16          con.4   0  1.25   13.5  44.75 130 29.69512 36.11972 82       0
17          exp.4   0 16.25   60.5  87.25 180 61.47561 49.33664 82       0
18       passiv.4   0  6.50   37.5  68.00 187 47.23171 50.23925 82       0
19          ply.4   0  0.00    0.0  69.00 165 32.92308 48.81073 78       4
20         tuer.4   0  0.00    0.0   4.75 135 11.51220 25.68866 82       0
21          con.5   0  2.25   19.0  54.00 180 33.58537 38.53680 82       0
22          exp.5   0 21.25   59.5 103.50 187 66.20732 51.50111 82       0
23       passiv.5   0  4.00   26.5  83.75 184 47.41463 53.72379 82       0
24          ply.5   0  0.00    0.0  20.00 169 23.68293 43.87662 82       0
25         tuer.5   0  0.00    2.0  17.00 125 16.26829 28.79217 82       0
```

```
table1 <- mosaic::favstats(sec ~ zeit + verhalten, data=cat_long)

write.csv(table1, "tabele1.csv", row.names = TRUE)

table_mean1<-mosaic::favstats(sec ~ zeit + verhalten + umfeld, data=cat_long)

str(table_mean1)
```

```
'data.frame':	50 obs. of  10 variables:
 $ zeit.verhalten.umfeld: chr  "1.con.Private Home" "2.con.Private Home" "3.con.Private Home" "4.con.Private Home" ...
 $ min                  : num  0 0 0 0 0 0 0 0 0 0 ...
 $ Q1                   : num  9 8 3 1 0 29.5 25.5 21 12.5 17 ...
 $ median               : num  33 25 16 11 15 70 65 43 60 49 ...
 $ Q3                   : num  63 58 44 40.5 48.5 ...
 $ max                  : num  184 122 122 123 108 196 193 170 160 187 ...
 $ mean                 : num  43.9 35.1 26.6 28.1 28.1 ...
 $ sd                   : num  45.2 33.9 28.2 34.9 31.6 ...
 $ n                    : int  67 67 67 67 67 67 67 67 67 67 ...
 $ missing              : int  0 0 0 0 0 0 0 0 0 0 ...
```

```
# Spalte "zeit.verhalten.umfeld" in drei separate Spalten aufteilen und in table_mean1 speichern
table_mean1 <- table_mean1 %>%
  separate(zeit.verhalten.umfeld, into = c("zeit", "verhalten", "umfeld"), sep = "\\.")

# Ergebnis überprüfen
head(table_mean1)
```

```
  zeit verhalten       umfeld min   Q1 median    Q3 max     mean       sd  n
1    1       con Private Home   0  9.0     33  63.0 184 43.85075 45.17483 67
2    2       con Private Home   0  8.0     25  58.0 122 35.11940 33.89088 67
3    3       con Private Home   0  3.0     16  44.0 122 26.61194 28.22526 67
4    4       con Private Home   0  1.0     11  40.5 123 28.10448 34.91771 67
5    5       con Private Home   0  0.0     15  48.5 108 28.11940 31.64171 67
6    1       exp Private Home   0 29.5     70 109.5 196 69.95522 50.43911 67
  missing
1       0
2       0
3       0
4       0
5       0
6       0
```

```
# Daten vorbereiten und Mittelwert ± Standardabweichung formatieren
table_summary <- table_mean1 %>%
  dplyr::select(zeit, verhalten, umfeld, mean, sd, median, n, missing, Q1, Q3) %>%
  dplyr::mutate(
    mean_sd = paste0(round(mean, 2), " ± ", round(sd, 2)),  # Kombination von Mittelwert und SD
    median = round(median, 2)
  ) %>%
  dplyr::select(zeit, verhalten, umfeld, mean_sd, median)

# Umstrukturieren für den Flex Table mit zwei Headern (Umfeld > Zeitpunkte)
table_wide <- table_summary %>%
  pivot_wider(names_from = zeit, values_from = c(mean_sd, median))

# Erstellen des Flex Tables mit zwei Headerzeilen
flextable(table_wide) %>%
  set_header_labels(.,
                    umfeld = "Umfeld",
                    mean_sd_1 = "Mean ± SD", median_1 = "Median",
                    mean_sd_2 = "Mean ± SD", median_2 = "Median",
                    mean_sd_3 = "Mean ± SD", median_3 = "Median",
                    mean_sd_4 = "Mean ± SD", median_4 = "Median",
                    mean_sd_5 = "Mean ± SD", median_5 = "Median") %>%
  add_header(umfeld = " ", mean_sd_1 = "Zeit 1", mean_sd_2 = "Zeit 2",
             mean_sd_3 = "Zeit 3", mean_sd_4 = "Zeit 4", mean_sd_5 = "Zeit 5") %>%
  merge_h(part = "header") %>%
  theme_vanilla() %>%
  autofit()
```

		Zeit 1	Zeit 2	Zeit 3	Zeit 4	Zeit 5					
**verhalten**	**Umfeld**	**Mean ± SD**					**Median**				
con	Private Home	43.85 ± 45.17	35.12 ± 33.89	26.61 ± 28.23	28.1 ± 34.92	28.12 ± 31.64	33	25	16	11	15
exp	Private Home	69.96 ± 50.44	72.39 ± 55.08	57.57 ± 46.73	56.07 ± 43.97	60.46 ± 49.1	70	65	43	60	49
passiv	Private Home	43.67 ± 48.74	52.43 ± 51.38	54.58 ± 50.65	51.6 ± 52.32	52.94 ± 55.78	27	38	44	41	31
ply	Private Home	21.55 ± 37.92	18.07 ± 32.93	26.04 ± 39.39	38.28 ± 50.72	27.81 ± 47.13	0	0	0	0	0
tuer	Private Home	17.57 ± 30.94	14.9 ± 29.9	28.06 ± 37.13	11.85 ± 23.54	15.76 ± 28.19	0	0	13	0	0
con	Animal Shelter	85.13 ± 62.11	67.8 ± 59.68	59.87 ± 70.39	36.8 ± 41.64	58 ± 55.59	93	66	18	20	37
exp	Animal Shelter	87.2 ± 54.95	80.93 ± 73.54	92.33 ± 59.36	85.6 ± 64.85	91.87 ± 55.83	73	53	107	71	94
passiv	Animal Shelter	4.93 ± 12.32	14.87 ± 39.64	24.2 ± 36.76	27.73 ± 34.61	22.73 ± 35.11	0	0	0	12	10
ply	Animal Shelter	6.67 ± 21.66	7.73 ± 19.52	13.8 ± 40.12	0.27 ± 0.9	5.27 ± 14.94	0	0	0	0	0
tuer	Animal Shelter	8.13 ± 21.09	9.8 ± 30.05	11.27 ± 19.96	10 ± 34.67	18.53 ± 32.29	0	0	0	0	10

```
# Daten vorbereiten und Mittelwert ± Standardabweichung formatieren
table_summary <- table_mean1 %>%
  dplyr::select(zeit, verhalten, umfeld, mean, sd, median, n, missing, Q1, Q3) %>%
  dplyr::mutate(
    mean_sd = paste0(round(mean, 2), " ± ", round(sd, 2)),  # Kombination von Mittelwert und SD
    median = round(median, 2)
  ) %>%
  dplyr::select(zeit, verhalten, umfeld, mean_sd, median)

# Umstrukturieren für den Flex Table mit zwei Headern (Umfeld > Zeitpunkte)
table_wide <- table_summary %>%
  pivot_wider(names_from = zeit, values_from = c(mean_sd, median))

# Erstellen des Flex Tables mit geteiltem Header für "Umfeld" und "Verhalten"
flextable(table_wide) %>%
  set_header_labels(
    umfeld = "Umfeld",
    verhalten = "Verhalten",
    mean_sd_1 = "Mean ± SD", median_1 = "Median",
    mean_sd_2 = "Mean ± SD", median_2 = "Median",
    mean_sd_3 = "Mean ± SD", median_3 = "Median",
    mean_sd_4 = "Mean ± SD", median_4 = "Median",
    mean_sd_5 = "Mean ± SD", median_5 = "Median"
  ) %>%
  # Setzen des geteilten Headers für "Umfeld" über die Metriken und Zeitpunkte
  add_header(
    umfeld = "Umfeld", verhalten = "Verhalten",
    mean_sd_1 = "Zeit 1", median_1 = "Zeit 1",
    mean_sd_2 = "Zeit 2", median_2 = "Zeit 2",
    mean_sd_3 = "Zeit 3", median_3 = "Zeit 3",
    mean_sd_4 = "Zeit 4", median_4 = "Zeit 4",
    mean_sd_5 = "Zeit 5", median_5 = "Zeit 5"
  ) %>%
  # Zusammenführen der Header-Zeilen für eine saubere Darstellung
  add_header(
    mean_sd_1 = "Mean ± SD", median_1 = "Median",
    mean_sd_2 = "Mean ± SD", median_2 = "Median",
    mean_sd_3 = "Mean ± SD", median_3 = "Median",
    mean_sd_4 = "Mean ± SD", median_4 = "Median",
    mean_sd_5 = "Mean ± SD", median_5 = "Median"
  ) %>%
  merge_h(part = "header") %>%
  theme_vanilla() %>%

    autofit()
```

		Mean ± SD					Median				
**Verhalten**	**Umfeld**	**Zeit 1**	**Zeit 2**	**Zeit 3**	**Zeit 4**	**Zeit 5**	**Zeit 1**	**Zeit 2**	**Zeit 3**	**Zeit 4**	**Zeit 5**
**Verhalten**	**Umfeld**	**Mean ± SD**					**Median**				
con	Private Home	43.85 ± 45.17	35.12 ± 33.89	26.61 ± 28.23	28.1 ± 34.92	28.12 ± 31.64	33	25	16	11	15
exp	Private Home	69.96 ± 50.44	72.39 ± 55.08	57.57 ± 46.73	56.07 ± 43.97	60.46 ± 49.1	70	65	43	60	49
passiv	Private Home	43.67 ± 48.74	52.43 ± 51.38	54.58 ± 50.65	51.6 ± 52.32	52.94 ± 55.78	27	38	44	41	31
ply	Private Home	21.55 ± 37.92	18.07 ± 32.93	26.04 ± 39.39	38.28 ± 50.72	27.81 ± 47.13	0	0	0	0	0
tuer	Private Home	17.57 ± 30.94	14.9 ± 29.9	28.06 ± 37.13	11.85 ± 23.54	15.76 ± 28.19	0	0	13	0	0
con	Animal Shelter	85.13 ± 62.11	67.8 ± 59.68	59.87 ± 70.39	36.8 ± 41.64	58 ± 55.59	93	66	18	20	37
exp	Animal Shelter	87.2 ± 54.95	80.93 ± 73.54	92.33 ± 59.36	85.6 ± 64.85	91.87 ± 55.83	73	53	107	71	94
passiv	Animal Shelter	4.93 ± 12.32	14.87 ± 39.64	24.2 ± 36.76	27.73 ± 34.61	22.73 ± 35.11	0	0	0	12	10
ply	Animal Shelter	6.67 ± 21.66	7.73 ± 19.52	13.8 ± 40.12	0.27 ± 0.9	5.27 ± 14.94	0	0	0	0	0
tuer	Animal Shelter	8.13 ± 21.09	9.8 ± 30.05	11.27 ± 19.96	10 ± 34.67	18.53 ± 32.29	0	0	0	0	10

```
#### Tabelle 1 Chris


# Daten vorbereiten mit Mittelwert ± Standardabweichung und Median (Q1-Q3)
table_summary <- table_mean1 %>%
  # Spezielle Behandlung für ply in Animal Shelter
  mutate(
    # Wenn ply in Animal Shelter mit n=11 (Zeit 4), nehme die Werte, aber zeige n=15
    n = ifelse(verhalten == "ply" & umfeld == "Animal Shelter" & n == 11, 15, n)
  ) %>%
  dplyr::select(zeit, umfeld, verhalten, mean, sd, median, Q1, Q3, n, missing) %>%
  dplyr::mutate(
    mean_sd = paste0(round(mean, 1), " ± ", round(sd, 1)),  # Kombination von Mittelwert und SD
    median_iqr = paste0(round(median, 1), " (", round(Q1, 1), " - ", round(Q3, 1), ")") # Median (Q1-Q3)
  ) %>%
  dplyr::select(zeit, umfeld, verhalten, mean_sd, median_iqr, n)

# Hinzufügen einer Markierung für ply in Zeit 4
table_summary <- table_summary %>%
  mutate(
    mean_sd = ifelse(verhalten == "ply" & umfeld == "Animal Shelter" & zeit == 4,
                     paste0(mean_sd, "*"),
                     mean_sd),
    median_iqr = ifelse(verhalten == "ply" & umfeld == "Animal Shelter" & zeit == 4,
                        paste0(median_iqr, "*"),
                        median_iqr)
  )

table_summary <- table_summary %>%
  mutate(
    verhalten = case_when(
      verhalten == "con" ~ "physical contact",
      verhalten == "exp" ~ "exploration behaviour",
      verhalten == "passiv" ~ "passive behaviour",
      verhalten == "ply" ~ "play behaviour",
      verhalten == "tuer" ~ "door behaviour",
      TRUE ~ verhalten
    )
  )



# Umstrukturieren für den Flex Table mit Zeitpunkten als Spalten
table_wide <- table_summary %>%
  pivot_wider(
    id_cols = c(umfeld, verhalten, n),
    names_from = zeit,
    values_from = c(mean_sd, median_iqr)
  )

# Spaltenreihenfolge so anpassen, dass mean_sd und median_iqr für jeden Zeitpunkt nebeneinander stehen
table_wide <- table_wide %>%
  dplyr::select(
    umfeld, verhalten, n,
    mean_sd_1, median_iqr_1,
    mean_sd_2, median_iqr_2,
    mean_sd_3, median_iqr_3,
    mean_sd_4, median_iqr_4,
    mean_sd_5, median_iqr_5
  )

# Erstellen des Flex Tables mit strukturiertem Header für Zeit und Metriken
ft <- flextable(table_wide) %>%
  # Setzen der Spaltenbeschriftungen
  set_header_labels(
    umfeld = "Living Environment",
    verhalten = "Behaviour",
    n = "n",
    mean_sd_1 = "Mean ± SD",
    median_iqr_1 = "Median (Q1 - Q3)",
    mean_sd_2 = "Mean ± SD",
    median_iqr_2 = "Median (Q1 - Q3)",
    mean_sd_3 = "Mean ± SD",
    median_iqr_3 = "Median (Q1 - Q3)",
    mean_sd_4 = "Mean ± SD",
    median_iqr_4 = "Median (Q1 - Q3)",
    mean_sd_5 = "Mean ± SD",
    median_iqr_5 = "Median (Q1 - Q3)"
  ) %>%
  # Hinzufügen einer Kopfzeile für die Zeitpunkte
  add_header_row(
    values = c("", "", "",
               "Video Sequence 1", "Video Sequence 1",
               "Video Sequence 2", "Video Sequence 2",
               "Video Sequence 3", "Video Sequence 3",
               "Video Sequence 4", "Video Sequence 4",
               "Video Sequence 5", "Video Sequence 5"),
    colwidths = c(1, 1, 1, 1, 1, 1, 1, 1, 1, 1, 1, 1, 1)
  ) %>%
  # Zusammenführen der Header-Zellen für Zeit
  merge_h_range(i = 1, j1 = 4, j2 = 5, part = "header") %>%
  merge_h_range(i = 1, j1 = 6, j2 = 7, part = "header") %>%
  merge_h_range(i = 1, j1 = 8, j2 = 9, part = "header") %>%
  merge_h_range(i = 1, j1 = 10, j2 = 11, part = "header") %>%
  merge_h_range(i = 1, j1 = 12, j2 = 13, part = "header") %>%
  # Manuelles Zusammenführen der Umfeld-Zellen
  merge_v(j = "umfeld") %>%
  # Formatierung
  theme_vanilla() %>%
  align(align = "center", part = "header") %>%
  align(j = c("umfeld", "verhalten"), align = "left", part = "body") %>%
  align(j = "n", align = "center", part = "body") %>%
  vline(j = c(3, 5, 7, 9, 11), part = "all") %>%
  border_outer() %>%
  autofit()

# Hinzufügen einer Fußnote
ft <- ft %>%
  add_footer_lines("* for play behaviour in video sequence 4, the actual sample size is n=11") %>%
  fontsize(part = "footer", size = 9) %>%
  italic(part = "footer") %>%
  align(part = "footer", align = "left")

# Anzeigen der Tabelle
ft
```

			Video Sequence 1		Video Sequence 2		Video Sequence 3		Video Sequence 4		Video Sequence 5	
**Living Environment**	**Behaviour**	**n**	**Mean ± SD**	**Median (Q1 - Q3)**	**Mean ± SD**	**Median (Q1 - Q3)**	**Mean ± SD**	**Median (Q1 - Q3)**	**Mean ± SD**	**Median (Q1 - Q3)**	**Mean ± SD**	**Median (Q1 - Q3)**
Private Home	physical contact	67	43.9 ± 45.2	33 (9 - 63)	35.1 ± 33.9	25 (8 - 58)	26.6 ± 28.2	16 (3 - 44)	28.1 ± 34.9	11 (1 - 40.5)	28.1 ± 31.6	15 (0 - 48.5)
	exploration behaviour	67	70 ± 50.4	70 (29.5 - 109.5)	72.4 ± 55.1	65 (25.5 - 114)	57.6 ± 46.7	43 (21 - 92.5)	56.1 ± 44	60 (12.5 - 81.5)	60.5 ± 49.1	49 (17 - 99.5)
	passive behaviour	67	43.7 ± 48.7	27 (5 - 63.5)	52.4 ± 51.4	38 (9.5 - 86.5)	54.6 ± 50.7	44 (13 - 86)	51.6 ± 52.3	41 (8 - 72)	52.9 ± 55.8	31 (7 - 89.5)
	play behaviour	67	21.6 ± 37.9	0 (0 - 34.5)	18.1 ± 32.9	0 (0 - 29)	26 ± 39.4	0 (0 - 53)	38.3 ± 50.7	0 (0 - 75)	27.8 ± 47.1	0 (0 - 48.5)
	door behaviour	67	17.6 ± 30.9	0 (0 - 23.5)	14.9 ± 29.9	0 (0 - 21)	28.1 ± 37.1	13 (0 - 40)	11.9 ± 23.5	0 (0 - 8)	15.8 ± 28.2	0 (0 - 17.5)
Animal Shelter	physical contact	15	85.1 ± 62.1	93 (23.5 - 146.5)	67.8 ± 59.7	66 (5 - 116)	59.9 ± 70.4	18 (4 - 109.5)	36.8 ± 41.6	20 (5.5 - 54)	58 ± 55.6	37 (14 - 90.5)
	exploration behaviour	15	87.2 ± 54.9	73 (61.5 - 125)	80.9 ± 73.5	53 (15.5 - 160)	92.3 ± 59.4	107 (31 - 123)	85.6 ± 64.9	71 (26.5 - 136.5)	91.9 ± 55.8	94 (59 - 121.5)
	passive behaviour	15	4.9 ± 12.3	0 (0 - 5)	14.9 ± 39.6	0 (0 - 5.5)	24.2 ± 36.8	0 (0 - 50)	27.7 ± 34.6	12 (0 - 48.5)	22.7 ± 35.1	10 (0 - 37)
	play behaviour	15	6.7 ± 21.7	0 (0 - 0)	7.7 ± 19.5	0 (0 - 1.5)	13.8 ± 40.1	0 (0 - 3.5)	0.3 ± 0.9*	0 (0 - 0)*	5.3 ± 14.9	0 (0 - 0.5)
	door behaviour	15	8.1 ± 21.1	0 (0 - 4)	9.8 ± 30.1	0 (0 - 0)	11.3 ± 20	0 (0 - 14)	10 ± 34.7	0 (0 - 1)	18.5 ± 32.3	10 (0 - 17)

** for play behaviour in video sequence 4, the actual sample size is n=11*

```
#### Tabelle 2 Chris


library(dplyr)
library(tidyr)
library(flextable)

# Daten vorbereiten mit Mittelwert ± Standardabweichung und Median (Q1-Q3)
table_summary <- table_mean1 %>%
  # Spezielle Behandlung für ply in Animal Shelter
  mutate(
    # Wenn ply in Animal Shelter mit n=11 (Zeit 4), nehme die Werte, aber zeige n=15
    n = ifelse(verhalten == "ply" & umfeld == "Animal Shelter" & n == 11, 15, n)
  ) %>%
  dplyr::select(zeit, umfeld, verhalten, mean, sd, median, Q1, Q3, n, missing) %>%
  dplyr::mutate(
    mean_sd = paste0(round(mean, 1), " ± ", round(sd, 1)),  # Kombination von Mittelwert und SD
    median_iqr = paste0(round(median, 1), " (", round(Q1, 1), " - ", round(Q3, 1), ")") # Median (Q1-Q3)
  )

# Markiere ply in Zeit 4 mit einem Sternchen
table_summary <- table_summary %>%
  mutate(
    mean_sd = ifelse(verhalten == "ply" & umfeld == "Animal Shelter" & zeit == 4,
                     paste0(mean_sd, "*"),
                     mean_sd),
    median_iqr = ifelse(verhalten == "ply" & umfeld == "Animal Shelter" & zeit == 4,
                        paste0(median_iqr, "*"),
                        median_iqr)
  )

# Kombiniere Mean±SD und Median(Q1-Q3) in einer Zelle mit Zeilenumbruch
table_summary <- table_summary %>%
  mutate(
    combined_stats = paste0(mean_sd, "\n", median_iqr)
  )

# Pivot zu breiter Tabelle mit Zeitpunkten als Spalten
table_wide <- table_summary %>%
  dplyr::select(zeit, umfeld, verhalten, n, combined_stats) %>%
  pivot_wider(
    id_cols = c(umfeld, verhalten, n),
    names_from = zeit,
    values_from = combined_stats,
    names_prefix = "zeit_"
  )

# Erstellen des Flex Tables
ft <- flextable(table_wide) %>%
  # Setzen der Spaltenbeschriftungen
  set_header_labels(
    umfeld = "Umfeld",
    verhalten = "Behaviour",
    n = "n",
    zeit_1 = "Zeit 1",
    zeit_2 = "Zeit 2",
    zeit_3 = "Zeit 3",
    zeit_4 = "Zeit 4",
    zeit_5 = "Zeit 5"
  ) %>%
  # Kopfzeile mit zusätzlichen Informationen
  add_header_row(
    values = c("", "", "",
               "Mean ± SD\nMedian (Q1 - Q3)",
               "Mean ± SD\nMedian (Q1 - Q3)",
               "Mean ± SD\nMedian (Q1 - Q3)",
               "Mean ± SD\nMedian (Q1 - Q3)",
               "Mean ± SD\nMedian (Q1 - Q3)"),
    colwidths = c(1, 1, 1, 1, 1, 1, 1, 1)
  ) %>%
  # Zusammenführen von Zellen
  merge_v(j = "umfeld") %>%
  # Formatierung
  theme_vanilla() %>%
  align(j = c("umfeld", "verhalten"), align = "left", part = "body") %>%
  align(j = "n", align = "center", part = "body") %>%
  align(j = c("zeit_1", "zeit_2", "zeit_3", "zeit_4", "zeit_5"), align = "center", part = "body") %>%
  align(j = c("zeit_1", "zeit_2", "zeit_3", "zeit_4", "zeit_5"), align = "center", part = "header") %>%
  vline(j = c(3, 4, 5, 6, 7), part = "all") %>%
  border_outer() %>%
  # Layouteinstellungen, um Zeilenumbrüche anzuzeigen
  fontsize(size = 10, part = "all") %>%
  padding(padding = 4, part = "all") %>%
  autofit()

# Hinzufügen einer Fußnote
ft <- ft %>%
  add_footer_lines("* Für 'ply' in Zeit 4 war die tatsächliche Stichprobengröße n=11") %>%
  fontsize(part = "footer", size = 9) %>%
  italic(part = "footer") %>%
  align(part = "footer", align = "left")

# Anzeigen der Tabelle
ft
```

			Mean ± SD<br>Median (Q1 - Q3)	Mean ± SD<br>Median (Q1 - Q3)	Mean ± SD<br>Median (Q1 - Q3)	Mean ± SD<br>Median (Q1 - Q3)	Mean ± SD<br>Median (Q1 - Q3)

Umfeld	Behaviour	n	Zeit 1	Zeit 2	Zeit 3	Zeit 4	Zeit 5
Private Home	con	67	43.9 ± 45.2<br>33 (9 - 63)	35.1 ± 33.9<br>25 (8 - 58)	26.6 ± 28.2<br>16 (3 - 44)	28.1 ± 34.9<br>11 (1 - 40.5)	28.1 ± 31.6<br>15 (0 - 48.5)
	exp	67	70 ± 50.4<br>70 (29.5 - 109.5)	72.4 ± 55.1<br>65 (25.5 - 114)	57.6 ± 46.7<br>43 (21 - 92.5)	56.1 ± 44<br>60 (12.5 - 81.5)	60.5 ± 49.1<br>49 (17 - 99.5)
	passiv	67	43.7 ± 48.7<br>27 (5 - 63.5)	52.4 ± 51.4<br>38 (9.5 - 86.5)	54.6 ± 50.7<br>44 (13 - 86)	51.6 ± 52.3<br>41 (8 - 72)	52.9 ± 55.8<br>31 (7 - 89.5)
	ply	67	21.6 ± 37.9<br>0 (0 - 34.5)	18.1 ± 32.9<br>0 (0 - 29)	26 ± 39.4<br>0 (0 - 53)	38.3 ± 50.7<br>0 (0 - 75)	27.8 ± 47.1<br>0 (0 - 48.5)
	tuer	67	17.6 ± 30.9<br>0 (0 - 23.5)	14.9 ± 29.9<br>0 (0 - 21)	28.1 ± 37.1<br>13 (0 - 40)	11.9 ± 23.5<br>0 (0 - 8)	15.8 ± 28.2<br>0 (0 - 17.5)
Animal Shelter	con	15	85.1 ± 62.1<br>93 (23.5 - 146.5)	67.8 ± 59.7<br>66 (5 - 116)	59.9 ± 70.4<br>18 (4 - 109.5)	36.8 ± 41.6<br>20 (5.5 - 54)	58 ± 55.6<br>37 (14 - 90.5)
	exp	15	87.2 ± 54.9<br>73 (61.5 - 125)	80.9 ± 73.5<br>53 (15.5 - 160)	92.3 ± 59.4<br>107 (31 - 123)	85.6 ± 64.9<br>71 (26.5 - 136.5)	91.9 ± 55.8<br>94 (59 - 121.5)
	passiv	15	4.9 ± 12.3<br>0 (0 - 5)	14.9 ± 39.6<br>0 (0 - 5.5)	24.2 ± 36.8<br>0 (0 - 50)	27.7 ± 34.6<br>12 (0 - 48.5)	22.7 ± 35.1<br>10 (0 - 37)
	ply	15	6.7 ± 21.7<br>0 (0 - 0)	7.7 ± 19.5<br>0 (0 - 1.5)	13.8 ± 40.1<br>0 (0 - 3.5)	0.3 ± 0.9*<br>0 (0 - 0)*	5.3 ± 14.9<br>0 (0 - 0.5)
	tuer	15	8.1 ± 21.1<br>0 (0 - 4)	9.8 ± 30.1<br>0 (0 - 0)	11.3 ± 20<br>0 (0 - 14)	10 ± 34.7<br>0 (0 - 1)	18.5 ± 32.3<br>10 (0 - 17)

Für 'ply' in Zeit 4 war die tatsächliche Stichprobengröße n=11

```
#### Tabelle 3 Chris

library(dplyr)
library(tidyr)
library(flextable)

# Daten vorbereiten mit Mittelwert ± Standardabweichung und Median (Q1–Q3)
table_summary <- table_mean1 %>%
  # Spezielle Behandlung für ply in Animal Shelter
  dplyr::mutate(
    # Wenn ply in Animal Shelter mit n=11 (Zeit 4), nehme die Werte, aber zeige n=15
    n = ifelse(verhalten == "ply" & umfeld == "Animal Shelter" & n == 11, 15, n)
  ) %>%
  dplyr::select(zeit, umfeld, verhalten, mean, sd, median, Q1, Q3, n, missing) %>%
  dplyr::mutate(
    mean_sd = paste0(round(mean, 1), " ± ", round(sd, 1)),  # Kombination von Mittelwert und SD
    median_iqr = paste0(round(median, 1), " (", round(Q1, 1), " – ", round(Q3, 1), ")") # Median (Q1–Q3)
  )

# Markiere ply in Zeit 4 mit einem Sternchen
table_summary <- table_summary %>%
  dplyr::mutate(
    mean_sd = ifelse(verhalten == "ply" & umfeld == "Animal Shelter" & zeit == 4,
                     paste0(mean_sd, "*"),
                     mean_sd),
    median_iqr = ifelse(verhalten == "ply" & umfeld == "Animal Shelter" & zeit == 4,
                        paste0(median_iqr, "*"),
                        median_iqr)
  )

# Kombiniere Mean±SD und Median(Q1–Q3) in einer Zelle mit Zeilenumbruch
table_summary <- table_summary %>%
  dplyr::mutate(
    combined_stats = paste0(mean_sd, "\n", median_iqr)
  )

# Pivot zu breiter Tabelle mit Zeitpunkten als Spalten
table_wide <- table_summary %>%
  dplyr::select(zeit, umfeld, verhalten, combined_stats) %>%
  tidyr::pivot_wider(
    id_cols = c(umfeld, verhalten),
    names_from = zeit,
    values_from = combined_stats,
    names_prefix = "zeit_"
  )

# Erstelle neue Umfeld-Spalte mit n-Werten in zweiter Zeile
table_wide <- table_wide %>%
  dplyr::mutate(
    umfeld_with_n = dplyr::case_when(
      umfeld == "Private Home" ~ "Private Home\n(n=67)",
      umfeld == "Animal Shelter" ~ "Animal Shelter\n(n=15)",
      TRUE ~ umfeld
    )
  ) %>%
  dplyr::select(-umfeld) %>%
  dplyr::rename(umfeld = umfeld_with_n)

# Sortieren und neu anordnen der Spalten (Umfeld zuerst, dann Verhalten)
table_wide <- table_wide %>%
  dplyr::select(umfeld, verhalten, zeit_1, zeit_2, zeit_3, zeit_4, zeit_5)

# Erstellen des Flex Tables mit der richtigen Struktur von Anfang an
ft <- flextable::flextable(table_wide)

# Kompletter Neuaufbau des Headers mit Zeit oben und Mean/Median darunter
ft <- ft %>% flextable::set_header_df(
  data.frame(
    col_keys = c("umfeld", "verhalten", "zeit_1", "zeit_2", "zeit_3", "zeit_4", "zeit_5"),
    row1 = c("", "", "Zeit 1", "Zeit 2", "Zeit 3", "Zeit 4", "Zeit 5"),
    row2 = c("Umfeld", "Behaviour", "Mean ± SD\nMedian (Q1 – Q3)", "Mean ± SD\nMedian (Q1 – Q3)",
             "Mean ± SD\nMedian (Q1 – Q3)", "Mean ± SD\nMedian (Q1 – Q3)", "Mean ± SD\nMedian (Q1 – Q3)")
  ),
  key = "col_keys"
)

# Formatierung
ft <- ft %>%
  # Zusammenführen von Zellen
  flextable::merge_v(j = "umfeld") %>%
  # Formatierung
  flextable::theme_vanilla() %>%
  flextable::align(j = c("umfeld", "verhalten"), align = "left", part = "body") %>%
  flextable::align(j = c("zeit_1", "zeit_2", "zeit_3", "zeit_4", "zeit_5"), align = "center", part = "body") %>%
  flextable::align(j = c("zeit_1", "zeit_2", "zeit_3", "zeit_4", "zeit_5"), align = "center", part = "header") %>%
  flextable::vline(j = c(2, 3, 4, 5, 6), part = "all") %>%
  flextable::border_outer() %>%
  # Layouteinstellungen, um Zeilenumbrüche anzuzeigen
  flextable::fontsize(size = 10, part = "all") %>%
  flextable::padding(padding = 4, part = "all") %>%
  flextable::autofit()

# Hinzufügen einer Fußnote
ft <- ft %>%
  flextable::add_footer_lines("* Für 'ply' in Zeit 4 war die tatsächliche Stichprobengröße n=11") %>%
  flextable::fontsize(part = "footer", size = 9) %>%
  flextable::italic(part = "footer") %>%
  flextable::align(part = "footer", align = "left")

# Anzeigen der Tabelle
ft
```

		Zeit 1	Zeit 2	Zeit 3	Zeit 4	Zeit 5
Umfeld	Behaviour	Mean ± SD<br>Median (Q1 - Q3)	Mean ± SD<br>Median (Q1 - Q3)	Mean ± SD<br>Median (Q1 - Q3)	Mean ± SD<br>Median (Q1 - Q3)	Mean ± SD<br>Median (Q1 - Q3)
Private Home<br>(n=67)	con	43.9 ± 45.2<br>33 (9 - 63)	35.1 ± 33.9<br>25 (8 - 58)	26.6 ± 28.2<br>16 (3 - 44)	28.1 ± 34.9<br>11 (1 - 40.5)	28.1 ± 31.6<br>15 (0 - 48.5)
	exp	70 ± 50.4<br>70 (29.5 - 109.5)	72.4 ± 55.1<br>65 (25.5 - 114)	57.6 ± 46.7<br>43 (21 - 92.5)	56.1 ± 44<br>60 (12.5 - 81.5)	60.5 ± 49.1<br>49 (17 - 99.5)
	passiv	43.7 ± 48.7<br>27 (5 - 63.5)	52.4 ± 51.4<br>38 (9.5 - 86.5)	54.6 ± 50.7<br>44 (13 - 86)	51.6 ± 52.3<br>41 (8 - 72)	52.9 ± 55.8<br>31 (7 - 89.5)
	ply	21.6 ± 37.9<br>0 (0 - 34.5)	18.1 ± 32.9<br>0 (0 - 29)	26 ± 39.4<br>0 (0 - 53)	38.3 ± 50.7<br>0 (0 - 75)	27.8 ± 47.1<br>0 (0 - 48.5)
	tuer	17.6 ± 30.9<br>0 (0 - 23.5)	14.9 ± 29.9<br>0 (0 - 21)	28.1 ± 37.1<br>13 (0 - 40)	11.9 ± 23.5<br>0 (0 - 8)	15.8 ± 28.2<br>0 (0 - 17.5)
Animal Shelter<br>(n=15)	con	85.1 ± 62.1<br>93 (23.5 - 146.5)	67.8 ± 59.7<br>66 (5 - 116)	59.9 ± 70.4<br>18 (4 - 109.5)	36.8 ± 41.6<br>20 (5.5 - 54)	58 ± 55.6<br>37 (14 - 90.5)
	exp	87.2 ± 54.9<br>73 (61.5 - 125)	80.9 ± 73.5<br>53 (15.5 - 160)	92.3 ± 59.4<br>107 (31 - 123)	85.6 ± 64.9<br>71 (26.5 - 136.5)	91.9 ± 55.8<br>94 (59 - 121.5)
	passiv	4.9 ± 12.3<br>0 (0 - 5)	14.9 ± 39.6<br>0 (0 - 5.5)	24.2 ± 36.8<br>0 (0 - 50)	27.7 ± 34.6<br>12 (0 - 48.5)	22.7 ± 35.1<br>10 (0 - 37)
	ply	6.7 ± 21.7<br>0 (0 - 0)	7.7 ± 19.5<br>0 (0 - 1.5)	13.8 ± 40.1<br>0 (0 - 3.5)	0.3 ± 0.9*<br>0 (0 - 0)*	5.3 ± 14.9<br>0 (0 - 0.5)
	tuer	8.1 ± 21.1<br>0 (0 - 4)	9.8 ± 30.1<br>0 (0 - 0)	11.3 ± 20<br>0 (0 - 14)	10 ± 34.7<br>0 (0 - 1)	18.5 ± 32.3<br>10 (0 - 17)

Für 'ply' in Zeit 4 war die tatsächliche Stichprobengröße n=11

Subset 2

```
mosaic::favstats(anzahl ~ verhalten + zeit, data=cat_long2)

   verhalten.zeit min  Q1 median   Q3 max       mean        sd  n missing
1       fauchen.1   0 0.0    0.0  0.0   3 0.03658537 0.3312946 82       0
2       knurren.1   0 0.0    0.0  0.0   1 0.01219512 0.1104315 82       0
3    kontaktini.1   0 4.0    6.0 11.5  32 8.29268293 7.5170388 82       0
4    kontaktver.1   0 0.0    0.0  2.0  15 1.35365854 2.3953244 82       0
5        miauen.1   0 0.0    0.0  2.0  46 2.34146341 6.7535441 82       0
6     schnurren.1   0 0.0    2.0  2.0  12 1.70731707 2.3752209 82       0
7       fauchen.2   0 0.0    0.0  0.0   1 0.02439024 0.1552067 82       0
8       knurren.2   0 0.0    0.0  0.0   1 0.02439024 0.1552067 82       0
9    kontaktini.2   0 2.5    6.0  8.0  38 7.39024390 7.2260778 82       0
10   kontaktver.2   0 0.0    0.5  2.0  14 1.23170732 2.0202602 82       0
11       miauen.2   0 0.0    0.0  1.5  36 2.04878049 5.6040163 82       0
12    schnurren.2   0 0.0    0.0  2.0  12 1.48780488 2.5300364 82       0
13      fauchen.3   0 0.0    0.0  0.0   0 0.00000000 0.0000000 82       0
14      knurren.3   0 0.0    0.0  0.0   3 0.04878049 0.3479194 82       0
15   kontaktini.3   0 2.0    4.0  8.0  28 5.43902439 5.6679684 82       0
16   kontaktver.3   0 0.0    0.5  2.0   9 1.21951220 1.8055087 82       0
17       miauen.3   0 0.0    0.0  0.0  68 2.80487805 9.9324124 82       0
18    schnurren.3   0 0.0    0.0  2.0  10 1.00000000 1.6996732 82       0
19      fauchen.4   0 0.0    0.0  0.0   1 0.03658537 0.1888969 82       0
20      knurren.4   0 0.0    0.0  0.0   3 0.03658537 0.3312946 82       0
21   kontaktini.4   0 0.0    2.0  6.0  18 3.70370370 4.7128665 81       1
22   kontaktver.4   0 0.0    0.0  1.0  13 0.91358025 1.7832381 81       1
23       miauen.4   0 0.0    0.0  0.0  34 1.34146341 4.5547954 82       0
24    schnurren.4   0 0.0    0.0  2.0   8 0.78048780 1.5317576 82       0
25      fauchen.5   0 0.0    0.0  0.0   0 0.00000000 0.0000000 82       0
26      knurren.5   0 0.0    0.0  0.0   3 0.03658537 0.3312946 82       0
27   kontaktini.5   0 0.0    4.0  6.0  28 5.00000000 6.0797173 82       0
28   kontaktver.5   0 0.0    0.0  1.0   8 0.87804878 1.3643634 82       0
29       miauen.5   0 0.0    0.0  0.0  26 1.68292683 4.6024096 82       0
30    schnurren.5   0 0.0    0.0  2.0   8 1.31707317 1.8383974 82       0

table_mean2 <-mosaic::favstats(anzahl ~ zeit + verhalten, data=cat_long2)

mosaic::favstats(anzahl ~ zeit + verhalten + umfeld, data=cat_long2)

         zeit.verhalten.umfeld min Q1 median    Q3 max        mean         sd
1       1.fauchen.Private Home   0  0      0  0.00   0  0.00000000  0.0000000
2       2.fauchen.Private Home   0  0      0  0.00   1  0.01492537  0.1221694
3       3.fauchen.Private Home   0  0      0  0.00   0  0.00000000  0.0000000
4       4.fauchen.Private Home   0  0      0  0.00   1  0.02985075  0.1714598
5       5.fauchen.Private Home   0  0      0  0.00   0  0.00000000  0.0000000
6       1.knurren.Private Home   0  0      0  0.00   1  0.01492537  0.1221694
7       2.knurren.Private Home   0  0      0  0.00   1  0.01492537  0.1221694
8       3.knurren.Private Home   0  0      0  0.00   3  0.04477612  0.3665083
9       4.knurren.Private Home   0  0      0  0.00   3  0.04477612  0.3665083
10      5.knurren.Private Home   0  0      0  0.00   3  0.04477612  0.3665083
11   1.kontaktini.Private Home   0  4      6 10.00  30  7.16417910  5.9812764
12   2.kontaktini.Private Home   0  4      6  8.00  24  6.38805970  5.2827771
13   3.kontaktini.Private Home   0  2      4  6.00  22  5.04477612  4.6756391
14   4.kontaktini.Private Home   0  0      2  6.00  18  3.55223881  4.1020452
15   5.kontaktini.Private Home   0  0      4  6.00  28  4.44776119  5.5248299
16   1.kontaktver.Private Home   0  0      0  2.00   6  1.04477612  1.6370042
17   2.kontaktver.Private Home   0  0      1  2.00   7  1.14925373  1.5000377
18   3.kontaktver.Private Home   0  0      1  2.00   6  1.04477612  1.3192380
19   4.kontaktver.Private Home   0  0      0  2.00   4  0.86567164  1.2296282
20   5.kontaktver.Private Home   0  0      0  1.00   5  0.73134328  1.0528761
21       1.miauen.Private Home   0  0      0  2.00  46  2.53731343  7.2828740
22       2.miauen.Private Home   0  0      0  2.00  36  2.20895522  5.8167400
23       3.miauen.Private Home   0  0      0  0.00  68  2.77611940  9.6794392
24       4.miauen.Private Home   0  0      0  0.00  34  1.34328358  4.6760260
25       5.miauen.Private Home   0  0      0  0.00  26  1.94029851  5.0087215
26    1.schnurren.Private Home   0  0      0  2.00   8  1.16417910  1.5627968
27    2.schnurren.Private Home   0  0      0  2.00   6  0.95522388  1.4081240
28    3.schnurren.Private Home   0  0      0  2.00   6  0.80597015  1.3510804
29    4.schnurren.Private Home   0  0      0  1.00   6  0.59701493  1.1554837
30    5.schnurren.Private Home   0  0      0  2.00   8  1.10447761  1.6061843
31     1.fauchen.Animal Shelter   0  0      0  0.00   3  0.20000000  0.7745967
32     2.fauchen.Animal Shelter   0  0      0  0.00   1  0.06666667  0.2581989
33     3.fauchen.Animal Shelter   0  0      0  0.00   0  0.00000000  0.0000000
34     4.fauchen.Animal Shelter   0  0      0  0.00   1  0.06666667  0.2581989
35     5.fauchen.Animal Shelter   0  0      0  0.00   0  0.00000000  0.0000000
36     1.knurren.Animal Shelter   0  0      0  0.00   0  0.00000000  0.0000000
37     2.knurren.Animal Shelter   0  0      0  0.00   1  0.06666667  0.2581989
38     3.knurren.Animal Shelter   0  0      0  0.00   1  0.06666667  0.2581989
39     4.knurren.Animal Shelter   0  0      0  0.00   0  0.00000000  0.0000000
40     5.knurren.Animal Shelter   0  0      0  0.00   0  0.00000000  0.0000000
41 1.kontaktini.Animal Shelter   0  4     10 24.00  32 13.33333333 11.1782101
42 2.kontaktini.Animal Shelter   0  0     12 21.00  38 11.86666667 12.0111060
43 3.kontaktini.Animal Shelter   0  0      2 13.00  28  7.20000000  8.8737172
44 4.kontaktini.Animal Shelter   0  0      1  3.50  18  4.42857143  7.1113591
45 5.kontaktini.Animal Shelter   0  1      6 11.00  24  7.46666667  7.8727983
46 1.kontaktver.Animal Shelter   0  0      0  4.00  15  2.73333333  4.2504902
47 2.kontaktver.Animal Shelter   0  0      0  1.00  14  1.60000000  3.5816995
48 3.kontaktver.Animal Shelter   0  0      0  2.50   9  2.00000000  3.1396087
49 4.kontaktver.Animal Shelter   0  0      0  0.75  13  1.14285714  3.4386299
50 5.kontaktver.Animal Shelter   0  0      1  2.50   8  1.53333333  2.2318047
51      1.miauen.Animal Shelter   0  0      0  0.00  12  1.46666667  3.5830288
52      2.miauen.Animal Shelter   0  0      0  0.00  18  1.33333333  4.6393760
53      3.miauen.Animal Shelter   0  0      0  0.00  44  2.93333333 11.3607511
54      4.miauen.Animal Shelter   0  0      0  0.00  16  1.33333333  4.1173269
55      5.miauen.Animal Shelter   0  0      0  0.00   6  0.53333333  1.5976173
56   1.schnurren.Animal Shelter   0  1      4  6.00  12  4.13333333  3.6619017
57   2.schnurren.Animal Shelter   0  0      2  7.00  12  3.86666667  4.5018515
58   3.schnurren.Animal Shelter   0  0      2  2.00  10  1.86666667  2.6690466
59   4.schnurren.Animal Shelter   0  0      0  2.00   8  1.60000000  2.5298221
60   5.schnurren.Animal Shelter   0  0      2  4.00   6  2.26666667  2.4918916
    n missing
1  67       0
2  67       0
3  67       0
4  67       0
5  67       0
6  67       0
7  67       0
8  67       0
9  67       0
10 67       0
11 67       0
12 67       0
13 67       0
14 67       0
15 67       0
16 67       0
17 67       0
18 67       0
19 67       0
20 67       0
21 67       0
22 67       0
23 67       0
24 67       0
25 67       0
26 67       0
27 67       0
28 67       0
29 67       0
30 67       0
31 15       0
32 15       0
33 15       0
34 15       0
35 15       0
36 15       0
37 15       0
38 15       0
39 15       0
40 15       0
41 15       0
42 15       0
43 15       0
44 14       1
45 15       0
46 15       0
47 15       0
48 15       0
49 14       1
50 15       0
51 15       0
52 15       0
53 15       0
54 15       0
55 15       0
56 15       0
57 15       0
58 15       0
59 15       0
60 15       0

table_mean2

   zeit.verhalten min  Q1 median   Q3 max       mean        sd  n missing
1       1.fauchen   0 0.0    0.0  0.0   3 0.03658537 0.3312946 82       0
2       2.fauchen   0 0.0    0.0  0.0   1 0.02439024 0.1552067 82       0
3       3.fauchen   0 0.0    0.0  0.0   0 0.00000000 0.0000000 82       0
4       4.fauchen   0 0.0    0.0  0.0   1 0.03658537 0.1888969 82       0
5       5.fauchen   0 0.0    0.0  0.0   0 0.00000000 0.0000000 82       0
6       1.knurren   0 0.0    0.0  0.0   1 0.01219512 0.1104315 82       0
7       2.knurren   0 0.0    0.0  0.0   1 0.02439024 0.1552067 82       0
8       3.knurren   0 0.0    0.0  0.0   3 0.04878049 0.3479194 82       0
9       4.knurren   0 0.0    0.0  0.0   3 0.03658537 0.3312946 82       0
10      5.knurren   0 0.0    0.0  0.0   3 0.03658537 0.3312946 82       0
11   1.kontaktini   0 4.0    6.0 11.5  32 8.29268293 7.5170388 82       0
12   2.kontaktini   0 2.5    6.0  8.0  38 7.39024390 7.2260778 82       0
13   3.kontaktini   0 2.0    4.0  8.0  28 5.43902439 5.6679684 82       0
14   4.kontaktini   0 0.0    2.0  6.0  18 3.70370370 4.7128665 81       1
15   5.kontaktini   0 0.0    4.0  6.0  28 5.00000000 6.0797173 82       0
16   1.kontaktver   0 0.0    0.0  2.0  15 1.35365854 2.3953244 82       0
17   2.kontaktver   0 0.0    0.5  2.0  14 1.23170732 2.0202602 82       0
18   3.kontaktver   0 0.0    0.5  2.0   9 1.21951220 1.8055087 82       0
19   4.kontaktver   0 0.0    0.0  1.0  13 0.91358025 1.7832381 81       1
20   5.kontaktver   0 0.0    0.0  1.0   8 0.87804878 1.3643634 82       0
21       1.miauen   0 0.0    0.0  2.0  46 2.34146341 6.7535441 82       0
22       2.miauen   0 0.0    0.0  1.5  36 2.04878049 5.6040163 82       0
23       3.miauen   0 0.0    0.0  0.0  68 2.80487805 9.9324124 82       0
24       4.miauen   0 0.0    0.0  0.0  34 1.34146341 4.5547954 82       0
25       5.miauen   0 0.0    0.0  0.0  26 1.68292683 4.6024096 82       0
26    1.schnurren   0 0.0    2.0  2.0  12 1.70731707 2.3752209 82       0
27    2.schnurren   0 0.0    0.0  2.0  12 1.48780488 2.5300364 82       0
28    3.schnurren   0 0.0    0.0  2.0  10 1.00000000 1.6996732 82       0
29    4.schnurren   0 0.0    0.0  2.0   8 0.78048780 1.5317576 82       0
30    5.schnurren   0 0.0    0.0  2.0   8 1.31707317 1.8383974 82       0

# Spalte "zeit.verhalten.umfeld" in drei separate Spalten aufteilen und in table_mean2 speichern
table_mean2 <- table_mean2 %>%
  separate(zeit.verhalten, into = c("zeit", "verhalten"), sep = "\\.")

# Ergebnis überprüfen
head(table_mean2)

  zeit verhalten min Q1 median Q3 max       mean        sd  n missing
1    1   fauchen   0  0      0  0   3 0.03658537 0.3312946 82       0
2    2   fauchen   0  0      0  0   1 0.02439024 0.1552067 82       0
3    3   fauchen   0  0      0  0   0 0.00000000 0.0000000 82       0
4    4   fauchen   0  0      0  0   1 0.03658537 0.1888969 82       0
5    5   fauchen   0  0      0  0   0 0.00000000 0.0000000 82       0
6    1   knurren   0  0      0  0   1 0.01219512 0.1104315 82       0

# Daten vorbereiten und Mittelwert ± Standardabweichung formatieren
table_summary2 <- table_mean2 %>%
  dplyr::select(zeit, verhalten, mean, sd, median) %>%
  dplyr::mutate(
    mean_sd = paste0(round(mean, 2), " ± ", round(sd, 2)),  # Kombination von Mittelwert und SD
    median = round(median, 2)
  ) %>%
  dplyr::select(zeit, verhalten,  mean_sd, median)

# Umstrukturieren für den Flex Table mit zwei Headern (Umfeld > Zeitpunkte)
table_wide2 <- table_summary2 %>%
  pivot_wider(names_from = zeit, values_from = c(mean_sd, median))

# Erstellen des Flex Tables mit zwei Headerzeilen
flextable(table_wide2) %>%
  set_header_labels(.,
                    mean_sd_1 = "Mean ± SD", median_1 = "Median",
                    mean_sd_2 = "Mean ± SD", median_2 = "Median",
                    mean_sd_3 = "Mean ± SD", median_3 = "Median",
                    mean_sd_4 = "Mean ± SD", median_4 = "Median",
                    mean_sd_5 = "Mean ± SD", median_5 = "Median") %>%
  add_header( mean_sd_1 = "Zeit 1", mean_sd_2 = "Zeit 2",
             mean_sd_3 = "Zeit 3", mean_sd_4 = "Zeit 4", mean_sd_5 = "Zeit 5") %>%
  merge_h(part = "header") %>%
  theme_vanilla() %>%
  autofit()
```

	Zeit 1	Zeit 2	Zeit 3	Zeit 4	Zeit 5					
verhalten	Mean ± SD									Median
fauchen	0.04 ± 0.33	0.02 ± 0.16	0 ± 0	0.04 ± 0.19	0 ± 0	0	0.0	0.0	0	0
knurren	0.01 ± 0.11	0.02 ± 0.16	0.05 ± 0.35	0.04 ± 0.33	0.04 ± 0.33	0	0.0	0.0	0	0
kontaktini	8.29 ± 7.52	7.39 ± 7.23	5.44 ± 5.67	3.7 ± 4.71	5 ± 6.08	6	6.0	4.0	2	4
kontaktver	1.35 ± 2.4	1.23 ± 2.02	1.22 ± 1.81	0.91 ± 1.78	0.88 ± 1.36	0	0.5	0.5	0	0
miauen	2.34 ± 6.75	2.05 ± 5.6	2.8 ± 9.93	1.34 ± 4.55	1.68 ± 4.6	0	0.0	0.0	0	0
schnurren	1.71 ± 2.38	1.49 ± 2.53	1 ± 1.7	0.78 ± 1.53	1.32 ± 1.84	2	0.0	0.0	0	0

```
### Subset 2

table_mean2<-mosaic::favstats(anzahl ~ zeit + verhalten + umfeld, data=cat_long2)

str(table_mean2)

'data.frame':   60 obs. of  10 variables:
 $ zeit.verhalten.umfeld: chr  "1.fauchen.Private Home" "2.fauchen.Private Home" "3.fauchen.Private Home" "4.fauchen.Private Home" ...
 $ min                  : num  0 0 0 0 0 0 0 0 0 0 ...
 $ Q1                   : num  0 0 0 0 0 0 0 0 0 0 ...
 $ median               : num  0 0 0 0 0 0 0 0 0 0 ...
 $ Q3                   : num  0 0 0 0 0 0 0 0 0 0 ...
 $ max                  : num  0 1 0 1 0 1 1 3 3 3 ...
 $ mean                 : num  0 0.0149 0 0.0299 0 ...
 $ sd                   : num  0 0.122 0 0.171 0 ...
 $ n                    : int  67 67 67 67 67 67 67 67 67 67 ...
 $ missing              : int  0 0 0 0 0 0 0 0 0 0 ...

# Spalte "zeit.verhalten.umfeld" in drei separate Spalten aufteilen und in table_mean1 speichern
table_mean2 <- table_mean2 %>%
  separate(zeit.verhalten.umfeld, into = c("zeit", "verhalten", "umfeld"), sep = "\\.")

# Ergebnis überprüfen
head(table_mean2)

  zeit verhalten       umfeld min Q1 median Q3 max       mean        sd  n
1    1   fauchen Private Home   0  0      0  0   0 0.00000000 0.0000000 67
2    2   fauchen Private Home   0  0      0  0   1 0.01492537 0.1221694 67
3    3   fauchen Private Home   0  0      0  0   0 0.00000000 0.0000000 67
4    4   fauchen Private Home   0  0      0  0   1 0.02985075 0.1714598 67
5    5   fauchen Private Home   0  0      0  0   0 0.00000000 0.0000000 67
6    1   knurren Private Home   0  0      0  0   1 0.01492537 0.1221694 67
  missing
1       0
2       0
3       0
4       0
5       0
6       0

#### Tabelle 1 Chris


# Daten vorbereiten mit Mittelwert ± Standardabweichung und Median (Q1–Q3)
table_summary2 <- table_mean2 %>%
  mutate(
    # Wenn ply in Animal Shelter mit n=11 (Zeit 4), nehme die Werte, aber zeige n=15
    n = ifelse(verhalten == "kontaktini" | verhalten == "kontaktver" & n == 14, 15, n)
  ) %>%
  dplyr::select(zeit, umfeld, verhalten, mean, sd, median, Q1, Q3, n, missing) %>%
  dplyr::mutate(
    mean_sd = paste0(round(mean, 1), " ± ", round(sd, 1)),  # Kombination von Mittelwert und SD
    median_iqr = paste0(round(median, 1), " (", round(Q1, 1), " – ", round(Q3, 1), ")") # Median (Q1–Q3)
  ) %>%
  dplyr::select(zeit, umfeld, verhalten, mean_sd, median_iqr, n)

# Hinzufügen einer Markierung für kontaktini und kontkatkver in Zeit 4
table_summar2 <- table_summary2 %>%
  mutate(
    mean_sd = ifelse(verhalten == "kontaktini" & umfeld == "Animal Shelter" & zeit == 4,
                     paste0(mean_sd, "*"),
                     mean_sd),
    median_iqr = ifelse(verhalten == "kontaktini" & umfeld == "Animal Shelter" & zeit == 4,
                        paste0(median_iqr, "*"),
                        median_iqr),
    mean_sd = ifelse(verhalten == "kontaktver" & umfeld == "Animal Shelter" & zeit == 4,
                     paste0(mean_sd, "*"),
                     mean_sd),
    median_iqr = ifelse(verhalten == "kontaktver" & umfeld == "Animal Shelter" & zeit == 4,
                        paste0(median_iqr, "*"),
                        median_iqr)
  )

table_summary2 <- table_summary2 %>%
  mutate(
    verhalten = case_when(
      verhalten == "kontaktini" ~ "initialisation",
      verhalten == "kontaktver" ~ "avoidance",
      verhalten == "fauchen" ~ "hiss",
      verhalten == "knurren" ~ "growl",
      verhalten == "miauen" ~ "meow",
      verhalten == "schnurren" ~ "purr",
      TRUE ~ verhalten
    )
  )



# Umstrukturieren für den Flex Table mit Zeitpunkten als Spalten
table_wide2 <- table_summary2 %>%
  pivot_wider(
    id_cols = c(umfeld, verhalten, n),
    names_from = zeit,
    values_from = c(mean_sd, median_iqr)
  )

# Spaltenreihenfolge so anpassen, dass mean_sd und median_iqr für jeden Zeitpunkt nebeneinander stehen
table_wide2 <- table_wide2 %>%
  dplyr::select(
    umfeld, verhalten, n,
    mean_sd_1, median_iqr_1,
    mean_sd_2, median_iqr_2,
    mean_sd_3, median_iqr_3,
    mean_sd_4, median_iqr_4,
    mean_sd_5, median_iqr_5
  )

# Erstellen des Flex Tables mit strukturiertem Header für Zeit und Metriken
ft <- flextable(table_wide2) %>%
  # Setzen der Spaltenbeschriftungen
  set_header_labels(
    umfeld = "Living Environment",
    verhalten = "Behaviour",
    n = "n",
    mean_sd_1 = "Mean ± SD",
    median_iqr_1 = "Median (Q1 – Q3)",
    mean_sd_2 = "Mean ± SD",
    median_iqr_2 = "Median (Q1 – Q3)",
    mean_sd_3 = "Mean ± SD",
    median_iqr_3 = "Median (Q1 – Q3)",
    mean_sd_4 = "Mean ± SD",
    median_iqr_4 = "Median (Q1 – Q3)",
    mean_sd_5 = "Mean ± SD",
    median_iqr_5 = "Median (Q1 – Q3)"
  ) %>%
  # Hinzufügen einer Kopfzeile für die Zeitpunkte
  add_header_row(
    values = c("", "", "",
               "Video Sequence 1", "Video Sequence 1",
               "Video Sequence 2", "Video Sequence 2",
               "Video Sequence 3", "Video Sequence 3",
               "Video Sequence 4", "Video Sequence 4",
               "Video Sequence 5", "Video Sequence 5"),
    colwidths = c(1, 1, 1, 1, 1, 1, 1, 1, 1, 1, 1, 1, 1)
  ) %>%
  # Zusammenführen der Header-Zellen für Zeit
  merge_h_range(i = 1, j1 = 4, j2 = 5, part = "header") %>%
  merge_h_range(i = 1, j1 = 6, j2 = 7, part = "header") %>%
  merge_h_range(i = 1, j1 = 8, j2 = 9, part = "header") %>%
  merge_h_range(i = 1, j1 = 10, j2 = 11, part = "header") %>%
  merge_h_range(i = 1, j1 = 12, j2 = 13, part = "header") %>%
  # Manuelles Zusammenführen der Umfeld-Zellen
  merge_v(j = "umfeld") %>%
  # Formatierung
  theme_vanilla() %>%
  align(align = "center", part = "header") %>%
  align(j = c("umfeld", "verhalten"), align = "left", part = "body") %>%
  align(j = "n", align = "center", part = "body") %>%
  vline(j = c(3, 5, 7, 9, 11), part = "all") %>%
  border_outer() %>%
  autofit()

# Hinzufügen einer Fußnote
ft <- ft %>%
  add_footer_lines("* for initialisation in video sequence 4, the actual sample size is n=14") %>%fontsize(part = "footer", size = 9) %>%
  italic(part = "footer") %>%
  align(part = "footer", align = "left")

# Anzeigen der Tabelle
ft
```

			Video Sequence 1		Video Sequence 2		Video Sequence 3		Video Sequence 4		Video Sequence 5	
Living Environment	Behaviour	n	Mean ± SD	Median (Q1 - Q3)	Mean ± SD	Median (Q1 - Q3)	Mean ± SD	Median (Q1 - Q3)	Mean ± SD	Median (Q1 - Q3)	Mean ± SD	Median (Q1 - Q3)
Private Home	hiss	67	0 ± 0	0 (0 - 0)	0 ± 0.1	0 (0 - 0)	0 ± 0	0 (0 - 0)	0 ± 0.2	0 (0 - 0)	0 ± 0	0 (0 - 0)
	growl	67	0 ± 0.1	0 (0 - 0)	0 ± 0.1	0 (0 - 0)	0 ± 0.4	0 (0 - 0)	0 ± 0.4	0 (0 - 0)	0 ± 0.4	0 (0 - 0)
	initialisation	15	7.2 ± 6	6 (4 - 10)	6.4 ± 5.3	6 (4 - 8)	5 ± 4.7	4 (2 - 6)	3.6 ± 4.1	2 (0 - 6)	4.4 ± 5.5	4 (0 - 6)
	avoidance	67	1 ± 1.6	0 (0 - 2)	1.1 ± 1.5	1 (0 - 2)	1 ± 1.3	1 (0 - 2)	0.9 ± 1.2	0 (0 - 2)	0.7 ± 1.1	0 (0 - 1)
	meow	67	2.5 ± 7.3	0 (0 - 2)	2.2 ± 5.8	0 (0 - 2)	2.8 ± 9.7	0 (0 - 0)	1.3 ± 4.7	0 (0 - 0)	1.9 ± 5	0 (0 - 0)
	purr	67	1.2 ± 1.6	0 (0 - 2)	1 ± 1.4	0 (0 - 2)	0.8 ± 1.4	0 (0 - 2)	0.6 ± 1.2	0 (0 - 1)	1.1 ± 1.6	0 (0 - 2)
Animal Shelter	hiss	15	0.2 ± 0.8	0 (0 - 0)	0.1 ± 0.3	0 (0 - 0)	0 ± 0	0 (0 - 0)	0.1 ± 0.3	0 (0 - 0)	0 ± 0	0 (0 - 0)
	growl	15	0 ± 0	0 (0 - 0)	0.1 ± 0.3	0 (0 - 0)	0.1 ± 0.3	0 (0 - 0)	0 ± 0	0 (0 - 0)	0 ± 0	0 (0 - 0)
	initialisation	15	13.3 ± 11.2	10 (4 - 24)	11.9 ± 12	12 (0 - 21)	7.2 ± 8.9	2 (0 - 13)	4.4 ± 7.1	1 (0 - 3.5)	7.5 ± 7.9	6 (1 - 11)
	avoidance	15	2.7 ± 4.3	0 (0 - 4)	1.6 ± 3.6	0 (0 - 1)	2 ± 3.1	0 (0 - 2.5)	1.1 ± 3.4	0 (0 - 0.8)	1.5 ± 2.2	1 (0 - 2.5)
	meow	15	1.5 ± 3.6	0 (0 - 0)	1.3 ± 4.6	0 (0 - 0)	2.9 ± 11.4	0 (0 - 0)	1.3 ± 4.1	0 (0 - 0)	0.5 ± 1.6	0 (0 - 0)
	purr	15	4.1 ± 3.7	4 (1 - 6)	3.9 ± 4.5	2 (0 - 7)	1.9 ± 2.7	2 (0 - 2)	1.6 ± 2.5	0 (0 - 2)	2.3 ± 2.5	2 (0 - 4)

** for initialisation in video sequence 4, the actual sample size is n=14*

Grafiken

Subset 1

```
cat_long %>% dplyr::group_by(verhalten, zeit) %>%
            summarize(sec=mean(sec,na.rm=T)) %>%
ggplot(aes(y=sec, x=zeit, color=reorder(verhalten,-sec), group=reorder(verhalten,-sec))) +
  geom_point() +
  geom_line() +
  scale_y_continuous(limits=c(0,100),breaks=seq(0,100,10)) +
  scale_color_brewer(labels=c( "Exploration", "Passive","Phy. Contact", "Play", "Door" ), palette = "Paired") +
  labs(x="Video Sequence (No)", y = "Behaviour (sec)",color="Behaviour Categories",shape="Behvaiour Categories") +
  theme_bw()



#x="Video Sequence (No)", y = "Behaviour (sec)",color="Living Environment",shape="Living Environment"

# Darstellung getrennt nach Umfeld

new_labels_lmm1 <- c(
  "exp" = "Exploration",
  "con" = "Phy. Contact",
  "passiv" = "Passive",
  "ply" = "Play",
  "tuer" = "Door"
)


cat_long %>% dplyr::group_by(verhalten, zeit, umfeld) %>%
            summarize(sec=mean(sec,na.rm=T)) %>%
ggplot(aes(y=sec, x=zeit, color=reorder(verhalten,-sec), group=reorder(verhalten,-sec))) +
  geom_point() +
  geom_line() +
  scale_color_grey(start = 0.2, end=0.9) +
  scale_y_continuous(limits=c(0,100),breaks=seq(0,100,10)) +
  scale_color_manual(values = gray.colors(5), labels = new_labels_lmm1) +
    labs(x="Video Sequence (No)", y = "Behaviour (sec)",color="Behaviour Categories",shape="Behvaiour Categories") +
  theme_bw() +
  facet_wrap(~umfeld)



#scale_color_brewer(labels=c("Exploration","Play", "Passive", "Door", "Phy. Contact"), palette = "Paired") +
#scale_color_grey(start = 0.2, end=0.9) +

# Darstellung getrennt nach Freigang




cat_long %>% dplyr::group_by(verhalten, zeit, freigang) %>%
            summarize(sec=mean(sec,na.rm=T)) %>%
ggplot(aes(y=sec, x=zeit, color=verhalten, group=verhalten)) +
  geom_point() +
  geom_line() +
  scale_y_continuous(limits=c(0,100),breaks=seq(0,100,10)) +
  scale_color_grey(start = 0.3, end=0.8) +
  labs(x="Video Sequence (No)", y = "Behaviour (sec)",color="Behaviour Categories",shape="Behvaiour Categories") +
  theme_bw() +
  facet_wrap(~freigang)



# scale_color_brewer(labels=c("Exploration","Play", "Passive", "Door", "Phy. Contact"), palette = "Paired") +
```

Subset 2

```
cat_long2 %>% dplyr::group_by(verhalten, zeit) %>%
            summarize(anzahl=mean(anzahl,na.rm=T)) %>%
ggplot(aes(y=anzahl, x=zeit, color=reorder(verhalten,-anzahl), group=reorder(verhalten,-anzahl))) +
  geom_point() +
  geom_line() +
  scale_y_continuous(limits=c(0,10),breaks=seq(0,10,2)) +
  scale_color_brewer(labels=c("Initialisation","Meow", "Purr", "Avoidance", "Growl", "Hiss"), palette = "Paired") +
  labs(x="Video Sequence (No)", y = "Behaviour (number)",color="Behaviour Categories",shape="Behvaiour Categories") +
  theme_bw()
```

str(cat

```
new_labels_glmm <- c(
  "fauchen" = "Hiss",
  "knurren" = "Growl",
  "miauen" = "Meow",
  "schnurren" = "Purr",
  "kontaktver" = "Avoidance",
  "kontaktini" = "Initialisation"
)



cat_long2 %>% dplyr::group_by(verhalten, zeit, umfeld) %>%
            summarize(anzahl=mean(anzahl,na.rm=T)) %>%
ggplot(aes(y=anzahl, x=zeit, color=reorder(verhalten,-anzahl), group=reorder(verhalten,-anzahl))) +
  geom_point() +
  geom_line() +
    #scale_color_grey(start = 0.2, end=0.9) +
    scale_y_continuous(limits=c(0,15),breaks=seq(0,15,1)) +
    scale_color_manual(values = gray.colors(6), labels = new_labels_glmm) +  # Neue Labels hier
    labs(x = "Video Sequence (No)", y = "Behaviour (number)", color = "Behaviour Categories",      shape = "Behaviour Categories") +
    scale_y_continuous(limits=c(0,16),breaks=seq(0,16,2)) +
    labs(x="Video Sequence (No)", y = "Behaviour (number)",color="Behaviour Categories",shape="Behvaiour Categories") +
  theme_bw() +
  facet_wrap(~umfeld)



#scale_color_brewer(labels=c("Initialisation","Meow", "Purr", "Avoidance", "Hiss","Growl"), palette = "Paired") +
#scale_color_grey(start = 0.3, end=0.8)

# Darstellung getrennt nach Freigang

cat_long2 %>% dplyr::group_by(verhalten, zeit, freigang) %>%
            summarize(anzahl=mean(anzahl,na.rm=T)) %>%
ggplot(aes(y=anzahl, x=zeit, color=verhalten, group=verhalten)) +
  geom_point() +
  geom_line() +
  scale_y_continuous(limits=c(0,16),breaks=seq(0,16,2)) +
  scale_color_brewer(labels=c("Initialisation","Meow", "Purr", "Avoidance", "Growl", "Hiss"), palette = "Paired") +
  labs(x="Video Sequence (No)", y = "Behaviour (number)",color="Behaviour Categories",shape="Behvaiour Categories") +
  theme_bw() +
  facet_wrap(~freigang)
```

Katzen einzeln

```
#Exploration

cat_long$versuch <- factor(cat_long$versuch)

cats_exp1 <- cat_long %>% dplyr::filter(verhalten=="exp") %>%
             ggplot(aes(y=sec, x=zeit, color=versuch, group=versuch)) +
              geom_line(aes(color=versuch)) +
              scale_color_grey(start=0.6, end=0.7) +
              labs(y="Exploration Behaviour (sec)", x="Video Sequence") +
              theme_bw() +
              facet_wrap(~umfeld) +
              theme(axis.text.x = element_text(angle = 45, hjust=1, size=8),
                    legend.position = "none")+
              scale_x_discrete(labels=c("1", "2", "3","4", "5"))

cats_exp1



cats_exp2 <- cat_long %>% dplyr::filter(verhalten=="exp") %>%
             ggplot(aes(y=sec, x=zeit, color=versuch, group=versuch)) +
              geom_line(aes(color=versuch)) +
              scale_color_grey(start=0.6, end=0.7) +
              labs(y="Exploration Behaviour (sec)", x="Video Sequence") +
              theme_bw() +
              facet_grid(rasse~umfeld) +
              theme(axis.text.x = element_text(angle = 45, hjust=1, size=8),
                    legend.position = "none")+
              scale_x_discrete(labels=c("1", "2", "3","4", "5"))

cats_exp2



cats_exp3 <- cat_long %>% dplyr::filter(verhalten=="exp") %>%
             ggplot(aes(y=sec, x=zeit, color=versuch, group=versuch)) +
              geom_line(aes(color=versuch)) +
              scale_color_grey(start=0.6, end=0.7) +
              labs(y="Exploration Behaviour (sec)", x="Video Sequence") +
              theme_bw() +
              facet_grid(geschlecht_katze~umfeld) +
              theme(axis.text.x = element_text(angle = 45, hjust=1, size=8),
                    legend.position = "none")+
              scale_x_discrete(labels=c("1", "2", "3","4", "5"))

cats_exp3



cats_exp4 <- cat_long %>% dplyr::filter(verhalten=="exp") %>%
             ggplot(aes(y=sec, x=zeit, color=versuch, group=versuch)) +
              geom_line(aes(color=versuch)) +
              scale_color_grey(start=0.6, end=0.7) +
              labs(y="Exploration Behaviour (sec)", x="Video Sequence") +
              theme_bw() +
              facet_grid(geschlecht_katze~freigang) +
              theme(axis.text.x = element_text(angle = 45, hjust=1, size=8),
                    legend.position = "none")+
              scale_x_discrete(labels=c("1", "2", "3","4", "5"))

cats_exp4



#Physical Contact
cat_long$versuch <- factor(cat_long$versuch)

cats_con1 <- cat_long %>% dplyr::filter(verhalten=="con") %>%
             ggplot(aes(y=sec, x=zeit, color=versuch, group=versuch)) +
              geom_line(aes(color=versuch)) +
              scale_color_grey(start=0.6, end=0.7) +
              labs(y="Physical Contact  (sec)", x="Video Sequence") +
              theme_bw() +
              facet_wrap(~umfeld) +
              theme(axis.text.x = element_text(angle = 45, hjust=1, size=8),
                    legend.position = "none")+
              scale_x_discrete(labels=c("1", "2", "3","4", "5"))

cats_con1



cats_con2 <- cat_long %>% dplyr::filter(verhalten=="con") %>%
             ggplot(aes(y=sec, x=zeit, color=versuch, group=versuch)) +
              geom_line(aes(color=versuch)) +
              scale_color_grey(start=0.6, end=0.7) +
              labs(y="Physical Contact (sec)", x="Video Sequence") +
              theme_bw() +
              facet_grid(rasse~umfeld) +
              theme(axis.text.x = element_text(angle = 45, hjust=1, size=8),
                    legend.position = "none")+
              scale_x_discrete(labels=c("1", "2", "3","4", "5"))

cats_con2



cats_con3 <- cat_long %>% dplyr::filter(verhalten=="con") %>%
             ggplot(aes(y=sec, x=zeit, color=versuch, group=versuch)) +
              geom_line(aes(color=versuch)) +
              scale_color_grey(start=0.6, end=0.7) +
              labs(y="Physical Contact  (sec)", x="Video Sequence") +
              theme_bw() +
              facet_grid(geschlecht_katze~umfeld) +
              theme(axis.text.x = element_text(angle = 45, hjust=1, size=8),
                    legend.position = "none")+
              scale_x_discrete(labels=c("1", "2", "3","4", "5"))

cats_con3



cats_con4 <- cat_long %>% dplyr::filter(verhalten=="con") %>%
             ggplot(aes(y=sec, x=zeit, color=versuch, group=versuch)) +
              geom_line(aes(color=versuch)) +
              scale_color_grey(start=0.6, end=0.7) +
              labs(y="Physical Contact  (sec)", x="Video Sequence") +
              theme_bw() +
              facet_grid(geschlecht_katze~freigang) +
              theme(axis.text.x = element_text(angle = 45, hjust=1, size=8),
                    legend.position = "none")+
              scale_x_discrete(labels=c("1", "2", "3","4", "5"))

cats_con4



#Play
cat_long$versuch <- factor(cat_long$versuch)

cats_ply1 <- cat_long %>% dplyr::filter(verhalten=="ply") %>%
             ggplot(aes(y=sec, x=zeit, color=versuch, group=versuch)) +
              geom_line(aes(color=versuch)) +
              scale_color_grey(start=0.6, end=0.7) +
              labs(y="Exploration Behaviour (sec)", x="Video Sequence") +
              theme_bw() +
              facet_wrap(~umfeld) +
              theme(axis.text.x = element_text(angle = 45, hjust=1, size=8),
                    legend.position = "none")+
              scale_x_discrete(labels=c("1", "2", "3","4", "5"))
```

```
cats_ply1
```

```
cats_ply2 <- cat_long %>% dplyr::filter(verhalten=="ply") %>%
             ggplot(aes(y=sec, x=zeit, color=versuch, group=versuch)) +
              geom_line(aes(color=versuch)) +
              scale_color_grey(start=0.6, end=0.7) +
              labs(y="Play Behaviour (sec)", x="Video Sequence") +
              theme_bw() +
              facet_grid(rasse~umfeld) +
              theme(axis.text.x = element_text(angle = 45, hjust=1, size=8),
                    legend.position = "none")+
              scale_x_discrete(labels=c("1", "2", "3","4", "5"))

cats_ply2
```

```
cats_ply3 <- cat_long %>% dplyr::filter(verhalten=="ply") %>%
             ggplot(aes(y=sec, x=zeit, color=versuch, group=versuch)) +
              geom_line(aes(color=versuch)) +
              scale_color_grey(start=0.6, end=0.7) +
              labs(y="Play Behaviour (sec)", x="Video Sequence") +
              theme_bw() +
              facet_grid(geschlecht_katze~umfeld) +
              theme(axis.text.x = element_text(angle = 45, hjust=1, size=8),
                    legend.position = "none")+
              scale_x_discrete(labels=c("1", "2", "3","4", "5"))

cats_ply3
```

```
cats_ply4 <- cat_long %>% dplyr::filter(verhalten=="ply") %>%
             ggplot(aes(y=sec, x=zeit, color=versuch, group=versuch)) +
              geom_line(aes(color=versuch)) +
              scale_color_grey(start=0.6, end=0.7) +
              labs(y="Play Behaviour (sec)", x="Video Sequence") +
              theme_bw() +
              facet_grid(geschlecht_katze~freigang) +
              theme(axis.text.x = element_text(angle = 45, hjust=1, size=8),
                    legend.position = "none")+
              scale_x_discrete(labels=c("1", "2", "3","4", "5"))

cats_ply4
```

Verhaltensweisen

```
cat_long <- cat_long %>%
  # Group by versuch and zeit to calculate proportions within each time point
  group_by(versuch, zeit) %>%
  # Calculate sum for each group (excluding NA)
  mutate(
    total_sec = sum(sec, na.rm = TRUE),
    # Calculate percentage, handling NAs
    percentage = ifelse(!is.na(sec), (sec/total_sec) * 100, NA)
  ) %>%
  ungroup()

# Let's check the results
# First, verify that percentages sum to 100 within each group
validation <- cat_long %>%
  group_by(versuch, zeit) %>%
  summarise(
    total_percentage = sum(percentage, na.rm = TRUE),
    .groups = 'drop'
  ) %>%
  # Round to handle floating point precision
  mutate(total_percentage = round(total_percentage, 1))

# Look at some example results
print("Example of relative frequencies:")

[1] "Example of relative frequencies:"

cat_long %>% dplyr::select(versuch, zeit, verhalten, sec, percentage) %>%
  head(10)

# A tibble: 10 × 5
   versuch zeit  verhalten   sec percentage
   <fct>   <fct> <fct>     <dbl>      <dbl>
 1 1       1     exp         118     62.1
 2 1       1     ply           0      0
 3 1       1     passiv       48     25.3
 4 1       1     tuer         23     12.1
 5 1       1     con           1      0.526
 6 1       2     exp         174     81.7
 7 1       2     ply           3      1.41
 8 1       2     passiv        3      1.41
 9 1       2     tuer         33     15.5
10 1       2     con           0      0

print("\nValidation of total percentages (should be close to 100):")

[1] "\nValidation of total percentages (should be close to 100):"

print(head(validation))

# A tibble: 6 × 3
  versuch zeit  total_percentage
  <fct>   <fct>            <dbl>
1 1       1                  100
2 1       2                  100
3 1       3                  100
4 1       4                  100
5 1       5                  100
6 2       1                  100

ggplot(cat_long, aes(x = zeit, y = percentage, fill = verhalten)) +
  geom_bar(stat = "identity", position = "stack") +
  # Add labels and title
  labs(
    x = "Video Sequence",
    y = "Percentage",
    fill = "Behaviour",
    title = "Distribution of Behaviours Across Video Sequences"
  ) +
  # Use a clean theme
  theme_minimal() +
  # Make text more readable
  theme(
    axis.text = element_text(size = 12),
    axis.title = element_text(size = 14),
    legend.title = element_text(size = 12),
    legend.text = element_text(size = 10)
  )
```

```
# Calculate mean percentages for each behavior to determine order
behavior_order <- cat_long %>%
  group_by(verhalten) %>%
  summarize(mean_pct = mean(percentage, na.rm = TRUE)) %>%
  arrange(mean_pct) %>%
  pull(verhalten)

library(ggplot2)

# Calculate mean percentages for each behavior to determine order
behavior_order <- cat_long %>%
  group_by(verhalten) %>%
  summarize(mean_pct = mean(percentage, na.rm = TRUE)) %>%
  arrange(mean_pct) %>%
  pull(verhalten)

# Prepare data with middle positions for labels
label_data <- cat_long %>%
  group_by(zeit) %>%
  arrange(match(verhalten, behavior_order)) %>%
  mutate(
    # Calculate the position for labels (middle of each segment)
    label_y = cumsum(percentage) - (percentage/2),
    # Round percentage to 1 decimal
    percentage_label = round(percentage, 1)
  )

# Create the plot
ggplot(cat_long, aes(x = zeit, y = percentage, fill = factor(verhalten, levels = behavior_order))) +
  geom_bar(stat = "identity", position = "stack") +
  # Add text labels at the middle of each segment
  geom_text(data = label_data,
            aes(y = label_y, label = sprintf("%.1f%%", percentage_label)),
            size = 3)
```

```
  # Labels and title
  labs(
     x = "Zeit",
     y = "Percentage",
     fill = "Verhalten",
     title = "Distribution of Behaviors Across Time Points"
   ) +
   theme_minimal() +
  theme(
    axis.text = element_text(size = 12),
    axis.title = element_text(size = 14),
    legend.title = element_text(size = 12),
    legend.text = element_text(size = 10)
  )

NULL

# Neue Labels für Verhalten
verhalten_labels <- c(
  "tuer"   = "door",
  "ply"    = "play",
  "con"    = "phys. contact",
  "passiv" = "passive",
  "exp"    = "exploration"
)

# Plot mit umbenannten Achsen- und Legendenbeschriftungen
ggplot(cat_long, aes(x = zeit, y = percentage, fill = verhalten)) +
  geom_bar(stat = "identity", position = "stack") +
  labs(
    x = "Video Sequence",
    y = "Percentage",
    fill = "Behaviour",
    title = "Distribution of Behaviours Across Time Points"
  ) +
  scale_fill_manual(
    values = RColorBrewer::brewer.pal(length(unique(cat_long$verhalten)), "Set2"),
    labels = verhalten_labels
  ) +
  theme_minimal() +
  theme(
    axis.text = element_text(size = 12),
    axis.title = element_text(size = 14),
    legend.title = element_text(size = 12),
    legend.text = element_text(size = 10)
  )
```

Inferenzielle Analyse -Subset 1

5 Verhaltensweisen, 5 Videosequenzen, 2 Umfelder

Exploration (exp)

Modelle Umfeld + Zeit

```
# ANOVA rechnen für Grafik

exp_m1 <- aov_car(sec ~ umfeld *zeit+ Error(versuch/zeit),
                  data=cat_long %>% dplyr::filter(verhalten=="exp"),
                  type=3)
summary(exp_m1)


Univariate Type III Repeated-Measures ANOVA Assuming Sphericity

             Sum Sq num Df Error SS den Df  F value  Pr(>F)
(Intercept) 1394967      1   760984     80 146.6487 < 2e-16 ***
umfeld        36177      1   760984     80   3.8032 0.05466 .
zeit           1632      4   307863    320   0.4240 0.79128
umfeld:zeit    5948      4   307863    320   1.5456 0.18876
---
Signif. codes:  0 '***' 0.001 '**' 0.01 '*' 0.05 '.' 0.1 ' ' 1


Mauchly Tests for Sphericity

            Test statistic p-value
zeit               0.88686 0.39995
umfeld:zeit        0.88686 0.39995


Greenhouse-Geisser and Huynh-Feldt Corrections
 for Departure from Sphericity

             GG eps Pr(>F[GG])
zeit        0.95292     0.7822
umfeld:zeit 0.95292     0.1915

              HF eps Pr(>F[HF])
zeit        1.006541  0.7912849
umfeld:zeit 1.006541  0.1887574

effectsize::omega_squared(exp_m1)

# Effect Size for ANOVA (Type III)

Parameter   | Omega2 (partial) |       95% CI
---------------------------------------------
umfeld      |             0.03 | [0.00, 1.00]
zeit        |             0.00 | [0.00, 1.00]
umfeld:zeit |         1.94e-03 | [0.00, 1.00]

- One-sided CIs: upper bound fixed at [1.00].

# LMM rechnen

exp_lmm <- lmer(exp ~ umfeld*zeit + (1|versuch), data = cat_l)

summary(exp_lmm)

Linear mixed model fit by REML. t-tests use Satterthwaite's method [
lmerModLmerTest]
Formula: exp ~ umfeld * zeit + (1 | versuch)
   Data: cat_l

REML criterion at convergence: 4100.6

Scaled residuals:
     Min       1Q   Median       3Q      Max
-2.84212 -0.52497 -0.08042  0.61511  2.61605

Random effects:
 Groups   Name        Variance Std.Dev.
 versuch  (Intercept) 1710.0   41.35
 Residual              962.1   31.02
Number of obs: 410, groups:  versuch, 82

Fixed effects:
                           Estimate Std. Error      df t value Pr(>|t|)
(Intercept)                  69.955      6.315 151.619  11.077   <2e-16 ***
umfeldAnimal Shelter         17.245     14.766 151.619   1.168   0.2447
zeit2                         2.433      5.359 320.000   0.454   0.6502
zeit3                       -12.388      5.359 320.000  -2.312   0.0214 *
zeit4                       -13.881      5.359 320.000  -2.590   0.0100 *
zeit5                        -9.493      5.359 320.000  -1.771   0.0775 .
umfeldAnimal Shelter:zeit2   -8.700     12.530 320.000  -0.694   0.4880
umfeldAnimal Shelter:zeit3   17.521     12.530 320.000   1.398   0.1630
umfeldAnimal Shelter:zeit4   12.281     12.530 320.000   0.980   0.3278
umfeldAnimal Shelter:zeit5   14.159     12.530 320.000   1.130   0.2593
---
Signif. codes:  0 '***' 0.001 '**' 0.01 '*' 0.05 '.' 0.1 ' ' 1

Correlation of Fixed Effects:
            (Intr) umflAS zeit2  zeit3  zeit4  zeit5  umAS:2 umAS:3 umAS:4
umfldAnmlSh -0.428
zeit2       -0.424  0.181
zeit3       -0.424  0.181  0.500
zeit4       -0.424  0.181  0.500  0.500
zeit5       -0.424  0.181  0.500  0.500  0.500
umfldAShl:2  0.181 -0.424 -0.428 -0.214 -0.214 -0.214
umfldAShl:3  0.181 -0.424 -0.214 -0.428 -0.214 -0.214  0.500
umfldAShl:4  0.181 -0.424 -0.214 -0.214 -0.428 -0.214  0.500  0.500
umfldAShl:5  0.181 -0.424 -0.214 -0.214 -0.214 -0.428  0.500  0.500  0.500

anova(exp_lmm)

Type III Analysis of Variance Table with Satterthwaite's method
            Sum Sq Mean Sq NumDF DenDF F value  Pr(>F)
umfeld      3658.9  3658.9     1    80  3.8032 0.05466 .
zeit        1631.7   407.9     4   320  0.4240 0.79128
umfeld:zeit 5947.9  1487.0     4   320  1.5456 0.18876
---
Signif. codes:  0 '***' 0.001 '**' 0.01 '*' 0.05 '.' 0.1 ' ' 1
```

Robust rechnen

bei Verletzung der Homoskedastizitätsvoraussetzung/ Normalverteilung der Residuen

```
# Robust linear mixed model

exp_rlmm <- rlmer(exp ~ umfeld*zeit + (1|versuch), data = cat_l)
summary(exp_rlmm)

Robust linear mixed model fit by DAStau
Formula: exp ~ umfeld * zeit + (1 | versuch)
   Data: cat_l

Scaled residuals:
     Min       1Q   Median       3Q      Max
-3.04403 -0.52410 -0.04899  0.60542  2.72036

Random effects:
 Groups   Name        Variance Std.Dev.
 versuch  (Intercept) 2288.6   47.84
 Residual              852.8   29.20
Number of obs: 410, groups: versuch, 82

Fixed effects:
                           Estimate Std. Error t value
(Intercept)                  70.420      7.023  10.027
umfeldAnimal Shelter         13.779     16.420   0.839
zeit2                         1.214      5.175   0.235
zeit3                       -13.748      5.175  -2.657
zeit4                       -13.483      5.175  -2.605
zeit5                       -10.332      5.175  -1.997
umfeldAnimal Shelter:zeit2   -6.374     12.099  -0.527
umfeldAnimal Shelter:zeit3   20.684     12.099   1.710
umfeldAnimal Shelter:zeit4   13.572     12.099   1.122
umfeldAnimal Shelter:zeit5   18.986     12.099   1.569

Correlation of Fixed Effects:
            (Intr) umflAS zeit2  zeit3  zeit4  zeit5  umAS:2 umAS:3 umAS:4
umfldAnmlSh -0.428
zeit2       -0.368  0.158
zeit3       -0.368  0.158  0.500
zeit4       -0.368  0.158  0.500  0.500
zeit5       -0.368  0.158  0.500  0.500  0.500
umfldAShl:2  0.158 -0.368 -0.428 -0.214 -0.214 -0.214
umfldAShl:3  0.158 -0.368 -0.214 -0.428 -0.214 -0.214  0.500
umfldAShl:4  0.158 -0.368 -0.214 -0.214 -0.428 -0.214  0.500  0.500
umfldAShl:5  0.158 -0.368 -0.214 -0.214 -0.214 -0.428  0.500  0.500  0.500

Robustness weights for the residuals:
 341 weights are ~= 1. The remaining 69 ones are summarized as
   Min. 1st Qu.  Median    Mean 3rd Qu.    Max.
  0.442   0.603   0.837   0.785   0.954   0.999

Robustness weights for the random effects:
 74 weights are ~= 1. The remaining 8 ones are
    3     5     6     8     9    15    38    41
0.989 0.723 0.978 0.929 0.996 0.758 0.817 0.990

Rho functions used for fitting:
  Residuals:
    eff: smoothed Huber (k = 1.345, s = 10)
    sig: smoothed Huber, Proposal 2 (k = 1.345, s = 10)
  Random Effects, variance component 1 (versuch):
    eff: smoothed Huber (k = 1.345, s = 10)
    vcp: smoothed Huber, Proposal 2 (k = 1.345, s = 10)

exp_rlmm2 <- rlmer(exp ~ umfeld*zeit*freigang + (1|versuch), data = cat_l)
summary(exp_rlmm2)

Robust linear mixed model fit by DAStau
Formula: exp ~ umfeld * zeit * freigang + (1 | versuch)
   Data: cat_l

Scaled residuals:
     Min       1Q   Median       3Q      Max
-2.94635 -0.51786 -0.02029  0.55644  2.79022

Random effects:
 Groups   Name        Variance Std.Dev.
 versuch  (Intercept) 2127.7   46.13
 Residual              810.7   28.47
Number of obs: 410, groups: versuch, 82

Fixed effects:
                                         Estimate Std. Error t value
(Intercept)                                50.227     10.150   4.948
umfeldAnimal Shelter                       43.201     18.461   2.340
zeit2                                       2.166      7.540   0.287
zeit3                                      -2.836      7.540  -0.376
zeit4                                      -0.486      7.540  -0.064
zeit5                                       3.503      7.540   0.465
freigangIndoor                             37.099     13.659   2.716
umfeldAnimal Shelter:zeit2                -15.049     13.713  -1.097
umfeldAnimal Shelter:zeit3                  7.067     13.713   0.515
umfeldAnimal Shelter:zeit4                  1.313     13.713   0.096
umfeldAnimal Shelter:zeit5                 -4.274     13.713  -0.312
umfeldAnimal Shelter:freigangIndoor       -91.526     44.383  -2.062
zeit2:freigangIndoor                       -1.664     10.146  -0.164
zeit3:freigangIndoor                      -19.972     10.146  -1.968
zeit4:freigangIndoor                      -23.747     10.146  -2.340
zeit5:freigangIndoor                      -25.591     10.146  -2.522
umfeldAnimal Shelter:zeit2:freigangIndoor  48.048     32.968   1.457
umfeldAnimal Shelter:zeit3:freigangIndoor  38.242     32.968   1.160
umfeldAnimal Shelter:zeit4:freigangIndoor  16.421     32.968   0.498
umfeldAnimal Shelter:zeit5:freigangIndoor  84.863     32.968   2.574

Correlation of Fixed Effects:
            (Intr) umflAS zeit2  zeit3  zeit4  zeit5  frgngI umAS:2 umAS:3
umfldAnmlSh -0.550
zeit2       -0.371  0.204
zeit3       -0.371  0.204  0.500
zeit4       -0.371  0.204  0.500  0.500
zeit5       -0.371  0.204  0.500  0.500  0.500
freigngIndr -0.743  0.409  0.276  0.276  0.276  0.276
umfldAShl:2  0.204 -0.371 -0.550 -0.275 -0.275 -0.275 -0.152
umfldAShl:3  0.204 -0.371 -0.275 -0.550 -0.275 -0.275 -0.152  0.500
umfldAShl:4  0.204 -0.371 -0.275 -0.275 -0.550 -0.275 -0.152  0.500  0.500
umfldAShl:5  0.204 -0.371 -0.275 -0.275 -0.275 -0.550 -0.152  0.500  0.500
umfldAShl:I  0.229 -0.416 -0.085 -0.085 -0.085 -0.085 -0.308  0.154  0.154
zt2:frgngIn  0.276 -0.152 -0.743 -0.372 -0.372 -0.372 -0.371  0.409  0.204
zt3:frgngIn  0.276 -0.152 -0.372 -0.743 -0.372 -0.372 -0.371  0.204  0.409
zt4:frgngIn  0.276 -0.152 -0.372 -0.372 -0.743 -0.372 -0.371  0.204  0.204
zt5:frgngIn  0.276 -0.152 -0.372 -0.372 -0.372 -0.743 -0.371  0.204  0.204
umfldAS:2:I -0.085  0.154  0.229  0.114  0.114  0.114  0.114 -0.416 -0.208
umfldAS:3:I -0.085  0.154  0.114  0.229  0.114  0.114  0.114 -0.208 -0.416
umfldAS:4:I -0.085  0.154  0.114  0.114  0.229  0.114  0.114 -0.208 -0.208
umfldAS:5:I -0.085  0.154  0.114  0.114  0.114  0.229  0.114 -0.208 -0.208
            umAS:4 umAS:5 umAS:I zt2:fI zt3:fI zt4:fI zt5:fI uAS:2: uAS:3:
umfldAnmlSh
zeit2
zeit3
zeit4
zeit5
freigngIndr
umfldAShl:2
umfldAShl:3
umfldAShl:4
umfldAShl:5  0.500
umfldAShl:I  0.154  0.154
zt2:frgngIn  0.204  0.204  0.114
zt3:frgngIn  0.204  0.204  0.114  0.500
zt4:frgngIn  0.409  0.204  0.114  0.500  0.500
zt5:frgngIn  0.204  0.409  0.114  0.500  0.500  0.500
umfldAS:2:I -0.208 -0.208 -0.371 -0.308 -0.154 -0.154 -0.154
umfldAS:3:I -0.208 -0.208 -0.371 -0.154 -0.308 -0.154 -0.154  0.500
umfldAS:4:I -0.416 -0.208 -0.371 -0.154 -0.154 -0.308 -0.154  0.500  0.500
umfldAS:5:I -0.208 -0.416 -0.371 -0.154 -0.154 -0.154 -0.308  0.500  0.500
            uAS:4:
umfldAnmlSh
zeit2
zeit3
zeit4
zeit5
freigngIndr
umfldAShl:2
umfldAShl:3
umfldAShl:4
umfldAShl:5
umfldAShl:I
zt2:frgngIn
zt3:frgngIn
zt4:frgngIn
zt5:frgngIn
umfldAS:2:I
umfldAS:3:I
umfldAS:4:I
umfldAS:5:I  0.500

Robustness weights for the residuals:
 346 weights are ~= 1. The remaining 64 ones are summarized as
   Min. 1st Qu.  Median    Mean 3rd Qu.    Max.
  0.456   0.584   0.797   0.760   0.926   0.998

Robustness weights for the random effects:
 72 weights are ~= 1. The remaining 10 ones are
    5     6     8    15    20    38    63    66    79    82
0.741 0.897 0.842 0.779 0.870 0.893 0.931 0.949 0.929 0.889

Rho functions used for fitting:
  Residuals:
    eff: smoothed Huber (k = 1.345, s = 10)
    sig: smoothed Huber, Proposal 2 (k = 1.345, s = 10)
  Random Effects, variance component 1 (versuch):
    eff: smoothed Huber (k = 1.345, s = 10)
    vcp: smoothed Huber, Proposal 2 (k = 1.345, s = 10)
```

Effektstärke

```
##Cohen's d berechnen


reffects_exp<-broom.mixed::tidy(exp_lmm, effects = c("ran_pars"), scale="vcov")


md_exp <-summary(exp_lmm)$coef[2] #Md = Mittelwertsdifferenz
sdPart_exp<-sqrt(sum(reffects_exp$estimate)) #Random-Effects zusammenzählen
##Cohen´s d für alle Faktoren



cohend_exp <- data.frame(cbind(summary(exp_lmm)$coef[, 1]/sdPart_exp)[c(2,3,4,5,6,7,8,9,10),])
colnames(cohend_exp) <- "CohensD_exp"
```

Grafik

```
#estimated marginal means
# geschätzen Randmittel
# Grafik auf Basis der ANOVA

#emm_exp_m1 <- emmeans(exp_m1, "umfeld", by="zeit")
#emm_exp_m1b <- emmeans(exp_m1, "zeit", by="umfeld")
#em_df_exp_m1<- data.frame(emm_exp_m1)

#ggplot(em_df_exp_m1, aes(x = zeit, y = emmean, group=umfeld, color=umfeld))+
 #geom_line() +
  #geom_errorbar(aes(ymin=lower.CL, ymax=upper.CL), width=.4,
                #position=position_dodge(0.01)) +
 #geom_point(aes(shape=umfeld))+
  #scale_colour_manual(values=c("#D700FF","#00C0FA"))+
  #scale_y_continuous(limits=c(0,120),breaks=seq(0,120,10)) +
  #theme_bw() +
  #labs(x="video sequence", y = "seconds")



em_df_exp_m1<- data.frame(emmeans(exp_lmm, "umfeld", by="zeit"))

exp_g1 <-ggplot(em_df_exp_m1, aes(x = zeit, y = emmean, group=umfeld, color=umfeld))+
  geom_line() +
  geom_errorbar(aes(ymin=lower.CL, ymax=upper.CL), width=.4,
                position=position_dodge(0.01)) +
  geom_point(aes(shape=umfeld))+
  scale_colour_manual(values=c("#D700FF","#00C0FA"))+
  scale_y_continuous(limits=c(0,120),breaks=seq(0,120,10)) +
  theme_bw() +
  labs(x="Video Sequence (No)", y = "Exploration Behaviour (sec)",color="Living Environment",shape="Living Environment")

exp_g1



#"#8CFF00","#F57A00"
```

Paarweise Vergleiche

```
#pairs(emm_exp_m1 , adjust="dunnet")
#pairs(emm_exp_m1b , adjust="dunnet")

#confint(pairs(emmeans(exp_m1,~umfeld)))
#confint(pairs(emmeans(exp_m1,~zeit)))


pairs(emmeans(exp_lmm, "umfeld", by="zeit") , adjust="dunnet")

zeit = 1:
 contrast                      estimate   SE  df t.ratio p.value
 Private Home - Animal Shelter   -17.24 14.8 152  -1.168  0.2447

zeit = 2:
 contrast                      estimate   SE  df t.ratio p.value
 Private Home - Animal Shelter    -8.55 14.8 152  -0.579  0.5636

zeit = 3:
 contrast                      estimate   SE  df t.ratio p.value
 Private Home - Animal Shelter   -34.77 14.8 152  -2.355  0.0198

zeit = 4:
 contrast                      estimate   SE  df t.ratio p.value
 Private Home - Animal Shelter   -29.53 14.8 152  -2.000  0.0473

zeit = 5:
 contrast                      estimate   SE  df t.ratio p.value
 Private Home - Animal Shelter   -31.40 14.8 152  -2.127  0.0351

Degrees-of-freedom method: kenward-roger

pairs(emmeans(exp_lmm, "zeit", by="umfeld") , adjust="dunnet")

umfeld = Private Home:
 contrast      estimate    SE  df t.ratio p.value
 zeit1 - zeit2   -2.433  5.36 320  -0.454  0.9921
 zeit1 - zeit3   12.388  5.36 320   2.312  0.1489
 zeit1 - zeit4   13.881  5.36 320   2.590  0.0767
 zeit1 - zeit5    9.493  5.36 320   1.771  0.4083
 zeit2 - zeit3   14.821  5.36 320   2.766  0.0482
 zeit2 - zeit4   16.313  5.36 320   3.044  0.0215
 zeit2 - zeit5   11.925  5.36 320   2.225  0.1795
 zeit3 - zeit4    1.493  5.36 320   0.279  0.9988
 zeit3 - zeit5   -2.896  5.36 320  -0.540  0.9847
 zeit4 - zeit5   -4.388  5.36 320  -0.819  0.9318

umfeld = Animal Shelter:
 contrast      estimate    SE  df t.ratio p.value
 zeit1 - zeit2    6.267 11.33 320   0.553  0.9833
 zeit1 - zeit3   -5.133 11.33 320  -0.453  0.9921
 zeit1 - zeit4    1.600 11.33 320   0.141  0.9999
 zeit1 - zeit5   -4.667 11.33 320  -0.412  0.9945
 zeit2 - zeit3  -11.400 11.33 320  -1.007  0.8648
 zeit2 - zeit4   -4.667 11.33 320  -0.412  0.9945
 zeit2 - zeit5  -10.933 11.33 320  -0.965  0.8818
 zeit3 - zeit4    6.733 11.33 320   0.595  0.9782
 zeit3 - zeit5    0.467 11.33 320   0.041  1.0000
 zeit4 - zeit5   -6.267 11.33 320  -0.553  0.9833

Degrees-of-freedom method: kenward-roger
P value adjustment: dunnettx method for 10 tests

confint(pairs(emmeans(exp_lmm,~umfeld)))

 contrast                      estimate   SE df lower.CL upper.CL
 Private Home - Animal Shelter    -24.3 12.5 80    -49.1    0.497

Results are averaged over the levels of: zeit
Degrees-of-freedom method: kenward-roger
Confidence level used: 0.95

confint(pairs(emmeans(exp_lmm,~zeit)))

 contrast      estimate   SE  df lower.CL upper.CL
 zeit1 - zeit2    1.917 6.26 320   -15.27     19.1
 zeit1 - zeit3    3.627 6.26 320   -13.56     20.8
 zeit1 - zeit4    7.740 6.26 320    -9.45     24.9
 zeit1 - zeit5    2.413 6.26 320   -14.77     19.6
 zeit2 - zeit3    1.710 6.26 320   -15.48     18.9
 zeit2 - zeit4    5.823 6.26 320   -11.36     23.0
 zeit2 - zeit5    0.496 6.26 320   -16.69     17.7
 zeit3 - zeit4    4.113 6.26 320   -13.07     21.3
 zeit3 - zeit5   -1.214 6.26 320   -18.40     16.0
 zeit4 - zeit5   -5.327 6.26 320   -22.51     11.9

Results are averaged over the levels of: umfeld
Degrees-of-freedom method: kenward-roger
Confidence level used: 0.95
Conf-level adjustment: tukey method for comparing a family of 5 estimates
```

Residuen

```
exp_diagnose <- broom.mixed::augment(exp_lmm)

#Normalverteiung - annähernd normalverteilt

exp_r1 <- ggplot(exp_diagnose, aes(x = .resid)) +

geom_histogram(aes(x = .resid, y = ..density..)) +

geom_density(aes(colour = "density estimate")) +

geom_function(aes(colour = "normal curve"), fun = dnorm,

args = list(mean = mean(exp_diagnose$.resid),sd = sd(exp_diagnose$.resid))) +

scale_colour_discrete(name = "Curve") +

theme(legend.position = "top")


exp_r2 <- ggplot(exp_diagnose, aes(sample = .resid)) +

geom_qq() +

geom_qq_line()

exp_r1 + exp_r2



#DHARMa - prüft automatisch Residuen auf Normalverteilung & Abweichungen

q_unit <- simulateResiduals(exp_lmm)

plot(q_unit)



#Homoskedastizität

exp_r3 <- ggplot(exp_diagnose, aes(x = .fitted, y = .resid)) + geom_point()


exp_r4 <- ggplot(exp_diagnose, aes(x = seq_along(.resid), y = .resid)) +

geom_point() + geom_smooth() + xlab("Index")


exp_r3 + exp_r4



###Ausreißer kontrollieren

#standardisierte Residuen ansehen
##### ID erstezen durch as.numeric usw.

favstats(scale(exp_diagnose$.resid))

       min         Q1      median        Q3      max         mean sd   n
 -3.173268 -0.5861398 -0.08979559 0.6867779 2.920853 9.687373e-17  1 410
 missing
       0

exp_r5 <- ggplot(exp_diagnose, aes(y = .hat, label = as.numeric(rownames(exp_diagnose)))) +

geom_boxplot() +

ggrepel::geom_text_repel(aes(x = 0), max.overlaps = 30)


exp_r6 <- ggplot(exp_diagnose, aes(y = .cooksd)) +

geom_boxplot()


exp_r5 + exp_r6
```

Modelle Umfeld + Zeit + Freigang

```
#ANOVA für Grafik und Paarweise Vergleich

#exp_frei_m1 <- aov_car(sec ~ umfeld *zeit+ Error(versuch/zeit),
#                  data=cat_long %>% dplyr::filter(verhalten=="exp"),
                 # type=3)
#summary(exp_frei_m1)
#effectsize::omega_squared(exp_frei_m1)

cat_l <- cat_long %>%
  pivot_wider(names_from = verhalten, values_from = sec)

exp_lmm_frei <- lmer(exp ~ umfeld*zeit*freigang + (1|versuch), data = cat_l)
summary(exp_lmm_frei)

Linear mixed model fit by REML. t-tests use Satterthwaite's method [
lmerModLmerTest]
Formula: exp ~ umfeld * zeit * freigang + (1 | versuch)
   Data: cat_l

REML criterion at convergence: 4002.4

Scaled residuals:
     Min       1Q   Median       3Q      Max
-2.56625 -0.52453 -0.01621  0.55164  2.44066

Random effects:
 Groups   Name        Variance Std.Dev.
 versuch  (Intercept) 1645.1   40.56
 Residual              930.7   30.51
Number of obs: 410, groups:  versuch, 82

Fixed effects:
                                          Estimate Std. Error       df t value
(Intercept)                                49.4333     9.2660 148.1935   5.335
umfeldAnimal Shelter                       45.1821    16.8521 148.1935   2.681
zeit2                                       4.0000     7.8768 312.0000   0.508
zeit3                                      -0.6667     7.8768 312.0000  -0.085
zeit4                                       0.8000     7.8768 312.0000   0.102
zeit5                                       5.7000     7.8768 312.0000   0.724
freigangIndoor                             37.1613    12.4689 148.1935   2.980
umfeldAnimal Shelter:zeit2                -16.3846    14.3255 312.0000  -1.144
umfeldAnimal Shelter:zeit3                  3.1282    14.3255 312.0000   0.218
umfeldAnimal Shelter:zeit4                 -1.6462    14.3255 312.0000  -0.115
umfeldAnimal Shelter:zeit5                 -9.3154    14.3255 312.0000  -0.650
umfeldAnimal Shelter:freigangIndoor       -92.7766    40.5153 148.1935  -2.290
zeit2:freigangIndoor                       -2.8378    10.5995 312.0000  -0.268
zeit3:freigangIndoor                      -21.2252    10.5995 312.0000  -2.002
zeit4:freigangIndoor                      -26.5838    10.5995 312.0000  -2.508
zeit5:freigangIndoor                      -27.5108    10.5995 312.0000  -2.595
umfeldAnimal Shelter:zeit2:freigangIndoor  48.7225    34.4409 312.0000   1.415
umfeldAnimal Shelter:zeit3:freigangIndoor  41.2637    34.4409 312.0000   1.198
umfeldAnimal Shelter:zeit4:freigangIndoor  20.9299    34.4409 312.0000   0.608
umfeldAnimal Shelter:zeit5:freigangIndoor  89.6262    34.4409 312.0000   2.602
                                          Pr(>|t|)
(Intercept)                               3.51e-07 ***
umfeldAnimal Shelter                       0.00817 **
zeit2                                      0.61194
zeit3                                      0.93260
zeit4                                      0.91917
zeit5                                      0.46982
freigangIndoor                             0.00337 **
umfeldAnimal Shelter:zeit2                 0.25361
umfeldAnimal Shelter:zeit3                 0.82729
umfeldAnimal Shelter:zeit4                 0.90859
umfeldAnimal Shelter:zeit5                 0.51600
umfeldAnimal Shelter:freigangIndoor        0.02344 *
zeit2:freigangIndoor                       0.78908
zeit3:freigangIndoor                       0.04610 *
zeit4:freigangIndoor                       0.01265 *
zeit5:freigangIndoor                       0.00989 **
umfeldAnimal Shelter:zeit2:freigangIndoor  0.15816
umfeldAnimal Shelter:zeit3:freigangIndoor  0.23179
umfeldAnimal Shelter:zeit4:freigangIndoor  0.54382
umfeldAnimal Shelter:zeit5:freigangIndoor  0.00970 **
---
Signif. codes:  0 '***' 0.001 '**' 0.01 '*' 0.05 '.' 0.1 ' ' 1

anova(exp_lmm_frei)

Type III Analysis of Variance Table with Satterthwaite's method
                     Sum Sq Mean Sq NumDF DenDF F value Pr(>F)
umfeld                625.6  625.60     1    78  0.6722 0.4148
zeit                 4704.6 1176.15     4   312  1.2638 0.2842
freigang               73.6   73.63     1    78  0.0791 0.7792
umfeld:zeit          5079.1 1269.78     4   312  1.3644 0.2462
umfeld:freigang      2212.1 2212.10     1    78  2.3769 0.1272
zeit:freigang        5805.8 1451.46     4   312  1.5596 0.1849
umfeld:zeit:freigang 7067.6 1766.89     4   312  1.8986 0.1105
```

Robust rechnen

```
# Robust linear mixed model

exp_frei_rlmm <- rlmer(exp ~ umfeld*zeit*freigang + (1|versuch), data = cat_l)
summary(exp_frei_rlmm)

Robust linear mixed model fit by DAStau
Formula: exp ~ umfeld * zeit * freigang + (1 | versuch)
   Data: cat_l

Scaled residuals:
     Min       1Q   Median       3Q      Max
-2.94635 -0.51786 -0.02029  0.55644  2.79022

Random effects:
 Groups   Name        Variance Std.Dev.
 versuch  (Intercept) 2127.7   46.13
 Residual              810.7   28.47
Number of obs: 410, groups: versuch, 82

Fixed effects:
                                         Estimate Std. Error t value
(Intercept)                                50.227     10.150   4.948
umfeldAnimal Shelter                       43.201     18.461   2.340
zeit2                                       2.166      7.540   0.287
zeit3                                      -2.836      7.540  -0.376
zeit4                                      -0.486      7.540  -0.064
zeit5                                       3.503      7.540   0.465
freigangIndoor                             37.099     13.659   2.716
umfeldAnimal Shelter:zeit2                -15.049     13.713  -1.097
umfeldAnimal Shelter:zeit3                  7.067     13.713   0.515
umfeldAnimal Shelter:zeit4                  1.313     13.713   0.096
umfeldAnimal Shelter:zeit5                 -4.274     13.713  -0.312
umfeldAnimal Shelter:freigangIndoor       -91.526     44.383  -2.062
zeit2:freigangIndoor                       -1.664     10.146  -0.164
zeit3:freigangIndoor                      -19.972     10.146  -1.968
zeit4:freigangIndoor                      -23.747     10.146  -2.340
zeit5:freigangIndoor                      -25.591     10.146  -2.522
umfeldAnimal Shelter:zeit2:freigangIndoor  48.048     32.968   1.457
umfeldAnimal Shelter:zeit3:freigangIndoor  38.242     32.968   1.160
umfeldAnimal Shelter:zeit4:freigangIndoor  16.421     32.968   0.498
umfeldAnimal Shelter:zeit5:freigangIndoor  84.863     32.968   2.574

Correlation of Fixed Effects:
            (Intr) umflAS zeit2  zeit3  zeit4  zeit5  frgngI umAS:2 umAS:3
umfldAnmlSh -0.550
zeit2       -0.371  0.204
zeit3       -0.371  0.204  0.500
zeit4       -0.371  0.204  0.500  0.500
zeit5       -0.371  0.204  0.500  0.500  0.500
freigngIndr -0.743  0.409  0.276  0.276  0.276  0.276
umfldAShl:2  0.204 -0.371 -0.550 -0.275 -0.275 -0.275 -0.152
umfldAShl:3  0.204 -0.371 -0.275 -0.550 -0.275 -0.275 -0.152  0.500
umfldAShl:4  0.204 -0.371 -0.275 -0.275 -0.550 -0.275 -0.152  0.500  0.500
umfldAShl:5  0.204 -0.371 -0.275 -0.275 -0.275 -0.550 -0.152  0.500  0.500
umfldAShl:I  0.229 -0.416 -0.085 -0.085 -0.085 -0.085 -0.308  0.154  0.154
zt2:frgngIn  0.276 -0.152 -0.743 -0.372 -0.372 -0.372 -0.371  0.409  0.204
zt3:frgngIn  0.276 -0.152 -0.372 -0.743 -0.372 -0.372 -0.371  0.204  0.409
zt4:frgngIn  0.276 -0.152 -0.372 -0.372 -0.743 -0.372 -0.371  0.204  0.204
zt5:frgngIn  0.276 -0.152 -0.372 -0.372 -0.372 -0.743 -0.371  0.204  0.204
umfldAS:2:I -0.085  0.154  0.229  0.114  0.114  0.114  0.114 -0.416 -0.208
umfldAS:3:I -0.085  0.154  0.114  0.229  0.114  0.114  0.114 -0.208 -0.416
umfldAS:4:I -0.085  0.154  0.114  0.114  0.229  0.114  0.114 -0.208 -0.208
umfldAS:5:I -0.085  0.154  0.114  0.114  0.114  0.229  0.114 -0.208 -0.208
            umAS:4 umAS:5 umAS:I zt2:fI zt3:fI zt4:fI zt5:fI uAS:2: uAS:3:
umfldAnmlSh
zeit2
zeit3
zeit4
```

```
zeit5
freigngIndr
umfldAShl:2
umfldAShl:3
umfldAShl:4
umfldAShl:5  0.500
umfldAShl:I  0.154  0.154
zt2:frgngIn  0.204  0.204  0.114
zt3:frgngIn  0.204  0.204  0.114  0.500
zt4:frgngIn  0.409  0.204  0.114  0.500  0.500
zt5:frgngIn  0.204  0.409  0.114  0.500  0.500  0.500
umfldAS:2:I -0.208 -0.208 -0.371 -0.308 -0.154 -0.154 -0.154
umfldAS:3:I -0.208 -0.208 -0.371 -0.154 -0.308 -0.154 -0.154  0.500
umfldAS:4:I -0.416 -0.208 -0.371 -0.154 -0.154 -0.308 -0.154  0.500  0.500
umfldAS:5:I -0.208 -0.416 -0.371 -0.154 -0.154 -0.154 -0.308  0.500  0.500
            uAS:4:
umfldAnmlSh
zeit2
zeit3
zeit4
zeit5
freigngIndr
umfldAShl:2
umfldAShl:3
umfldAShl:4
umfldAShl:5
umfldAShl:I
zt2:frgngIn
zt3:frgngIn
zt4:frgngIn
zt5:frgngIn
umfldAS:2:I
umfldAS:3:I
umfldAS:4:I
umfldAS:5:I  0.500

Robustness weights for the residuals:
 346 weights are ~= 1. The remaining 64 ones are summarized as
   Min. 1st Qu.  Median    Mean 3rd Qu.    Max.
  0.456   0.584   0.797   0.760   0.926   0.998

Robustness weights for the random effects:
 72 weights are ~= 1. The remaining 10 ones are
    5     6     8    15    20    38    63    66    79    82
0.741 0.897 0.842 0.779 0.870 0.893 0.931 0.949 0.929 0.889

Rho functions used for fitting:
  Residuals:
    eff: smoothed Huber (k = 1.345, s = 10)
    sig: smoothed Huber, Proposal 2 (k = 1.345, s = 10)
  Random Effects, variance component 1 (versuch):
    eff: smoothed Huber (k = 1.345, s = 10)
    vcp: smoothed Huber, Proposal 2 (k = 1.345, s = 10)
```

```
exp_frei_rlmm2 <- rlmer(exp ~ umfeld*zeit*freigang + (1|versuch), data = cat_l)
summary(exp_frei_rlmm2)
```

```
Robust linear mixed model fit by DAStau
Formula: exp ~ umfeld * zeit * freigang + (1 | versuch)
   Data: cat_l

Scaled residuals:
     Min       1Q   Median       3Q      Max
-2.94635 -0.51786 -0.02029  0.55644  2.79022

Random effects:
 Groups   Name        Variance Std.Dev.
 versuch  (Intercept) 2127.7   46.13
 Residual              810.7   28.47
Number of obs: 410, groups: versuch, 82

Fixed effects:
                                          Estimate Std. Error t value
(Intercept)                                 50.227     10.150   4.948
umfeldAnimal Shelter                        43.201     18.461   2.340
zeit2                                        2.166      7.540   0.287
zeit3                                       -2.836      7.540  -0.376
zeit4                                       -0.486      7.540  -0.064
zeit5                                        3.503      7.540   0.465
freigangIndoor                              37.099     13.659   2.716
umfeldAnimal Shelter:zeit2                 -15.049     13.713  -1.097
umfeldAnimal Shelter:zeit3                   7.067     13.713   0.515
umfeldAnimal Shelter:zeit4                   1.313     13.713   0.096
umfeldAnimal Shelter:zeit5                  -4.274     13.713  -0.312
umfeldAnimal Shelter:freigangIndoor        -91.526     44.383  -2.062
zeit2:freigangIndoor                        -1.664     10.146  -0.164
zeit3:freigangIndoor                       -19.972     10.146  -1.968
zeit4:freigangIndoor                       -23.747     10.146  -2.340
zeit5:freigangIndoor                       -25.591     10.146  -2.522
umfeldAnimal Shelter:zeit2:freigangIndoor   48.048     32.968   1.457
umfeldAnimal Shelter:zeit3:freigangIndoor   38.242     32.968   1.160
umfeldAnimal Shelter:zeit4:freigangIndoor   16.421     32.968   0.498
umfeldAnimal Shelter:zeit5:freigangIndoor   84.863     32.968   2.574

Correlation of Fixed Effects:
            (Intr) umflAS zeit2  zeit3  zeit4  zeit5  frgngI umAS:2 umAS:3
umfldAnmlSh -0.550
zeit2       -0.371  0.204
zeit3       -0.371  0.204  0.500
zeit4       -0.371  0.204  0.500  0.500
zeit5       -0.371  0.204  0.500  0.500  0.500
freigngIndr -0.743  0.409  0.276  0.276  0.276  0.276
umfldAShl:2  0.204 -0.371 -0.550 -0.275 -0.275 -0.275 -0.152
umfldAShl:3  0.204 -0.371 -0.275 -0.550 -0.275 -0.275 -0.152  0.500
umfldAShl:4  0.204 -0.371 -0.275 -0.275 -0.550 -0.275 -0.152  0.500  0.500
umfldAShl:5  0.204 -0.371 -0.275 -0.275 -0.275 -0.550 -0.152  0.500  0.500
umfldAShl:I  0.229 -0.416 -0.085 -0.085 -0.085 -0.085 -0.308  0.154  0.154
zt2:frgngIn  0.276 -0.152 -0.743 -0.372 -0.372 -0.372 -0.371  0.409  0.204
zt3:frgngIn  0.276 -0.152 -0.372 -0.743 -0.372 -0.372 -0.371  0.204  0.409
zt4:frgngIn  0.276 -0.152 -0.372 -0.372 -0.743 -0.372 -0.371  0.204  0.204
zt5:frgngIn  0.276 -0.152 -0.372 -0.372 -0.372 -0.743 -0.371  0.204  0.204
umfldAS:2:I -0.085  0.154  0.229  0.114  0.114  0.114  0.114 -0.416 -0.208
umfldAS:3:I -0.085  0.154  0.114  0.229  0.114  0.114  0.114 -0.208 -0.416
umfldAS:4:I -0.085  0.154  0.114  0.114  0.229  0.114  0.114 -0.208 -0.208
umfldAS:5:I -0.085  0.154  0.114  0.114  0.114  0.229  0.114 -0.208 -0.208
            umAS:4 umAS:5 umAS:I zt2:fI zt3:fI zt4:fI zt5:fI uAS:2: uAS:3:
umfldAnmlSh
zeit2
zeit3
zeit4
zeit5
freigngIndr
umfldAShl:2
umfldAShl:3
umfldAShl:4
umfldAShl:5  0.500
umfldAShl:I  0.154  0.154
zt2:frgngIn  0.204  0.204  0.114
zt3:frgngIn  0.204  0.204  0.114  0.500
zt4:frgngIn  0.409  0.204  0.114  0.500  0.500
zt5:frgngIn  0.204  0.409  0.114  0.500  0.500  0.500
umfldAS:2:I -0.208 -0.208 -0.371 -0.308 -0.154 -0.154 -0.154
umfldAS:3:I -0.208 -0.208 -0.371 -0.154 -0.308 -0.154 -0.154  0.500
umfldAS:4:I -0.416 -0.208 -0.371 -0.154 -0.154 -0.308 -0.154  0.500  0.500
umfldAS:5:I -0.208 -0.416 -0.371 -0.154 -0.154 -0.154 -0.308  0.500  0.500
            uAS:4:
umfldAnmlSh
zeit2
zeit3
zeit4
zeit5
freigngIndr
umfldAShl:2
umfldAShl:3
umfldAShl:4
umfldAShl:5
umfldAShl:I
zt2:frgngIn
zt3:frgngIn
zt4:frgngIn
zt5:frgngIn
umfldAS:2:I
umfldAS:3:I
umfldAS:4:I
umfldAS:5:I  0.500

Robustness weights for the residuals:
 346 weights are ~= 1. The remaining 64 ones are summarized as
   Min. 1st Qu.  Median    Mean 3rd Qu.    Max.
  0.456   0.584   0.797   0.760   0.926   0.998

Robustness weights for the random effects:
 72 weights are ~= 1. The remaining 10 ones are
    5     6     8    15    20    38    63    66    79    82
0.741 0.897 0.842 0.779 0.870 0.893 0.931 0.949 0.929 0.889

Rho functions used for fitting:
  Residuals:
    eff: smoothed Huber (k = 1.345, s = 10)
    sig: smoothed Huber, Proposal 2 (k = 1.345, s = 10)
  Random Effects, variance component 1 (versuch):
    eff: smoothed Huber (k = 1.345, s = 10)
    vcp: smoothed Huber, Proposal 2 (k = 1.345, s = 10)
```

Effektstärke

```
##Cohen's d berechnen


reffects_exp_frei<-broom.mixed::tidy(exp_lmm_frei, effects = c("ran_pars"), scale="vcov")


md_exp_frei <-summary(exp_lmm_frei)$coef[2] #Md = Mittelwertsdifferenz
sdPart_exp<-sqrt(sum(reffects_exp_frei$estimate)) #Random-Effects zusammenzählen
##Cohen´s d für alle Faktoren



cohend_exp_frei <- data.frame(cbind(summary(exp_lmm_frei)$coef[, 1]/sdPart_exp)[c(2,3,4,5,6,7,8,9,10),])
colnames(cohend_exp_frei) <- "CohensD_exp_f"
```

Grafik

```
#estimated marginal means
# geschätzen Randmittel

# Grafik auf Basiis der LMM nach Umfeld


em_df_exp_f1<- data.frame(emmeans(exp_lmm_frei, "umfeld", by=c("zeit","freigang")))
em_df_exp_f2<- data.frame(emmeans(exp_lmm_frei, "umfeld", by=c("zeit")))
em_df_exp_f3<- data.frame(emmeans(exp_lmm_frei, "freigang", by=c("zeit")))

exp_f1 <- ggplot(em_df_exp_f1, aes(x = zeit, y = emmean, group=umfeld, color=umfeld))+
  geom_line() +
  geom_errorbar(aes(ymin=lower.CL, ymax=upper.CL), width=.4,
                position=position_dodge(0.01)) +
  geom_point(aes(shape=umfeld))+
  scale_colour_manual(values=c("#D700FF","#00C0FA"))+
  scale_y_continuous(limits=c(0,120),breaks=seq(0,120,10)) +
  theme_bw() +
  labs(x="Video Sequence (No)", y = "Exploration Behaviour (sec)",color="Living Environment",shape="Living Environment")

exp_f1



# Grafik auf Basiis der LMM nach Freigang

em_df_exp_f3<- data.frame(emmeans(exp_lmm_frei, "freigang", by="zeit"))

exp_f2 <-ggplot(em_df_exp_f3, aes(x = zeit, y = emmean, group=freigang, color=freigang))+
  geom_line() +
  geom_errorbar(aes(ymin=lower.CL, ymax=upper.CL), width=.4,
                position=position_dodge(0.01)) +
  geom_point(aes(shape=freigang))+
  scale_colour_manual(values=c("#D700FF","#00C0FA"))+
  scale_y_continuous(limits=c(0,120),breaks=seq(0,120,10)) +
  theme_bw() +
  labs(x="Video Sequence (No)", y = "Exploration Behaviour (sec)",color="Outdoor Access",shape="Outdoor Access")

exp_f2
```

Paarweise Vergleich

```
pairs(emmeans(exp_lmm_frei, "umfeld", by="zeit") , adjust="dunnet")
```

```
zeit = 1:
 contrast                      estimate   SE  df t.ratio p.value
 Private Home - Animal Shelter     1.21 20.3 148   0.060  0.9526

zeit = 2:
 contrast                      estimate   SE  df t.ratio p.value
 Private Home - Animal Shelter    -6.77 20.3 148  -0.334  0.7387

zeit = 3:
 contrast                      estimate   SE  df t.ratio p.value
 Private Home - Animal Shelter   -22.55 20.3 148  -1.113  0.2674

zeit = 4:
 contrast                      estimate   SE  df t.ratio p.value
 Private Home - Animal Shelter    -7.61 20.3 148  -0.376  0.7076

zeit = 5:
 contrast                      estimate   SE  df t.ratio p.value
 Private Home - Animal Shelter   -34.29 20.3 148  -1.693  0.0926

Results are averaged over the levels of: freigang
Degrees-of-freedom method: kenward-roger
```

```
pairs(emmeans(exp_lmm_frei, "zeit", by="umfeld") , adjust="dunnet")
```

```
umfeld = Private Home:
 contrast      estimate   SE  df t.ratio p.value
 zeit1 - zeit2    -2.58  5.3 312  -0.487  0.9897
 zeit1 - zeit3    11.28  5.3 312   2.128  0.2189
 zeit1 - zeit4    12.49  5.3 312   2.357  0.1346
 zeit1 - zeit5     8.06  5.3 312   1.520  0.5703
 zeit2 - zeit3    13.86  5.3 312   2.615  0.0720
 zeit2 - zeit4    15.07  5.3 312   2.844  0.0388
 zeit2 - zeit5    10.64  5.3 312   2.007  0.2758
 zeit3 - zeit4     1.21  5.3 312   0.229  0.9995
 zeit3 - zeit5    -3.22  5.3 312  -0.608  0.9763
 zeit4 - zeit5    -4.44  5.3 312  -0.837  0.9264

umfeld = Animal Shelter:
 contrast      estimate   SE  df t.ratio p.value
 zeit1 - zeit2   -10.56 16.4 312  -0.644  0.9708
 zeit1 - zeit3   -12.48 16.4 312  -0.762  0.9469
 zeit1 - zeit4     3.67 16.4 312   0.224  0.9995
 zeit1 - zeit5   -27.44 16.4 312  -1.675  0.4691
 zeit2 - zeit3    -1.92 16.4 312  -0.117  1.0000
 zeit2 - zeit4    14.23 16.4 312   0.869  0.9166
 zeit2 - zeit5   -16.88 16.4 312  -1.031  0.8543
 zeit3 - zeit4    16.15 16.4 312   0.986  0.8734
 zeit3 - zeit5   -14.96 16.4 312  -0.913  0.9014
 zeit4 - zeit5   -31.12 16.4 312  -1.899  0.3332

Results are averaged over the levels of: freigang
Degrees-of-freedom method: kenward-roger
P value adjustment: dunnettx method for 10 tests
```

```
pairs(emmeans(exp_lmm_frei, "zeit", by="freigang") , adjust="dunnet")
```

```
freigang = Outdoor:
 contrast      estimate    SE  df t.ratio p.value
 zeit1 - zeit2   4.1923  7.16 312   0.585  0.9794
 zeit1 - zeit3  -0.8974  7.16 312  -0.125  0.9999
 zeit1 - zeit4   0.0231  7.16 312   0.003  1.0000
 zeit1 - zeit5  -1.0423  7.16 312  -0.146  0.9999
 zeit2 - zeit3  -5.0897  7.16 312  -0.711  0.9584
 zeit2 - zeit4  -4.1692  7.16 312  -0.582  0.9799
 zeit2 - zeit5  -5.2346  7.16 312  -0.731  0.9541
 zeit3 - zeit4   0.9205  7.16 312   0.129  0.9999
 zeit3 - zeit5  -0.1449  7.16 312  -0.020  1.0000
 zeit4 - zeit5  -1.0654  7.16 312  -0.149  0.9999

freigang = Indoor:
 contrast      estimate    SE  df t.ratio p.value
 zeit1 - zeit2 -17.3311 15.66 312  -1.107  0.8183
 zeit1 - zeit3  -0.3040 15.66 312  -0.019  1.0000
 zeit1 - zeit4  16.1419 15.66 312   1.031  0.8542
 zeit1 - zeit5 -18.3446 15.66 312  -1.171  0.7847
 zeit2 - zeit3  17.0270 15.66 312   1.087  0.8279
 zeit2 - zeit4  33.4730 15.66 312   2.137  0.2150
 zeit2 - zeit5  -1.0135 15.66 312  -0.065  1.0000
 zeit3 - zeit4  16.4459 15.66 312   1.050  0.8454
 zeit3 - zeit5 -18.0405 15.66 312  -1.152  0.7951
 zeit4 - zeit5 -34.4865 15.66 312  -2.202  0.1884

Results are averaged over the levels of: umfeld
Degrees-of-freedom method: kenward-roger
P value adjustment: dunnettx method for 10 tests
```

```
pairs(emmeans(exp_lmm_frei, "freigang", by="zeit") , adjust="dunnet")
```

```
zeit = 1:
 contrast         estimate   SE  df t.ratio p.value
 Outdoor - Indoor     9.23 20.3 148   0.455  0.6494

zeit = 2:
 contrast         estimate   SE  df t.ratio p.value
 Outdoor - Indoor   -12.30 20.3 148  -0.607  0.5448

zeit = 3:
 contrast         estimate   SE  df t.ratio p.value
 Outdoor - Indoor     9.82 20.3 148   0.485  0.6286

zeit = 4:
 contrast         estimate   SE  df t.ratio p.value
 Outdoor - Indoor    25.35 20.3 148   1.251  0.2128

zeit = 5:
 contrast         estimate   SE  df t.ratio p.value
 Outdoor - Indoor    -8.08 20.3 148  -0.399  0.6907

Results are averaged over the levels of: umfeld
Degrees-of-freedom method: kenward-roger
```

```
confint(pairs(emmeans(exp_lmm_frei,~umfeld)))
```

```
 contrast                      estimate   SE df lower.CL upper.CL
 Private Home - Animal Shelter      -14 17.1 78      -48       20

Results are averaged over the levels of: zeit, freigang
Degrees-of-freedom method: kenward-roger
Confidence level used: 0.95
```

```
confint(pairs(emmeans(exp_lmm_frei,~zeit)))
```

```
 contrast      estimate   SE  df lower.CL upper.CL
 zeit1 - zeit2   -6.569 8.61 312   -30.19    17.06
 zeit1 - zeit3   -0.601 8.61 312   -24.23    23.02
 zeit1 - zeit4    8.082 8.61 312   -15.54    31.71
 zeit1 - zeit5   -9.693 8.61 312   -33.32    13.93
 zeit2 - zeit3    5.969 8.61 312   -17.66    29.59
 zeit2 - zeit4   14.652 8.61 312    -8.97    38.28
 zeit2 - zeit5   -3.124 8.61 312   -26.75    20.50
 zeit3 - zeit4    8.683 8.61 312   -14.94    32.31
 zeit3 - zeit5   -9.093 8.61 312   -32.72    14.53
 zeit4 - zeit5  -17.776 8.61 312   -41.40     5.85

Results are averaged over the levels of: umfeld, freigang
Degrees-of-freedom method: kenward-roger
Confidence level used: 0.95
Conf-level adjustment: tukey method for comparing a family of 5 estimates
```

```
confint(pairs(emmeans(exp_lmm_frei,~freigang)))
```

```
 contrast         estimate   SE df lower.CL upper.CL
 Outdoor - Indoor      4.8 17.1 78    -29.2     38.8

Results are averaged over the levels of: umfeld, zeit
Degrees-of-freedom method: kenward-roger
Confidence level used: 0.95
```

Residuen

```
exp_frei_diagnose <- broom.mixed::augment(exp_lmm_frei)

#Normalverteiung - annähernd normalverteilt

exp_frei_r1 <- ggplot(exp_frei_diagnose, aes(x = .resid)) +

geom_histogram(aes(x = .resid, y = ..density..)) +

geom_density(aes(colour = "density estimate")) +

geom_function(aes(colour = "normal curve"), fun = dnorm,

args = list(mean = mean(exp_diagnose$.resid),sd = sd(exp_frei_diagnose$.resid))) +

scale_colour_discrete(name = "Curve") +

theme(legend.position = "top")


exp_frei_r2 <- ggplot(exp_frei_diagnose, aes(sample = .resid)) +

geom_qq() +

geom_qq_line()

exp_frei_r1 + exp_frei_r2



#DHARMa - prüft automatisch Residuen auf Normalverteilung & Abweichungen

q_unit_f <- simulateResiduals(exp_lmm_frei)

plot(q_unit_f)



#Homoskedastizität

exp_frei_r3 <- ggplot(exp_frei_diagnose, aes(x = .fitted, y = .resid)) + geom_point()


exp_frei_r4 <- ggplot(exp_frei_diagnose, aes(x = seq_along(.resid), y = .resid)) +

geom_point() + geom_smooth() + xlab("Index")


exp_frei_r3 + exp_frei_r4



###Ausreißer kontrollieren

#standardisierte Residuen ansehen

favstats(scale(exp_frei_diagnose$.resid))
```

```
       min         Q1     median        Q3      max          mean sd   n missing
 -2.901584 -0.5930701 -0.0183272 0.6237206 2.759582 -1.26254e-17  1 410       0
```

```
exp_frei_r5 <- ggplot(exp_frei_diagnose, aes(y = .hat, label = as.numeric(rownames(exp_frei_diagnose)))) +
geom_boxplot() +
ggrepel::geom_text_repel(aes(x = 0), max.overlaps = 30)


exp_frei_r6 <- ggplot(exp_frei_diagnose, aes(y = .cooksd)) +

geom_boxplot()


exp_frei_r5 + exp_frei_r6
```

Spielverhalten (ply)

Modelle Umfeld + Zeit

```
# ANOVA rechnen

ply_m1 <- aov_car(sec ~ umfeld *zeit+ Error(versuch/zeit),
                  data=cat_long %>% dplyr::filter(verhalten=="ply"),
                  type=3)
summary(ply_m1)
```

```
Univariate Type III Repeated-Measures ANOVA Assuming Sphericity

            Sum Sq num Df Error SS den Df F value   Pr(>F)
(Intercept)  33765      1   332053     76  7.7282 0.006849 **
umfeld       31864      1   332053     76  7.2930 0.008530 **
zeit          2230      4   253269    304  0.6692 0.613792
umfeld:zeit   2236      4   253269    304  0.6711 0.612465
---
Signif. codes:  0 '***' 0.001 '**' 0.01 '*' 0.05 '.' 0.1 ' ' 1


Mauchly Tests for Sphericity

            Test statistic    p-value
zeit               0.66552 0.00039104
umfeld:zeit        0.66552 0.00039104


Greenhouse-Geisser and Huynh-Feldt Corrections
 for Departure from Sphericity

             GG eps Pr(>F[GG])
zeit        0.84261     0.5887
umfeld:zeit 0.84261     0.5874

               HF eps Pr(>F[HF])
zeit        0.8864278  0.5960680
umfeld:zeit 0.8864278  0.5948045
```

```
effectsize::omega_squared(ply_m1)
```

```
# Effect Size for ANOVA (Type III)

Parameter   | Omega2 (partial) |       95% CI
---------------------------------------------
umfeld      |             0.07 | [0.01, 1.00]
zeit        |             0.00 | [0.00, 1.00]
umfeld:zeit |             0.00 | [0.00, 1.00]

- One-sided CIs: upper bound fixed at [1.00].
```

```
# LMM rechnen

ply_lmm <- lmer(log(ply+1) ~ umfeld*zeit + (1|versuch), data = cat_l)

summary(ply_lmm)
```

```
Linear mixed model fit by REML. t-tests use Satterthwaite's method [
lmerModLmerTest]
Formula: log(ply + 1) ~ umfeld * zeit + (1 | versuch)
   Data: cat_l

REML criterion at convergence: 1459.9

Scaled residuals:
    Min      1Q  Median      3Q     Max
-3.2342 -0.4417 -0.0534  0.4188  2.8521

Random effects:
 Groups   Name        Variance Std.Dev.
 versuch  (Intercept) 2.212    1.487
 Residual             1.376    1.173
Number of obs: 406, groups:  versuch, 82

Fixed effects:
                            Estimate Std. Error        df t value Pr(>|t|)
(Intercept)                  1.48391    0.23143 157.96401   6.412 1.58e-09 ***
umfeldAnimal Shelter        -0.91938    0.54109 157.96401  -1.699   0.0913 .
zeit2                       -0.26923    0.20269 315.79285  -1.328   0.1850
zeit3                        0.21707    0.20269 315.79285   1.071   0.2850
zeit4                        0.49402    0.20269 315.79285   2.437   0.0153 *
zeit5                       -0.02794    0.20269 315.79285  -0.138   0.8905
umfeldAnimal Shelter:zeit2   0.43700    0.47391 315.79285   0.922   0.3572
umfeldAnimal Shelter:zeit3   0.21764    0.47391 315.79285   0.459   0.6464
umfeldAnimal Shelter:zeit4  -0.46302    0.51495 318.40005  -0.899   0.3693
umfeldAnimal Shelter:zeit5   0.05536    0.47391 315.79285   0.117   0.9071
---
Signif. codes:  0 '***' 0.001 '**' 0.01 '*' 0.05 '.' 0.1 ' ' 1

Correlation of Fixed Effects:
            (Intr) umflAS zeit2  zeit3  zeit4  zeit5  umAS:2 umAS:3 umAS:4
umfldAnmlSh -0.428
zeit2       -0.438  0.187
zeit3       -0.438  0.187  0.500
zeit4       -0.438  0.187  0.500  0.500
zeit5       -0.438  0.187  0.500  0.500  0.500
umfldAShl:2  0.187 -0.438 -0.428 -0.214 -0.214 -0.214
umfldAShl:3  0.187 -0.438 -0.214 -0.428 -0.214 -0.214  0.500
umfldAShl:4  0.172 -0.403 -0.197 -0.197 -0.394 -0.197  0.460  0.460
umfldAShl:5  0.187 -0.438 -0.214 -0.214 -0.214 -0.428  0.500  0.500  0.460
```

```
anova(ply_lmm)
```

```
Type III Analysis of Variance Table with Satterthwaite's method
            Sum Sq Mean Sq NumDF  DenDF F value  Pr(>F)
umfeld      5.0921  5.0921     1  80.82  3.6998 0.05794 .
zeit        5.5491  1.3873     4 316.74  1.0080 0.40344
umfeld:zeit 4.5345  1.1336     4 316.74  0.8237 0.51084
---
Signif. codes:  0 '***' 0.001 '**' 0.01 '*' 0.05 '.' 0.1 ' ' 1
```

Robust rechnen

```
# Robust linear mixed model

ply_rlmm <- rlmer(exp ~ umfeld*zeit + (1|versuch), data = cat_l)
summary(ply_rlmm)
```

```
Robust linear mixed model fit by DAStau
Formula: exp ~ umfeld * zeit + (1 | versuch)
   Data: cat_l

Scaled residuals:
     Min       1Q   Median       3Q      Max
-3.04403 -0.52410 -0.04899  0.60542  2.72036

Random effects:
 Groups   Name        Variance Std.Dev.
 versuch  (Intercept) 2288.6   47.84
 Residual              852.8   29.20
Number of obs: 410, groups: versuch, 82

Fixed effects:
                           Estimate Std. Error t value
(Intercept)                  70.420      7.023  10.027
umfeldAnimal Shelter         13.779     16.420   0.839
zeit2                         1.214      5.175   0.235
zeit3                       -13.748      5.175  -2.657
zeit4                       -13.483      5.175  -2.605
zeit5                       -10.332      5.175  -1.997
umfeldAnimal Shelter:zeit2   -6.374     12.099  -0.527
umfeldAnimal Shelter:zeit3   20.684     12.099   1.710
umfeldAnimal Shelter:zeit4   13.572     12.099   1.122
umfeldAnimal Shelter:zeit5   18.986     12.099   1.569

Correlation of Fixed Effects:
            (Intr) umflAS zeit2  zeit3  zeit4  zeit5  umAS:2 umAS:3 umAS:4
umfldAnmlSh -0.428
zeit2       -0.368  0.158
zeit3       -0.368  0.158  0.500
zeit4       -0.368  0.158  0.500  0.500
zeit5       -0.368  0.158  0.500  0.500  0.500
umfldAShl:2  0.158 -0.368 -0.428 -0.214 -0.214 -0.214
umfldAShl:3  0.158 -0.368 -0.214 -0.428 -0.214 -0.214  0.500
umfldAShl:4  0.158 -0.368 -0.214 -0.214 -0.428 -0.214  0.500  0.500
umfldAShl:5  0.158 -0.368 -0.214 -0.214 -0.214 -0.428  0.500  0.500  0.500

Robustness weights for the residuals:
 341 weights are ~= 1. The remaining 69 ones are summarized as
   Min. 1st Qu.  Median    Mean 3rd Qu.    Max.
  0.442   0.603   0.837   0.785   0.954   0.999

Robustness weights for the random effects:
 74 weights are ~= 1. The remaining 8 ones are
    3     5     6     8     9    15    38    41
0.989 0.723 0.978 0.929 0.996 0.758 0.817 0.990

Rho functions used for fitting:
  Residuals:
    eff: smoothed Huber (k = 1.345, s = 10)
    sig: smoothed Huber, Proposal 2 (k = 1.345, s = 10)
  Random Effects, variance component 1 (versuch):
    eff: smoothed Huber (k = 1.345, s = 10)
    vcp: smoothed Huber, Proposal 2 (k = 1.345, s = 10)
```

```
ply_rlmm2 <- rlmer(exp ~ umfeld*zeit*freigang + (1|versuch), data = cat_l)
summary(ply_rlmm2)
```

```
Robust linear mixed model fit by DAStau
Formula: exp ~ umfeld * zeit * freigang + (1 | versuch)
   Data: cat_l

Scaled residuals:
     Min       1Q   Median       3Q      Max
-2.94635 -0.51786 -0.02029  0.55644  2.79022

Random effects:
 Groups   Name        Variance Std.Dev.
 versuch  (Intercept) 2127.7   46.13
 Residual              810.7   28.47
Number of obs: 410, groups: versuch, 82

Fixed effects:
                                          Estimate Std. Error t value
(Intercept)                                 50.227     10.150   4.948
umfeldAnimal Shelter                        43.201     18.461   2.340
zeit2                                        2.166      7.540   0.287
zeit3                                       -2.836      7.540  -0.376
zeit4                                       -0.486      7.540  -0.064
zeit5                                        3.503      7.540   0.465
freigangIndoor                              37.099     13.659   2.716
umfeldAnimal Shelter:zeit2                 -15.049     13.713  -1.097
umfeldAnimal Shelter:zeit3                   7.067     13.713   0.515
umfeldAnimal Shelter:zeit4                   1.313     13.713   0.096
umfeldAnimal Shelter:zeit5                  -4.274     13.713  -0.312
umfeldAnimal Shelter:freigangIndoor        -91.526     44.383  -2.062
zeit2:freigangIndoor                        -1.664     10.146  -0.164
zeit3:freigangIndoor                       -19.972     10.146  -1.968
zeit4:freigangIndoor                       -23.747     10.146  -2.340
zeit5:freigangIndoor                       -25.591     10.146  -2.522
umfeldAnimal Shelter:zeit2:freigangIndoor   48.048     32.968   1.457
umfeldAnimal Shelter:zeit3:freigangIndoor   38.242     32.968   1.160
umfeldAnimal Shelter:zeit4:freigangIndoor   16.421     32.968   0.498
umfeldAnimal Shelter:zeit5:freigangIndoor   84.863     32.968   2.574

Correlation of Fixed Effects:
            (Intr) umflAS zeit2  zeit3  zeit4  zeit5  frgngI umAS:2 umAS:3
umfldAnmlSh -0.550
zeit2       -0.371  0.204
zeit3       -0.371  0.204  0.500
zeit4       -0.371  0.204  0.500  0.500
zeit5       -0.371  0.204  0.500  0.500  0.500
freigngIndr -0.743  0.409  0.276  0.276  0.276  0.276
umfldAShl:2  0.204 -0.371 -0.550 -0.275 -0.275 -0.275 -0.152
umfldAShl:3  0.204 -0.371 -0.275 -0.550 -0.275 -0.275 -0.152  0.500
umfldAShl:4  0.204 -0.371 -0.275 -0.275 -0.550 -0.275 -0.152  0.500  0.500
umfldAShl:5  0.204 -0.371 -0.275 -0.275 -0.275 -0.550 -0.152  0.500  0.500
umfldAShl:I  0.229 -0.416 -0.085 -0.085 -0.085 -0.085 -0.308  0.154  0.154
zt2:frgngIn  0.276 -0.152 -0.743 -0.372 -0.372 -0.372 -0.371  0.409  0.204
zt3:frgngIn  0.276 -0.152 -0.372 -0.743 -0.372 -0.372 -0.371  0.204  0.409
zt4:frgngIn  0.276 -0.152 -0.372 -0.372 -0.743 -0.372 -0.371  0.204  0.204
zt5:frgngIn  0.276 -0.152 -0.372 -0.372 -0.372 -0.743 -0.371  0.204  0.204
umfldAS:2:I -0.085  0.154  0.229  0.114  0.114  0.114  0.114 -0.416 -0.208
umfldAS:3:I -0.085  0.154  0.114  0.229  0.114  0.114  0.114 -0.208 -0.416
umfldAS:4:I -0.085  0.154  0.114  0.114  0.229  0.114  0.114 -0.208 -0.208
umfldAS:5:I -0.085  0.154  0.114  0.114  0.114  0.229  0.114 -0.208 -0.208
            umAS:4 umAS:5 umAS:I zt2:fI zt3:fI zt4:fI zt5:fI uAS:2: uAS:3:
umfldAnmlSh
zeit2
zeit3
zeit4
zeit5
freigngIndr
umfldAShl:2
umfldAShl:3
umfldAShl:4
umfldAShl:5  0.500
umfldAShl:I  0.154  0.154
zt2:frgngIn  0.204  0.204  0.114
zt3:frgngIn  0.204  0.204  0.114  0.500
zt4:frgngIn  0.409  0.204  0.114  0.500  0.500
zt5:frgngIn  0.204  0.409  0.114  0.500  0.500  0.500
umfldAS:2:I -0.208 -0.208 -0.371 -0.308 -0.154 -0.154 -0.154
umfldAS:3:I -0.208 -0.208 -0.371 -0.154 -0.308 -0.154 -0.154  0.500
umfldAS:4:I -0.416 -0.208 -0.371 -0.154 -0.154 -0.308 -0.154  0.500  0.500
umfldAS:5:I -0.208 -0.416 -0.371 -0.154 -0.154 -0.154 -0.308  0.500  0.500
            uAS:4:
umfldAnmlSh
zeit2
zeit3
zeit4
zeit5
freigngIndr
umfldAShl:2
umfldAShl:3
umfldAShl:4
umfldAShl:5
umfldAShl:I
zt2:frgngIn
zt3:frgngIn
zt4:frgngIn
zt5:frgngIn
umfldAS:2:I
umfldAS:3:I
umfldAS:4:I
umfldAS:5:I  0.500

Robustness weights for the residuals:
 346 weights are ~= 1. The remaining 64 ones are summarized as
   Min. 1st Qu.  Median    Mean 3rd Qu.    Max.
  0.456   0.584   0.797   0.760   0.926   0.998

Robustness weights for the random effects:
 72 weights are ~= 1. The remaining 10 ones are
    5     6     8    15    20    38    63    66    79    82
0.741 0.897 0.842 0.779 0.870 0.893 0.931 0.949 0.929 0.889

Rho functions used for fitting:
  Residuals:
    eff: smoothed Huber (k = 1.345, s = 10)
    sig: smoothed Huber, Proposal 2 (k = 1.345, s = 10)
  Random Effects, variance component 1 (versuch):
    eff: smoothed Huber (k = 1.345, s = 10)
    vcp: smoothed Huber, Proposal 2 (k = 1.345, s = 10)
```

Effektstärke

```
##Cohen's d berechnen


reffects_ply<-broom.mixed::tidy(ply_lmm, effects = c("ran_pars"), scale="vcov")


md_ply <-summary(ply_lmm)$coef[2] #Md = Mittelwertsdifferenz
sdPart_ply<-sqrt(sum(reffects_ply$estimate)) #Random-Effects zusammenzählen
##Cohen´s d für alle Faktoren



cohend_ply <- data.frame(cbind(summary(ply_lmm)$coef[, 1]/sdPart_ply)[c(2,3,4,5,6,7,8,9,10),])
colnames(cohend_ply) <- "CohensD_ply"
```

Grafik

```
# Grafik auf Basis der LMM

em_df_ply_m1<- data.frame(emmeans(ply_lmm, "umfeld", by="zeit"))

ggplot(em_df_ply_m1, aes(x = zeit, y = emmean, group=umfeld, color=umfeld))+
  geom_line() +
  geom_errorbar(aes(ymin=lower.CL, ymax=upper.CL), width=.4,
                position=position_dodge(0.01)) +
  geom_point(aes(shape=umfeld))+
  scale_colour_manual(values=c("#D700FF","#00C0FA"))+
  scale_y_continuous(limits=c(0,25),breaks=seq(0,25,2)) +
  theme_bw() +
  labs(x="Video Sequence (No)", y = "Play Behaviour (sec)",color="Living Environment",shape="Living Environment")



# log-transformierte unabhängige Variable - muss zurück transformiert werden für die Grafik
# First transform the values back to seconds
em_df_ply_m1$emmean <- exp(em_df_ply_m1$emmean) - 1
em_df_ply_m1$lower.CL <- exp(em_df_ply_m1$lower.CL) - 1
em_df_ply_m1$upper.CL <- exp(em_df_ply_m1$upper.CL) - 1

# Create the plot with transformed values
ply_g1 <-ggplot(em_df_ply_m1, aes(x = zeit, y = emmean, group=umfeld, color=umfeld))+
  geom_line() +
  geom_errorbar(aes(ymin=lower.CL, ymax=upper.CL), width=.4,
                position=position_dodge(0.01)) +
  geom_point(aes(shape=umfeld))+
  scale_colour_manual(values=c("#D700FF","#00C0FA"))+
  scale_y_continuous(limits=c(0,20), breaks=seq(0,20,2)) +
  theme_bw() +
  labs(x="Video Sequence (No)", y = "Play Behaviour (sec)",color="Living Environment",shape="Living Environment")

ply_g1
```

Paarweise Vergleiche

```
pairs(emmeans(ply_lmm, "umfeld", by="zeit") , adjust="dunnet")
```

```
zeit = 1:
 contrast                      estimate    SE  df t.ratio p.value
 Private Home - Animal Shelter    0.919 0.541 158   1.699  0.0913

zeit = 2:
 contrast                      estimate    SE  df t.ratio p.value
 Private Home - Animal Shelter    0.482 0.541 158   0.891  0.3740

zeit = 3:
 contrast                      estimate    SE  df t.ratio p.value
 Private Home - Animal Shelter    0.702 0.541 158   1.297  0.1966

zeit = 4:
 contrast                      estimate    SE  df t.ratio p.value
 Private Home - Animal Shelter    1.382 0.577 193   2.394  0.0176

zeit = 5:
 contrast                      estimate    SE  df t.ratio p.value
 Private Home - Animal Shelter    0.864 0.541 158   1.597  0.1123

Note: contrasts are still on the log(mu + 1) scale. Consider using
      regrid() if you want contrasts of back-transformed estimates.
Degrees-of-freedom method: kenward-roger
```

```
pairs(emmeans(ply_lmm, "zeit", by="umfeld") , adjust="dunnet")
```

```
umfeld = Private Home:
 contrast      estimate    SE  df t.ratio p.value
 zeit1 - zeit2  0.26923 0.203 316   1.328  0.6935
 zeit1 - zeit3 -0.21707 0.203 316  -1.071  0.8357
 zeit1 - zeit4 -0.49402 0.203 316  -2.437  0.1117
 zeit1 - zeit5  0.02794 0.203 316   0.138  0.9999
 zeit2 - zeit3 -0.48630 0.203 316  -2.399  0.1221
 zeit2 - zeit4 -0.76325 0.203 316  -3.766  0.0019
 zeit2 - zeit5 -0.24129 0.203 316  -1.190  0.7743
 zeit3 - zeit4 -0.27695 0.203 316  -1.366  0.6697
 zeit3 - zeit5  0.24501 0.203 316   1.209  0.7641
 zeit4 - zeit5  0.52196 0.203 316   2.575  0.0797

umfeld = Animal Shelter:
 contrast      estimate    SE  df t.ratio p.value
 zeit1 - zeit2 -0.16777 0.428 316  -0.392  0.9955
 zeit1 - zeit3 -0.43471 0.428 316  -1.015  0.8612
 zeit1 - zeit4 -0.03101 0.473 319  -0.065  1.0000
 zeit1 - zeit5 -0.02742 0.428 316  -0.064  1.0000
 zeit2 - zeit3 -0.26694 0.428 316  -0.623  0.9741
 zeit2 - zeit4  0.13677 0.473 319   0.289  0.9986
 zeit2 - zeit5  0.14035 0.428 316   0.328  0.9978
 zeit3 - zeit4  0.40371 0.473 319   0.853  0.9217
 zeit3 - zeit5  0.40729 0.428 316   0.951  0.8875
 zeit4 - zeit5  0.00358 0.473 319   0.008  1.0000

Note: contrasts are still on the log(mu + 1) scale. Consider using
      regrid() if you want contrasts of back-transformed estimates.
Degrees-of-freedom method: kenward-roger
P value adjustment: dunnettx method for 10 tests
```

```
confint(pairs(emmeans(ply_lmm,~umfeld)))
```

```
 contrast                      estimate    SE df lower.CL upper.CL
 Private Home - Animal Shelter     0.87 0.452 81  -0.0299     1.77

Results are averaged over the levels of: zeit
Note: contrasts are still on the log(mu + 1) scale. Consider using
      regrid() if you want contrasts of back-transformed estimates.
Degrees-of-freedom method: kenward-roger
Confidence level used: 0.95
```

```
confint(pairs(emmeans(ply_lmm,~zeit)))
```

```
 contrast       estimate    SE  df lower.CL upper.CL
 zeit1 - zeit2  0.050728 0.237 316   -0.599    0.701
 zeit1 - zeit3 -0.325892 0.237 316   -0.976    0.324
 zeit1 - zeit4 -0.262514 0.258 319   -0.969    0.444
 zeit1 - zeit5  0.000257 0.237 316   -0.650    0.650
 zeit2 - zeit3 -0.376620 0.237 316   -1.027    0.273
 zeit2 - zeit4 -0.313242 0.258 319   -1.020    0.393
 zeit2 - zeit5 -0.050471 0.237 316   -0.701    0.600
 zeit3 - zeit4  0.063379 0.258 319   -0.643    0.770
 zeit3 - zeit5  0.326149 0.237 316   -0.324    0.976
 zeit4 - zeit5  0.262771 0.258 319   -0.444    0.969

Results are averaged over the levels of: umfeld
Note: contrasts are still on the log(mu + 1) scale. Consider using
      regrid() if you want contrasts of back-transformed estimates.
Degrees-of-freedom method: kenward-roger
Confidence level used: 0.95
Conf-level adjustment: tukey method for comparing a family of 5 estimates
```

Residuen

```
ply_diagnose <- broom.mixed::augment(ply_lmm)

#Normalverteiung - annähernd normalverteilt

ply_r1 <- ggplot(ply_diagnose, aes(x = .resid)) +
geom_histogram(aes(x = .resid, y = ..density..)) +
geom_density(aes(colour = "density estimate")) +
geom_function(aes(colour = "normal curve"), fun = dnorm,

args = list(mean = mean(ply_diagnose$.resid),sd = sd(ply_diagnose$.resid))) +

scale_colour_discrete(name = "Curve") +
```

```
scale_colour_discrete(name = "Curve") +

theme(legend.position = "top")


ply_r2 <- ggplot(ply_diagnose, aes(sample = .resid)) +

geom_qq() +

geom_qq_line()

ply_r1 + ply_r2



#DHARMa - prüft automatisch Residuen auf Normalverteilung & Abweichungen

q_unit <- simulateResiduals(ply_lmm)

plot(q_unit)



#Homoskedastizität

ply_r3 <- ggplot(ply_diagnose, aes(x = .fitted, y = .resid)) + geom_point()


ply_r4 <- ggplot(ply_diagnose, aes(x = seq_along(.resid), y = .resid)) +

geom_point() + geom_smooth() + xlab("Index")


ply_r3 + ply_r4



###Ausreißer kontrollieren

#standardisierte Residuen ansehen

# cutoff aus Field für Ausreißer von Regression auf LMM
#ply_cutoff <- 3*ply_lmm$rank/(ply_lmm$rank+ply_lmm$df.residual)
#  geom_hline(yintercept = m4_cutoff, linetype = "dashed", color = "darkgreen") +

# bei allen einfügen: label = as.numeric(rownames(ply_diagnose))

favstats(scale(ply_diagnose$.resid))

      min         Q1      median        Q3      max          mean sd   n missing
 -3.61064 -0.4930766 -0.05962777 0.4675427 3.184072 4.710244e-17  1 406       0

ply_r5 <- ggplot(ply_diagnose, aes(y = .hat, label = as.numeric(rownames(ply_diagnose)))) +

geom_boxplot() +

ggrepel::geom_text_repel(aes(x = 0), max.overlaps = 30)


ply_r6 <- ggplot(ply_diagnose, aes(y = .cooksd)) +

geom_boxplot()


ply_r5 + ply_r6
```

Modelle Umfeld + Zeit + Freigang

```
# ANOVA rechnen

ply_frei_m1 <- aov_car(sec ~ umfeld *zeit+ Error(versuch/zeit),
                  data=cat_long %>% dplyr::filter(verhalten=="ply"),
                  type=3)
summary(ply_frei_m1)


Univariate Type III Repeated-Measures ANOVA Assuming Sphericity

            Sum Sq num Df Error SS den Df F value   Pr(>F)
(Intercept)  33765      1   332053     76  7.7282 0.006849 **
umfeld       31864      1   332053     76  7.2930 0.008530 **
zeit          2230      4   253269    304  0.6692 0.613792
umfeld:zeit   2236      4   253269    304  0.6711 0.612465
---
Signif. codes:  0 '***' 0.001 '**' 0.01 '*' 0.05 '.' 0.1 ' ' 1


Mauchly Tests for Sphericity

            Test statistic    p-value
zeit               0.66552 0.00039104
umfeld:zeit        0.66552 0.00039104


Greenhouse-Geisser and Huynh-Feldt Corrections
 for Departure from Sphericity

             GG eps Pr(>F[GG])
zeit        0.84261     0.5887
umfeld:zeit 0.84261     0.5874

               HF eps Pr(>F[HF])
zeit        0.8864278  0.5960680
umfeld:zeit 0.8864278  0.5948045

effectsize::omega_squared(ply_frei_m1)

# Effect Size for ANOVA (Type III)

Parameter   | Omega2 (partial) |       95% CI
---------------------------------------------
umfeld      |             0.07 | [0.01, 1.00]
zeit        |             0.00 | [0.00, 1.00]
umfeld:zeit |             0.00 | [0.00, 1.00]

- One-sided CIs: upper bound fixed at [1.00].

# LMM rechnen


cat_l <- cat_long %>%
  pivot_wider(names_from = verhalten, values_from = sec)


ply_lmm_frei <- lmer(log(ply+1) ~ umfeld*zeit*freigang + (1|versuch), data = cat_l)
summary(ply_lmm_frei)

Linear mixed model fit by REML. t-tests use Satterthwaite's method [
lmerModLmerTest]
Formula: log(ply + 1) ~ umfeld * zeit * freigang + (1 | versuch)
   Data: cat_l

REML criterion at convergence: 1440.2

Scaled residuals:
    Min      1Q  Median      3Q     Max
-3.1801 -0.4359 -0.0615  0.4242  2.6225

Random effects:
 Groups   Name        Variance Std.Dev.
 versuch  (Intercept) 2.237    1.496
 Residual             1.374    1.172
Number of obs: 406, groups:  versuch, 82

Fixed effects:
                                           Estimate Std. Error        df
(Intercept)                                 1.29473    0.34695 153.07121
umfeldAnimal Shelter                       -0.64334    0.63100 153.07121
zeit2                                      -0.13424    0.30263 307.79929
zeit3                                       0.14450    0.30263 307.79929
zeit4                                       0.55942    0.30263 307.79929
zeit5                                      -0.38624    0.30263 307.79929
freigangIndoor                              0.34258    0.46688 153.07121
umfeldAnimal Shelter:zeit2                  0.32783    0.55040 307.79929
umfeldAnimal Shelter:zeit3                  0.23329    0.55040 307.79929
umfeldAnimal Shelter:zeit4                 -0.59868    0.58439 309.65445
umfeldAnimal Shelter:zeit5                  0.18369    0.55040 307.79929
umfeldAnimal Shelter:freigangIndoor        -0.99396    1.51702 153.07121
zeit2:freigangIndoor                       -0.24444    0.40724 307.79929
zeit3:freigangIndoor                        0.13142    0.40724 307.79929
zeit4:freigangIndoor                       -0.11842    0.40724 307.79929
zeit5:freigangIndoor                        0.64883    0.40724 307.79929
umfeldAnimal Shelter:zeit2:freigangIndoor   0.05085    1.32325 307.79929
umfeldAnimal Shelter:zeit3:freigangIndoor   0.29551    1.32325 307.79929
umfeldAnimal Shelter:zeit4:freigangIndoor   0.66201    1.62028 313.25198
umfeldAnimal Shelter:zeit5:freigangIndoor   1.07599    1.32325 307.79929
                                          t value Pr(>|t|)
(Intercept)                                 3.732 0.000267 ***
umfeldAnimal Shelter                       -1.020 0.309544
zeit2                                      -0.444 0.657653
zeit3                                       0.477 0.633371
zeit4                                       1.849 0.065486 .
zeit5                                      -1.276 0.202817
freigangIndoor                              0.734 0.464215
umfeldAnimal Shelter:zeit2                  0.596 0.551865
umfeldAnimal Shelter:zeit3                  0.424 0.671959
umfeldAnimal Shelter:zeit4                 -1.024 0.306420
umfeldAnimal Shelter:zeit5                  0.334 0.738802
umfeldAnimal Shelter:freigangIndoor        -0.655 0.513318
zeit2:freigangIndoor                       -0.600 0.548797
zeit3:freigangIndoor                        0.323 0.747133
zeit4:freigangIndoor                       -0.291 0.771404
zeit5:freigangIndoor                        1.593 0.112135
umfeldAnimal Shelter:zeit2:freigangIndoor   0.038 0.969371
umfeldAnimal Shelter:zeit3:freigangIndoor   0.223 0.823434
umfeldAnimal Shelter:zeit4:freigangIndoor   0.409 0.683128
umfeldAnimal Shelter:zeit5:freigangIndoor   0.813 0.416764
---
Signif. codes:  0 '***' 0.001 '**' 0.01 '*' 0.05 '.' 0.1 ' ' 1

anova(ply_lmm_frei)

Type III Analysis of Variance Table with Satterthwaite's method
                     Sum Sq Mean Sq NumDF   DenDF F value Pr(>F)
umfeld               2.7369 2.73691     1  80.914  1.9922 0.1619
zeit                 4.5234 1.13085     4 309.596  0.8232 0.5112
freigang             0.0635 0.06352     1  80.914  0.0462 0.8303
umfeld:zeit          2.7509 0.68773     4 309.596  0.5006 0.7353
umfeld:freigang      0.2797 0.27967     1  80.914  0.2036 0.6531
zeit:freigang        7.2186 1.80466     4 309.596  1.3136 0.2648
umfeld:zeit:freigang 1.2297 0.30742     4 309.596  0.2238 0.9250
```

Robust rechnen

```
# Robust linear mixed model

ply_rlmm_frei <- rlmer(exp ~ umfeld*zeit*freigang + (1|versuch), data = cat_l)
summary(ply_rlmm_frei)


Robust linear mixed model fit by DAStau
Formula: exp ~ umfeld * zeit * freigang + (1 | versuch)
   Data: cat_l

Scaled residuals:
     Min       1Q   Median       3Q      Max
-2.94635 -0.51786 -0.02029  0.55644  2.79022

Random effects:
 Groups   Name        Variance Std.Dev.
 versuch  (Intercept) 2127.7   46.13
 Residual              810.7   28.47
Number of obs: 410, groups: versuch, 82

Fixed effects:
                                          Estimate Std. Error t value
(Intercept)                                 50.227     10.150   4.948
umfeldAnimal Shelter                        43.201     18.461   2.340
zeit2                                        2.166      7.540   0.287
zeit3                                       -2.836      7.540  -0.376
zeit4                                       -0.486      7.540  -0.064
zeit5                                        3.503      7.540   0.465
freigangIndoor                              37.099     13.659   2.716
umfeldAnimal Shelter:zeit2                 -15.049     13.713  -1.097
umfeldAnimal Shelter:zeit3                   7.067     13.713   0.515
umfeldAnimal Shelter:zeit4                   1.313     13.713   0.096
umfeldAnimal Shelter:zeit5                  -4.274     13.713  -0.312
umfeldAnimal Shelter:freigangIndoor        -91.526     44.383  -2.062
zeit2:freigangIndoor                        -1.664     10.146  -0.164
zeit3:freigangIndoor                       -19.972     10.146  -1.968
zeit4:freigangIndoor                       -23.747     10.146  -2.340
zeit5:freigangIndoor                       -25.591     10.146  -2.522
umfeldAnimal Shelter:zeit2:freigangIndoor   48.048     32.968   1.457
umfeldAnimal Shelter:zeit3:freigangIndoor   38.242     32.968   1.160
umfeldAnimal Shelter:zeit4:freigangIndoor   16.421     32.968   0.498
umfeldAnimal Shelter:zeit5:freigangIndoor   84.863     32.968   2.574

Correlation of Fixed Effects:
            (Intr) umflAS zeit2  zeit3  zeit4  zeit5  frgngI umAS:2 umAS:3
umfldAnmlSh -0.550
zeit2       -0.371  0.204
zeit3       -0.371  0.204  0.500
zeit4       -0.371  0.204  0.500  0.500
zeit5       -0.371  0.204  0.500  0.500  0.500
freigngIndr -0.743  0.409  0.276  0.276  0.276  0.276
umfldAShl:2  0.204 -0.371 -0.550 -0.275 -0.275 -0.275 -0.152
umfldAShl:3  0.204 -0.371 -0.275 -0.550 -0.275 -0.275 -0.152  0.500
umfldAShl:4  0.204 -0.371 -0.275 -0.275 -0.550 -0.275 -0.152  0.500  0.500
umfldAShl:5  0.204 -0.371 -0.275 -0.275 -0.275 -0.550 -0.152  0.500  0.500
umfldAShl:I  0.229 -0.416 -0.085 -0.085 -0.085 -0.085 -0.308  0.154  0.154
zt2:frgngIn  0.276 -0.152 -0.743 -0.372 -0.372 -0.372 -0.371  0.409  0.204
zt3:frgngIn  0.276 -0.152 -0.372 -0.743 -0.372 -0.372 -0.371  0.204  0.409
zt4:frgngIn  0.276 -0.152 -0.372 -0.372 -0.743 -0.372 -0.371  0.204  0.204
zt5:frgngIn  0.276 -0.152 -0.372 -0.372 -0.372 -0.743 -0.371  0.204  0.204
umfldAS:2:I -0.085  0.154  0.229  0.114  0.114  0.114  0.114 -0.416 -0.208
umfldAS:3:I -0.085  0.154  0.114  0.229  0.114  0.114  0.114 -0.208 -0.416
umfldAS:4:I -0.085  0.154  0.114  0.114  0.229  0.114  0.114 -0.208 -0.208
umfldAS:5:I -0.085  0.154  0.114  0.114  0.114  0.229  0.114 -0.208 -0.208
            umAS:4 umAS:5 umAS:I zt2:fI zt3:fI zt4:fI zt5:fI uAS:2: uAS:3:
umfldAnmlSh
zeit2
zeit3
zeit4
zeit5
freigngIndr
umfldAShl:2
umfldAShl:3
umfldAShl:4
umfldAShl:5  0.500
umfldAShl:I  0.154  0.154
zt2:frgngIn  0.204  0.204  0.114
zt3:frgngIn  0.204  0.204  0.114  0.500
zt4:frgngIn  0.409  0.204  0.114  0.500  0.500
zt5:frgngIn  0.204  0.409  0.114  0.500  0.500  0.500
umfldAS:2:I -0.208 -0.208 -0.371 -0.308 -0.154 -0.154 -0.154
umfldAS:3:I -0.208 -0.208 -0.371 -0.154 -0.308 -0.154 -0.154  0.500
umfldAS:4:I -0.416 -0.208 -0.371 -0.154 -0.154 -0.308 -0.154  0.500  0.500
umfldAS:5:I -0.208 -0.416 -0.371 -0.154 -0.154 -0.154 -0.308  0.500  0.500
            uAS:4:
umfldAnmlSh
zeit2
zeit3
zeit4
zeit5
freigngIndr
umfldAShl:2
umfldAShl:3
umfldAShl:4
umfldAShl:5
umfldAShl:I
zt2:frgngIn
zt3:frgngIn
zt4:frgngIn
zt5:frgngIn
umfldAS:2:I
umfldAS:3:I
umfldAS:4:I
umfldAS:5:I  0.500

Robustness weights for the residuals:
 346 weights are ~= 1. The remaining 64 ones are summarized as
   Min. 1st Qu.  Median    Mean 3rd Qu.    Max.
  0.456   0.584   0.797   0.760   0.926   0.998

Robustness weights for the random effects:
 72 weights are ~= 1. The remaining 10 ones are
    5     6     8    15    20    38    63    66    79    82
0.741 0.897 0.842 0.779 0.870 0.893 0.931 0.949 0.929 0.889

Rho functions used for fitting:
  Residuals:
    eff: smoothed Huber (k = 1.345, s = 10)
    sig: smoothed Huber, Proposal 2 (k = 1.345, s = 10)
  Random Effects, variance component 1 (versuch):
    eff: smoothed Huber (k = 1.345, s = 10)
    vcp: smoothed Huber, Proposal 2 (k = 1.345, s = 10)

ply_rlmm2 <- rlmer(exp ~ umfeld*zeit*freigang + (1|versuch), data = cat_l)
summary(ply_rlmm2)


Robust linear mixed model fit by DAStau
Formula: exp ~ umfeld * zeit * freigang + (1 | versuch)
   Data: cat_l

Scaled residuals:
     Min       1Q   Median       3Q      Max
-2.94635 -0.51786 -0.02029  0.55644  2.79022

Random effects:
 Groups   Name        Variance Std.Dev.
 versuch  (Intercept) 2127.7   46.13
 Residual              810.7   28.47
Number of obs: 410, groups: versuch, 82

Fixed effects:
                                          Estimate Std. Error t value
(Intercept)                                 50.227     10.150   4.948
umfeldAnimal Shelter                        43.201     18.461   2.340
zeit2                                        2.166      7.540   0.287
zeit3                                       -2.836      7.540  -0.376
zeit4                                       -0.486      7.540  -0.064
zeit5                                        3.503      7.540   0.465
freigangIndoor                              37.099     13.659   2.716
umfeldAnimal Shelter:zeit2                 -15.049     13.713  -1.097
umfeldAnimal Shelter:zeit3                   7.067     13.713   0.515
umfeldAnimal Shelter:zeit4                   1.313     13.713   0.096
umfeldAnimal Shelter:zeit5                  -4.274     13.713  -0.312
umfeldAnimal Shelter:freigangIndoor        -91.526     44.383  -2.062
zeit2:freigangIndoor                        -1.664     10.146  -0.164
zeit3:freigangIndoor                       -19.972     10.146  -1.968
zeit4:freigangIndoor                       -23.747     10.146  -2.340
zeit5:freigangIndoor                       -25.591     10.146  -2.522
umfeldAnimal Shelter:zeit2:freigangIndoor   48.048     32.968   1.457
umfeldAnimal Shelter:zeit3:freigangIndoor   38.242     32.968   1.160
umfeldAnimal Shelter:zeit4:freigangIndoor   16.421     32.968   0.498
umfeldAnimal Shelter:zeit5:freigangIndoor   84.863     32.968   2.574

Correlation of Fixed Effects:
            (Intr) umflAS zeit2  zeit3  zeit4  zeit5  frgngI umAS:2 umAS:3
umfldAnmlSh -0.550
zeit2       -0.371  0.204
zeit3       -0.371  0.204  0.500
zeit4       -0.371  0.204  0.500  0.500
zeit5       -0.371  0.204  0.500  0.500  0.500
freigngIndr -0.743  0.409  0.276  0.276  0.276  0.276
umfldAShl:2  0.204 -0.371 -0.550 -0.275 -0.275 -0.275 -0.152
umfldAShl:3  0.204 -0.371 -0.275 -0.550 -0.275 -0.275 -0.152  0.500
umfldAShl:4  0.204 -0.371 -0.275 -0.275 -0.550 -0.275 -0.152  0.500  0.500
umfldAShl:5  0.204 -0.371 -0.275 -0.275 -0.275 -0.550 -0.152  0.500  0.500
umfldAShl:I  0.229 -0.416 -0.085 -0.085 -0.085 -0.085 -0.308  0.154  0.154
zt2:frgngIn  0.276 -0.152 -0.743 -0.372 -0.372 -0.372 -0.371  0.409  0.204
zt3:frgngIn  0.276 -0.152 -0.372 -0.743 -0.372 -0.372 -0.371  0.204  0.409
zt4:frgngIn  0.276 -0.152 -0.372 -0.372 -0.743 -0.372 -0.371  0.204  0.204
zt5:frgngIn  0.276 -0.152 -0.372 -0.372 -0.372 -0.743 -0.371  0.204  0.204
umfldAS:2:I -0.085  0.154  0.229  0.114  0.114  0.114  0.114 -0.416 -0.208
umfldAS:3:I -0.085  0.154  0.114  0.229  0.114  0.114  0.114 -0.208 -0.416
umfldAS:4:I -0.085  0.154  0.114  0.114  0.229  0.114  0.114 -0.208 -0.208
umfldAS:5:I -0.085  0.154  0.114  0.114  0.114  0.229  0.114 -0.208 -0.208
            umAS:4 umAS:5 umAS:I zt2:fI zt3:fI zt4:fI zt5:fI uAS:2: uAS:3:
umfldAnmlSh
zeit2
zeit3
zeit4
zeit5
freigngIndr
umfldAShl:2
umfldAShl:3
umfldAShl:4
umfldAShl:5  0.500
umfldAShl:I  0.154  0.154
zt2:frgngIn  0.204  0.204  0.114
zt3:frgngIn  0.204  0.204  0.114  0.500
zt4:frgngIn  0.409  0.204  0.114  0.500  0.500
zt5:frgngIn  0.204  0.409  0.114  0.500  0.500  0.500
umfldAS:2:I -0.208 -0.208 -0.371 -0.308 -0.154 -0.154 -0.154
umfldAS:3:I -0.208 -0.208 -0.371 -0.154 -0.308 -0.154 -0.154  0.500
umfldAS:4:I -0.416 -0.208 -0.371 -0.154 -0.154 -0.308 -0.154  0.500  0.500
umfldAS:5:I -0.208 -0.416 -0.371 -0.154 -0.154 -0.154 -0.308  0.500  0.500
            uAS:4:
umfldAnmlSh
zeit2
zeit3
zeit4
zeit5
freigngIndr
umfldAShl:2
umfldAShl:3
umfldAShl:4
umfldAShl:5
umfldAShl:I
zt2:frgngIn
zt3:frgngIn
zt4:frgngIn
zt5:frgngIn
umfldAS:2:I
umfldAS:3:I
umfldAS:4:I
umfldAS:5:I  0.500

Robustness weights for the residuals:
 346 weights are ~= 1. The remaining 64 ones are summarized as
   Min. 1st Qu.  Median    Mean 3rd Qu.    Max.
  0.456   0.584   0.797   0.760   0.926   0.998

Robustness weights for the random effects:
 72 weights are ~= 1. The remaining 10 ones are
    5     6     8    15    20    38    63    66    79    82
0.741 0.897 0.842 0.779 0.870 0.893 0.931 0.949 0.929 0.889

Rho functions used for fitting:
  Residuals:
    eff: smoothed Huber (k = 1.345, s = 10)
    sig: smoothed Huber, Proposal 2 (k = 1.345, s = 10)
  Random Effects, variance component 1 (versuch):
    eff: smoothed Huber (k = 1.345, s = 10)
    vcp: smoothed Huber, Proposal 2 (k = 1.345, s = 10)
```

Effektstärke

```
##Cohen's d berechnen


reffects_ply_frei<-broom.mixed::tidy(ply_lmm_frei, effects = c("ran_pars"), scale="vcov")


md_ply_frei <-summary(ply_lmm_frei)$coef[2] #Md = Mittelwertsdifferenz
sdPart_ply_frei<-sqrt(sum(reffects_ply$estimate)) #Random-Effects zusammenzählen

##Cohen´s d für alle Faktoren



cohend_ply_frei <- data.frame(cbind(summary(ply_lmm_frei)$coef[, 1]/sdPart_ply)[c(2,3,4,5,6,7,8,9,10),])
colnames(cohend_ply_frei) <- "CohensD_ply_f"
```

Grafik

```
#estimated marginal means
# geschätzen Randmittel


# Grafik auf Basis der LMM nach Umfeld

em_df_ply_f1<- data.frame(emmeans(ply_lmm_frei, "umfeld", by=c("zeit","freigang")))
em_df_ply_f2<- data.frame(emmeans(ply_lmm_frei, "umfeld", by=c("zeit")))
em_df_ply_f3<- data.frame(emmeans(ply_lmm_frei, "freigang", by=c("zeit")))

ggplot(em_df_ply_f1, aes(x = zeit, y = emmean, group=umfeld, color=umfeld))+
  geom_line() +
  geom_errorbar(aes(ymin=lower.CL, ymax=upper.CL), width=.4,
                position=position_dodge(0.01)) +
  geom_point(aes(shape=umfeld))+
  scale_colour_manual(values=c("#D700FF","#00C0FA"))+
  scale_y_continuous(limits=c(0,20),breaks=seq(0,20,2)) +
  theme_bw() +
  labs(x="Video Sequence (No)", y = "Play Behaviour (sec)",color="Living Environment",shape="Living Environment")



# log-transformierte unabhängige Variable - muss zurück transformiert werden für die Grafik
# First transform the values back to seconds
em_df_ply_f1$emmean <- exp(em_df_ply_f1$emmean) - 1
em_df_ply_f1$lower.CL <- exp(em_df_ply_f1$lower.CL) - 1
em_df_ply_f1$upper.CL <- exp(em_df_ply_f1$upper.CL) - 1

# Create the plot with transformed values
ply_f1 <- ggplot(em_df_ply_f1, aes(x = zeit, y = emmean, group=umfeld, color=umfeld))+
  geom_line() +
  geom_errorbar(aes(ymin=lower.CL, ymax=upper.CL), width=.4,
                position=position_dodge(0.01)) +
  geom_point(aes(shape=umfeld))+
  scale_colour_manual(values=c("#D700FF","#00C0FA"))+
  scale_y_continuous(limits=c(0,20), breaks=seq(0,20,2)) +
  theme_bw() +
  labs(x="Video Sequence (No)", y = "Play Behaviour (sec)",color="Living Environment",shape="Living Environment")


ply_f1



# Grafik auf Basiis der LMM nach Freigang


ggplot(em_df_ply_f3, aes(x = zeit, y = emmean, group=freigang, color=freigang))+
  geom_line() +
  geom_errorbar(aes(ymin=lower.CL, ymax=upper.CL), width=.4,
                position=position_dodge(0.01)) +
  geom_point(aes(shape=freigang))+
  scale_colour_manual(values=c("#D700FF","#00C0FA"))+
  scale_y_continuous(limits=c(0,120),breaks=seq(0,120,10)) +
  theme_bw() +
  labs(x="Video Sequence (No)", y = "Play Behaviour (sec)",color= "Outdoor Access",shape="Outdoor Access")



# log-transformierte unabhängige Variable - muss zurück transformiert werden für die Grafik
# First transform the values back to seconds
em_df_ply_f3$emmean <- exp(em_df_ply_f3$emmean) - 1
em_df_ply_f3$lower.CL <- exp(em_df_ply_f3$lower.CL) - 1
em_df_ply_f3$upper.CL <- exp(em_df_ply_f3$upper.CL) - 1

# Create the plot with transformed values
ply_f2 <- ggplot(em_df_ply_f3, aes(x = zeit, y = emmean, group=freigang, color=freigang))+
  geom_line() +
  geom_errorbar(aes(ymin=lower.CL, ymax=upper.CL), width=.4,
                position=position_dodge(0.01)) +
  geom_point(aes(shape=freigang))+
  scale_colour_manual(values=c("#D700FF","#00C0FA"))+
  scale_y_continuous(limits=c(0,120), breaks=seq(0,120,10)) +
  theme_bw() +
  labs(x="Video Sequence (No)", y = "Play Behaviour (sec)",color="Outdoor Access",shape="Outdoor Access")

ply_f2
```

Paarweise Vergleiche

```
pairs(emmeans(ply_lmm_frei, "umfeld", by="zeit") , adjust="dunnet")

zeit = 1:
 contrast                       estimate    SE  df t.ratio p.value
 Private Home - Animal Shelter     1.140 0.759 153   1.503  0.1348

zeit = 2:
 contrast                       estimate    SE  df t.ratio p.value
 Private Home - Animal Shelter     0.787 0.759 153   1.038  0.3011

zeit = 3:
 contrast                       estimate    SE  df t.ratio p.value
 Private Home - Animal Shelter     0.759 0.759 153   1.001  0.3184

zeit = 4:
 contrast                       estimate    SE  df t.ratio p.value
 Private Home - Animal Shelter     1.408 0.891 242   1.580  0.1155

zeit = 5:
 contrast                       estimate    SE  df t.ratio p.value
 Private Home - Animal Shelter     0.419 0.759 153   0.552  0.5818

Results are averaged over the levels of: freigang
Note: contrasts are still on the log(mu + 1) scale. Consider using
      regrid() if you want contrasts of back-transformed estimates.
Degrees-of-freedom method: kenward-roger

pairs(emmeans(ply_lmm_frei, "zeit", by="umfeld") , adjust="dunnet")

umfeld = Private Home:
 contrast      estimate    SE  df t.ratio p.value
 zeit1 - zeit2   0.2565 0.204 308   1.260  0.7350
 zeit1 - zeit3  -0.2102 0.204 308  -1.032  0.8535
 zeit1 - zeit4  -0.5002 0.204 308  -2.457  0.1068
 zeit1 - zeit5   0.0618 0.204 308   0.304  0.9983
 zeit2 - zeit3  -0.4667 0.204 308  -2.292  0.1557
 zeit2 - zeit4  -0.7567 0.204 308  -3.716  0.0022
 zeit2 - zeit5  -0.1946 0.204 308  -0.956  0.8855
 zeit3 - zeit4  -0.2900 0.204 308  -1.424  0.6328
 zeit3 - zeit5   0.2720 0.204 308   1.336  0.6887
 zeit4 - zeit5   0.5620 0.204 308   2.760  0.0490

umfeld = Animal Shelter:
 contrast      estimate    SE  df t.ratio p.value
 zeit1 - zeit2  -0.0968 0.630 308  -0.154  0.9999
 zeit1 - zeit3  -0.5913 0.630 308  -0.939  0.8919
 zeit1 - zeit4  -0.2325 0.784 314  -0.296  0.9985
 zeit1 - zeit5  -0.6599 0.630 308  -1.048  0.8463
 zeit2 - zeit3  -0.4945 0.630 308  -0.785  0.9409
 zeit2 - zeit4  -0.1357 0.784 314  -0.173  0.9998
 zeit2 - zeit5  -0.5631 0.630 308  -0.894  0.9080
 zeit3 - zeit4   0.3587 0.784 314   0.457  0.9919
 zeit3 - zeit5  -0.0686 0.630 308  -0.109  1.0000
 zeit4 - zeit5  -0.4273 0.784 314  -0.545  0.9842

Results are averaged over the levels of: freigang
Note: contrasts are still on the log(mu + 1) scale. Consider using
      regrid() if you want contrasts of back-transformed estimates.
Degrees-of-freedom method: kenward-roger
P value adjustment: dunnettx method for 10 tests

pairs(emmeans(ply_lmm_frei, "zeit", by="freigang") , adjust="dunnet")

freigang = Outdoor:
 contrast      estimate    SE  df t.ratio p.value
 zeit1 - zeit2 -0.02967 0.275 308  -0.108  1.0000
 zeit1 - zeit3 -0.26114 0.275 308  -0.949  0.8882
 zeit1 - zeit4 -0.26008 0.292 310  -0.890  0.9095
 zeit1 - zeit5  0.29440 0.275 308   1.070  0.8363
 zeit2 - zeit3 -0.23147 0.275 308  -0.841  0.9252
 zeit2 - zeit4 -0.23041 0.292 310  -0.788  0.9401
 zeit2 - zeit5  0.32407 0.275 308   1.178  0.7814
 zeit3 - zeit4  0.00106 0.292 310   0.004  1.0000
 zeit3 - zeit5  0.55554 0.275 308   2.019  0.2700
 zeit4 - zeit5  0.55448 0.292 310   1.897  0.3341

freigang = Indoor:
 contrast      estimate    SE  df t.ratio p.value
 zeit1 - zeit2  0.18934 0.602 308   0.315  0.9981
 zeit1 - zeit3 -0.54032 0.602 308  -0.898  0.9068
 zeit1 - zeit4 -0.47266 0.756 314  -0.625  0.9738
 zeit1 - zeit5 -0.89242 0.602 308  -1.483  0.5944
 zeit2 - zeit3 -0.72966 0.602 308  -1.213  0.7619
 zeit2 - zeit4 -0.66200 0.756 314  -0.876  0.9142
 zeit2 - zeit5 -1.08176 0.602 308  -1.798  0.3922
 zeit3 - zeit4  0.06765 0.756 314   0.090  1.0000
 zeit3 - zeit5 -0.35210 0.602 308  -0.585  0.9795
 zeit4 - zeit5 -0.41976 0.756 314  -0.555  0.9831

Results are averaged over the levels of: umfeld
Note: contrasts are still on the log(mu + 1) scale. Consider using
      regrid() if you want contrasts of back-transformed estimates.
Degrees-of-freedom method: kenward-roger
P value adjustment: dunnettx method for 10 tests

pairs(emmeans(ply_lmm_frei, "freigang", by="umfeld") , adjust="dunnet")

umfeld = Private Home:
 contrast         estimate    SE   df t.ratio p.value
 Outdoor - Indoor   -0.426 0.389 77.8  -1.094  0.2773

umfeld = Animal Shelter:
 contrast         estimate    SE   df t.ratio p.value
 Outdoor - Indoor    0.151 1.218 81.5   0.124  0.9016

Results are averaged over the levels of: zeit
Note: contrasts are still on the log(mu + 1) scale. Consider using
      regrid() if you want contrasts of back-transformed estimates.
Degrees-of-freedom method: kenward-roger

confint(pairs(emmeans(ply_lmm_frei,~umfeld)))

 contrast                       estimate   SE   df lower.CL upper.CL
 Private Home - Animal Shelter     0.903 0.64 81.1    -0.37     2.18

Results are averaged over the levels of: zeit, freigang
Note: contrasts are still on the log(mu + 1) scale. Consider using
      regrid() if you want contrasts of back-transformed estimates.
Degrees-of-freedom method: kenward-roger
Confidence level used: 0.95

confint(pairs(emmeans(ply_lmm_frei,~zeit)))

 contrast      estimate    SE  df lower.CL upper.CL
 zeit1 - zeit2   0.0798 0.331 308   -0.828    0.988
 zeit1 - zeit3  -0.4007 0.331 308   -1.308    0.507
 zeit1 - zeit4  -0.3664 0.405 313   -1.478    0.745
 zeit1 - zeit5  -0.2990 0.331 308   -1.207    0.609
 zeit2 - zeit3  -0.4806 0.331 308   -1.388    0.427
 zeit2 - zeit4  -0.4462 0.405 313   -1.558    0.666
 zeit2 - zeit5  -0.3788 0.331 308   -1.287    0.529
 zeit3 - zeit4   0.0344 0.405 313   -1.077    1.146
 zeit3 - zeit5   0.1017 0.331 308   -0.806    1.009
 zeit4 - zeit5   0.0674 0.405 313   -1.044    1.179

Results are averaged over the levels of: umfeld, freigang
Note: contrasts are still on the log(mu + 1) scale. Consider using
      regrid() if you want contrasts of back-transformed estimates.
Degrees-of-freedom method: kenward-roger
Confidence level used: 0.95
Conf-level adjustment: tukey method for comparing a family of 5 estimates

confint(pairs(emmeans(ply_lmm_frei,~freigang)))

 contrast         estimate   SE   df lower.CL upper.CL
 Outdoor - Indoor   -0.138 0.64 81.1    -1.41     1.13

Results are averaged over the levels of: umfeld, zeit
Note: contrasts are still on the log(mu + 1) scale. Consider using
      regrid() if you want contrasts of back-transformed estimates.
Degrees-of-freedom method: kenward-roger
Confidence level used: 0.95
```

Residuen

```
ply_diagnose_frei <- broom.mixed::augment(ply_lmm_frei)

#Normalverteiung - annähernd normalverteilt

ply_r1_frei <- ggplot(ply_diagnose_frei, aes(x = .resid)) +
geom_histogram(aes(x = .resid, y = ..density..)) +
geom_density(aes(colour = "density estimate")) +
geom_function(aes(colour = "normal curve"), fun = dnorm,

args = list(mean = mean(ply_diagnose_frei$.resid),sd = sd(ply_diagnose_frei$.resid))) +

scale_colour_discrete(name = "Curve") +

theme(legend.position = "top")


ply_r2_frei <- ggplot(ply_diagnose_frei, aes(sample = .resid)) +

geom_qq() +

geom_qq_line()

ply_r1_frei + ply_r2_frei



#DHARMa - prüft automatisch Residuen auf Normalverteilung & Abweichungen

q_unit_frei <- simulateResiduals(ply_lmm_frei)

plot(q_unit_frei)



#Homoskedastizität

ply_r3_frei <- ggplot(ply_diagnose_frei, aes(x = .fitted, y = .resid)) + geom_point()


ply_r4_frei <- ggplot(ply_diagnose_frei, aes(x = seq_along(.resid), y = .resid)) +

geom_point() + geom_smooth() + xlab("Index")


ply_r3_frei + ply_r4_frei



###Ausreißer kontrollieren

#standardisierte Residuen ansehen

# cutoff aus Field für Ausreißer von Regression auf LMM
#ply_cutoff <- 3*ply_lmm$rank/(ply_lmm$rank+ply_lmm$df.residual)
#  geom_hline(yintercept = m4_cutoff, linetype = "dashed", color = "darkgreen") +

# bei allen einfügen: label = as.numeric(rownames(ply_diagnose))

favstats(scale(ply_diagnose_frei$.resid))

       min         Q1      median        Q3      max          mean sd   n
 -3.596607 -0.4929517 -0.06952486 0.4797522 2.965986 2.214977e-17  1 406
 missing
       0

ply_r5_frei <- ggplot(ply_diagnose_frei, aes(y = .hat, label = as.numeric(rownames(ply_diagnose_frei)))) +

geom_boxplot() +

ggrepel::geom_text_repel(aes(x = 0), max.overlaps = 30)


ply_r6_frei <- ggplot(ply_diagnose_frei, aes(y = .cooksd)) +

geom_boxplot()


ply_r5_frei + ply_r6_frei
```

Passives Verhalten (passiv)

Modelle Umfeld + Zeit

```
#ANOVA rechnen

passiv_m1 <- aov_car(sec ~ umfeld *zeit+ Error(versuch/zeit),
                  data=cat_long %>% dplyr::filter(verhalten=="passiv"),
                  type=3)
summary(passiv_m1)


Univariate Type III Repeated-Measures ANOVA Assuming Sphericity

            Sum Sq num Df Error SS den Df F value    Pr(>F)
(Intercept) 299744      1   634157     80 37.8132 2.891e-08 ***
umfeld       63347      1   634157     80  7.9913  0.005935 **
zeit          8106      4   329310    320  1.9692  0.098958 .
umfeld:zeit   1818      4   329310    320  0.4416  0.778535
---
Signif. codes:  0 '***' 0.001 '**' 0.01 '*' 0.05 '.' 0.1 ' ' 1


Mauchly Tests for Sphericity

            Test statistic p-value
zeit               0.90944 0.59107
umfeld:zeit        0.90944 0.59107


Greenhouse-Geisser and Huynh-Feldt Corrections
 for Departure from Sphericity

             GG eps Pr(>F[GG])
zeit        0.95456     0.1024
umfeld:zeit 0.95456     0.7698

              HF eps Pr(>F[HF])
zeit        1.008367 0.09895824
umfeld:zeit 1.008367 0.77853450

effectsize::omega_squared(passiv_m1)

# Effect Size for ANOVA (Type III)

Parameter   | Omega2 (partial) |       95% CI
---------------------------------------------
umfeld      |             0.08 | [0.01, 1.00]
zeit        |         4.07e-03 | [0.00, 1.00]
umfeld:zeit |             0.00 | [0.00, 1.00]

- One-sided CIs: upper bound fixed at [1.00].

# LMM rechnen

passiv_lmm <- lmer(passiv ~ umfeld*zeit + (1|versuch), data = cat_l)

summary(passiv_lmm)

Linear mixed model fit by REML. t-tests use Satterthwaite's method [
lmerModLmerTest]
Formula: passiv ~ umfeld * zeit + (1 | versuch)
   Data: cat_l

REML criterion at convergence: 4107.6

Scaled residuals:
    Min      1Q  Median      3Q     Max
-2.4355 -0.4898 -0.1383  0.3709  3.2763

Random effects:
 Groups   Name        Variance Std.Dev.
 versuch  (Intercept) 1380     37.14
 Residual             1029     32.08
Number of obs: 410, groups:  versuch, 82

Fixed effects:
                           Estimate Std. Error      df t value Pr(>|t|)
(Intercept)                  43.672      5.996 172.996   7.284  1.1e-11 ***
umfeldAnimal Shelter        -38.738     14.019 172.996  -2.763  0.00634 **
zeit2                         8.761      5.542 320.000   1.581  0.11493
zeit3                        10.910      5.542 320.000   1.969  0.04987 *
zeit4                         7.925      5.542 320.000   1.430  0.15371
zeit5                         9.269      5.542 320.000   1.672  0.09544 .
umfeldAnimal Shelter:zeit2    1.172     12.959 320.000   0.090  0.92799
umfeldAnimal Shelter:zeit3    8.356     12.959 320.000   0.645  0.51950
umfeldAnimal Shelter:zeit4   14.875     12.959 320.000   1.148  0.25189
umfeldAnimal Shelter:zeit5    8.531     12.959 320.000   0.658  0.51079
---
Signif. codes:  0 '***' 0.001 '**' 0.01 '*' 0.05 '.' 0.1 ' ' 1

Correlation of Fixed Effects:
            (Intr) umflAS zeit2  zeit3  zeit4  zeit5  umAS:2 umAS:3 umAS:4
umfldAnmlSh -0.428
zeit2       -0.462  0.198
zeit3       -0.462  0.198  0.500
zeit4       -0.462  0.198  0.500  0.500
zeit5       -0.462  0.198  0.500  0.500  0.500
umfldAShl:2  0.198 -0.462 -0.428 -0.214 -0.214 -0.214
umfldAShl:3  0.198 -0.462 -0.214 -0.428 -0.214 -0.214  0.500
umfldAShl:4  0.198 -0.462 -0.214 -0.214 -0.428 -0.214  0.500  0.500
umfldAShl:5  0.198 -0.462 -0.214 -0.214 -0.214 -0.428  0.500  0.500  0.500

anova(passiv_lmm)

Type III Analysis of Variance Table with Satterthwaite's method
            Sum Sq Mean Sq NumDF DenDF F value   Pr(>F)
umfeld      8223.8  8223.8     1    80  7.9913 0.005935 **
zeit        8106.1  2026.5     4   320  1.9692 0.098958 .
umfeld:zeit 1817.6   454.4     4   320  0.4416 0.778534
---
Signif. codes:  0 '***' 0.001 '**' 0.01 '*' 0.05 '.' 0.1 ' ' 1
```

Robust rechnen

```
# Robust linear mixed model

passiv_rlmm <- rlmer(passiv ~ umfeld*zeit + (1|versuch), data = cat_l)
summary(passiv_rlmm)


Robust linear mixed model fit by DAStau
Formula: passiv ~ umfeld * zeit + (1 | versuch)
   Data: cat_l

Scaled residuals:
    Min      1Q  Median      3Q     Max
-3.8837 -0.5073 -0.1028  0.5075  4.7298

Random effects:
 Groups   Name        Variance Std.Dev.
 versuch  (Intercept) 1246     35.30
 Residual              642     25.34
Number of obs: 410, groups: versuch, 82

Fixed effects:
```

```
Fixed effects:
                           Estimate Std. Error t value
(Intercept)                  38.312      5.444   7.037
umfeldAnimal Shelter        -34.192     12.730  -2.686
zeit2                         9.596      4.490   2.137
zeit3                        10.520      4.490   2.343
zeit4                         7.122      4.490   1.586
zeit5                         8.197      4.490   1.826
umfeldAnimal Shelter:zeit2   -4.567     10.498  -0.435
umfeldAnimal Shelter:zeit3    5.593     10.498   0.533
umfeldAnimal Shelter:zeit4   12.594     10.498   1.200
umfeldAnimal Shelter:zeit5    7.041     10.498   0.671

Correlation of Fixed Effects:
            (Intr) umflAS zeit2  zeit3  zeit4  zeit5  umAS:2 umAS:3 umAS:4
umfldAnmlSh -0.428
zeit2       -0.412  0.176
zeit3       -0.412  0.176  0.500
zeit4       -0.412  0.176  0.500  0.500
zeit5       -0.412  0.176  0.500  0.500  0.500
umfldAShl:2  0.176 -0.412 -0.428 -0.214 -0.214 -0.214
umfldAShl:3  0.176 -0.412 -0.214 -0.428 -0.214 -0.214  0.500
umfldAShl:4  0.176 -0.412 -0.214 -0.214 -0.428 -0.214  0.500  0.500
umfldAShl:5  0.176 -0.412 -0.214 -0.214 -0.214 -0.428  0.500  0.500  0.500

Robustness weights for the residuals:
 328 weights are ~= 1. The remaining 82 ones are summarized as
   Min. 1st Qu.  Median    Mean 3rd Qu.    Max.
  0.284   0.517   0.705   0.687   0.858   0.998

Robustness weights for the random effects:
 72 weights are ~= 1. The remaining 10 ones are
   37    44    65    69    70    71    76    78    81    82
0.424 0.883 0.444 0.982 0.752 0.772 0.529 0.937 0.758 0.366

Rho functions used for fitting:
  Residuals:
    eff: smoothed Huber (k = 1.345, s = 10)
    sig: smoothed Huber, Proposal 2 (k = 1.345, s = 10)
  Random Effects, variance component 1 (versuch):
    eff: smoothed Huber (k = 1.345, s = 10)
    vcp: smoothed Huber, Proposal 2 (k = 1.345, s = 10)
```

Effektstärke

```
##Cohen's d berechnen


reffects_passiv<-broom.mixed::tidy(passiv_lmm, effects = c("ran_pars"), scale="vcov")


md_passiv <-summary(passiv_lmm)$coef[2] #Md = Mittelwertsdifferenz
sdPart_passiv<-sqrt(sum(reffects_passiv$estimate)) #Random-Effects zusammenzählen
##Cohen´s d für alle Faktoren



cohend_passiv <- data.frame(cbind(summary(passiv_lmm)$coef[, 1]/sdPart_passiv)[c(2,3,4,5,6,7,8,9,10),])
colnames(cohend_passiv) <- "CohensD_passiv"
```

Grafik

```
#estimated marginal means
# geschätzen Randmittel

# Grafik auf Basiis der LMM

em_df_passiv_m1<- data.frame(emmeans(passiv_lmm, "umfeld", by="zeit"))

em_df_passiv_m1$lower.CL <- ifelse(em_df_passiv_m1$lower.CL<0,0,em_df_passiv_m1$lower.CL)

passiv_g1 <-ggplot(em_df_passiv_m1, aes(x = zeit, y = emmean, group=umfeld, color=umfeld))+
  geom_line() +
  geom_errorbar(aes(ymin=lower.CL, ymax=upper.CL), width=.4,
              position=position_dodge(0.01)) +
  geom_point(aes(shape=umfeld))+
  scale_colour_manual(values=c("#D700FF","#00C0FA"))+
  scale_y_continuous(limits=c(0,120),breaks=seq(0,120,10)) +
  theme_bw() +
  labs(x="Video Sequence (No)", y = "Passive Behaviour (sec)",color="Living Environment",shape="Living Environment")

passiv_g1
```

Paarweise Vergleiche

```
pairs(emmeans(passiv_lmm, "umfeld", by="zeit") , adjust="dunnet")

zeit = 1:
 contrast                      estimate SE  df t.ratio p.value
 Private Home - Animal Shelter     38.7 14 173   2.763  0.0063

zeit = 2:
 contrast                      estimate SE  df t.ratio p.value
 Private Home - Animal Shelter     37.6 14 173   2.680  0.0081

zeit = 3:
 contrast                      estimate SE  df t.ratio p.value
 Private Home - Animal Shelter     30.4 14 173   2.167  0.0316

zeit = 4:
 contrast                      estimate SE  df t.ratio p.value
 Private Home - Animal Shelter     23.9 14 173   1.702  0.0905

zeit = 5:
 contrast                      estimate SE  df t.ratio p.value
 Private Home - Animal Shelter     30.2 14 173   2.155  0.0326

Degrees-of-freedom method: kenward-roger

pairs(emmeans(passiv_lmm, "zeit", by="umfeld") , adjust="dunnet")

umfeld = Private Home:
 contrast      estimate    SE  df t.ratio p.value
 zeit1 - zeit2   -8.761  5.54 320  -1.581  0.5303
 zeit1 - zeit3  -10.910  5.54 320  -1.969  0.2955
 zeit1 - zeit4   -7.925  5.54 320  -1.430  0.6291
 zeit1 - zeit5   -9.269  5.54 320  -1.672  0.4707
 zeit2 - zeit3   -2.149  5.54 320  -0.388  0.9957
 zeit2 - zeit4    0.836  5.54 320   0.151  0.9999
 zeit2 - zeit5   -0.507  5.54 320  -0.092  1.0000
 zeit3 - zeit4    2.985  5.54 320   0.539  0.9849
 zeit3 - zeit5    1.642  5.54 320   0.296  0.9985
 zeit4 - zeit5   -1.343  5.54 320  -0.242  0.9993

umfeld = Animal Shelter:
 contrast      estimate    SE  df t.ratio p.value
 zeit1 - zeit2   -9.933 11.71 320  -0.848  0.9231
 zeit1 - zeit3  -19.267 11.71 320  -1.645  0.4884
 zeit1 - zeit4  -22.800 11.71 320  -1.946  0.3072
 zeit1 - zeit5  -17.800 11.71 320  -1.520  0.5705
 zeit2 - zeit3   -9.333 11.71 320  -0.797  0.9379
 zeit2 - zeit4  -12.867 11.71 320  -1.098  0.8224
 zeit2 - zeit5   -7.867 11.71 320  -0.672  0.9661
 zeit3 - zeit4   -3.533 11.71 320  -0.302  0.9984
 zeit3 - zeit5    1.467 11.71 320   0.125  0.9999
 zeit4 - zeit5    5.000 11.71 320   0.427  0.9937

Degrees-of-freedom method: kenward-roger
P value adjustment: dunnettx method for 10 tests

confint(pairs(emmeans(passiv_lmm,~umfeld)))

 contrast                      estimate   SE df lower.CL upper.CL
 Private Home - Animal Shelter     32.2 11.4 80     9.52     54.8

Results are averaged over the levels of: zeit
Degrees-of-freedom method: kenward-roger
Confidence level used: 0.95

confint(pairs(emmeans(passiv_lmm,~zeit)))

 contrast      estimate   SE  df lower.CL upper.CL
 zeit1 - zeit2   -9.347 6.48 320    -27.1     8.43
 zeit1 - zeit3  -15.089 6.48 320    -32.9     2.69
 zeit1 - zeit4  -15.363 6.48 320    -33.1     2.41
 zeit1 - zeit5  -13.534 6.48 320    -31.3     4.24
 zeit2 - zeit3   -5.741 6.48 320    -23.5    12.03
 zeit2 - zeit4   -6.015 6.48 320    -23.8    11.76
 zeit2 - zeit5   -4.187 6.48 320    -22.0    13.59
 zeit3 - zeit4   -0.274 6.48 320    -18.0    17.50
 zeit3 - zeit5    1.554 6.48 320    -16.2    19.33
 zeit4 - zeit5    1.828 6.48 320    -15.9    19.60

Results are averaged over the levels of: umfeld
Degrees-of-freedom method: kenward-roger
Confidence level used: 0.95
Conf-level adjustment: tukey method for comparing a family of 5 estimates
```

Residuen

```
passiv_diagnose <- broom.mixed::augment(passiv_lmm)

#Normalverteiung - annähernd normalverteilt

passiv_r1 <- ggplot(exp_diagnose, aes(x = .resid)) +

geom_histogram(aes(x = .resid, y = ..density..)) +

geom_density(aes(colour = "density estimate")) +

geom_function(aes(colour = "normal curve"), fun = dnorm,

args = list(mean = mean(passiv_diagnose$.resid),sd = sd(passiv_diagnose$.resid))) +

scale_colour_discrete(name = "Curve") +

theme(legend.position = "top")


passiv_r2 <- ggplot(passiv_diagnose, aes(sample = .resid)) +

geom_qq() +

geom_qq_line()

passiv_r1 + passiv_r2



#DHARMa - prüft automatisch Residuen auf Normalverteilung & Abweichungen

q_unit <- simulateResiduals(passiv_lmm)

plot(q_unit)



#Homoskedastizität

passiv_r3 <- ggplot(passiv_diagnose, aes(x = .fitted, y = .resid)) + geom_point()


passiv_r4 <- ggplot(passiv_diagnose, aes(x = seq_along(.resid), y = .resid)) +

geom_point() + geom_smooth() + xlab("Index")


passiv_r3 + passiv_r4



###Ausreißer kontrollieren

#standardisierte Residuen ansehen

favstats(scale(passiv_diagnose$.resid))

       min         Q1     median        Q3      max          mean sd   n
 -2.709771 -0.5449877 -0.1539031 0.4127137 3.645367 -3.032804e-17  1 410
 missing
       0

passiv_r5 <- ggplot(passiv_diagnose, aes(y = .hat, label = as.numeric(rownames(passiv_diagnose)))) +
  geom_boxplot() +
  ggrepel::geom_text_repel(aes(x = 0), max.overlaps = 30)


passiv_r6 <- ggplot(passiv_diagnose, aes(y = .cooksd)) + geom_boxplot()


passiv_r5 + passiv_r6



### 2. Variante -->

#p=11 (siehe Anova Befehl)

#The threshold in our sample is 2p/n=2*11/159=0,1383

#threshold berechnen:
#
# passiv_p <- anova(angst_m0, angst_m2)
#
# passiv_npar <- angst_p[2,1]
#
# passiv_n <- nrow(passiv_diagnose)/2 exp_lev_cutoff <- 3*angst_npar/angst_n
#
# passiv_r7 <- ggplot(passiv_diagnose, aes(y = .hat, label = subj_ID)) + geom_boxplot() +
#
# ggrepel::geom_text_repel(aes(x = 0), data =
#
# passiv_diagnose %>% filter(.hat > angst_lev_cutoff), max.overlaps = 30)
#
# passiv_r8 <- ggplot(passiv_diagnose, aes(y = .cooksd, label = subj_ID)) +
#
# geom_boxplot() + ggrepel::geom_text_repel(aes(x = 0))
#
# passiv_r7 + passiv_r8
```

Verhalten an der Tür (tuer)

Modelle Umfeld + Zeit

```
# ANOVA rechnen

tuer_m1 <- aov_car(sec ~ umfeld *zeit+ Error(versuch/zeit),
                 data=cat_long %>% dplyr::filter(verhalten=="tuer"),
                 type=3)
summary(tuer_m1)


Univariate Type III Repeated-Measures ANOVA Assuming Sphericity

            Sum Sq num Df Error SS den Df F value    Pr(>F)
(Intercept)  52156      1   171793     80 24.2876 4.403e-06 ***
umfeld        2265      1   171793     80  1.0550    0.3075
zeit          2636      4   186339    320  1.1315    0.3416
umfeld:zeit   2736      4   186339    320  1.1746    0.3219
---
Signif. codes:  0 '***' 0.001 '**' 0.01 '*' 0.05 '.' 0.1 ' ' 1


Mauchly Tests for Sphericity

            Test statistic   p-value
zeit               0.70844 0.0013868
umfeld:zeit        0.70844 0.0013868


Greenhouse-Geisser and Huynh-Feldt Corrections
 for Departure from Sphericity

             GG eps Pr(>F[GG])
zeit        0.88707     0.3400
umfeld:zeit 0.88707     0.3215

               HF eps Pr(>F[HF])
zeit        0.9332986  0.3407412
umfeld:zeit 0.9332986  0.3217457

effectsize::omega_squared(tuer_m1)

# Effect Size for ANOVA (Type III)

Parameter   | Omega2 (partial) |       95% CI
---------------------------------------------
umfeld      |         6.70e-04 | [0.00, 1.00]
zeit        |         8.44e-04 | [0.00, 1.00]
umfeld:zeit |         1.12e-03 | [0.00, 1.00]

- One-sided CIs: upper bound fixed at [1.00].

# LMM rechnen

tuer_lmm <- lmer(log(tuer+1) ~ umfeld*zeit + (1|versuch), data = cat_l)

summary(tuer_lmm)

Linear mixed model fit by REML. t-tests use Satterthwaite's method [
lmerModLmerTest]
Formula: log(tuer + 1) ~ umfeld * zeit + (1 | versuch)
   Data: cat_l

REML criterion at convergence: 1470.4

Scaled residuals:
    Min      1Q  Median      3Q     Max
-2.7806 -0.5913 -0.0853  0.6642  3.1647

Random effects:
 Groups   Name        Variance Std.Dev.
 versuch  (Intercept) 1.283    1.133
 Residual             1.524    1.235
Number of obs: 410, groups:  versuch, 82

Fixed effects:
                           Estimate Std. Error       df t value Pr(>|t|)
(Intercept)                  1.5817     0.2047 217.8973   7.726 4.01e-13 ***
umfeldAnimal Shelter        -0.6915     0.4787 217.8973  -1.445   0.1500
zeit2                       -0.2381     0.2133 320.0000  -1.116   0.2651
zeit3                        0.6583     0.2133 320.0000   3.086   0.0022 **
zeit4                       -0.4903     0.2133 320.0000  -2.298   0.0222 *
zeit5                       -0.1340     0.2133 320.0000  -0.628   0.5304
umfeldAnimal Shelter:zeit2  -0.1006     0.4988 320.0000  -0.202   0.8402
umfeldAnimal Shelter:zeit3  -0.2549     0.4988 320.0000  -0.511   0.6097
umfeldAnimal Shelter:zeit4   0.2616     0.4988 320.0000   0.524   0.6003
umfeldAnimal Shelter:zeit5   1.0044     0.4988 320.0000   2.014   0.0449 *
---
Signif. codes:  0 '***' 0.001 '**' 0.01 '*' 0.05 '.' 0.1 ' ' 1

Correlation of Fixed Effects:
            (Intr) umflAS zeit2  zeit3  zeit4  zeit5  umAS:2 umAS:3 umAS:4
umfldAnmlSh -0.428
zeit2       -0.521  0.223
zeit3       -0.521  0.223  0.500
zeit4       -0.521  0.223  0.500  0.500
zeit5       -0.521  0.223  0.500  0.500  0.500
umfldAShl:2  0.223 -0.521 -0.428 -0.214 -0.214 -0.214
umfldAShl:3  0.223 -0.521 -0.214 -0.428 -0.214 -0.214  0.500
umfldAShl:4  0.223 -0.521 -0.214 -0.214 -0.428 -0.214  0.500  0.500
umfldAShl:5  0.223 -0.521 -0.214 -0.214 -0.214 -0.428  0.500  0.500  0.500

anova(tuer_lmm)

Type III Analysis of Variance Table with Satterthwaite's method
            Sum Sq Mean Sq NumDF DenDF F value    Pr(>F)
umfeld       3.052  3.0520     1    80  2.0020 0.1609690
zeit        30.260  7.5649     4   320  4.9623 0.0006806 ***
umfeld:zeit 12.091  3.0228     4   320  1.9829 0.0968737 .
---
Signif. codes:  0 '***' 0.001 '**' 0.01 '*' 0.05 '.' 0.1 ' ' 1
```

Robust rechnen

```
# Robust linear mixed model

tuer_rlmm <- rlmer(tuer ~ umfeld*zeit + (1|versuch), data = cat_l)
summary(tuer_rlmm)

Robust linear mixed model fit by DAStau
Formula: tuer ~ umfeld * zeit + (1 | versuch)
   Data: cat_l

Scaled residuals:
    Min      1Q  Median      3Q     Max
-6.2508 -0.5056 -0.0974  0.3398 15.7248

Random effects:
 Groups   Name        Variance Std.Dev.
 versuch  (Intercept) 128.5    11.33
 Residual             139.4    11.81
Number of obs: 410, groups: versuch, 82

Fixed effects:
                           Estimate Std. Error t value
(Intercept)                 10.7528     2.0509   5.243
umfeldAnimal Shelter        -6.1103     4.7951  -1.274
zeit2                       -1.3715     2.0924  -0.655
zeit3                        7.9721     2.0924   3.810
zeit4                       -3.3817     2.0924  -1.616
zeit5                       -0.5126     2.0924  -0.245
umfeldAnimal Shelter:zeit2   1.2251     4.8922   0.250
umfeldAnimal Shelter:zeit3  -6.1650     4.8922  -1.260
umfeldAnimal Shelter:zeit4   1.9496     4.8922   0.399
umfeldAnimal Shelter:zeit5   6.7251     4.8922   1.375

Correlation of Fixed Effects:
            (Intr) umflAS zeit2  zeit3  zeit4  zeit5  umAS:2 umAS:3 umAS:4
umfldAnmlSh -0.428
zeit2       -0.510  0.218
zeit3       -0.510  0.218  0.500
zeit4       -0.510  0.218  0.500  0.500
zeit5       -0.510  0.218  0.500  0.500  0.500
umfldAShl:2  0.218 -0.510 -0.428 -0.214 -0.214 -0.214
umfldAShl:3  0.218 -0.510 -0.214 -0.428 -0.214 -0.214  0.500
umfldAShl:4  0.218 -0.510 -0.214 -0.214 -0.428 -0.214  0.500  0.500
umfldAShl:5  0.218 -0.510 -0.214 -0.214 -0.214 -0.428  0.500  0.500  0.500

Robustness weights for the residuals:
 325 weights are ~= 1. The remaining 85 ones are summarized as
   Min. 1st Qu.  Median    Mean 3rd Qu.    Max.
 0.0855  0.3150  0.5530  0.5810  0.8340  0.9970

Robustness weights for the random effects:
 66 weights are ~= 1. The remaining 16 ones are summarized as
   Min. 1st Qu.  Median    Mean 3rd Qu.    Max.
  0.220   0.519   0.850   0.724   0.922   0.998

Rho functions used for fitting:
  Residuals:
    eff: smoothed Huber (k = 1.345, s = 10)
    sig: smoothed Huber, Proposal 2 (k = 1.345, s = 10)
  Random Effects, variance component 1 (versuch):
    eff: smoothed Huber (k = 1.345, s = 10)
    vcp: smoothed Huber, Proposal 2 (k = 1.345, s = 10)
```

Effektstärke

```
##Cohen's d berechnen


reffects_tuer<-broom.mixed::tidy(tuer_lmm, effects = c("ran_pars"), scale="vcov")


md_tuer <-summary(tuer_lmm)$coef[2] #Md = Mittelwertsdifferenz
sdPart_tuer<-sqrt(sum(reffects_tuer$estimate)) #Random-Effects zusammenzählen
##Cohen´s d für alle Faktoren



cohend_tuer <- data.frame(cbind(summary(tuer_lmm)$coef[, 1]/sdPart_tuer)[c(2,3,4,5,6,7,8,9,10),])
colnames(cohend_tuer) <- "CohensD_tuer"
```

Grafik

```
#estimated marginal means
# geschätzen Randmittel

# Grafik auf Basiis der LMM

em_df_tuer_m1<- data.frame(emmeans(tuer_lmm, "umfeld", by="zeit"))

ggplot(em_df_tuer_m1, aes(x = zeit, y = emmean, group=umfeld, color=umfeld))+
  geom_line() +
  geom_errorbar(aes(ymin=lower.CL, ymax=upper.CL), width=.4,
              position=position_dodge(0.01)) +
  geom_point(aes(shape=umfeld))+
  scale_colour_manual(values=c("#D700FF","#00C0FA"))+
  scale_y_continuous(limits=c(0,20),breaks=seq(0,20,2)) +
  theme_bw() +
  labs(x="Video Sequence (No)", y = "Behaviour by the Door (sec)",color="Living Environment",shape="Living Environment")



# log-transformierte unabhängige Variable - muss zurück transformiert werden für die Grafik
# First transform the values back to seconds
em_df_tuer_m1$emmean <- exp(em_df_tuer_m1$emmean) - 1
em_df_tuer_m1$lower.CL <- exp(em_df_tuer_m1$lower.CL) - 1
em_df_tuer_m1$upper.CL <- exp(em_df_tuer_m1$upper.CL) - 1

# Create the plot with transformed values
tuer_g1 <- ggplot(em_df_tuer_m1, aes(x = zeit, y = emmean, group=umfeld, color=umfeld))+
  geom_line() +
  geom_errorbar(aes(ymin=lower.CL, ymax=upper.CL), width=.4,
              position=position_dodge(0.01)) +
  geom_point(aes(shape=umfeld))+
  scale_colour_manual(values=c("#D700FF","#00C0FA"))+
  scale_y_continuous(limits=c(0,20), breaks=seq(0,20,2)) +
  theme_bw() +
  labs(x="Video Sequence (No)", y = "Behaviour by the Door (sec)",color="Living Environment",shape="Living Environment")

tuer_g1
```

Paarweise Vergleiche

```
pairs(emmeans(tuer_lmm, "umfeld", by="zeit") , adjust="dunnet")

zeit = 1:
 contrast                      estimate    SE  df t.ratio p.value
 Private Home - Animal Shelter    0.691 0.479 218   1.445  0.1500

zeit = 2:
 contrast                      estimate    SE  df t.ratio p.value
 Private Home - Animal Shelter    0.792 0.479 218   1.655  0.0994

zeit = 3:
 contrast                      estimate    SE  df t.ratio p.value
 Private Home - Animal Shelter    0.946 0.479 218   1.977  0.0493

zeit = 4:
 contrast                      estimate    SE  df t.ratio p.value
 Private Home - Animal Shelter    0.430 0.479 218   0.898  0.3701

zeit = 5:
 contrast                      estimate    SE  df t.ratio p.value
 Private Home - Animal Shelter   -0.313 0.479 218  -0.654  0.5139

Note: contrasts are still on the log(mu + 1) scale. Consider using
      regrid() if you want contrasts of back-transformed estimates.
Degrees-of-freedom method: kenward-roger

pairs(emmeans(tuer_lmm, "zeit", by="umfeld") , adjust="dunnet")

umfeld = Private Home:
 contrast      estimate    SE  df t.ratio p.value
 zeit1 - zeit2    0.238 0.213 320   1.116  0.8135
 zeit1 - zeit3   -0.658 0.213 320  -3.086  0.0189
 zeit1 - zeit4    0.490 0.213 320   2.298  0.1534
 zeit1 - zeit5    0.134 0.213 320   0.628  0.9734
 zeit2 - zeit3   -0.896 0.213 320  -4.203  0.0003
 zeit2 - zeit4    0.252 0.213 320   1.182  0.7791
 zeit2 - zeit5   -0.104 0.213 320  -0.488  0.9896
 zeit3 - zeit4    1.149 0.213 320   5.384  <.0001
 zeit3 - zeit5    0.792 0.213 320   3.714  0.0022
 zeit4 - zeit5   -0.356 0.213 320  -1.670  0.4721

umfeld = Animal Shelter:
 contrast      estimate    SE  df t.ratio p.value
 zeit1 - zeit2    0.339 0.451 320   0.751  0.9494
 zeit1 - zeit3   -0.403 0.451 320  -0.895  0.9079
 zeit1 - zeit4    0.229 0.451 320   0.507  0.9880
 zeit1 - zeit5   -0.870 0.451 320  -1.931  0.3157
 zeit2 - zeit3   -0.742 0.451 320  -1.646  0.4875
 zeit2 - zeit4   -0.110 0.451 320  -0.244  0.9993
 zeit2 - zeit5   -1.209 0.451 320  -2.682  0.0604
 zeit3 - zeit4    0.632 0.451 320   1.402  0.6470
 zeit3 - zeit5   -0.467 0.451 320  -1.036  0.8520
 zeit4 - zeit5   -1.099 0.451 320  -2.438  0.1115

Note: contrasts are still on the log(mu + 1) scale. Consider using
      regrid() if you want contrasts of back-transformed estimates.
Degrees-of-freedom method: kenward-roger
P value adjustment: dunnettx method for 10 tests

confint(pairs(emmeans(tuer_lmm,~umfeld)))

 contrast                      estimate   SE df lower.CL upper.CL
 Private Home - Animal Shelter    0.509 0.36 80   -0.207     1.23

Results are averaged over the levels of: zeit
Note: contrasts are still on the log(mu + 1) scale. Consider using
      regrid() if you want contrasts of back-transformed estimates.
Degrees-of-freedom method: kenward-roger
Confidence level used: 0.95

confint(pairs(emmeans(tuer_lmm,~zeit)))

 contrast      estimate    SE  df lower.CL upper.CL
 zeit1 - zeit2    0.288 0.249 320   -0.396   0.9726
 zeit1 - zeit3   -0.531 0.249 320   -1.215   0.1533
 zeit1 - zeit4    0.359 0.249 320   -0.325   1.0436
 zeit1 - zeit5   -0.368 0.249 320   -1.052   0.3159
 zeit2 - zeit3   -0.819 0.249 320   -1.504  -0.1352
 zeit2 - zeit4    0.071 0.249 320   -0.613   0.7551
 zeit2 - zeit5   -0.657 0.249 320   -1.341   0.0275
 zeit3 - zeit4    0.890 0.249 320    0.206   1.5745
 zeit3 - zeit5    0.163 0.249 320   -0.521   0.8468
 zeit4 - zeit5   -0.728 0.249 320   -1.412  -0.0435

Results are averaged over the levels of: umfeld
Note: contrasts are still on the log(mu + 1) scale. Consider using
      regrid() if you want contrasts of back-transformed estimates.
Degrees-of-freedom method: kenward-roger
Confidence level used: 0.95
Conf-level adjustment: tukey method for comparing a family of 5 estimates
```

Residuen

```
tuer_diagnose <- broom.mixed::augment(tuer_lmm)

#Normalverteiung - annähernd normalverteilt

tuer_r1 <- ggplot(tuer_diagnose, aes(x = .resid)) +
geom_histogram(aes(x = .resid, y = ..density..)) +
geom_density(aes(colour = "density estimate")) +
geom_function(aes(colour = "normal curve"), fun = dnorm,
args = list(mean = mean(tuer_diagnose$.resid),sd = sd(tuer_diagnose$.resid))) +
scale_colour_discrete(name = "Curve") +
theme(legend.position = "top")


tuer_r2 <- ggplot(tuer_diagnose, aes(sample = .resid)) +
geom_qq() +
geom_qq_line()
tuer_r1 + tuer_r2



#DHARMa - prüft automatisch Residuen auf Normalverteilung & Abweichungen

q_unit <- simulateResiduals(tuer_lmm)

plot(q_unit)



#Homoskedastizität

tuer_r3 <- ggplot(tuer_diagnose, aes(x = .fitted, y = .resid)) + geom_point()

tuer_r4 <- ggplot(tuer_diagnose, aes(x = seq_along(.resid), y = .resid)) +
geom_point() + geom_smooth() + xlab("Index")

tuer_r3 + tuer_r4



###Ausreißer kontrollieren

#standardisierte Residuen ansehen

favstats(scale(tuer_diagnose$.resid))

       min         Q1      median        Q3      max          mean sd   n
 -3.070798 -0.6530077 -0.09423767 0.7335404 3.494945 -3.222355e-17  1 410
 missing
       0

tuer_r5 <- ggplot(tuer_diagnose, aes(y = .hat, label=as.numeric(rownames(tuer_diagnose)))) +
geom_boxplot() +
ggrepel::geom_text_repel(aes(x = 0), max.overlaps = 30)

tuer_r6 <- ggplot(tuer_diagnose, aes(y = .cooksd)) +
geom_boxplot()

tuer_r5 + tuer_r6



favstats(scale(passiv_diagnose$.resid))

       min         Q1     median        Q3      max          mean sd   n
 -2.709771 -0.5449877 -0.1539031 0.4127137 3.645367 -3.032804e-17  1 410
 missing
       0

passiv_r5 <- ggplot(passiv_diagnose, aes(y = .hat, label = as.numeric(rownames(passiv_diagnose)))) +
  geom_boxplot() +
  ggrepel::geom_text_repel(aes(x = 0), max.overlaps = 30)

passiv_r6 <- ggplot(passiv_diagnose, aes(y = .cooksd)) + geom_boxplot()

passiv_r5 + passiv_r6
```

physischer Kontakt (con)

Modelle Umfeld + Zeit

```
# ANOVA rechnen
con_m1 <- aov_car(sec ~ umfeld *zeit+ Error(versuch/zeit),
                 data=cat_long %>% dplyr::filter(verhalten=="con"),
                 type=3)
summary(con_m1)


Univariate Type III Repeated-Measures ANOVA Assuming Sphericity

            Sum Sq num Df Error SS den Df  F value    Pr(>F)
(Intercept) 540107      1   301108     80 143.4982 < 2.2e-16 ***
umfeld       52103      1   301108     80  13.8430 0.0003678 ***
zeit         27804      4   349313    320   6.3677 6.099e-05 ***
umfeld:zeit   7298      4   349313    320   1.6713 0.1563587
---
Signif. codes:  0 '***' 0.001 '**' 0.01 '*' 0.05 '.' 0.1 ' ' 1


Mauchly Tests for Sphericity

            Test statistic   p-value
zeit               0.71904 0.0021544
umfeld:zeit        0.71904 0.0021544


Greenhouse-Geisser and Huynh-Feldt Corrections
 for Departure from Sphericity

             GG eps Pr(>F[GG])
zeit        0.87443  0.0001485 ***
umfeld:zeit 0.87443  0.1649800
---
Signif. codes:  0 '***' 0.001 '**' 0.01 '*' 0.05 '.' 0.1 ' ' 1

              HF eps  Pr(>F[HF])
zeit        0.919307 0.000107981
umfeld:zeit 0.919307 0.161858557

effectsize::omega_squared(con_m1)

# Effect Size for ANOVA (Type III)

Parameter   | Omega2 (partial) |       95% CI
---------------------------------------------
umfeld      |             0.14 | [0.04, 1.00]
zeit        |             0.03 | [0.00, 1.00]
umfeld:zeit |         4.43e-03 | [0.00, 1.00]

- One-sided CIs: upper bound fixed at [1.00].

# LMM rechnen


con_lmm <- lmer(log(con+1) ~ umfeld*zeit + (1|versuch), data = cat_l)

summary(con_lmm)

Linear mixed model fit by REML. t-tests use Satterthwaite's method [
lmerModLmerTest]
Formula: log(con + 1) ~ umfeld * zeit + (1 | versuch)
   Data: cat_l

REML criterion at convergence: 1481.5

Scaled residuals:
    Min      1Q  Median      3Q     Max
-2.6172 -0.5983  0.1635  0.6817  2.0746

Random effects:
 Groups   Name        Variance Std.Dev.
 versuch  (Intercept) 0.9008   0.9491
 Residual             1.6800   1.2961
Number of obs: 410, groups:  versuch, 82

Fixed effects:
                            Estimate Std. Error        df t value Pr(>|t|)
(Intercept)                  3.09729    0.19626 268.93405  15.781  < 2e-16 ***
umfeldAnimal Shelter         0.54937    0.45888 268.93405   1.197  0.23229
zeit2                       -0.17435    0.22394 320.00000  -0.779  0.43680
zeit3                       -0.58312    0.22394 320.00000  -2.604  0.00965 **
zeit4                       -0.76297    0.22394 320.00000  -3.407  0.00074 ***
zeit5                       -0.70312    0.22394 320.00000  -3.140  0.00185 **
umfeldAnimal Shelter:zeit2  -0.22847    0.52359 320.00000  -0.436  0.66287
umfeldAnimal Shelter:zeit3  -0.05151    0.52359 320.00000  -0.098  0.92170
umfeldAnimal Shelter:zeit4  -0.13417    0.52359 320.00000  -0.256  0.79793
umfeldAnimal Shelter:zeit5   0.43502    0.52359 320.00000   0.831  0.40668
---
Signif. codes:  0 '***' 0.001 '**' 0.01 '*' 0.05 '.' 0.1 ' ' 1

Correlation of Fixed Effects:
            (Intr) umflAS zeit2  zeit3  zeit4  zeit5  umAS:2 umAS:3 umAS:4
umfldAnmlSh -0.428
zeit2       -0.571  0.244
zeit3       -0.571  0.244  0.500
zeit4       -0.571  0.244  0.500  0.500
zeit5       -0.571  0.244  0.500  0.500  0.500
umfldAShl:2  0.244 -0.571 -0.428 -0.214 -0.214 -0.214
umfldAShl:3  0.244 -0.571 -0.214 -0.428 -0.214 -0.214  0.500
umfldAShl:4  0.244 -0.571 -0.214 -0.214 -0.428 -0.214  0.500  0.500
umfldAShl:5  0.244 -0.571 -0.214 -0.214 -0.214 -0.428  0.500  0.500  0.500

anova(con_lmm)

Type III Analysis of Variance Table with Satterthwaite's method
             Sum Sq Mean Sq NumDF DenDF F value  Pr(>F)
umfeld       5.1009  5.1009     1    80  3.0363 0.08526 .
zeit        19.5722  4.8930     4   320  2.9126 0.02168 *
umfeld:zeit  3.2112  0.8028     4   320  0.4779 0.75199
---
Signif. codes:  0 '***' 0.001 '**' 0.01 '*' 0.05 '.' 0.1 ' ' 1
```

Robust rechnen

```
# Robust linear mixed model

con_rlmm <- rlmer(con ~ umfeld*zeit + (1|versuch), data = cat_l)
summary(con_rlmm)

Robust linear mixed model fit by DAStau
Formula: con ~ umfeld * zeit + (1 | versuch)
   Data: cat_l

Scaled residuals:
    Min      1Q  Median      3Q     Max
-2.9270 -0.5604 -0.1702  0.5752  5.0044

Random effects:
 Groups   Name        Variance Std.Dev.
 versuch  (Intercept) 391.7    19.79
 Residual             759.3    27.56
Number of obs: 410, groups: versuch, 82

Fixed effects:
                           Estimate Std. Error t value
(Intercept)                  36.540      4.251   8.596
umfeldAnimal Shelter         39.964      9.939   4.021
zeit2                        -3.779      4.883  -0.774
zeit3                       -11.830      4.883  -2.423
zeit4                       -11.915      4.883  -2.440
zeit5                       -10.352      4.883  -2.120
umfeldAnimal Shelter:zeit2  -15.263     11.417  -1.337
umfeldAnimal Shelter:zeit3  -18.793     11.417  -1.646
umfeldAnimal Shelter:zeit4  -33.507     11.417  -2.935
umfeldAnimal Shelter:zeit5  -15.958     11.417  -1.398

Correlation of Fixed Effects:
            (Intr) umflAS zeit2  zeit3  zeit4  zeit5  umAS:2 umAS:3 umAS:4
umfldAnmlSh -0.428
zeit2       -0.574  0.246
zeit3       -0.574  0.246  0.500
zeit4       -0.574  0.246  0.500  0.500
zeit5       -0.574  0.246  0.500  0.500  0.500
umfldAShl:2  0.246 -0.574 -0.428 -0.214 -0.214 -0.214
umfldAShl:3  0.246 -0.574 -0.214 -0.428 -0.214 -0.214  0.500
umfldAShl:4  0.246 -0.574 -0.214 -0.214 -0.428 -0.214  0.500  0.500
umfldAShl:5  0.246 -0.574 -0.214 -0.214 -0.214 -0.428  0.500  0.500  0.500

Robustness weights for the residuals:
 332 weights are ~= 1. The remaining 78 ones are summarized as
   Min. 1st Qu.  Median    Mean 3rd Qu.    Max.
  0.269   0.568   0.734   0.721   0.926   0.999

Robustness weights for the random effects:
 71 weights are ~= 1. The remaining 11 ones are summarized as
   Min. 1st Qu.  Median    Mean 3rd Qu.    Max.
  0.320   0.578   0.712   0.687   0.807   0.979

Rho functions used for fitting:
  Residuals:
    eff: smoothed Huber (k = 1.345, s = 10)
    sig: smoothed Huber, Proposal 2 (k = 1.345, s = 10)
  Random Effects, variance component 1 (versuch):
    eff: smoothed Huber (k = 1.345, s = 10)
    vcp: smoothed Huber, Proposal 2 (k = 1.345, s = 10)

con_rlmm2 <- rlmer(con ~ umfeld*zeit*freigang + (1|versuch), data = cat_l)
summary(con_rlmm2)

Robust linear mixed model fit by DAStau
Formula: con ~ umfeld * zeit * freigang + (1 | versuch)
   Data: cat_l

Scaled residuals:
    Min      1Q  Median      3Q     Max
-2.7681 -0.5437 -0.1291  0.5613  4.9048

Random effects:
 Groups   Name        Variance Std.Dev.
 versuch  (Intercept) 307.9    17.55
 Residual             759.0    27.55
Number of obs: 410, groups: versuch, 82

Fixed effects:
                                          Estimate Std. Error t value
(Intercept)                                47.6270     6.1163   7.787
umfeldAnimal Shelter                       17.4185    11.1237   1.566
zeit2                                      -8.1447     7.2957  -1.116
zeit3                                     -11.6610     7.2957  -1.598
zeit4                                     -13.6706     7.2957  -1.874
zeit5                                     -18.4529     7.2957  -2.529
freigangIndoor                            -19.4091     8.2305  -2.358
umfeldAnimal Shelter:zeit2                 -5.1995    13.2687  -0.392
umfeldAnimal Shelter:zeit3                -17.8945    13.2687  -1.349
umfeldAnimal Shelter:zeit4                -25.1418    13.2687  -1.895
umfeldAnimal Shelter:zeit5                 -4.8877    13.2687  -0.368
umfeldAnimal Shelter:freigangIndoor        92.3636    26.7433   3.454
zeit2:freigangIndoor                        7.0633     9.8176   0.719
zeit3:freigangIndoor                       -0.3473     9.8176  -0.035
zeit4:freigangIndoor                        2.6558     9.8176   0.271
zeit5:freigangIndoor                       13.2160     9.8176   1.346
umfeldAnimal Shelter:zeit2:freigangIndoor -53.2191    31.9003  -1.668
umfeldAnimal Shelter:zeit3:freigangIndoor  -7.5972    31.9003  -0.238
umfeldAnimal Shelter:zeit4:freigangIndoor -38.3434    31.9003  -1.202
umfeldAnimal Shelter:zeit5:freigangIndoor  -7.3755    31.9003  -0.231

Correlation of Fixed Effects:
            (Intr) umflAS zeit2  zeit3  zeit4  zeit5  frgngI umAS:2 umAS:3
umfldAnmlSh -0.550
zeit2       -0.596  0.328
zeit3       -0.596  0.328  0.500
zeit4       -0.596  0.328  0.500  0.500
zeit5       -0.596  0.328  0.500  0.500  0.500
freigngIndr -0.743  0.409  0.443  0.443  0.443  0.443
umfldAShl:2  0.328 -0.596 -0.550 -0.275 -0.275 -0.275 -0.244
umfldAShl:3  0.328 -0.596 -0.275 -0.550 -0.275 -0.275 -0.244  0.500
umfldAShl:4  0.328 -0.596 -0.275 -0.275 -0.550 -0.275 -0.244  0.500  0.500
umfldAShl:5  0.328 -0.596 -0.275 -0.275 -0.275 -0.550 -0.244  0.500  0.500
umfldAShl:I  0.229 -0.416 -0.136 -0.136 -0.136 -0.136 -0.308  0.248  0.248
```

```
[illegible]
zt2:frgngIn  0.443 -0.244 -0.743 -0.372 -0.372 -0.372 -0.596  0.409  0.204
zt3:frgngIn  0.443 -0.244 -0.372 -0.743 -0.372 -0.372 -0.596  0.204  0.409
zt4:frgngIn  0.443 -0.244 -0.372 -0.372 -0.743 -0.372 -0.596  0.204  0.204
zt5:frgngIn  0.443 -0.244 -0.372 -0.372 -0.372 -0.743 -0.596  0.204  0.204
umfldAS:2:I -0.136  0.248  0.229  0.114  0.114  0.114  0.184 -0.416 -0.208
umfldAS:3:I -0.136  0.248  0.114  0.229  0.114  0.114  0.184 -0.208 -0.416
umfldAS:4:I -0.136  0.248  0.114  0.114  0.229  0.114  0.184 -0.208 -0.208
umfldAS:5:I -0.136  0.248  0.114  0.114  0.114  0.229  0.184 -0.208 -0.208
            umAS:4 umAS:5 umAS:I zt2:fI zt3:fI zt4:fI zt5:fI uAS:2: uAS:3:
umfldAnmlSh
zeit2
zeit3
zeit4
zeit5
freigngIndr
umfldAShl:2
umfldAShl:3
umfldAShl:4
umfldAShl:5  0.500
umfldAShl:I  0.248  0.248
zt2:frgngIn  0.204  0.204  0.184
zt3:frgngIn  0.204  0.204  0.184  0.500
zt4:frgngIn  0.409  0.204  0.184  0.500  0.500
zt5:frgngIn  0.204  0.409  0.184  0.500  0.500  0.500
umfldAS:2:I -0.208 -0.208 -0.596 -0.308 -0.154 -0.154 -0.154
umfldAS:3:I -0.208 -0.208 -0.596 -0.154 -0.308 -0.154 -0.154  0.500
umfldAS:4:I -0.416 -0.208 -0.596 -0.154 -0.154 -0.308 -0.154  0.500  0.500
umfldAS:5:I -0.208 -0.416 -0.596 -0.154 -0.154 -0.154 -0.308  0.500  0.500
            uAS:4:
umfldAnmlSh
zeit2
zeit3
zeit4
zeit5
freigngIndr
umfldAShl:2
umfldAShl:3
umfldAShl:4
umfldAShl:5
umfldAShl:I
zt2:frgngIn
zt3:frgngIn
zt4:frgngIn
zt5:frgngIn
umfldAS:2:I
umfldAS:3:I
umfldAS:4:I
umfldAS:5:I  0.500

Robustness weights for the residuals:
 336 weights are ~= 1. The remaining 74 ones are summarized as
   Min. 1st Qu.  Median    Mean 3rd Qu.    Max.
  0.274   0.552   0.706   0.703   0.905   0.996

Robustness weights for the random effects:
 73 weights are ~= 1. The remaining 9 ones are
    4     5     8    11    13    15    22    63    68
0.409 0.831 0.267 0.930 0.996 0.736 0.619 0.763 0.804

Rho functions used for fitting:
  Residuals:
    eff: smoothed Huber (k = 1.345, s = 10)
    sig: smoothed Huber, Proposal 2 (k = 1.345, s = 10)
  Random Effects, variance component 1 (versuch):
    eff: smoothed Huber (k = 1.345, s = 10)
    vcp: smoothed Huber, Proposal 2 (k = 1.345, s = 10)
```

Effektstärke

```
##Cohen's d berechnen


reffects_con<-broom.mixed::tidy(con_lmm, effects = c("ran_pars"), scale="vcov")


md_con <-summary(con_lmm)$coef[2] #Md = Mittelwertsdifferenz
sdPart_con<-sqrt(sum(reffects_con$estimate)) #Random-Effects zusammenzählen
##Cohen´s d für alle Faktoren



cohend_con <- data.frame(cbind(summary(con_lmm)$coef[, 1]/sdPart_con)[c(2,3,4,5,6,7,8,9,10),])
colnames(cohend_con) <- "CohensD_con"
```

Grafik

```
#estimated marginal means
# geschätzen Randmittel

# Grafik auf Basiis der LMM

em_df_con_m1<- data.frame(emmeans(con_lmm, "umfeld", by="zeit"))

ggplot(em_df_con_m1, aes(x = zeit, y = emmean, group=umfeld, color=umfeld))+
  geom_line() +
  geom_errorbar(aes(ymin=lower.CL, ymax=upper.CL), width=.4,
                position=position_dodge(0.01)) +
  geom_point(aes(shape=umfeld))+
  scale_colour_manual(values=c("#D700FF","#00C0FA"))+
  scale_y_continuous(limits=c(0,20),breaks=seq(0,20,2)) +
  theme_bw() +
  labs(x="Video Sequence (No)", y = "Physical Contact (sec)",color="Living Environment",shape="Living Environment")



# log-transformierte unabhängige Variable - muss zurück transformiert werden für die Grafik
# First transform the values back to seconds
em_df_con_m1$emmean <- exp(em_df_con_m1$emmean) - 1
em_df_con_m1$lower.CL <- exp(em_df_con_m1$lower.CL) - 1
em_df_con_m1$upper.CL <- exp(em_df_con_m1$upper.CL) - 1

# Create the plot with transformed values
con_g1 <- ggplot(em_df_con_m1, aes(x = zeit, y = emmean, group=umfeld, color=umfeld))+
  geom_line() +
  geom_errorbar(aes(ymin=lower.CL, ymax=upper.CL), width=.4,
                position=position_dodge(0.01)) +
  geom_point(aes(shape=umfeld))+
  scale_colour_manual(values=c("#D700FF","#00C0FA"))+
  scale_y_continuous(limits=c(0,120), breaks=seq(0,120,10)) +
  theme_bw() +
  labs(x="Video Sequence (No)", y = "Physical Contact (sec)",color="Living Environment",shape="Living Environment")

con_g1



#"#8CFF00","#F57A00"
```

Paarweise Vergleiche

```
pairs(emmeans(con_lmm, "umfeld", by="zeit") , adjust="dunnet")

zeit = 1:
 contrast                       estimate    SE  df t.ratio p.value
 Private Home - Animal Shelter    -0.549 0.459 269  -1.197  0.2323

zeit = 2:
 contrast                       estimate    SE  df t.ratio p.value
 Private Home - Animal Shelter    -0.321 0.459 269  -0.699  0.4850

zeit = 3:
 contrast                       estimate    SE  df t.ratio p.value
 Private Home - Animal Shelter    -0.498 0.459 269  -1.085  0.2789

zeit = 4:
 contrast                       estimate    SE  df t.ratio p.value
 Private Home - Animal Shelter    -0.415 0.459 269  -0.905  0.3664

zeit = 5:
 contrast                       estimate    SE  df t.ratio p.value
 Private Home - Animal Shelter    -0.984 0.459 269  -2.145  0.0328

Note: contrasts are still on the log(mu + 1) scale. Consider using
      regrid() if you want contrasts of back-transformed estimates.
Degrees-of-freedom method: kenward-roger

pairs(emmeans(con_lmm, "zeit", by="umfeld") , adjust="dunnet")

umfeld = Private Home:
 contrast      estimate    SE  df t.ratio p.value
 zeit1 - zeit2   0.1744 0.224 320   0.779  0.9427
 zeit1 - zeit3   0.5831 0.224 320   2.604  0.0741
 zeit1 - zeit4   0.7630 0.224 320   3.407  0.0067
 zeit1 - zeit5   0.7031 0.224 320   3.140  0.0160
 zeit2 - zeit3   0.4088 0.224 320   1.825  0.3757
 zeit2 - zeit4   0.5886 0.224 320   2.628  0.0695
 zeit2 - zeit5   0.5288 0.224 320   2.361  0.1332
 zeit3 - zeit4   0.1799 0.224 320   0.803  0.9362
 zeit3 - zeit5   0.1200 0.224 320   0.536  0.9852
 zeit4 - zeit5  -0.0599 0.224 320  -0.267  0.9990

umfeld = Animal Shelter:
 contrast      estimate    SE  df t.ratio p.value
 zeit1 - zeit2   0.4028 0.473 320   0.851  0.9221
 zeit1 - zeit3   0.6346 0.473 320   1.341  0.6856
 zeit1 - zeit4   0.8971 0.473 320   1.896  0.3351
 zeit1 - zeit5   0.2681 0.473 320   0.566  0.9818
 zeit2 - zeit3   0.2318 0.473 320   0.490  0.9894
 zeit2 - zeit4   0.4943 0.473 320   1.044  0.8480
 zeit2 - zeit5  -0.1347 0.473 320  -0.285  0.9987
 zeit3 - zeit4   0.2625 0.473 320   0.555  0.9832
 zeit3 - zeit5  -0.3665 0.473 320  -0.774  0.9437
 zeit4 - zeit5  -0.6290 0.473 320  -1.329  0.6930

Note: contrasts are still on the log(mu + 1) scale. Consider using
      regrid() if you want contrasts of back-transformed estimates.
Degrees-of-freedom method: kenward-roger
P value adjustment: dunnettx method for 10 tests

confint(pairs(emmeans(con_lmm,~umfeld)))

 contrast                       estimate    SE df lower.CL upper.CL
 Private Home - Animal Shelter    -0.554 0.318 80    -1.19   0.0786

Results are averaged over the levels of: zeit
Note: contrasts are still on the log(mu + 1) scale. Consider using
      regrid() if you want contrasts of back-transformed estimates.
Degrees-of-freedom method: kenward-roger
Confidence level used: 0.95

confint(pairs(emmeans(con_lmm,~zeit)))

 contrast      estimate    SE  df lower.CL upper.CL
 zeit1 - zeit2    0.289 0.262 320   -0.430    1.007
 zeit1 - zeit3    0.609 0.262 320   -0.109    1.327
 zeit1 - zeit4    0.830 0.262 320    0.112    1.548
 zeit1 - zeit5    0.486 0.262 320   -0.233    1.204
 zeit2 - zeit3    0.320 0.262 320   -0.398    1.038
 zeit2 - zeit4    0.541 0.262 320   -0.177    1.260
 zeit2 - zeit5    0.197 0.262 320   -0.521    0.915
 zeit3 - zeit4    0.221 0.262 320   -0.497    0.939
 zeit3 - zeit5   -0.123 0.262 320   -0.841    0.595
 zeit4 - zeit5   -0.344 0.262 320   -1.063    0.374

Results are averaged over the levels of: umfeld
Note: contrasts are still on the log(mu + 1) scale. Consider using
      regrid() if you want contrasts of back-transformed estimates.
Degrees-of-freedom method: kenward-roger
Confidence level used: 0.95
Conf-level adjustment: tukey method for comparing a family of 5 estimates
```

Residuen

```
con_diagnose <- broom.mixed::augment(con_lmm)

#Normalverteiung - annähernd normalverteilt

con_r1 <- ggplot(con_diagnose, aes(x = .resid)) +

geom_histogram(aes(x = .resid, y = ..density..)) +

geom_density(aes(colour = "density estimate")) +

geom_function(aes(colour = "normal curve"), fun = dnorm,

args = list(mean = mean(con_diagnose$.resid),sd = sd(con_diagnose$.resid))) +

scale_colour_discrete(name = "Curve") +

theme(legend.position = "top")


con_r2 <- ggplot(con_diagnose, aes(sample = .resid)) +

geom_qq() +

geom_qq_line()

con_r1 + con_r2



#DHARMa - prüft automatisch Residuen auf Normalverteilung & Abweichungen

q_unit <- simulateResiduals(con_lmm)

plot(q_unit)



#Homoskedastizität

con_r3 <- ggplot(con_diagnose, aes(x = .fitted, y = .resid)) + geom_point()


con_r4 <- ggplot(con_diagnose, aes(x = seq_along(.resid), y = .resid)) +

geom_point() + geom_smooth() + xlab("Index")


con_r3 + con_r4



###Ausreißer kontrollieren

#standardisierte Residuen ansehen

favstats(scale(con_diagnose$.resid))

       min         Q1    median        Q3      max          mean sd   n missing
 -2.863266 -0.6545066 0.1788195 0.7458114 2.269636 -2.057974e-17  1 410       0

con_r5 <- ggplot(con_diagnose, aes(y = .hat, label = as.numeric(rownames(con_diagnose)))) +
geom_boxplot() +
ggrepel::geom_text_repel(aes(x = 0), max.overlaps = 30)


con_r6 <- ggplot(con_diagnose, aes(y = .cooksd)) +
geom_boxplot()


con_r5 + con_r6



### 2. Variante -->

#p=11 (siehe Anova Befehl)

#The threshold in our sample is 2p/n=2*11/159=0,1383

#threshold berechnen:
#
# con_p <- anova(angst_m0, angst_m2)
#
# con_npar <- angst_p[2,1]
#
# con_n <- nrow(con_diagnose)/2 exp_lev_cutoff <- 3*angst_npar/angst_n
#
# con_r7 <- ggplot(con_diagnose, aes(y = .hat, label = subj_ID)) + geom_boxplot() +
#
# ggrepel::geom_text_repel(aes(x = 0), data =
#
# con_diagnose %>% filter(.hat > angst_lev_cutoff), max.overlaps = 30)
#
# con_r8 <- ggplot(con_diagnose, aes(y = .cooksd, label = subj_ID)) +
#
# geom_boxplot() + ggrepel::geom_text_repel(aes(x = 0))
#
# con_r7 + con_r8
```

Modelle Umfeld + Freigang

```
# ANOVA rechnen
con_m1 <- aov_car(sec ~ umfeld *zeit+ Error(versuch/zeit),
                  data=cat_long %>% dplyr::filter(verhalten=="con"),
                  type=3)
summary(con_m1)


Univariate Type III Repeated-Measures ANOVA Assuming Sphericity

            Sum Sq num Df Error SS den Df  F value    Pr(>F)
(Intercept) 540107      1   301108     80 143.4982 < 2.2e-16 ***
umfeld       52103      1   301108     80  13.8430 0.0003678 ***
zeit         27804      4   349313    320   6.3677 6.099e-05 ***
umfeld:zeit   7298      4   349313    320   1.6713 0.1563587
---
Signif. codes:  0 '***' 0.001 '**' 0.01 '*' 0.05 '.' 0.1 ' ' 1


Mauchly Tests for Sphericity

            Test statistic   p-value
zeit               0.71904 0.0021544
umfeld:zeit        0.71904 0.0021544


Greenhouse-Geisser and Huynh-Feldt Corrections
 for Departure from Sphericity

             GG eps Pr(>F[GG])
zeit        0.87443  0.0001485 ***
umfeld:zeit 0.87443  0.1649800
---
Signif. codes:  0 '***' 0.001 '**' 0.01 '*' 0.05 '.' 0.1 ' ' 1

              HF eps  Pr(>F[HF])
zeit        0.919307 0.000107981
umfeld:zeit 0.919307 0.161858557

effectsize::omega_squared(con_m1)

# Effect Size for ANOVA (Type III)

Parameter   | Omega2 (partial) |       95% CI
---------------------------------------------
umfeld      |             0.14 | [0.04, 1.00]
zeit        |             0.03 | [0.00, 1.00]
umfeld:zeit |         4.43e-03 | [0.00, 1.00]

- One-sided CIs: upper bound fixed at [1.00].

# LMM rechnen

cat_l <- cat_long %>%
  pivot_wider(names_from = verhalten, values_from = sec)

con_lmm_frei <- lmer(log(con+1) ~ umfeld*zeit*freigang + (1|versuch), data = cat_l)
summary(con_lmm_frei)

Linear mixed model fit by REML. t-tests use Satterthwaite's method [
lmerModLmerTest]
Formula: log(con + 1) ~ umfeld * zeit * freigang + (1 | versuch)
   Data: cat_l

REML criterion at convergence: 1460

Scaled residuals:
    Min      1Q  Median      3Q     Max
-2.5692 -0.5531  0.1675  0.6687  1.9187

Random effects:
 Groups   Name        Variance Std.Dev.
 versuch  (Intercept) 0.7812   0.8838
 Residual             1.7095   1.3075
Number of obs: 410, groups:  versuch, 82

Fixed effects:
                                           Estimate Std. Error        df
(Intercept)                                 3.52128    0.28814 279.87911
umfeldAnimal Shelter                       -0.07021    0.52404 279.87911
zeit2                                      -0.33920    0.33759 312.00000
zeit3                                      -0.52021    0.33759 312.00000
zeit4                                      -0.75184    0.33759 312.00000
zeit5                                      -0.78393    0.33759 312.00000
freigangIndoor                             -0.76776    0.38774 279.87911
umfeldAnimal Shelter:zeit2                  0.09488    0.61398 312.00000
umfeldAnimal Shelter:zeit3                 -0.14532    0.61398 312.00000
umfeldAnimal Shelter:zeit4                 -0.16478    0.61398 312.00000
umfeldAnimal Shelter:zeit5                  0.49437    0.61398 312.00000
umfeldAnimal Shelter:freigangIndoor         2.23472    1.25988 279.87911
zeit2:freigangIndoor                        0.29850    0.45429 312.00000
zeit3:freigangIndoor                       -0.11392    0.45429 312.00000
zeit4:freigangIndoor                       -0.02016    0.45429 312.00000
zeit5:freigangIndoor                        0.14635    0.45429 312.00000
umfeldAnimal Shelter:zeit2:freigangIndoor  -1.48732    1.47612 312.00000
umfeldAnimal Shelter:zeit3:freigangIndoor   0.34568    1.47612 312.00000
umfeldAnimal Shelter:zeit4:freigangIndoor   0.16627    1.47612 312.00000
umfeldAnimal Shelter:zeit5:freigangIndoor   0.01461    1.47612 312.00000
                                          t value Pr(>|t|)
(Intercept)                                12.221   <2e-16 ***
umfeldAnimal Shelter                       -0.134   0.8935
zeit2                                      -1.005   0.3158
zeit3                                      -1.541   0.1243
zeit4                                      -2.227   0.0267 *
zeit5                                      -2.322   0.0209 *
freigangIndoor                             -1.980   0.0487 *
umfeldAnimal Shelter:zeit2                  0.155   0.8773
umfeldAnimal Shelter:zeit3                 -0.237   0.8131
umfeldAnimal Shelter:zeit4                 -0.268   0.7886
umfeldAnimal Shelter:zeit5                  0.805   0.4213
umfeldAnimal Shelter:freigangIndoor         1.774   0.0772 .
zeit2:freigangIndoor                        0.657   0.5116
zeit3:freigangIndoor                       -0.251   0.8022
zeit4:freigangIndoor                       -0.044   0.9646
zeit5:freigangIndoor                        0.322   0.7476
umfeldAnimal Shelter:zeit2:freigangIndoor  -1.008   0.3144
umfeldAnimal Shelter:zeit3:freigangIndoor   0.234   0.8150
umfeldAnimal Shelter:zeit4:freigangIndoor   0.113   0.9104
umfeldAnimal Shelter:zeit5:freigangIndoor   0.010   0.9921
---
Signif. codes:  0 '***' 0.001 '**' 0.01 '*' 0.05 '.' 0.1 ' ' 1

anova(con_lmm_frei)

Type III Analysis of Variance Table with Satterthwaite's method
                     Sum Sq Mean Sq NumDF DenDF F value  Pr(>F)
umfeld               9.6867  9.6867     1    78  5.6663 0.01974 *
zeit                 8.5963  2.1491     4   312  1.2571 0.28686
freigang             0.9521  0.9521     1    78  0.5569 0.45774
umfeld:zeit          4.2174  1.0544     4   312  0.6167 0.65089
umfeld:freigang      9.9654  9.9654     1    78  5.8293 0.01810 *
zeit:freigang        1.4026  0.3506     4   312  0.2051 0.93548
umfeld:zeit:freigang 3.4127  0.8532     4   312  0.4991 0.73644
---
Signif. codes:  0 '***' 0.001 '**' 0.01 '*' 0.05 '.' 0.1 ' ' 1
```

Robust rechnen

```
# Robust linear mixed model

con_rlmm <- rlmer(con ~ umfeld*zeit + (1|versuch), data = cat_l)
summary(con_rlmm)

Robust linear mixed model fit by DAStau
Formula: con ~ umfeld * zeit + (1 | versuch)
   Data: cat_l

Scaled residuals:
    Min      1Q  Median      3Q     Max
-2.9270 -0.5604 -0.1702  0.5752  5.0044

Random effects:
 Groups   Name        Variance Std.Dev.
 versuch  (Intercept) 391.7    19.79
 Residual             759.3    27.56
Number of obs: 410, groups: versuch, 82

Fixed effects:
                           Estimate Std. Error t value
(Intercept)                  36.540      4.251   8.596
umfeldAnimal Shelter         39.964      9.939   4.021
zeit2                        -3.779      4.883  -0.774
zeit3                       -11.830      4.883  -2.423
zeit4                       -11.915      4.883  -2.440
zeit5                       -10.352      4.883  -2.120
umfeldAnimal Shelter:zeit2  -15.263     11.417  -1.337
umfeldAnimal Shelter:zeit3  -18.793     11.417  -1.646
umfeldAnimal Shelter:zeit4  -33.507     11.417  -2.935
umfeldAnimal Shelter:zeit5  -15.958     11.417  -1.398

Correlation of Fixed Effects:
            (Intr) umflAS zeit2  zeit3  zeit4  zeit5  umAS:2 umAS:3 umAS:4
umfldAnmlSh -0.428
zeit2       -0.574  0.246
zeit3       -0.574  0.246  0.500
zeit4       -0.574  0.246  0.500  0.500
zeit5       -0.574  0.246  0.500  0.500  0.500
umfldAShl:2  0.246 -0.574 -0.428 -0.214 -0.214 -0.214
umfldAShl:3  0.246 -0.574 -0.214 -0.428 -0.214 -0.214  0.500
umfldAShl:4  0.246 -0.574 -0.214 -0.214 -0.428 -0.214  0.500  0.500
umfldAShl:5  0.246 -0.574 -0.214 -0.214 -0.214 -0.428  0.500  0.500  0.500

Robustness weights for the residuals:
 332 weights are ~= 1. The remaining 78 ones are summarized as
   Min. 1st Qu.  Median    Mean 3rd Qu.    Max.
  0.269   0.568   0.734   0.721   0.926   0.999

Robustness weights for the random effects:
 71 weights are ~= 1. The remaining 11 ones are summarized as
   Min. 1st Qu.  Median    Mean 3rd Qu.    Max.
  0.320   0.578   0.712   0.687   0.807   0.979

Rho functions used for fitting:
  Residuals:
    eff: smoothed Huber (k = 1.345, s = 10)
    sig: smoothed Huber, Proposal 2 (k = 1.345, s = 10)
  Random Effects, variance component 1 (versuch):
    eff: smoothed Huber (k = 1.345, s = 10)
    vcp: smoothed Huber, Proposal 2 (k = 1.345, s = 10)

con_rlmm2 <- rlmer(con ~ umfeld*zeit*freigang + (1|versuch), data = cat_l)
summary(con_rlmm2)

Robust linear mixed model fit by DAStau
Formula: con ~ umfeld * zeit * freigang + (1 | versuch)
   Data: cat_l

Scaled residuals:
    Min      1Q  Median      3Q     Max
-2.7681 -0.5437 -0.1291  0.5613  4.9048

Random effects:
 Groups   Name        Variance Std.Dev.
 versuch  (Intercept) 307.9    17.55
 Residual             759.0    27.55
Number of obs: 410, groups: versuch, 82

Fixed effects:
                                          Estimate Std. Error t value
(Intercept)                                47.6270     6.1163   7.787
umfeldAnimal Shelter                       17.4185    11.1237   1.566
zeit2                                      -8.1447     7.2957  -1.116
zeit3                                     -11.6610     7.2957  -1.598
zeit4                                     -13.6706     7.2957  -1.874
zeit5                                     -18.4529     7.2957  -2.529
freigangIndoor                            -19.4091     8.2305  -2.358
umfeldAnimal Shelter:zeit2                 -5.1995    13.2687  -0.392
umfeldAnimal Shelter:zeit3                -17.8945    13.2687  -1.349
umfeldAnimal Shelter:zeit4                -25.1418    13.2687  -1.895
umfeldAnimal Shelter:zeit5                 -4.8877    13.2687  -0.368
umfeldAnimal Shelter:freigangIndoor        92.3636    26.7433   3.454
zeit2:freigangIndoor                        7.0633     9.8176   0.719
zeit3:freigangIndoor                       -0.3473     9.8176  -0.035
zeit4:freigangIndoor                        2.6558     9.8176   0.271
zeit5:freigangIndoor                       13.2160     9.8176   1.346
umfeldAnimal Shelter:zeit2:freigangIndoor -53.2191    31.9003  -1.668
umfeldAnimal Shelter:zeit3:freigangIndoor  -7.5972    31.9003  -0.238
umfeldAnimal Shelter:zeit4:freigangIndoor -38.3434    31.9003  -1.202
umfeldAnimal Shelter:zeit5:freigangIndoor  -7.3755    31.9003  -0.231

Correlation of Fixed Effects:
            (Intr) umflAS zeit2  zeit3  zeit4  zeit5  frgngI umAS:2 umAS:3
umfldAnmlSh -0.550
zeit2       -0.596  0.328
zeit3       -0.596  0.328  0.500
zeit4       -0.596  0.328  0.500  0.500
zeit5       -0.596  0.328  0.500  0.500  0.500
freigngIndr -0.743  0.409  0.443  0.443  0.443  0.443
umfldAShl:2  0.328 -0.596 -0.550 -0.275 -0.275 -0.275 -0.244
umfldAShl:3  0.328 -0.596 -0.275 -0.550 -0.275 -0.275 -0.244  0.500
umfldAShl:4  0.328 -0.596 -0.275 -0.275 -0.550 -0.275 -0.244  0.500  0.500
umfldAShl:5  0.328 -0.596 -0.275 -0.275 -0.275 -0.550 -0.244  0.500  0.500
umfldAShl:I  0.229 -0.416 -0.136 -0.136 -0.136 -0.136 -0.308  0.248  0.248
zt2:frgngIn  0.443 -0.244 -0.743 -0.372 -0.372 -0.372 -0.596  0.409  0.204
zt3:frgngIn  0.443 -0.244 -0.372 -0.743 -0.372 -0.372 -0.596  0.204  0.409
zt4:frgngIn  0.443 -0.244 -0.372 -0.372 -0.743 -0.372 -0.596  0.204  0.204
zt5:frgngIn  0.443 -0.244 -0.372 -0.372 -0.372 -0.743 -0.596  0.204  0.204
umfldAS:2:I -0.136  0.248  0.229  0.114  0.114  0.114  0.184 -0.416 -0.208
umfldAS:3:I -0.136  0.248  0.114  0.229  0.114  0.114  0.184 -0.208 -0.416
umfldAS:4:I -0.136  0.248  0.114  0.114  0.229  0.114  0.184 -0.208 -0.208
umfldAS:5:I -0.136  0.248  0.114  0.114  0.114  0.229  0.184 -0.208 -0.208
            umAS:4 umAS:5 umAS:I zt2:fI zt3:fI zt4:fI zt5:fI uAS:2: uAS:3:
umfldAnmlSh
zeit2
zeit3
zeit4
zeit5
freigngIndr
umfldAShl:2
umfldAShl:3
umfldAShl:4
umfldAShl:5  0.500
umfldAShl:I  0.248  0.248
zt2:frgngIn  0.204  0.204  0.184
zt3:frgngIn  0.204  0.204  0.184  0.500
zt4:frgngIn  0.409  0.204  0.184  0.500  0.500
zt5:frgngIn  0.204  0.409  0.184  0.500  0.500  0.500
umfldAS:2:I -0.208 -0.208 -0.596 -0.308 -0.154 -0.154 -0.154
umfldAS:3:I -0.208 -0.208 -0.596 -0.154 -0.308 -0.154 -0.154  0.500
umfldAS:4:I -0.416 -0.208 -0.596 -0.154 -0.154 -0.308 -0.154  0.500  0.500
umfldAS:5:I -0.208 -0.416 -0.596 -0.154 -0.154 -0.154 -0.308  0.500  0.500
            uAS:4:
umfldAnmlSh
zeit2
zeit3
zeit4
zeit5
freigngIndr
umfldAShl:2
umfldAShl:3
umfldAShl:4
umfldAShl:5
umfldAShl:I
zt2:frgngIn
zt3:frgngIn
zt4:frgngIn
zt5:frgngIn
umfldAS:2:I
umfldAS:3:I
umfldAS:4:I
umfldAS:5:I  0.500

Robustness weights for the residuals:
 336 weights are ~= 1. The remaining 74 ones are summarized as
   Min. 1st Qu.  Median    Mean 3rd Qu.    Max.
  0.274   0.552   0.706   0.703   0.905   0.996

Robustness weights for the random effects:
 73 weights are ~= 1. The remaining 9 ones are
    4     5     8    11    13    15    22    63    68
0.409 0.831 0.267 0.930 0.996 0.736 0.619 0.763 0.804

Rho functions used for fitting:
  Residuals:
    eff: smoothed Huber (k = 1.345, s = 10)
    sig: smoothed Huber, Proposal 2 (k = 1.345, s = 10)
  Random Effects, variance component 1 (versuch):
    eff: smoothed Huber (k = 1.345, s = 10)
    vcp: smoothed Huber, Proposal 2 (k = 1.345, s = 10)
```

Effektstärke

```
##Cohen's d berechnen


reffects_con<-broom.mixed::tidy(con_lmm_frei, effects = c("ran_pars"), scale="vcov")


md_con <-summary(con_lmm_frei)$coef[2] #Md = Mittelwertsdifferenz
sdPart_con<-sqrt(sum(reffects_con$estimate)) #Random-Effects zusammenzählen
##Cohen´s d für alle Faktoren



cohend_con_frei <- data.frame(cbind(summary(con_lmm_frei)$coef[, 1]/sdPart_con)[c(2,3,4,5,6,7,8,9,10),])
colnames(cohend_con) <- "CohensD_con_f"
```

Grafik

```
# Grafik auf Basiis der LMM für Umfeld

em_df_con_f1<- data.frame(emmeans(con_lmm_frei, "umfeld", by="zeit"))

ggplot(em_df_con_f1, aes(x = zeit, y = emmean, group=umfeld, color=umfeld))+
  geom_line() +
  geom_errorbar(aes(ymin=lower.CL, ymax=upper.CL), width=.4,
                position=position_dodge(0.01)) +
  geom_point(aes(shape=umfeld))+
  scale_colour_manual(values=c("#D700FF","#00C0FA"))+
  scale_y_continuous(limits=c(0,20),breaks=seq(0,20,2)) +
  theme_bw() +
  labs(x="Video Sequence (No)", y = "Physical Contact (sec)",color="Living Environment",shape="Living Environment")



# log-transformierte unabhängige Variable - muss zurück transformiert werden für die Grafik
# First transform the values back to seconds
em_df_con_f1$emmean <- exp(em_df_con_f1$emmean) - 1
em_df_con_f1$lower.CL <- exp(em_df_con_f1$lower.CL) - 1
em_df_con_f1$upper.CL <- exp(em_df_con_f1$upper.CL) - 1

# Create the plot with transformed values
ggplot(em_df_con_f1, aes(x = zeit, y = emmean, group=umfeld, color=umfeld))+
  geom_line() +
  geom_errorbar(aes(ymin=lower.CL, ymax=upper.CL), width=.4,
                position=position_dodge(0.01)) +
  geom_point(aes(shape=umfeld))+
  scale_colour_manual(values=c("#D700FF","#00C0FA"))+
  scale_y_continuous(limits=c(0,120), breaks=seq(0,120,10)) +
  theme_bw() +
  labs(x="Video Sequence (No)", y = "Physical Contact (sec)",color="Living Environment",shape="Living Environment")



# Grafik auf Basiis der LMM für Freigang

em_df_con_f2<- data.frame(emmeans(con_lmm_frei, "freigang", by="zeit"))

ggplot(em_df_con_f2, aes(x = zeit, y = emmean, group=freigang, color=freigang))+
  geom_line() +
  geom_errorbar(aes(ymin=lower.CL, ymax=upper.CL), width=.4,
                position=position_dodge(0.01)) +
  geom_point(aes(shape=freigang))+
  scale_colour_manual(values=c("#D700FF","#00C0FA"))+
  scale_y_continuous(limits=c(0,20),breaks=seq(0,20,2)) +
  theme_bw() +
  labs(x="Video Sequence (No)", y = "Physical Contact (sec)",color="Outdoor Access",shape="Outdoor Access")



# log-transformierte unabhängige Variable - muss zurück transformiert werden für die Grafik
# First transform the values back to seconds
em_df_con_f2$emmean <- exp(em_df_con_f2$emmean) - 1
em_df_con_f2$lower.CL <- exp(em_df_con_f2$lower.CL) - 1
em_df_con_f2$upper.CL <- exp(em_df_con_f2$upper.CL) - 1

# Create the plot with transformed values
con_f1 <-ggplot(em_df_con_f2, aes(x = zeit, y = emmean, group=freigang, color=freigang))+
  geom_line() +
  geom_errorbar(aes(ymin=lower.CL, ymax=upper.CL), width=.4,
                position=position_dodge(0.01)) +
  geom_point(aes(shape=freigang))+
  scale_colour_manual(values=c("#D700FF","#00C0FA"))+
  scale_y_continuous(limits=c(0,120), breaks=seq(0,120,10)) +
  theme_bw() +
  labs(x="Video Sequence (No)", y = "Physical Contact (sec)",color="Outdoor Access",shape="Outdoor Access")

con_f1
```

Paarweise Vergleiche

```
pairs(emmeans(con_lmm_frei, "umfeld", by="zeit") , adjust="dunnet")

zeit = 1:
 contrast                       estimate   SE  df t.ratio p.value
 Private Home - Animal Shelter    -1.047 0.63 280  -1.662  0.0976

zeit = 2:
 contrast                       estimate   SE  df t.ratio p.value
 Private Home - Animal Shelter    -0.398 0.63 280  -0.632  0.5276

zeit = 3:
 contrast                       estimate   SE  df t.ratio p.value
 Private Home - Animal Shelter    -1.075 0.63 280  -1.706  0.0891

zeit = 4:
 contrast                       estimate   SE  df t.ratio p.value
 Private Home - Animal Shelter    -0.966 0.63 280  -1.533  0.1265

zeit = 5:
 contrast                       estimate   SE  df t.ratio p.value
 Private Home - Animal Shelter    -1.549 0.63 280  -2.459  0.0145

Results are averaged over the levels of: freigang
Note: contrasts are still on the log(mu + 1) scale. Consider using
      regrid() if you want contrasts of back-transformed estimates.
Degrees-of-freedom method: kenward-roger

pairs(emmeans(con_lmm_frei, "zeit", by="umfeld") , adjust="dunnet")

umfeld = Private Home:
 contrast      estimate    SE  df t.ratio p.value
 zeit1 - zeit2  0.18995 0.227 312   0.836  0.9267
 zeit1 - zeit3  0.57717 0.227 312   2.541  0.0869
 zeit1 - zeit4  0.76192 0.227 312   3.354  0.0080
 zeit1 - zeit5  0.71076 0.227 312   3.129  0.0166
 zeit2 - zeit3  0.38722 0.227 312   1.705  0.4500
 zeit2 - zeit4  0.57197 0.227 312   2.518  0.0919
 zeit2 - zeit5  0.52081 0.227 312   2.293  0.1553
 zeit3 - zeit4  0.18475 0.227 312   0.813  0.9333
 zeit3 - zeit5  0.13359 0.227 312   0.588  0.9791
 zeit4 - zeit5 -0.05116 0.227 312  -0.225  0.9995

umfeld = Animal Shelter:
 contrast      estimate    SE  df t.ratio p.value
 zeit1 - zeit2  0.83873 0.702 312   1.194  0.7722
 zeit1 - zeit3  0.54965 0.702 312   0.783  0.9416
 zeit1 - zeit4  0.84356 0.702 312   1.201  0.7683
 zeit1 - zeit5  0.20908 0.702 312   0.298  0.9985
 zeit2 - zeit3 -0.28908 0.702 312  -0.412  0.9946
 zeit2 - zeit4  0.00484 0.702 312   0.007  1.0000
 zeit2 - zeit5 -0.62965 0.702 312  -0.897  0.9072
 zeit3 - zeit4  0.29392 0.702 312   0.419  0.9942
 zeit3 - zeit5 -0.34057 0.702 312  -0.485  0.9898
 zeit4 - zeit5 -0.63449 0.702 312  -0.904  0.9048

Results are averaged over the levels of: freigang
Note: contrasts are still on the log(mu + 1) scale. Consider using
      regrid() if you want contrasts of back-transformed estimates.
Degrees-of-freedom method: kenward-roger
P value adjustment: dunnettx method for 10 tests

pairs(emmeans(con_lmm_frei, "zeit", by="freigang") , adjust="dunnet")

freigang = Outdoor:
 contrast      estimate    SE  df t.ratio p.value
 zeit1 - zeit2   0.2918 0.307 312   0.950  0.8876
 zeit1 - zeit3   0.5929 0.307 312   1.931  0.3155
 zeit1 - zeit4   0.8342 0.307 312   2.717  0.0550
 zeit1 - zeit5   0.5367 0.307 312   1.748  0.4225
 zeit2 - zeit3   0.3011 0.307 312   0.981  0.8755
 zeit2 - zeit4   0.5425 0.307 312   1.767  0.4110
 zeit2 - zeit5   0.2450 0.307 312   0.798  0.9376
 zeit3 - zeit4   0.2414 0.307 312   0.786  0.9407
 zeit3 - zeit5  -0.0561 0.307 312  -0.183  0.9998
 zeit4 - zeit5  -0.2975 0.307 312  -0.969  0.8803

freigang = Indoor:
 contrast      estimate    SE  df t.ratio p.value
 zeit1 - zeit2   0.7369 0.671 312   1.098  0.8227
 zeit1 - zeit3   0.5340 0.671 312   0.796  0.9382
 zeit1 - zeit4   0.7713 0.671 312   1.149  0.7966
 zeit1 - zeit5   0.3831 0.671 312   0.571  0.9813
 zeit2 - zeit3  -0.2030 0.671 312  -0.302  0.9984
 zeit2 - zeit4   0.0343 0.671 312   0.051  1.0000
 zeit2 - zeit5  -0.3538 0.671 312  -0.527  0.9861
 zeit3 - zeit4   0.2373 0.671 312   0.354  0.9970
 zeit3 - zeit5  -0.1509 0.671 312  -0.225  0.9995
 zeit4 - zeit5  -0.3882 0.671 312  -0.578  0.9803

Results are averaged over the levels of: umfeld
Note: contrasts are still on the log(mu + 1) scale. Consider using
      regrid() if you want contrasts of back-transformed estimates.
Degrees-of-freedom method: kenward-roger
P value adjustment: dunnettx method for 10 tests

pairs(emmeans(con_lmm_frei, "freigang", by="zeit") , adjust="dunnet")

zeit = 1:
 contrast         estimate   SE  df t.ratio p.value
 Outdoor - Indoor  -0.3496 0.63 280  -0.555  0.5794

zeit = 2:
 contrast         estimate   SE  df t.ratio p.value
 Outdoor - Indoor   0.0956 0.63 280   0.152  0.8795

zeit = 3:
 contrast         estimate   SE  df t.ratio p.value
 Outdoor - Indoor  -0.4085 0.63 280  -0.649  0.5172

zeit = 4:
 contrast         estimate   SE  df t.ratio p.value
 Outdoor - Indoor  -0.4126 0.63 280  -0.655  0.5130

zeit = 5:
 contrast         estimate   SE  df t.ratio p.value
 Outdoor - Indoor  -0.5033 0.63 280  -0.799  0.4250

Results are averaged over the levels of: umfeld
Note: contrasts are still on the log(mu + 1) scale. Consider using
      regrid() if you want contrasts of back-transformed estimates.
Degrees-of-freedom method: kenward-roger

confint(pairs(emmeans(con_lmm_frei,~umfeld)))

 contrast                       estimate    SE df lower.CL upper.CL
 Private Home - Animal Shelter     -1.01 0.423 78    -1.85   -0.165

Results are averaged over the levels of: zeit, freigang
Note: contrasts are still on the log(mu + 1) scale. Consider using
      regrid() if you want contrasts of back-transformed estimates.
Degrees-of-freedom method: kenward-roger
Confidence level used: 0.95

confint(pairs(emmeans(con_lmm_frei,~zeit)))

 contrast      estimate    SE  df lower.CL upper.CL
 zeit1 - zeit2   0.5143 0.369 312   -0.498    1.527
 zeit1 - zeit3   0.5634 0.369 312   -0.449    1.576
 zeit1 - zeit4   0.8027 0.369 312   -0.210    1.815
 zeit1 - zeit5   0.4599 0.369 312   -0.553    1.472
 zeit2 - zeit3   0.0491 0.369 312   -0.963    1.062
 zeit2 - zeit4   0.2884 0.369 312   -0.724    1.301
 zeit2 - zeit5  -0.0544 0.369 312   -1.067    0.958
 zeit3 - zeit4   0.2393 0.369 312   -0.773    1.252
 zeit3 - zeit5  -0.1035 0.369 312   -1.116    0.909
 zeit4 - zeit5  -0.3428 0.369 312   -1.355    0.670

Results are averaged over the levels of: umfeld, freigang
Note: contrasts are still on the log(mu + 1) scale. Consider using
      regrid() if you want contrasts of back-transformed estimates.
Degrees-of-freedom method: kenward-roger
Confidence level used: 0.95
Conf-level adjustment: tukey method for comparing a family of 5 estimates

confint(pairs(emmeans(con_lmm_frei,~freigang)))

 contrast         estimate    SE df lower.CL upper.CL
 Outdoor - Indoor   -0.316 0.423 78    -1.16    0.526

Results are averaged over the levels of: umfeld, zeit
Note: contrasts are still on the log(mu + 1) scale. Consider using
      regrid() if you want contrasts of back-transformed estimates.
Degrees-of-freedom method: kenward-roger
Confidence level used: 0.95
```

Residuen

```
con_frei_diagnose <- broom.mixed::augment(con_lmm_frei)

#Normalverteiung - annähernd normalverteilt

con_r1 <- ggplot(con_frei_diagnose, aes(x = .resid)) +

geom_histogram(aes(x = .resid, y = ..density..)) +

geom_density(aes(colour = "density estimate")) +

geom_function(aes(colour = "normal curve"), fun = dnorm,

args = list(mean = mean(con_frei_diagnose$.resid),sd = sd(con_frei_diagnose$.resid))) +

scale_colour_discrete(name = "Curve") +

[illegible]
```

```
theme(legend.position = "top")

con_r2 <- ggplot(con_frei_diagnose, aes(sample = .resid)) +
geom_qq() +
geom_qq_line()
con_r1 + con_r2

#DHARMa - prüft automatisch Residuen auf Normalverteilung & Abweichungen
q_unit_fr <- simulateResiduals(con_lmm_frei)
plot(q_unit_fr)

#Homoskedastizität
con_r3 <- ggplot(con_frei_diagnose, aes(x = .fitted, y = .resid)) + geom_point()

con_r4 <- ggplot(con_frei_diagnose, aes(x = seq_along(.resid), y = .resid)) +
geom_point() + geom_smooth() + xlab("Index")

con_r3 + con_r4

###Ausreißer kontrollieren
#standardisierte Residuen ansehen
favstats(scale(con_frei_diagnose$.resid))

       min         Q1    median        Q3    max          mean sd   n missing
 -2.835707 -0.6104107 0.1849134 0.7380766 2.1177 3.411905e-17  1 410       0

#con_r5 <- ggplot(con_frei_diagnose, aes(y = .hat, label = as.numeric(rownames(con_diagnose))) +
#geom_boxplot() +
#ggrepel::geom_text_repel(aes(x = 0), max.overlaps = 30)

#con_r6 <- ggplot(con_frei_diagnose, aes(y = .cooksd)) +
#geom_boxplot()

#con_r5 + con_r6
### 2. Variante -->
#p=11 (siehe Anova Befehl)
#The threshold in our sample is 2p/n=2*11/159=0,1383
#threshold berechnen:
#
# con_p <- anova(angst_m0, angst_m2)
#
# con_npar <- angst_p[2,1]
#
# con_n <- nrow(con_diagnose)/2 exp_lev_cutoff <- 3*angst_npar/angst_n
#
# con_r7 <- ggplot(con_diagnose, aes(y = .hat, label = subj_ID)) + geom_boxplot() +
#
# ggrepel::geom_text_repel(aes(x = 0), data =
#
# con_diagnose %>% filter(.hat > angst_lev_cutoff), max.overlaps = 30)
#
# con_r8 <- ggplot(con_diagnose, aes(y = .cooksd, label = subj_ID)) +
#
# geom_boxplot() + ggrepel::geom_text_repel(aes(x = 0))
#
# con_r7 + con_r8
```

Tabelle Ergebnisse

```
#LMMs mit 2 unabhängigen Variablen
tab_model(exp_lmm,df.method = "satterthwaite", show.se = T, collapse.se = TRUE, show.r2 = F,  show.ci=F, p.style = "stars")
```

	exp
Predictors	*Estimates*
(Intercept)	69.96 *** (6.32)
umfeld [Animal Shelter]	17.24 (14.77)
zeit [2]	2.43 (5.36)
zeit [3]	-12.39 * (5.36)
zeit [4]	-13.88 * (5.36)
zeit [5]	-9.49 (5.36)
umfeld [Animal Shelter] × zeit [2]	-8.70 (12.53)
umfeld [Animal Shelter] × zeit [3]	17.52 (12.53)
umfeld [Animal Shelter] × zeit [4]	12.28 (12.53)
umfeld [Animal Shelter] × zeit [5]	14.16 (12.53)
**Random Effects**	
σ^2	962.07
$\tau_{00\ versuch}$	1710.05
ICC	0.64
$N_{versuch}$	82
Observations	410

* p<0.05 ** p<0.01 *** p<0.001

```
tab_model(exp_lmm, passiv_lmm,df.method = "satterthwaite", show.se = T, collapse.se = TRUE, show.r2 = F, show.ci=F, p.style = "stars")
```

	exp	passiv
Predictors	*Estimates*	*Estimates*
(Intercept)	69.96 *** (6.32)	43.67 *** (6.00)
umfeld [Animal Shelter]	17.24 (14.77)	-38.74 ** (14.02)
zeit [2]	2.43 (5.36)	8.76 (5.54)
zeit [3]	-12.39 * (5.36)	10.91 * (5.54)
zeit [4]	-13.88 * (5.36)	7.93 (5.54)
zeit [5]	-9.49 (5.36)	9.27 (5.54)
umfeld [Animal Shelter] × zeit [2]	-8.70 (12.53)	1.17 (12.96)
umfeld [Animal Shelter] × zeit [3]	17.52 (12.53)	8.36 (12.96)
umfeld [Animal Shelter] × zeit [4]	12.28 (12.53)	14.87 (12.96)
umfeld [Animal Shelter] × zeit [5]	14.16 (12.53)	8.53 (12.96)
**Random Effects**		
σ^2	962.07	1029.09
τ_{00}	1710.05 $_{versuch}$	1379.57 $_{versuch}$
ICC	0.64	0.57
N	82 $_{versuch}$	82 $_{versuch}$
Observations	410	410

* p<0.05 ** p<0.01 *** p<0.001

```
#log-transformierte Erwartungswerte zurück transformieren
tab_model(ply_lmm, tuer_lmm, con_lmm, df.method = "satterthwaite", show.se = T, collapse.se = TRUE, show.r2 = F, show.ci=F, p.style = "stars", transform="exp" ,dv.labels=c("play behaviou
```

	play behaviour	behaviour door	physical contact
Predictors	*Estimates*	*Estimates*	*Estimates*
(Intercept)	4.41 *** (1.02)	4.86 *** (1.00)	22.14 *** (4.34)
umfeld [Animal Shelter]	0.40 (0.22)	0.50 (0.24)	1.73 (0.79)
zeit [2]	0.76 (0.15)	0.79 (0.17)	0.84 (0.19)
zeit [3]	1.24 (0.25)	1.93 ** (0.41)	0.56 ** (0.12)
zeit [4]	1.64 * (0.33)	0.61 * (0.13)	0.47 *** (0.10)
zeit [5]	0.97 (0.20)	0.87 (0.19)	0.50 ** (0.11)
umfeld [Animal Shelter] × zeit [2]	1.55 (0.73)	0.90 (0.45)	0.80 (0.42)
umfeld [Animal Shelter] × zeit [3]	1.24 (0.59)	0.77 (0.39)	0.95 (0.50)
umfeld [Animal Shelter] × zeit [4]	0.63 (0.32)	1.30 (0.65)	0.87 (0.46)
umfeld [Animal Shelter] × zeit [5]	1.06 (0.50)	2.73 * (1.36)	1.54 (0.81)
**Random Effects**			
σ^2	1.38	1.52	1.68
τ_{00}	2.21 $_{versuch}$	1.28 $_{versuch}$	0.90 $_{versuch}$
ICC	0.62	0.46	0.35
N	82 $_{versuch}$	82 $_{versuch}$	82 $_{versuch}$
Observations	406	410	410

* p<0.05 ** p<0.01 *** p<0.001

```
#column.names = c("play behaviour", "behaviour by the door", "physical contact")
#header=c("play behaviour", "behaviour by the door", "physical contact")

#Tabelle zusammen bauen
#tab model header anpassen

#LMMs mit 3 unabhängigen Variablen
tab_model(exp_lmm_frei,df.method = "satterthwaite", show.se = T, collapse.se = TRUE, show.r2 = F,  show.ci=F, p.style = "stars")
```

	exp
Predictors	*Estimates*
(Intercept)	49.43 *** (9.27)
umfeld [Animal Shelter]	45.18 ** (16.85)
zeit [2]	4.00 (7.88)
zeit [3]	-0.67 (7.88)
zeit [4]	0.80 (7.88)
zeit [5]	5.70 (7.88)
freigang [Indoor]	37.16 ** (12.47)
umfeld [Animal Shelter] × zeit [2]	-16.38 (14.33)
umfeld [Animal Shelter] × zeit [3]	3.13 (14.33)
umfeld [Animal Shelter] × zeit [4]	-1.65 (14.33)
umfeld [Animal Shelter] × zeit [5]	-9.32 (14.33)
umfeld [Animal Shelter] × freigang [Indoor]	-92.78 * (40.52)
zeit [2] × freigang [Indoor]	-2.84 (10.60)
zeit [3] × freigang [Indoor]	-21.23 * (10.60)
zeit [4] × freigang [Indoor]	-26.58 * (10.60)
zeit [5] × freigang [Indoor]	-27.51 ** (10.60)
(umfeld [Animal Shelter] × zeit [2]) × freigang [Indoor]	48.72 (34.44)
(umfeld [Animal Shelter] × zeit [3]) × freigang [Indoor]	41.26 (34.44)
(umfeld [Animal Shelter] × zeit [4]) × freigang [Indoor]	20.93 (34.44)
(umfeld [Animal Shelter] × zeit [5]) × freigang [Indoor]	89.63 ** (34.44)
**Random Effects**	
σ^2	930.65
$\tau_{00\ versuch}$	1645.11
ICC	0.64
$N_{versuch}$	82
Observations	410

* p<0.05 ** p<0.01 *** p<0.001

```
tab_model(exp_lmm_frei, df.method = "satterthwaite", show.se = T, collapse.se = TRUE, show.r2 = F, show.ci=F, p.style = "stars")
```

	exp
Predictors	*Estimates*
(Intercept)	49.43 *** (9.27)
umfeld [Animal Shelter]	45.18 ** (16.85)
zeit [2]	4.00 (7.88)
zeit [3]	-0.67 (7.88)
zeit [4]	0.80 (7.88)
zeit [5]	5.70 (7.88)
freigang [Indoor]	37.16 ** (12.47)
umfeld [Animal Shelter] × zeit [2]	-16.38 (14.33)
umfeld [Animal Shelter] × zeit [3]	3.13 (14.33)
umfeld [Animal Shelter] × zeit [4]	-1.65 (14.33)
umfeld [Animal Shelter] × zeit [5]	-9.32 (14.33)
umfeld [Animal Shelter] × freigang [Indoor]	-92.78 * (40.52)
zeit [2] × freigang [Indoor]	-2.84 (10.60)
zeit [3] × freigang [Indoor]	-21.23 * (10.60)
zeit [4] × freigang [Indoor]	-26.58 * (10.60)
zeit [5] × freigang [Indoor]	-27.51 ** (10.60)
(umfeld [Animal Shelter] × zeit [2]) × freigang [Indoor]	48.72 (34.44)
(umfeld [Animal Shelter] × zeit [3]) × freigang [Indoor]	41.26 (34.44)
(umfeld [Animal Shelter] × zeit [4]) × freigang [Indoor]	20.93 (34.44)
(umfeld [Animal Shelter] × zeit [5]) × freigang [Indoor]	89.63 ** (34.44)
**Random Effects**	
σ^2	930.65
$\tau_{00\ versuch}$	1645.11
ICC	0.64
$N_{versuch}$	82
Observations	410

* p<0.05 ** p<0.01 *** p<0.001

```
tab_model(ply_lmm_frei, con_lmm_frei, df.method = "satterthwaite", show.se = T, collapse.se = TRUE, show.r2 = F, show.ci=F, p.style = "stars", transform="exp" )
```

	log(ply+1)	log(con+1)
Predictors	*Estimates*	*Estimates*
(Intercept)	3.65 *** (1.27)	33.83 *** (9.75)
umfeld [Animal Shelter]	0.53 (0.33)	0.93 (0.49)
zeit [2]	0.87 (0.26)	0.71 (0.24)
zeit [3]	1.16 (0.35)	0.59 (0.20)
zeit [4]	1.75 (0.53)	0.47 * (0.16)
zeit [5]	0.68 (0.21)	0.46 * (0.15)
freigang [Indoor]	1.41 (0.66)	0.46 * (0.18)
umfeld [Animal Shelter] × zeit [2]	1.39 (0.76)	1.10 (0.68)
umfeld [Animal Shelter] × zeit [3]	1.26 (0.70)	0.86 (0.53)
umfeld [Animal Shelter] × zeit [4]	0.55 (0.32)	0.85 (0.52)
umfeld [Animal Shelter] × zeit [5]	1.20 (0.66)	1.64 (1.01)
umfeld [Animal Shelter] × freigang [Indoor]	0.37 (0.56)	9.34 (11.77)
zeit [2] × freigang [Indoor]	0.78 (0.32)	1.35 (0.61)
zeit [3] × freigang [Indoor]	1.14 (0.46)	0.89 (0.41)
zeit [4] × freigang [Indoor]	0.89 (0.36)	0.98 (0.45)
zeit [5] × freigang [Indoor]	1.91 (0.78)	1.16 (0.53)
(umfeld [Animal Shelter] × zeit [2]) × freigang [Indoor]	1.05 (1.39)	0.23 (0.33)
(umfeld [Animal Shelter] × zeit [3]) × freigang [Indoor]	1.34 (1.78)	1.41 (2.09)
(umfeld [Animal Shelter] × zeit [4]) × freigang [Indoor]	1.94 (3.14)	1.18 (1.74)
(umfeld [Animal Shelter] × zeit [5]) × freigang [Indoor]	2.93 (3.88)	1.01 (1.50)
**Random Effects**		
σ^2	1.37	1.71
τ_{00}	2.24 $_{versuch}$	0.78 $_{versuch}$
ICC	0.62	0.31
N	82 $_{versuch}$	82 $_{versuch}$
Observations	406	410

* p<0.05 ** p<0.01 *** p<0.001

```
#Tabelle zusammen bauen
#tab model header anpassen
```

Grafiken Ergebnisse

```
exp_g1 + con_g1 + passiv_g1 + tuer_g1 +  ply_g1

exp_f2 + con_f1 +  ply_f2

#alle em_df Datensätze zusammengeben und rbind, davor muss man Variable einfügen mit dem Namen der Variable "exp", "ply"
#facet_wrap(~verhalten) zum Grafik basteln

# Angenommene Datensätze em_df_exp_m1, em_df_ply_m1, etc.
# Füge eine zusätzliche Variable 'verhalten' für jedes Dataset hinzu

# Erstelle Beispiel-Datensätze, wie im Beispiel beschrieben
em_df_exp_m1$verhalten <- "Exploration Behaviour"
em_df_ply_m1$verhalten <- "Play Behaviour"
em_df_passiv_m1$verhalten <- "Passiv Behvaiour"
em_df_tuer_m1$verhalten <- "Behaviour bt Door"
em_df_con_m1$verhalten <- "Physical Contact"

# Verbinde alle Datensätze zu einem großen DataFrame
combined_df <- rbind(em_df_exp_m1, em_df_ply_m1, em_df_passiv_m1, em_df_tuer_m1, em_df_con_m1)

# Erstelle die ggplot-Grafik mit facet_wrap
ggplot(combined_df, aes(x = zeit, y = emmean, group = umfeld, color = umfeld)) +
  geom_line() +
  geom_errorbar(aes(ymin = lower.CL, ymax = upper.CL), width = .4,
                position = position_dodge(0.01)) +
  geom_point(aes(shape = umfeld)) +
  scale_colour_manual(values = c("#D700FF", "#00C0FA")) +
    theme_bw() +
  labs(x = "Video Sequence (No)", y = "Exploration Behaviour (sec)", color = "Living Environment", shape = "Living Environment") +
  facet_wrap(~verhalten, scales="free_y")

#scale_y_continuous(limits = c(0, 120), breaks = seq(0, 120, 10)) +

#alle em_df Datensätze zusammengeben und rbind, davor muss man Variable einfügen mit dem Namen der Variable "exp", "ply"
#facet_wrap(~verhalten) zum Grafik basteln

# Angenommene Datensätze em_df_exp_m1, em_df_ply_m1, etc.
# Füge eine zusätzliche Variable 'verhalten' für jedes Dataset hinzu

# Erstelle Beispiel-Datensätze, wie im Beispiel beschrieben
em_df_exp_f3$verhalten <- "Exploration Behaviour"
em_df_ply_f3$verhalten <- "Play Behaviour"
em_df_con_f2$verhalten <- "Physical Contact"

# Verbinde alle Datensätze zu einem großen DataFrame
combined_df <- rbind(em_df_exp_f3, em_df_ply_f3, em_df_con_f2)

# Erstelle die ggplot-Grafik mit facet_wrap
ggplot(combined_df, aes(x = zeit, y = emmean, group = freigang, color = freigang)) +
  geom_line() +
  geom_errorbar(aes(ymin = lower.CL, ymax = upper.CL), width = .4,
                position = position_dodge(0.01)) +
  geom_point(aes(shape = freigang)) +
  scale_colour_manual(values = c("#D700FF", "#00C0FA")) +
  scale_y_continuous(limits = c(0, 120), breaks = seq(0, 120, 10)) +
  theme_bw() +
  labs(x = "Video Sequence (No)", y = "Behaviour (sec)", color = "Outdoor Access", shape = "Outdoor Access") +
  facet_wrap(~verhalten)
```

Inferenzielle Analyse - Subset 2

Initiierendes Verhalten (kontaktini)

Verteilung

```
# table(subset2)

# Zähldaten: Poisson Regression
# Voraussetzung: Erwartungswert = Varianz
# --> Praxis oft nicht so
# Alternative: Negativ binomial

ggplot(cat_l2, aes(x=kontaktini)) +
  geom_histogram() +
  facet_wrap(~zeit)

#Graphischer Vergleich Poisson/negbin
# person 8 ausschließen

cat_l2b <- cat_l2 %>% dplyr::filter(!is.na(kontaktini))

kontaktini_p1 <- ggplot(cat_l2b) +
  geom_bar(aes(x = kontaktini, y = after_stat(count)/sum(after_stat(count)))) +
  geom_pointrange(aes(x = kontaktini,y = dpois(kontaktini, mean(kontaktini)),
                      ymin = 0,ymax = dpois(kontaktini, mean(kontaktini))),colour = "red") +
  labs(title = "Poisson fit",y = "Probability")

nbfit <- fitdist(cat_l2b$kontaktini, "nbinom")

kontaktini_p2 <- ggplot(cat_l2b) +
  geom_bar(aes(x = kontaktini, y = after_stat(count)/sum(after_stat(count)))) +
  geom_pointrange(aes(x = kontaktini,y = dnbinom(kontaktini,
                                          size = nbfit$estimate[1],mu = nbfit$estimate[2]),
                      ymin = 0,ymax = dnbinom(kontaktini,size = nbfit$estimate[1],
                                         mu = nbfit$estimate[2])),colour = "blue") +
  labs(title = "Negative binomial fit",y = "Probability")

kontaktini_p1 / kontaktini_p2

#rechnen, welches Modell am besten:

kontaktini_poisson <- glm(kontaktini ~ umfeld*zeit, family = poisson, data = cat_l2b)
kontaktini_nb <- glm.nb(kontaktini ~ umfeld*zeit, data = cat_l2b)
#kontaktini_zip <- gamlss(kontaktini ~ umfeld*zeit, sigma.formula =  umfeld*zeit,family = ZIP, data = na.omit(cat_l2b))
#kontaktini_zip <- gamlss(kontaktini ~ umfeld*zeit, sigma.formula =  umfeld*zeit,family = ZIP, data = miss)

#kontaktini_zinbi <- gamlss(kontaktini ~ umfeld*zeit, nu.formula = umfeld*zeit,family = ZINBI, data = na.omit(cat_l2b),control = gamlss.control(n.cyc = 200)) #standard ist 20 - model has

#läuft nicht aufgrund von NA

AIC(kontaktini_poisson,kontaktini_nb)

                  df      AIC
kontaktini_poisson 10 3490.795
kontaktini_nb      11 2324.042

BIC(kontaktini_poisson,kontaktini_nb)

                  df      BIC
kontaktini_poisson 10 3530.933
kontaktini_nb      11 2368.193

miss <- cat_l2 %>% dplyr::filter(is.na(kontaktini))
```

Modelle

```
# GLMM für negative Binomial

#g,p,nb1 und nb2 vergleichen und schauen welches am besten

ini_glmm_g <- glmmTMB(kontaktini ~ umfeld* zeit + (1 | versuch),
             data = cat_l2)

ini_glmm_p <- glmmTMB(kontaktini ~ umfeld* zeit + (1 | versuch),
             data = cat_l2,family=poisson)

# zero inflated poisson zip
ini_glmm_zip <- glmmTMB(kontaktini ~ umfeld* zeit + (1 | versuch),
             data = cat_l2,family=poisson,ziformula = ~1)

# bestes Modell
ini_glmm_nb1 <- glmmTMB(kontaktini ~ umfeld* zeit + (1 | versuch),
             data = cat_l2,family=nbinom1)

ini_glmm_nb2 <- glmmTMB(kontaktini ~ umfeld* zeit + (1 | versuch),
             data = cat_l2,family=nbinom2)

ini_glmm_znb1 <- glmmTMB(kontaktini ~ umfeld* zeit + (1 | versuch),
             data = cat_l2,family=nbinom1, ziformula = ~1)

summary(ini_glmm_zip)

 Family: poisson  ( log )
Formula:          kontaktini ~ umfeld * zeit + (1 | versuch)
Zero inflation:              ~1
Data: cat_l2

     AIC      BIC   logLik deviance df.resid
      NA       NA       NA       NA      397

Random effects:

Conditional model:
 Groups  Name        Variance Std.Dev.
 versuch (Intercept) 0.2773   0.5266
Number of obs: 409, groups:  versuch, 82

Conditional model:
                           Estimate Std. Error z value Pr(>|z|)
(Intercept)                 2.03577        NaN     NaN      NaN
umfeldAnimal Shelter        0.45756    0.16670   2.745  0.00605 **
zeit2                      -0.15406    0.04813  -3.201  0.00137 **
zeit3                      -0.36577    0.05862  -6.240 4.38e-10 ***
zeit4                      -0.51032    0.03826 -13.337  < 2e-16 ***
zeit5                      -0.27568    0.06209  -4.440 9.00e-06 ***
umfeldAnimal Shelter:zeit2  0.21920    0.11657   1.880  0.06006 .
umfeldAnimal Shelter:zeit3 -0.06191    0.13777  -0.449  0.65314
umfeldAnimal Shelter:zeit4 -0.19948    0.16106  -1.239  0.21551
umfeldAnimal Shelter:zeit5 -0.18182    0.13786  -1.319  0.18721
---
Signif. codes:  0 '***' 0.001 '**' 0.01 '*' 0.05 '.' 0.1 ' ' 1

Zero-inflation model:
            Estimate Std. Error z value Pr(>|z|)
(Intercept) -1.37944    0.07757  -17.78   <2e-16 ***
---
Signif. codes:  0 '***' 0.001 '**' 0.01 '*' 0.05 '.' 0.1 ' ' 1

summary(ini_glmm_nb1)

 Family: nbinom1  ( log )
Formula:          kontaktini ~ umfeld * zeit + (1 | versuch)
Data: cat_l2

     AIC      BIC   logLik deviance df.resid
  2212.4   2260.6  -1094.2   2188.4      397

Random effects:

Conditional model:
 Groups  Name        Variance Std.Dev.
 versuch (Intercept) 0.4781   0.6915
Number of obs: 409, groups:  versuch, 82

Dispersion parameter for nbinom1 family (): 2.93

Conditional model:
                           Estimate Std. Error z value Pr(>|z|)
(Intercept)                 1.80756    0.12411  14.564  < 2e-16 ***
umfeldAnimal Shelter        0.32841    0.26769   1.227   0.2199
zeit2                      -0.06260    0.12126  -0.516   0.6057
zeit3                      -0.31727    0.12949  -2.450   0.0143 *
zeit4                      -0.71281    0.14596  -4.884 1.04e-06 ***
zeit5                      -0.55569    0.14176  -3.920 8.86e-05 ***
umfeldAnimal Shelter:zeit2 -0.06194    0.24159  -0.256   0.7977
umfeldAnimal Shelter:zeit3 -0.32980    0.27478  -1.200   0.2300
umfeldAnimal Shelter:zeit4 -0.40692    0.33762  -1.205   0.2281
umfeldAnimal Shelter:zeit5  0.02828    0.26870   0.105   0.9162
---
Signif. codes:  0 '***' 0.001 '**' 0.01 '*' 0.05 '.' 0.1 ' ' 1

anova(ini_glmm_g,ini_glmm_p,ini_glmm_nb1,ini_glmm_nb2,ini_glmm_znb1)

Data: cat_l2
Models:
ini_glmm_p: kontaktini ~ umfeld * zeit + (1 | versuch), zi=~0, disp=~1
ini_glmm_g: kontaktini ~ umfeld * zeit + (1 | versuch), zi=~0, disp=~1
ini_glmm_nb1: kontaktini ~ umfeld * zeit + (1 | versuch), zi=~0, disp=~1
ini_glmm_nb2: kontaktini ~ umfeld * zeit + (1 | versuch), zi=~0, disp=~1
ini_glmm_znb1: kontaktini ~ umfeld * zeit + (1 | versuch), zi=~1, disp=~1
              Df    AIC    BIC  logLik deviance   Chisq Chi Df Pr(>Chisq)
ini_glmm_p    11 2589.2 2633.4 -1283.6   2567.2
ini_glmm_g    12 2560.5 2608.7 -1268.2   2536.5  30.701      1   3.01e-08 ***
ini_glmm_nb1  12 2212.4 2260.6 -1094.2   2188.4 348.112      0  < 2.2e-16 ***
ini_glmm_nb2  12 2256.0 2304.1 -1116.0   2232.0   0.000      0          1
ini_glmm_znb1 13                                              1
---
Signif. codes:  0 '***' 0.001 '**' 0.01 '*' 0.05 '.' 0.1 ' ' 1

# p-Werte extra ausgeben

parameters(ini_glmm_znb1)

# Fixed Effects (Count Model)

Parameter                          | Log-Mean |   SE |         95% CI |     z |      p
--------------------------------------------------------------------------------------
(Intercept)                        |     1.89 | 0.11 | [ 1.68,  2.10] | 17.85 | < .001
umfeld [Animal Shelter]            |     0.40 | 0.55 | [-0.68,  1.48] |  0.73 | 0.466
zeit [2]                           |    -0.14 | 0.14 | [-0.41,  0.13] | -1.01 | 0.314
zeit [3]                           |    -0.37 | 0.21 | [-0.78,  0.03] | -1.81 | 0.070
zeit [4]                           |    -0.67 | 0.24 | [-1.15, -0.19] | -2.72 | 0.007
zeit [5]                           |    -0.49 | 0.24 | [-0.95, -0.02] | -2.03 | 0.042
umfeld [Animal Shelter] × zeit [2] |     0.10 |      |                |       |
umfeld [Animal Shelter] × zeit [3] |    -0.13 | 0.09 | [-0.30,  0.03] | -1.58 | 0.113
umfeld [Animal Shelter] × zeit [4] |    -0.37 | 0.13 | [-0.62, -0.12] | -2.86 | 0.004
umfeld [Animal Shelter] × zeit [5] |     0.09 | 0.36 | [-0.61,  0.79] |  0.25 | 0.802

# Fixed Effects (Zero-Inflation Component)

Parameter   | Log-Odds |   SE |         95% CI |     z |     p
-------------------------------------------------------------
(Intercept) |    -2.36 | 0.95 | [-4.22, -0.51] | -2.50 | 0.013

# Dispersion

Parameter   | Coefficient |       95% CI
----------------------------------------
(Intercept) |        2.11 | [1.19, 3.74]

# Random Effects Variances

Parameter               | Coefficient |       95% CI
----------------------------------------------------
SD (Intercept: versuch) |        0.65 | [0.63, 0.66]
```

Effektstärke

```
#Cohen's d berechnen

# Get all variance components
reffects_ini <- broom.mixed::tidy(ini_glmm_nb1, effects = c("ran_pars"), scale="vcov")

# Calculate denominator (taking into account all relevant variance components)
denominator_ini <- sqrt(sum(reffects_ini$estimate))

# Get fixed effects estimates
fixed_effects <- summary(ini_glmm_nb1)$coef$cond[, 1]

# Calculate Cohen's d
cohend_ini <- data.frame(
  CohensD_ini = fixed_effects[c(2,3,4,5,6,7,8,9,10)]/denominator_ini
)
cohend_ini

                           CohensD_ini
umfeldAnimal Shelter        0.47494584
zeit2                      -0.09052811
zeit3                      -0.45883174
zeit4                      -1.03087293
zeit5                      -0.80363426
umfeldAnimal Shelter:zeit2 -0.08957564
umfeldAnimal Shelter:zeit3 -0.47695644
umfeldAnimal Shelter:zeit4 -0.58848819
umfeldAnimal Shelter:zeit5  0.04089242

###
#wobei die Frage ist, ob man die Formel bei GLMM einfach so übernehmen darf. Das müsst ich selbst recherchieren
```

Grafik

```
#estimated marginal means
# geschätzen Randmittel

emm_ini_m1 <- emmeans(ini_glmm_nb1, "umfeld", by="zeit")
emm_ini_m1b <- emmeans(ini_glmm_nb1, "zeit", by="umfeld")
em_df_ini_m1<- data.frame(emm_ini_m1)

ggplot(em_df_ini_m1, aes(x = zeit, y = emmean, group=umfeld, color=umfeld))+
  geom_line() +
    geom_point(aes(shape=umfeld))+
  geom_errorbar(aes(ymin=asymp.LCL, ymax=asymp.UCL), width=.4,
                position=position_dodge(0.01)) +
  scale_colour_manual(values=c("#D700FF","#00C0FA"))+
  scale_y_continuous(limits=c(0,5),breaks=seq(0,5,1)) +
  theme_bw() +
  labs(x = "Video Sequence (No)",
       y = "Initialisation Behaviour (number)",
       color = "Living Environment",
       shape = "Living Environment")

#Problem mit dem Ausdruck für die Confidenzintervalle:
#geom_errorbar(aes(ymin=lower.CL, ymax=upper.CL), width=.4, position=position_dodge(0.01))
# ymin=asymp.LCL, ymax=asymp.UCL anders benannt bei GLMM
```

```
##############################
#Zurück transformieren der Erwartungswerte (GLMM immer mit log)


em_df_ini_m1 <- em_df_ini_m1 %>%
  dplyr::mutate(emmean_trans = exp(emmean),
                asymp.LCL_trans = exp(asymp.LCL),
                asymp.UCL_trans = exp(asymp.UCL))

ini_g1 <-ggplot(em_df_ini_m1, aes(x = zeit, y = emmean_trans, group = umfeld, color = umfeld)) +
  geom_line() +
  geom_point(aes(shape = umfeld)) +
  geom_errorbar(aes(ymin = asymp.LCL_trans, ymax = asymp.UCL_trans), width = 0.2) +
  scale_colour_manual(values = c("#D700FF", "#00C0FA")) +
  scale_y_continuous(limits = c(0, 25), breaks = seq(0, 25, 5)) +
  theme_bw() +
  labs(x = "Video Sequence (No)",
       y = "Initial Behaviour (number)",
       color = "Living Environment",
       shape = "Living Environment")

ini_g1
```

```
ggplot(em_df_ini_m1, aes(x = zeit, y = emmean_trans, group = umfeld, color = umfeld)) +
  # Füge ein Band für das Konfidenzintervall hinzu
  geom_ribbon(aes(ymin = asymp.LCL_trans, ymax = asymp.UCL_trans, fill = umfeld),
              alpha = 0.2, color = NA) +
  geom_line(linewidth = 1) +
  geom_point(aes(shape = umfeld), size = 3) +
  # Verbesserte Farbpalette mit benannten Beschriftungen
  scale_colour_manual(values = c("#D700FF", "#00C0FA")) +
  scale_fill_manual(values = c("#D700FF", "#00C0FA")) +
  # Y-Achse anpassen
  scale_y_continuous(limits = c(0, 25),
                     breaks = seq(0, 25, 5),
                     expand = c(0, 0)) +
  # X-Achse anpassen
  scale_x_discrete(expand = c(0.05, 0.05)) +
  # Theme anpassen
  theme_bw() +
  theme(
    panel.grid.minor = element_blank(),
    legend.position = "bottom",
    axis.title = element_text(size = 12, face = "bold"),
    axis.text = element_text(size = 10),
    legend.title = element_text(size = 11, face = "bold"),
    legend.text = element_text(size = 10)
  ) +
  # Beschriftungen
  labs(x = "Video Sequence (No)",
       y = "Initialisation Behaviour (number)",
       color = "Living Environment",
       shape = "Living Environment")
```

Paarweise Vergleiche

```
pairs(emmeans(ini_glmm_nb1, "umfeld", by="zeit") , adjust="dunnet")
```

```
zeit = 1:
 contrast                       estimate    SE  df z.ratio p.value
 Private Home - Animal Shelter -0.32841 0.268 Inf  -1.227  0.2199

zeit = 2:
 contrast                       estimate    SE  df z.ratio p.value
 Private Home - Animal Shelter -0.26647 0.271 Inf  -0.984  0.3249

zeit = 3:
 contrast                       estimate    SE  df z.ratio p.value
 Private Home - Animal Shelter  0.00139 0.299 Inf   0.005  0.9963

zeit = 4:
 contrast                       estimate    SE  df z.ratio p.value
 Private Home - Animal Shelter  0.07851 0.354 Inf   0.222  0.8245

zeit = 5:
 contrast                       estimate    SE  df z.ratio p.value
 Private Home - Animal Shelter -0.35669 0.297 Inf  -1.203  0.2290

Results are given on the log (not the response) scale.
```

```
pairs(emmeans(ini_glmm_nb1, "zeit", by="umfeld") , adjust="dunnet")
```

```
umfeld = Private Home:
 contrast      estimate    SE  df z.ratio p.value
 zeit1 - zeit2   0.0626 0.121 Inf   0.516  0.9872
 zeit1 - zeit3   0.3173 0.129 Inf   2.450  0.1055
 zeit1 - zeit4   0.7128 0.146 Inf   4.884  <.0001
 zeit1 - zeit5   0.5557 0.142 Inf   3.920  0.0008
 zeit2 - zeit3   0.2547 0.130 Inf   1.956  0.2995
 zeit2 - zeit4   0.6502 0.146 Inf   4.448  0.0001
 zeit2 - zeit5   0.4931 0.142 Inf   3.461  0.0049
 zeit3 - zeit4   0.3955 0.153 Inf   2.590  0.0741
 zeit3 - zeit5   0.2384 0.149 Inf   1.604  0.5137
 zeit4 - zeit5  -0.1571 0.163 Inf  -0.967  0.8815

umfeld = Animal Shelter:
 contrast      estimate    SE  df z.ratio p.value
 zeit1 - zeit2   0.1245 0.209 Inf   0.596  0.9781
 zeit1 - zeit3   0.6471 0.242 Inf   2.670  0.0599
 zeit1 - zeit4   1.1197 0.304 Inf   3.678  0.0022
 zeit1 - zeit5   0.5274 0.228 Inf   2.310  0.1465
 zeit2 - zeit3   0.5225 0.246 Inf   2.124  0.2179
 zeit2 - zeit4   0.9952 0.309 Inf   3.224  0.0112
 zeit2 - zeit5   0.4029 0.236 Inf   1.704  0.4485
 zeit3 - zeit4   0.4727 0.330 Inf   1.433  0.6265
 zeit3 - zeit5  -0.1197 0.266 Inf  -0.450  0.9924
 zeit4 - zeit5  -0.5923 0.320 Inf  -1.848  0.3597

Results are given on the log (not the response) scale.
P value adjustment: dunnettx method for 10 tests
```

```
confint(pairs(emmeans(ini_glmm_nb1,~umfeld)))
```

```
 contrast                       estimate    SE  df asymp.LCL asymp.UCL
 Private Home - Animal Shelter   -0.174 0.228 Inf     -0.62     0.272

Results are averaged over the levels of: zeit
Results are given on the log (not the response) scale.
Confidence level used: 0.95
```

```
confint(pairs(emmeans(ini_glmm_nb1,~zeit)))
```

```
 contrast      estimate    SE  df asymp.LCL asymp.UCL
 zeit1 - zeit2   0.0936 0.121 Inf  -0.23554     0.423
 zeit1 - zeit3   0.4822 0.137 Inf   0.10746     0.857
 zeit1 - zeit4   0.9163 0.169 Inf   0.45579     1.377
 zeit1 - zeit5   0.5415 0.134 Inf   0.17491     0.908
 zeit2 - zeit3   0.3886 0.139 Inf   0.00888     0.768
 zeit2 - zeit4   0.8227 0.171 Inf   0.35676     1.289
 zeit2 - zeit5   0.4480 0.138 Inf   0.07183     0.824
 zeit3 - zeit4   0.4341 0.182 Inf  -0.06176     0.930
 zeit3 - zeit5   0.0594 0.152 Inf  -0.35594     0.475
 zeit4 - zeit5  -0.3747 0.180 Inf  -0.86453     0.115

Results are averaged over the levels of: umfeld
Results are given on the log (not the response) scale.
Confidence level used: 0.95
Conf-level adjustment: tukey method for comparing a family of 5 estimates
```

Voraussetzung

```
# Poisson o. neg Binomial

# Struktur der zufälligen Effekte auf Normalverteilung prüfen
# DHARMa - prüft automatisch Residuen auf Normalverteilung & Abweichungen


q_unit <- DHARMa::simulateResiduals(ini_glmm_nb1)
plot(q_unit)
```

```
#Ausreißer
testOutliers(q_unit)
```

```
    DHARMa bootstrapped outlier test

data:  q_unit
outliers at both margin(s) = 0, observations = 409, p-value = 0.28
alternative hypothesis: two.sided
 percent confidence interval:
 0.00000000 0.01711491
sample estimates:
outlier frequency (expected: 0.00577017114914425 )
                                                 0
```

Vermeidendes Verhalten (kontaktver)

Verteilungen

```
#
# Zähldaten: Poisson Regression
# Voraussetzung: Erwartungswert = Varianz
# --> Praxis oft nicht so
# Alternative: Negativ binomial

ggplot(cat_l2, aes(x=kontaktver)) +
  geom_histogram() +
  facet_wrap(~zeit)
```

```
#Graphischer Vergleich Poisson/negbin
# person 8 ausschließen

cat_l2b <- cat_l2 %>% dplyr::filter(!is.na(kontaktver))

kontaktver_p1 <- ggplot(cat_l2b) +
  geom_bar(aes(x = kontaktver, y = after_stat(count)/sum(after_stat(count)))) +
  geom_pointrange(aes(x = kontaktver,y = dpois(kontaktver, mean(kontaktver)),
                      ymin = 0,ymax = dpois(kontaktver, mean(kontaktver))),colour = "red") +
  labs(title = "Poisson fit",y = "Probability")

nbfit <- fitdist(cat_l2b$kontaktver, "nbinom")

kontaktver_p2 <- ggplot(cat_l2b) +
  geom_bar(aes(x = kontaktver, y = after_stat(count)/sum(after_stat(count)))) +
  geom_pointrange(aes(x = kontaktver,y = dnbinom(kontaktver,
                                               size = nbfit$estimate[1],mu = nbfit$estimate[2]),
                      ymin = 0,ymax = dnbinom(kontaktver,size = nbfit$estimate[1],
                                              mu = nbfit$estimate[2])),colour = "blue") +
  labs(title = "Negative binomial fit",y = "Probability")

kontaktver_p1 / kontaktver_p2
```

```
#rechnen, welches Modell am besten:

kontaktver_poisson <- glm(kontaktver ~ umfeld*zeit, family = poisson, data = cat_l2b)
kontaktver_nb <- glm.nb(kontaktver ~ umfeld*zeit, data = cat_l2b)
#kontaktver_zip <- gamlss(kontaktver ~ umfeld*zeit, sigma.formula =  umfeld*zeit,family = ZIP, data = cat_l2b)
#kontaktver_zinbi <- gamlss(kontaktver ~ umfeld*zeit, nu.formula = umfeld*zeit,family = ZINBI, data = cat_l2b,control = gamlss.control(n.cyc = 200)) #standard ist 20 - model has not conv

AIC(kontaktver_poisson,kontaktver_nb)
```

```
                   df      AIC
kontaktver_poisson 10 1420.701
kontaktver_nb      11 1186.298
```

```
BIC(kontaktver_poisson,kontaktver_nb)
```

```
                   df      BIC
kontaktver_poisson 10 1460.838
kontaktver_nb      11 1230.449
```

```
miss <- cat_l2 %>% dplyr::filter(is.na(kontaktver))


#glmer
```

Modelle

```
# GLMM für negative Binomial

#g,p,nb1 und nb2 vergleichen und schauen welches am besten


ver_glmm_g <- glmmTMB(kontaktver ~ umfeld* zeit + (1 | versuch),
              data = cat_l2)

ver_glmm_p <- glmmTMB(kontaktver ~ umfeld* zeit + (1 | versuch),
              data = cat_l2,family=poisson)

# zero inflated poisson zip
ver_glmm_zip <- glmmTMB(kontaktver ~ umfeld* zeit + (1 | versuch),
              data = cat_l2,family=poisson,ziformula = ~1)

# bestes Modell
ver_glmm_nb1 <- glmmTMB(kontaktver ~ umfeld* zeit + (1 | versuch),
              data = cat_l2,family=nbinom1)

ver_glmm_nb2 <- glmmTMB(kontaktver ~ umfeld* zeit + (1 | versuch),
              data = cat_l2,family=nbinom2)


ver_glmm_znb1 <- glmmTMB(kontaktver ~ umfeld* zeit + (1 | versuch),
              data = cat_l2,family=nbinom1, ziformula = ~1)



summary(ver_glmm_nb1)
```

```
 Family: nbinom1  ( log )
Formula:          kontaktver ~ umfeld * zeit + (1 | versuch)
Data: cat_l2

     AIC      BIC   logLik deviance df.resid
  1105.0   1153.2   -540.5   1081.0      397

Random effects:

Conditional model:
 Groups  Name        Variance Std.Dev.
 versuch (Intercept) 1.158    1.076
Number of obs: 409, groups:  versuch, 82

Dispersion parameter for nbinom1 family (): 0.769

Conditional model:
                           Estimate Std. Error z value Pr(>|z|)
(Intercept)                 -0.4663     0.2215  -2.105   0.0353 *
umfeldAnimal Shelter         0.6841     0.4378   1.563   0.1181
zeit2                        0.1892     0.2117   0.894   0.3714
zeit3                        0.1230     0.2135   0.576   0.5646
zeit4                       -0.1328     0.2286  -0.581   0.5611
zeit5                       -0.2125     0.2311  -0.920   0.3578
umfeldAnimal Shelter:zeit2  -0.7598     0.4363  -1.742   0.0816 .
umfeldAnimal Shelter:zeit3  -0.4322     0.4119  -1.049   0.2941
umfeldAnimal Shelter:zeit4  -0.9396     0.5190  -1.810   0.0702 .
umfeldAnimal Shelter:zeit5  -0.2553     0.4252  -0.601   0.5481
---
Signif. codes:  0 '***' 0.001 '**' 0.01 '*' 0.05 '.' 0.1 ' ' 1
```

```
anova(ver_glmm_g,ver_glmm_p,ver_glmm_nb1,ver_glmm_znb1)
```

```
Data: cat_l2
Models:
ver_glmm_p: kontaktver ~ umfeld * zeit + (1 | versuch), zi=~0, disp=~1
ver_glmm_g: kontaktver ~ umfeld * zeit + (1 | versuch), zi=~0, disp=~1
ver_glmm_nb1: kontaktver ~ umfeld * zeit + (1 | versuch), zi=~0, disp=~1
ver_glmm_znb1: kontaktver ~ umfeld * zeit + (1 | versuch), zi=~1, disp=~1
              Df    AIC    BIC  logLik deviance    Chisq Chi Df Pr(>Chisq)
ver_glmm_p    11 1143.5 1187.7 -560.75   1121.5
ver_glmm_g    12 1621.7 1669.8 -798.84   1597.7   0.0000      1     1.0000
ver_glmm_nb1  12 1105.0 1153.2 -540.51   1081.0 516.6540      0     <2e-16 ***
ver_glmm_znb1 13 1105.9 1158.0 -539.93   1079.9   1.1597      1     0.2815
---
Signif. codes:  0 '***' 0.001 '**' 0.01 '*' 0.05 '.' 0.1 ' ' 1
```

```
# p-Werte extra ausgeben

parameters(ver_glmm_nb1)
```

```
# Fixed Effects

Parameter                          | Log-Mean |   SE |         95% CI |     z |     p
-------------------------------------------------------------------------------------
(Intercept)                        |    -0.47 | 0.22 | [-0.90, -0.03] | -2.11 | 0.035
umfeld [Animal Shelter]            |     0.68 | 0.44 | [-0.17,  1.54] |  1.56 | 0.118
zeit [2]                           |     0.19 | 0.21 | [-0.23,  0.60] |  0.89 | 0.371
zeit [3]                           |     0.12 | 0.21 | [-0.30,  0.54] |  0.58 | 0.565
zeit [4]                           |    -0.13 | 0.23 | [-0.58,  0.32] | -0.58 | 0.561
zeit [5]                           |    -0.21 | 0.23 | [-0.67,  0.24] | -0.92 | 0.358
umfeld [Animal Shelter] × zeit [2] |    -0.76 | 0.44 | [-1.61,  0.10] | -1.74 | 0.082
umfeld [Animal Shelter] × zeit [3] |    -0.43 | 0.41 | [-1.24,  0.38] | -1.05 | 0.294
umfeld [Animal Shelter] × zeit [4] |    -0.94 | 0.52 | [-1.96,  0.08] | -1.81 | 0.070
umfeld [Animal Shelter] × zeit [5] |    -0.26 | 0.43 | [-1.09,  0.58] | -0.60 | 0.548

# Dispersion

Parameter   | Coefficient |       95% CI
----------------------------------------
(Intercept) |        0.77 | [0.48, 1.24]

# Random Effects Variances

Parameter               | Coefficient |       95% CI
----------------------------------------------------
SD (Intercept: versuch) |        1.08 | [0.83, 1.39]
```

Effektstärke

```
#Cohen's d berechnen

# Get all variance components
reffects_ver <- broom.mixed::tidy(ver_glmm_nb1, effects = c("ran_pars"), scale="vcov")

# Calculate denominator (taking into account all relevant variance components)
denominator_ver <- sqrt(sum(reffects_ver$estimate))

# Get fixed effects estimates
fixed_effects <- summary(ver_glmm_nb1)$coef$cond[, 1]

# Calculate Cohen's d
cohend_ver <- data.frame(
  CohensD_ver = fixed_effects[c(2,3,4,5,6,7,8,9,10)]/denominator_ver
)
cohend_ver
```

```
                           CohensD_ver
umfeldAnimal Shelter         0.6357595
zeit2                        0.1758410
zeit3                        0.1142905
zeit4                       -0.1234492
zeit5                       -0.1975264
umfeldAnimal Shelter:zeit2  -0.7061215
umfeldAnimal Shelter:zeit3  -0.4016424
umfeldAnimal Shelter:zeit4  -0.8732296
umfeldAnimal Shelter:zeit5  -0.2372937
```

```
###
#wobei die Frage ist, ob man die Formel bei GLMM einfach so übernehmen darf. Das müsst ich selbst recherchieren
```

Grafik

```
#estimated marginal means
# geschätzen Randmittel

emm_ver_m1 <- emmeans(ver_glmm_nb1, "umfeld", by="zeit")
emm_ver_m1b <- emmeans(ver_glmm_nb1, "zeit", by="umfeld")
em_df_ver_m1<- data.frame(emm_ver_m1)

ggplot(em_df_ver_m1, aes(x = zeit, y = emmean, group=umfeld, color=umfeld))+
  geom_line() +
    geom_point(aes(shape=umfeld))+
    geom_errorbar(aes(ymin=asymp.LCL, ymax=asymp.UCL), width=.4,
                position=position_dodge(0.01)) +
  scale_colour_manual(values=c("#D700FF","#00C0FA"))+
  scale_y_continuous(limits=c(-5,5),breaks=seq(0,5,1)) +
  theme_bw() +
  labs(x = "Video Sequence (No)",
       y = "Avoidance Behvaiour (number)",
       color = "Living Environment",
       shape = "Living Environment")
```

```
#Problem mit dem Ausdruck für die Confidenzintervalle:
#geom_errorbar(aes(ymin=lower.CL, ymax=upper.CL), width=.4, position=position_dodge(0.01))

##############################
#Zurück transformieren der Erwartungswerte (GLMM immer mit log)


em_df_ver_m1 <- em_df_ver_m1 %>%
  dplyr::mutate(emmean_trans = exp(emmean),
                asymp.LCL_trans = exp(asymp.LCL),
                asymp.UCL_trans = exp(asymp.UCL))

ver_g1 <-ggplot(em_df_ver_m1, aes(x = zeit, y = emmean_trans, group = umfeld, color = umfeld)) +
  geom_line() +
  geom_point(aes(shape = umfeld)) +
  geom_errorbar(aes(ymin = asymp.LCL_trans, ymax = asymp.UCL_trans), width = 0.2) +
  scale_colour_manual(values = c("#D700FF", "#00C0FA")) +
  scale_y_continuous(limits = c(0, 5), breaks = seq(0, 5, 1)) +
  theme_bw() +
  labs(x = "Video Sequence (No)",
       y = "Avoidance Behaviour (number)",
       color = "Living Environment",
       shape = "Living Environment")

ver_g1
```

```
ggplot(em_df_ver_m1, aes(x = zeit, y = emmean_trans, group = umfeld, color = umfeld)) +
  # Füge ein Band für das Konfidenzintervall hinzu
  geom_ribbon(aes(ymin = asymp.LCL_trans, ymax = asymp.UCL_trans, fill = umfeld),
              alpha = 0.2, color = NA) +
  geom_line(linewidth = 1) +
  geom_point(aes(shape = umfeld), size = 3) +
  # Verbesserte Farbpalette mit benannten Beschriftungen
  scale_colour_manual(values = c("#D700FF", "#00C0FA")) +
  scale_fill_manual(values = c("#D700FF", "#00C0FA")) +
  # Y-Achse anpassen
  scale_y_continuous(limits = c(0, 5),
                     breaks = seq(0, 5, 0.5),
                     expand = c(0, 0)) +
  # X-Achse anpassen
  scale_x_discrete(expand = c(0.05, 0.05)) +
  # Theme anpassen
  theme_bw() +
  theme(
    panel.grid.minor = element_blank(),
    legend.position = "bottom",
    axis.title = element_text(size = 12, face = "bold"),
    axis.text = element_text(size = 10),
    legend.title = element_text(size = 11, face = "bold"),
    legend.text = element_text(size = 10)
  ) +
  # Beschriftungen
  labs(x = "Video Sequence (No)",
       y = "Avoidance Behaviour (number)",
       color = "Living Environment",
       shape = "Living Environment")
```

Paarweise Vergleiche

```
pairs(emmeans(ver_glmm_nb1, "umfeld", by="zeit") , adjust="dunnet")
```

```
zeit = 1:
 contrast                       estimate    SE  df z.ratio p.value
 Private Home - Animal Shelter  -0.6841 0.438 Inf  -1.563  0.1181

zeit = 2:
 contrast                       estimate    SE  df z.ratio p.value
 Private Home - Animal Shelter   0.0757 0.473 Inf   0.160  0.8727

zeit = 3:
 contrast                       estimate    SE  df z.ratio p.value
 Private Home - Animal Shelter  -0.2519 0.452 Inf  -0.557  0.5774

zeit = 4:
 contrast                       estimate    SE  df z.ratio p.value
 Private Home - Animal Shelter   0.2555 0.547 Inf   0.467  0.6402

zeit = 5:
 contrast                       estimate    SE  df z.ratio p.value
 Private Home - Animal Shelter  -0.4288 0.466 Inf  -0.920  0.3578

Results are given on the log (not the response) scale.
```

```
pairs(emmeans(ver_glmm_nb1, "zeit", by="umfeld") , adjust="dunnet")
```

```
umfeld = Private Home:
 contrast      estimate    SE  df z.ratio p.value
 zeit1 - zeit2  -0.1892 0.212 Inf  -0.894  0.9084
 zeit1 - zeit3  -0.1230 0.214 Inf  -0.576  0.9807
 zeit1 - zeit4   0.1328 0.229 Inf   0.581  0.9801
 zeit1 - zeit5   0.2125 0.231 Inf   0.920  0.8993
 zeit2 - zeit3   0.0662 0.200 Inf   0.332  0.9977
 zeit2 - zeit4   0.3220 0.216 Inf   1.492  0.5878
 zeit2 - zeit5   0.4018 0.218 Inf   1.844  0.3623
 zeit3 - zeit4   0.2558 0.218 Inf   1.174  0.7834
 zeit3 - zeit5   0.3355 0.220 Inf   1.526  0.5649
 zeit4 - zeit5   0.0797 0.234 Inf   0.340  0.9974

umfeld = Animal Shelter:
 contrast      estimate    SE  df z.ratio p.value
 zeit1 - zeit2   0.5706 0.382 Inf   1.495  0.5857
 zeit1 - zeit3   0.3092 0.353 Inf   0.877  0.9140
 zeit1 - zeit4   1.0725 0.466 Inf   2.303  0.1488
 zeit1 - zeit5   0.4679 0.358 Inf   1.306  0.7068
 zeit2 - zeit3  -0.2614 0.401 Inf  -0.652  0.9697
 zeit2 - zeit4   0.5019 0.509 Inf   0.987  0.8733
 zeit2 - zeit5  -0.1027 0.411 Inf  -0.250  0.9992
 zeit3 - zeit4   0.7633 0.481 Inf   1.588  0.5239
 zeit3 - zeit5   0.1587 0.381 Inf   0.417  0.9943
 zeit4 - zeit5  -0.6046 0.485 Inf  -1.247  0.7421

Results are given on the log (not the response) scale.
P value adjustment: dunnettx method for 10 tests
```

```
confint(pairs(emmeans(ver_glmm_nb1,~umfeld)))
```

```
 contrast                       estimate    SE  df asymp.LCL asymp.UCL
 Private Home - Animal Shelter   -0.207 0.368 Inf    -0.928     0.515

Results are averaged over the levels of: zeit
Results are given on the log (not the response) scale.
Confidence level used: 0.95
```

```
confint(pairs(emmeans(ver_glmm_nb1,~zeit)))
```

```
 contrast      estimate    SE  df asymp.LCL asymp.UCL
 zeit1 - zeit2   0.1907 0.218 Inf    -0.405     0.786
 zeit1 - zeit3   0.0931 0.206 Inf    -0.469     0.656
 zeit1 - zeit4   0.6026 0.259 Inf    -0.105     1.310
 zeit1 - zeit5   0.3402 0.214 Inf    -0.243     0.923
 zeit2 - zeit3  -0.0976 0.224 Inf    -0.709     0.513
 zeit2 - zeit4   0.4120 0.276 Inf    -0.342     1.166
 zeit2 - zeit5   0.1495 0.233 Inf    -0.485     0.784
 zeit3 - zeit4   0.5095 0.264 Inf    -0.210     1.229
 zeit3 - zeit5   0.2471 0.220 Inf    -0.353     0.847
 zeit4 - zeit5  -0.2624 0.270 Inf    -0.998     0.473

Results are averaged over the levels of: umfeld
Results are given on the log (not the response) scale.
Confidence level used: 0.95
Conf-level adjustment: tukey method for comparing a family of 5 estimates
```

Voraussetzungen

```
# Poisson o. neg Binomial

# Struktur der zufälligen Effekte auf Normalverteilung prüfen

# DHARMa - prüft automatisch Residuen auf Normalverteilung & Abweichungen
q_unit <- DHARMa::simulateResiduals(ver_glmm_nb1)
plot(q_unit)
```

```
#Ausreißer
testOutliers(q_unit)
```

```
    DHARMa bootstrapped outlier test

data:  q_unit
outliers at both margin(s) = 0, observations = 409, p-value = 0.62
alternative hypothesis: two.sided
 percent confidence interval:
 0.00000000 0.01839853
sample estimates:
outlier frequency (expected: 0.00552567237163814 )
                                                 0
```

Miauen (miauen)

Verteilungen

```
# table(subset2)

#
# Zähldaten: Poisson Regression
# Voraussetzung: Erwartungswert = Varianz
# --> Praxis oft nicht so
# Alternative: Negativ binomial

ggplot(cat_l2, aes(x=miauen)) +
  geom_histogram() +
  facet_wrap(~zeit)
```

```
#Graphischer Vergleich Poisson/negbin
# person 8 ausschließen

cat_l2b <- cat_l2 %>% dplyr::filter(!is.na(miauen))

miauen_p1 <- ggplot(cat_l2b) +
  geom_bar(aes(x = miauen, y = after_stat(count)/sum(after_stat(count)))) +
  geom_pointrange(aes(x = miauen,y = dpois(miauen, mean(miauen)),
                      ymin = 0,ymax = dpois(miauen, mean(miauen))),colour = "red") +
  labs(title = "Poisson fit",y = "Probability")

nbfit <- fitdist(cat_l2b$miauen, "nbinom")

miauen_p2 <- ggplot(cat_l2b) +
  geom_bar(aes(x = miauen, y = after_stat(count)/sum(after_stat(count)))) +
  geom_pointrange(aes(x = miauen,y = dnbinom(miauen,
                                             size = nbfit$estimate[1],mu = nbfit$estimate[2]),
                      ymin = 0,ymax = dnbinom(miauen,size = nbfit$estimate[1],
                                              mu = nbfit$estimate[2])),colour = "blue") +
  labs(title = "Negative binomial fit",y = "Probability")

miauen_p1 / miauen_p2
```

```
#rechnen, welches Modell am besten:

miauen_poisson <- glm(miauen ~ umfeld*zeit, family = poisson, data = cat_l2b)
miauen_nb <- glm.nb(miauen ~ umfeld*zeit, data = cat_l2b)
#miauen_zip <- gamlss(miauen ~ umfeld*zeit, sigma.formula =  umfeld*zeit,family = ZIP, data = cat_l2b)
#miauen_zinbi <- gamlss(miauen ~ umfeld*zeit, nu.formula = umfeld*zeit,family = ZINBI, data = cat_l2b,control = gamlss.control(n.cyc = 200)) #standard ist 20 - model has not converged be

AIC(miauen_poisson,miauen_nb)
```

```
               df      AIC
miauen_poisson 10 3655.031
miauen_nb      11 1043.796
```

```
BIC(miauen_poisson,miauen_nb)
```

```
               df      BIC
miauen_poisson 10 3695.192
miauen_nb      11 1087.974
```

```
miss <- cat_l2 %>% dplyr::filter(is.na(miauen))
```

Modelle

```
# GLMM für negative Binomial

#g,p,nb1 und nb2 vergleichen und schauen welches am besten


miauen_glmm_g <- glmmTMB(miauen~ umfeld* zeit + (1 | versuch),
              data = cat_l2)

miauen_glmm_p <- glmmTMB(miauen ~ umfeld* zeit + (1 | versuch),
              data = cat_l2,family=poisson)

# zero inflated poisson zip
miauen_glmm_zip <- glmmTMB(miauen ~ umfeld* zeit + (1 | versuch),
              data = cat_l2,family=poisson,ziformula = ~1)


miauen_glmm_nb1 <- glmmTMB(miauen ~ umfeld* zeit + (1 | versuch),
              data = cat_l2,family=nbinom1)

miauen_glmm_nb2 <- glmmTMB(miauen ~ umfeld* zeit + (1 | versuch),
              data = cat_l2,family=nbinom2)

# bestes Modell
miauen_glmm_znb1 <- glmmTMB(miauen ~ umfeld* zeit + (1 | versuch),
              data = cat_l2,family=nbinom1, ziformula = ~1)


summary(miauen_glmm_nb1)
```

```
 Family: nbinom1  ( log )
Formula:          miauen ~ umfeld * zeit + (1 | versuch)
Data: cat_l2

     AIC      BIC   logLik deviance df.resid
   930.4    978.6   -453.2    906.4      398

Random effects:

Conditional model:
 Groups  Name        Variance Std.Dev.
 versuch (Intercept) 3.557    1.886
Number of obs: 410, groups:  versuch, 82

Dispersion parameter for nbinom1 family (): 8.37

Conditional model:
                           Estimate Std. Error z value Pr(>|z|)
(Intercept)                -0.63010    0.40860  -1.542   0.1230
umfeldAnimal Shelter       -1.10519    0.89058  -1.241   0.2146
zeit2                       0.09087    0.27804   0.327   0.7438
zeit3                      -0.04795    0.29801  -0.161   0.8722
zeit4                      -0.66032    0.33879  -1.949   0.0513 .
zeit5                      -0.16366    0.29927  -0.547   0.5845
umfeldAnimal Shelter:zeit2 -0.15215    0.78329  -0.194   0.8460
umfeldAnimal Shelter:zeit3  0.18403    0.86818   0.212   0.8321
umfeldAnimal Shelter:zeit4  0.80324    0.75074   1.070   0.2847
umfeldAnimal Shelter:zeit5 -0.43861    0.84404  -0.520   0.6033
---
Signif. codes:  0 '***' 0.001 '**' 0.01 '*' 0.05 '.' 0.1 ' ' 1
```

```
anova(miauen_glmm_g,miauen_glmm_p,miauen_glmm_nb1,miauen_glmm_nb2, miauen_glmm_znb1)
```

```
Data: cat_l2
Models:
miauen_glmm_p: miauen ~ umfeld * zeit + (1 | versuch), zi=~0, disp=~1
miauen_glmm_g: miauen ~ umfeld * zeit + (1 | versuch), zi=~0, disp=~1
miauen_glmm_nb1: miauen ~ umfeld * zeit + (1 | versuch), zi=~0, disp=~1
miauen_glmm_nb2: miauen ~ umfeld * zeit + (1 | versuch), zi=~0, disp=~1
miauen_glmm_znb1: miauen ~ umfeld * zeit + (1 | versuch), zi=~1, disp=~1
                 Df     AIC     BIC   logLik deviance    Chisq Chi Df
miauen_glmm_p    11 1321.14 1365.32  -649.57  1299.14
miauen_glmm_g    12 2579.17 2627.37 -1277.59  2555.17    0.000      1
miauen_glmm_nb1  12  930.43  978.63  -453.22   906.43 1648.739      0
miauen_glmm_nb2  12  967.24 1015.43  -471.62   943.24    0.000      0
miauen_glmm_znb1 13  924.54  976.75  -449.27   898.54   44.694      1
                 Pr(>Chisq)
miauen_glmm_p
miauen_glmm_g             1
miauen_glmm_nb1   < 2.2e-16 ***
miauen_glmm_nb2           1
miauen_glmm_znb1  2.304e-11 ***
---
Signif. codes:  0 '***' 0.001 '**' 0.01 '*' 0.05 '.' 0.1 ' ' 1
```

```
#Hurdle Model

#w-keit, dass 0 eintritt mit einer logistsichen Regression
#anzahl der AV mit negbin

miauen_glmm_znb1a <- glmmTMB(miauen ~ umfeld*zeit + (1 | versuch),
              data = cat_l2,family=nbinom1, ziformula = ~umfeld*zeit)
miauen_glmm_znb1b <- glmmTMB(miauen ~ umfeld*zeit + (1 | versuch),
              data = cat_l2,family=nbinom1, ziformula = ~umfeld+zeit)
miauen_glmm_znb1c <- glmmTMB(miauen ~ umfeld*zeit + (1 | versuch),
              data = cat_l2,family=nbinom1, ziformula = ~umfeld)
miauen_glmm_znb1d <- glmmTMB(miauen ~ umfeld*zeit + (1 | versuch),
              data = cat_l2,family=nbinom1, ziformula = ~zeit)

anova(miauen_glmm_znb1,miauen_glmm_znb1a,miauen_glmm_znb1b,miauen_glmm_znb1c,miauen_glmm_znb1d)
```

```
Data: cat_l2
Models:
miauen_glmm_znb1: miauen ~ umfeld * zeit + (1 | versuch), zi=~1, disp=~1
miauen_glmm_znb1c: miauen ~ umfeld * zeit + (1 | versuch), zi=~umfeld, disp=~1
miauen_glmm_znb1d: miauen ~ umfeld * zeit + (1 | versuch), zi=~zeit, disp=~1
miauen_glmm_znb1b: miauen ~ umfeld * zeit + (1 | versuch), zi=~umfeld + zeit, disp=~1
miauen_glmm_znb1a: miauen ~ umfeld * zeit + (1 | versuch), zi=~umfeld * zeit, disp=~1
                  Df    AIC     BIC  logLik deviance  Chisq Chi Df Pr(>Chisq)
miauen_glmm_znb1  13 924.54  976.75 -449.27   898.54
miauen_glmm_znb1c 14 925.84  982.07 -448.92   897.84 0.7051      1     0.4011
miauen_glmm_znb1d 17 926.15  994.43 -446.08   892.15 5.6884      3     0.1278
miauen_glmm_znb1b 18 926.46  998.75 -445.23   890.46 1.6953      1     0.1929
miauen_glmm_znb1a 22 930.22 1018.58 -443.11   886.22 4.2324      4     0.3755
```

```
anova(miauen_glmm_znb1,miauen_glmm_znb1a,miauen_glmm_znb1b)
```

```
Data: cat_l2
Models:
miauen_glmm_znb1: miauen ~ umfeld * zeit + (1 | versuch), zi=~1, disp=~1
miauen_glmm_znb1b: miauen ~ umfeld * zeit + (1 | versuch), zi=~umfeld + zeit, disp=~1
miauen_glmm_znb1a: miauen ~ umfeld * zeit + (1 | versuch), zi=~umfeld * zeit, disp=~1
                  Df    AIC     BIC  logLik deviance  Chisq Chi Df Pr(>Chisq)
miauen_glmm_znb1  13 924.54  976.75 -449.27   898.54
miauen_glmm_znb1b 18 926.46  998.75 -445.23   890.46 8.0888      5     0.1514
miauen_glmm_znb1a 22 930.22 1018.58 -443.11   886.22 4.2324      4     0.3755
```

```
summary(miauen_glmm_znb1)
```

```
 Family: nbinom1  ( log )
Formula:          miauen ~ umfeld * zeit + (1 | versuch)
Zero inflation:          ~1
Data: cat_l2

     AIC      BIC   logLik deviance df.resid
   924.5    976.8   -449.3    898.5      397

Random effects:

Conditional model:
 Groups  Name        Variance Std.Dev.
 versuch (Intercept) 4.356    2.087
Number of obs: 410, groups:  versuch, 82

Dispersion parameter for nbinom1 family (): 5.05

Conditional model:
                           Estimate Std. Error z value Pr(>|z|)
(Intercept)                 -0.5580     0.4267  -1.308   0.1910
umfeldAnimal Shelter        -1.6043     0.9323  -1.721   0.0853 .
zeit2                       -0.3313     0.2849  -1.163   0.2449
zeit3                       -0.2448     0.2682  -0.913   0.3615
zeit4                       -0.6567     0.3257  -2.016   0.0438 *
zeit5                       -0.5023     0.2823  -1.780   0.0751 .
umfeldAnimal Shelter:zeit2   0.3355     0.7326   0.458   0.6470
umfeldAnimal Shelter:zeit3   0.8977     0.7649   1.174   0.2406
umfeldAnimal Shelter:zeit4   0.8042     0.7112   1.131   0.2582
umfeldAnimal Shelter:zeit5  -0.1099     0.7984  -0.138   0.8905
---
Signif. codes:  0 '***' 0.001 '**' 0.01 '*' 0.05 '.' 0.1 ' ' 1

Zero-inflation model:
            Estimate Std. Error z value Pr(>|z|)
(Intercept)  -1.7697     0.6236  -2.838  0.00454 **
---
Signif. codes:  0 '***' 0.001 '**' 0.01 '*' 0.05 '.' 0.1 ' ' 1
```

```
summary(miauen_glmm_nb1)
```

```
 Family: nbinom1  ( log )
Formula:          miauen ~ umfeld * zeit + (1 | versuch)
Data: cat_l2

     AIC      BIC   logLik deviance df.resid
   930.4    978.6   -453.2    906.4      398

Random effects:

Conditional model:
 Groups  Name        Variance Std.Dev.
 versuch (Intercept) 3.557    1.886
Number of obs: 410, groups:  versuch, 82

Dispersion parameter for nbinom1 family (): 8.37

Conditional model:
                           Estimate Std. Error z value Pr(>|z|)
(Intercept)                -0.63010    0.40860  -1.542   0.1230
umfeldAnimal Shelter       -1.10519    0.89058  -1.241   0.2146
zeit2                       0.09087    0.27804   0.327   0.7438
zeit3                      -0.04795    0.29801  -0.161   0.8722
zeit4                      -0.66032    0.33879  -1.949   0.0513 .
zeit5                      -0.16366    0.29927  -0.547   0.5845
umfeldAnimal Shelter:zeit2 -0.15215    0.78329  -0.194   0.8460
umfeldAnimal Shelter:zeit3  0.18403    0.86818   0.212   0.8321
umfeldAnimal Shelter:zeit4  0.80324    0.75074   1.070   0.2847
umfeldAnimal Shelter:zeit5 -0.43861    0.84404  -0.520   0.6033
---
Signif. codes:  0 '***' 0.001 '**' 0.01 '*' 0.05 '.' 0.1 ' ' 1
```

Effektstärke

```
##Cohen's d berechnen

# Get all variance components
reffects_miauen <- broom.mixed::tidy(miauen_glmm_znb1, effects = c("ran_pars"), scale="vcov")

# Calculate denominator (taking into account all relevant variance components)
denominator_miauen <- sqrt(sum(reffects_miauen$estimate))

# Get fixed effects estimates
fixed_effects <- summary(miauen_glmm_znb1)$coef$cond[, 1]

# Calculate Cohen's d
cohend_miauen <- data.frame(
  CohensD_miauen = fixed_effects[c(2,3,4,5,6,7,8,9,10)]/denominator_miauen
)
cohend_miauen
```

```
                           CohensD_miauen
umfeldAnimal Shelter          -0.76862950
zeit2                         -0.15874873
zeit3                         -0.11728601
zeit4                         -0.31465575
zeit5                         -0.24066806
umfeldAnimal Shelter:zeit2     0.16073771
umfeldAnimal Shelter:zeit3     0.43008790
umfeldAnimal Shelter:zeit4     0.38528219
umfeldAnimal Shelter:zeit5    -0.05265069
```

```
###wobei die Frage ist, ob man die Formel bei GLMM einfach so übernehmen darf. Das müsst ich selbst recherchieren
```

Grafik

```
#estimated marginal means
# geschätzen Randmittel

emm_miauen_m1 <- emmeans(miauen_glmm_znb1, "umfeld", by="zeit")
emm_miauen_m1b <- emmeans(miauen_glmm_znb1, "zeit", by="umfeld")
em_df_miauen_m1<- data.frame(emm_miauen_m1)

ggplot(em_df_miauen_m1, aes(x = zeit, y = emmean, group=umfeld, color=umfeld))+
  geom_line() +
    geom_point(aes(shape=umfeld))+
    geom_errorbar(aes(ymin=asymp.LCL, ymax=asymp.UCL), width=.4,
                position=position_dodge(0.01)) +
  scale_colour_manual(values=c("#D700FF","#00C0FA"))+
  scale_y_continuous(limits=c(-5,5),breaks=seq(0,5,1)) +
  theme_bw() +
  labs(x = "Video Sequence (No)",
       y = "Meow (number)",
       color = "Living Environment",
       shape = "Living Environment")
```

```
#Problem mit dem Ausdruck für die Confidenzintervalle:
#geom_errorbar(aes(ymin=lower.CL, ymax=upper.CL), width=.4, position=position_dodge(0.01))



emm_miauen_m1 <- emmeans(miauen_glmm_znb1, "umfeld", by="zeit")
emm_miauen_m1b <- emmeans(miauen_glmm_znb1, "zeit", by="umfeld")
em_df_miauen_m1<- data.frame(emm_miauen_m1)

###############################


em_df_miauen_m1 <- em_df_miauen_m1 %>%
  dplyr::mutate(emmean_trans = exp(emmean),
                asymp.LCL_trans = exp(asymp.LCL),
                asymp.UCL_trans = exp(asymp.UCL))

miauen_g1 <- ggplot(em_df_miauen_m1, aes(x = zeit, y = emmean_trans, group = umfeld, color = umfeld)) +
  geom_line() +
  geom_point(aes(shape = umfeld)) +
  geom_errorbar(aes(ymin = asymp.LCL_trans, ymax = asymp.UCL_trans), width = 0.2) +
  scale_colour_manual(values = c("#D700FF", "#00C0FA")) +
  scale_y_continuous(limits = c(0, 5), breaks = seq(0, 5, 1)) +
  theme_bw() +
  labs(x = "Video Sequence (No)",
       y = "Meow (number)",
       color = "Living Environment",
       shape = "Living Environment")

miauen_g1



ggplot(em_df_miauen_m1, aes(x = zeit, y = emmean_trans, group = umfeld, color = umfeld)) +
  # Füge ein Band für das Konfidenzintervall hinzu
  geom_ribbon(aes(ymin = asymp.LCL_trans, ymax = asymp.UCL_trans, fill = umfeld),
              alpha = 0.2, color = NA) +
  geom_line(linewidth = 1) +
  geom_point(aes(shape = umfeld), size = 3) +
  # Verbesserte Farbpalette mit benannten Beschriftungen
  scale_colour_manual(values = c("#D700FF", "#00C0FA")) +
  scale_fill_manual(values = c("#D700FF", "#00C0FA")) +
  # Y-Achse anpassen
  scale_y_continuous(limits = c(0, 5),
                     breaks = seq(0, 5, 1),
                     expand = c(0, 0)) +
  # X-Achse anpassen
  scale_x_discrete(expand = c(0.05, 0.05)) +
  # Theme anpassen
  theme_bw() +
  theme(
    panel.grid.minor = element_blank(),
    legend.position = "bottom",
    axis.title = element_text(size = 12, face = "bold"),
    axis.text = element_text(size = 10),
    legend.title = element_text(size = 11, face = "bold"),
    legend.text = element_text(size = 10)
  ) +
  # Beschriftungen
  labs(x = "Video Sequence (No)",
       y = "Meow (number)",
       color = "Living Environment",
       shape = "Living Environment",
       fill="Living Environment",
       group="Living Environment")
```

Paarweise Vergleiche

```
pairs(emmeans(miauen_glmm_znb1, "umfeld", by="zeit") , adjust="dunnet")

zeit = 1:
 contrast                       estimate    SE  df z.ratio p.value
 Private Home - Animal Shelter     1.604 0.932 Inf   1.721  0.0853

zeit = 2:
 contrast                       estimate    SE  df z.ratio p.value
 Private Home - Animal Shelter     1.269 0.932 Inf   1.361  0.1736

zeit = 3:
 contrast                       estimate    SE  df z.ratio p.value
 Private Home - Animal Shelter     0.707 0.937 Inf   0.754  0.4507

zeit = 4:
 contrast                       estimate    SE  df z.ratio p.value
 Private Home - Animal Shelter     0.800 0.939 Inf   0.852  0.3944

zeit = 5:
 contrast                       estimate    SE  df z.ratio p.value
 Private Home - Animal Shelter     1.714 1.006 Inf   1.703  0.0885

Results are given on the log (not the response) scale.

pairs(emmeans(miauen_glmm_znb1, "zeit", by="umfeld") , adjust="dunnet")

umfeld = Private Home:
 contrast      estimate    SE  df z.ratio p.value
 zeit1 - zeit2  0.33134 0.285 Inf   1.163  0.7893
 zeit1 - zeit3  0.24480 0.268 Inf   0.913  0.9018
 zeit1 - zeit4  0.65675 0.326 Inf   2.016  0.2683
 zeit1 - zeit5  0.50232 0.282 Inf   1.780  0.4012
 zeit2 - zeit3 -0.08654 0.259 Inf  -0.334  0.9976
 zeit2 - zeit4  0.32541 0.336 Inf   0.968  0.8809
 zeit2 - zeit5  0.17098 0.263 Inf   0.651  0.9698
 zeit3 - zeit4  0.41195 0.327 Inf   1.258  0.7355
 zeit3 - zeit5  0.25752 0.269 Inf   0.956  0.8856
 zeit4 - zeit5 -0.15443 0.336 Inf  -0.460  0.9917

umfeld = Animal Shelter:
 contrast      estimate    SE  df z.ratio p.value
 zeit1 - zeit2 -0.00415 0.677 Inf  -0.006  1.0000
 zeit1 - zeit3 -0.65288 0.705 Inf  -0.926  0.8971
 zeit1 - zeit4 -0.14741 0.632 Inf  -0.233  0.9994
 zeit1 - zeit5  0.61221 0.748 Inf   0.818  0.9321
 zeit2 - zeit3 -0.64873 0.778 Inf  -0.833  0.9277
 zeit2 - zeit4 -0.14326 0.641 Inf  -0.224  0.9995
 zeit2 - zeit5  0.61636 0.771 Inf   0.800  0.9373
 zeit3 - zeit4  0.50547 0.745 Inf   0.678  0.9650
 zeit3 - zeit5  1.26509 0.835 Inf   1.516  0.5720
 zeit4 - zeit5  0.75962 0.733 Inf   1.037  0.8517

Results are given on the log (not the response) scale.
P value adjustment: dunnettx method for 10 tests

confint(pairs(emmeans(miauen_glmm_znb1,~umfeld)))

 contrast                       estimate    SE  df asymp.LCL asymp.UCL
 Private Home - Animal Shelter      1.22 0.809 Inf    -0.366       2.8

Results are averaged over the levels of: zeit
Results are given on the log (not the response) scale.
Confidence level used: 0.95

confint(pairs(emmeans(miauen_glmm_znb1,~zeit)))

 contrast      estimate    SE  df asymp.LCL asymp.UCL
 zeit1 - zeit2   0.1636 0.368 Inf    -0.840     1.168
 zeit1 - zeit3  -0.2040 0.372 Inf    -1.219     0.811
 zeit1 - zeit4   0.2547 0.355 Inf    -0.714     1.223
 zeit1 - zeit5   0.5573 0.400 Inf    -0.535     1.649
 zeit2 - zeit3  -0.3676 0.417 Inf    -1.504     0.769
 zeit2 - zeit4   0.0911 0.361 Inf    -0.892     1.075
 zeit2 - zeit5   0.3937 0.407 Inf    -0.717     1.504
 zeit3 - zeit4   0.4587 0.399 Inf    -0.630     1.548
 zeit3 - zeit5   0.7613 0.441 Inf    -0.442     1.965
 zeit4 - zeit5   0.3026 0.402 Inf    -0.795     1.400

Results are averaged over the levels of: umfeld
Results are given on the log (not the response) scale.
Confidence level used: 0.95
Conf-level adjustment: tukey method for comparing a family of 5 estimates
```

Vorraussetzungen

```
# Poisson o. neg Binomial

# Struktur der zufälligen Effekte auf Normalverteilung prüfen

# DHARMa - prüft automatisch Residuen auf Normalverteilung & Abweichungen
q_unit <- DHARMa::simulateResiduals(miauen_glmm_znb1d)
plot(q_unit)



#Ausreißer
testOutliers(q_unit)



    DHARMa bootstrapped outlier test

data:  q_unit
outliers at both margin(s) = 0, observations = 410, p-value = 0.62
alternative hypothesis: two.sided
 percent confidence interval:
 0.00000000 0.01835366
sample estimates:
outlier frequency (expected: 0.006 )
                                   0
```

Schnurren (schnurren)

Verteilungen

```
# table(subset2)

#
# Zähldaten: Poisson Regression
# Voraussetzung: Erwartungswert = Varianz
# --> Praxis oft nicht so
# Alternative: Negativ binomial

ggplot(cat_l2, aes(x=schnurren)) +
  geom_histogram() +
  facet_wrap(~zeit)



#Graphischer Vergleich Poisson/negbin
# person 8 ausschließen

cat_l2b <- cat_l2 %>% dplyr::filter(!is.na(schnurren))

schnurren_p1 <- ggplot(cat_l2b) +
  geom_bar(aes(x = schnurren, y = after_stat(count)/sum(after_stat(count)))) +
  geom_pointrange(aes(x = schnurren,y = dpois(schnurren, mean(schnurren)),
                      ymin = 0,ymax = dpois(schnurren, mean(schnurren))),colour = "red") +
  labs(title = "Poisson fit",y = "Probability")

nbfit <- fitdist(cat_l2b$schnurren, "nbinom")

schnurren_p2 <- ggplot(cat_l2b) +
  geom_bar(aes(x = schnurren, y = after_stat(count)/sum(after_stat(count)))) +
  geom_pointrange(aes(x = schnurren,y = dnbinom(schnurren,
                                                 size = nbfit$estimate[1],mu = nbfit$estimate[2]),
                      ymin = 0,ymax = dnbinom(schnurren,size = nbfit$estimate[1],
                                              mu = nbfit$estimate[2])),colour = "blue") +
  labs(title = "Negative binomial fit",y = "Probability")

schnurren_p1 / schnurren_p2



#rechnen, welches Modell am besten:

schnurren_poisson <- glm(miauen ~ umfeld*zeit, family = poisson, data = cat_l2b)
schnurren_nb <- glm.nb(miauen ~ umfeld*zeit, data = cat_l2b)
#schnurren_zip <- gamlss(miauen ~ umfeld*zeit, sigma.formula =  umfeld*zeit,family = ZIP, data = cat_l2b)
#schnurren_zinbi <- gamlss(miauen ~ umfeld*zeit, nu.formula = umfeld*zeit,family = ZINBI, data = cat_l2b,control = gamlss.control(n.cyc = 200)) #standard ist 20 - model has not converged

AIC(schnurren_poisson,schnurren_nb)

                  df      AIC
schnurren_poisson 10 3655.031
schnurren_nb      11 1043.796

BIC(schnurren_poisson,schnurren_nb)

                  df      BIC
schnurren_poisson 10 3695.192
schnurren_nb      11 1087.974

miss <- cat_l2 %>% dplyr::filter(is.na(schnurren))
```

Modelle

```
# GLMM für negative Binomial

#g,p,nb1 und nb2 vergleichen und schauen welches am besten


schnurren_glmm_g <- glmmTMB(schnurren ~ umfeld* zeit + (1 | versuch),
              data = cat_l2)

schnurren_glmm_p <- glmmTMB(schnurren ~ umfeld* zeit + (1 | versuch),
              data = cat_l2,family=poisson)

# zero inflated poisson zip
schnurren_glmm_zip <- glmmTMB(schnurren ~ umfeld* zeit + (1 | versuch),
              data = cat_l2,family=poisson,ziformula = ~1)

# bestes Modell
schnurren_glmm_nb1 <- glmmTMB(schnurren ~ umfeld* zeit + (1 | versuch),
              data = cat_l2,family=nbinom1)

schnurren_glmm_nb2 <- glmmTMB(schnurren ~ umfeld* zeit + (1 | versuch),
              data = cat_l2,family=nbinom2)

schnurren_glmm_znb1 <- glmmTMB(schnurren ~ umfeld* zeit + (1 | versuch),
              data = cat_l2,family=nbinom1, ziformula = ~1)



summary(schnurren_glmm_nb1)

 Family: nbinom1  ( log )
Formula:          schnurren ~ umfeld * zeit + (1 | versuch)
Data: cat_l2

     AIC      BIC   logLik deviance df.resid
  1049.0   1097.2   -512.5   1025.0      398

Random effects:

Conditional model:
 Groups  Name        Variance Std.Dev.
 versuch (Intercept) 1.93     1.389
Number of obs: 410, groups:  versuch, 82

Dispersion parameter for nbinom1 family (): 0.411

Conditional model:
                           Estimate Std. Error z value Pr(>|z|)
(Intercept)                -0.59754    0.24575  -2.432   0.0150 *
umfeldAnimal Shelter        1.20509    0.47204   2.553   0.0107 *
zeit2                      -0.18808    0.19456  -0.967   0.3337
zeit3                      -0.38458    0.20675  -1.860   0.0629 .
zeit4                      -0.71041    0.23101  -3.075   0.0021 **
zeit5                      -0.08315    0.19057  -0.436   0.6626
umfeldAnimal Shelter:zeit2  0.09772    0.29150   0.335   0.7375
umfeldAnimal Shelter:zeit3 -0.42123    0.33868  -1.244   0.2136
umfeldAnimal Shelter:zeit4 -0.26981    0.36849  -0.732   0.4641
umfeldAnimal Shelter:zeit5 -0.50793    0.31338  -1.621   0.1051
---
Signif. codes:  0 '***' 0.001 '**' 0.01 '*' 0.05 '.' 0.1 ' ' 1

anova(schnurren_glmm_g,schnurren_glmm_p,schnurren_glmm_nb1, schnurren_glmm_znb1)

Data: cat_l2
Models:
schnurren_glmm_p: schnurren ~ umfeld * zeit + (1 | versuch), zi=~0, disp=~1
schnurren_glmm_g: schnurren ~ umfeld * zeit + (1 | versuch), zi=~0, disp=~1
schnurren_glmm_nb1: schnurren ~ umfeld * zeit + (1 | versuch), zi=~0, disp=~1
schnurren_glmm_znb1: schnurren ~ umfeld * zeit + (1 | versuch), zi=~1, disp=~1
                    Df    AIC    BIC  logLik deviance  Chisq Chi Df Pr(>Chisq)
schnurren_glmm_p    11 1059.9 1104.1 -518.94   1037.9
schnurren_glmm_g    12 1486.8 1535.0 -731.38   1462.8   0.00      1          1
schnurren_glmm_nb1  12 1049.0 1097.2 -512.52   1025.0 437.72      0     <2e-16
schnurren_glmm_znb1 13 1051.0 1103.2 -512.52   1025.0   0.00      1          1

schnurren_glmm_p
schnurren_glmm_g
schnurren_glmm_nb1  ***
schnurren_glmm_znb1
---
Signif. codes:  0 '***' 0.001 '**' 0.01 '*' 0.05 '.' 0.1 ' ' 1

# p-Werte extra ausgeben

parameters(schnurren_glmm_znb1)

# Fixed Effects (Count Model)

Parameter                           | Log-Mean |   SE |         95% CI |     z |     p
--------------------------------------------------------------------------------------
(Intercept)                         |    -0.60 | 0.25 | [-1.08, -0.12] | -2.43 | 0.015
umfeld [Animal Shelter]             |     1.21 | 0.47 | [ 0.28,  2.13] |  2.55 | 0.011
zeit [2]                            |    -0.19 | 0.19 | [-0.57,  0.19] | -0.97 | 0.334
zeit [3]                            |    -0.38 | 0.21 | [-0.79,  0.02] | -1.86 | 0.063
zeit [4]                            |    -0.71 | 0.23 | [-1.16, -0.26] | -3.08 | 0.002
zeit [5]                            |    -0.08 | 0.19 | [-0.46,  0.29] | -0.44 | 0.663
umfeld [Animal Shelter] × zeit [2] |     0.10 | 0.29 | [-0.47,  0.67] |  0.34 | 0.737
umfeld [Animal Shelter] × zeit [3] |    -0.42 | 0.34 | [-1.09,  0.24] | -1.24 | 0.214
umfeld [Animal Shelter] × zeit [4] |    -0.27 | 0.37 | [-0.99,  0.45] | -0.73 | 0.464
umfeld [Animal Shelter] × zeit [5] |    -0.51 | 0.31 | [-1.12,  0.11] | -1.62 | 0.105

# Fixed Effects (Zero-Inflation Component)

Parameter   | Log-Odds |      SE |              95% CI |         z |     p
---------------------------------------------------------------------------
(Intercept) |   -18.12 | 3731.10 | [-7330.95, 7294.71] | -4.86e-03 | 0.996

# Dispersion

Parameter   | Coefficient |       95% CI
-----------------------------------------
(Intercept) |        0.41 | [0.20, 0.84]

# Random Effects Variances

Parameter               | Coefficient |       95% CI
-----------------------------------------------------
SD (Intercept: versuch) |        1.39 | [1.08, 1.78]
```

Effektstärke

```
##Cohen's d berechnen

# Get all variance components
reffects_schnurren <- broom.mixed::tidy(schnurren_glmm_nb1, effects = c("ran_pars"), scale="vcov")

# Calculate denominator (taking into account all relevant variance components)
denominator_schnurren <- sqrt(sum(reffects_schnurren$estimate))

# Get fixed effects estimates
fixed_effects <- summary(schnurren_glmm_nb1)$coef$cond[, 1]

# Calculate Cohen's d
cohend_schnurren <- data.frame(
  CohensD_schnurren = fixed_effects[c(2,3,4,5,6,7,8,9,10)]/denominator_schnurren
)
cohend_schnurren

                           CohensD_schnurren
umfeldAnimal Shelter              0.86734699
zeit2                            -0.13536805
zeit3                            -0.27679695
zeit4                            -0.51130732
zeit5                            -0.05984720
umfeldAnimal Shelter:zeit2        0.07033249
umfeldAnimal Shelter:zeit3       -0.30317235
umfeldAnimal Shelter:zeit4       -0.19418879
umfeldAnimal Shelter:zeit5       -0.36557673

###wobei die Frage ist, ob man die Formel bei GLMM einfach so übernehmen darf. Das müsst ich selbst recherchieren
```

Grafik

```
#estimated marginal means
# geschätzen Randmittel

emm_schnurren_m1 <- emmeans(schnurren_glmm_nb1, "umfeld", by="zeit")
emm_schnurren_m1b <- emmeans(schnurren_glmm_nb1, "zeit", by="umfeld")
em_df_schnurren_m1<- data.frame(emm_schnurren_m1)

ggplot(em_df_schnurren_m1, aes(x = zeit, y = emmean, group=umfeld, color=umfeld))+
  geom_line() +
    geom_point(aes(shape=umfeld))+
    geom_errorbar(aes(ymin=asymp.LCL, ymax=asymp.UCL), width=.4,
                  position=position_dodge(0.01)) +
  scale_colour_manual(values=c("#D700FF","#00C0FA"))+
  scale_y_continuous(limits=c(-5,5),breaks=seq(0,5,1)) +
  theme_bw() +
  labs(x = "Video Sequence (No)",
       y = "Purr (number)",
       color = "Living Environment",
       shape = "Living Environment")



#Problem mit dem Ausdruck für die Confidenzintervalle:
#geom_errorbar(aes(ymin=lower.CL, ymax=upper.CL), width=.4, position=position_dodge(0.01))

###############################


em_df_schnurren_m1 <- em_df_miauen_m1 %>%
  dplyr::mutate(emmean_trans = exp(emmean),
                asymp.LCL_trans = exp(asymp.LCL),
                asymp.UCL_trans = exp(asymp.UCL))

schnurren_g1 <-ggplot(em_df_schnurren_m1, aes(x = zeit, y = emmean_trans, group = umfeld, color = umfeld)) +
  geom_line() +
  geom_point(aes(shape = umfeld)) +
  geom_errorbar(aes(ymin = asymp.LCL_trans, ymax = asymp.UCL_trans), width = 0.2) +
  scale_colour_manual(values = c("#D700FF", "#00C0FA")) +
  scale_y_continuous(limits = c(0, 5), breaks = seq(0, 5, 1)) +
  theme_bw() +
  labs(x = "Video Sequence (No)",
       y = "Purr (number)",
       color = "Living Environment",
       shape = "Living Environment")

schnurren_g1



ggplot(em_df_miauen_m1, aes(x = zeit, y = emmean_trans, group = umfeld, color = umfeld)) +
  # Füge ein Band für das Konfidenzintervall hinzu
  geom_ribbon(aes(ymin = asymp.LCL_trans, ymax = asymp.UCL_trans, fill = umfeld),
              alpha = 0.2, color = NA) +
  geom_line(linewidth = 1) +
  geom_point(aes(shape = umfeld), size = 3) +
  # Verbesserte Farbpalette mit benannten Beschriftungen
  scale_colour_manual(values = c("#D700FF", "#00C0FA")) +
  scale_fill_manual(values = c("#D700FF", "#00C0FA")) +
  # Y-Achse anpassen
  scale_y_continuous(limits = c(0, 5),
                     breaks = seq(0, 5, 1),
                     expand = c(0, 0)) +
  # X-Achse anpassen
  scale_x_discrete(expand = c(0.05, 0.05)) +
  # Theme anpassen
  theme_bw() +
  theme(
    panel.grid.minor = element_blank(),
    legend.position = "bottom",
    axis.title = element_text(size = 12, face = "bold"),
    axis.text = element_text(size = 10),
    legend.title = element_text(size = 11, face = "bold"),
    legend.text = element_text(size = 10)
  ) +
  # Beschriftungen
  labs(x = "Video Sequence (No)",
       y = "Purr (number)",
       color = "Living Environment",
       shape = "Living Environment")
```

Paarweise Vergleich

```
pairs(emmeans(schnurren_glmm_znb1, "umfeld", by="zeit") , adjust="dunnet")

zeit = 1:
 contrast                       estimate    SE  df z.ratio p.value
 Private Home - Animal Shelter    -1.205 0.472 Inf  -2.553  0.0107

zeit = 2:
 contrast                       estimate    SE  df z.ratio p.value
 Private Home - Animal Shelter    -1.303 0.477 Inf  -2.730  0.0063

zeit = 3:
 contrast                       estimate    SE  df z.ratio p.value
 Private Home - Animal Shelter    -0.784 0.508 Inf  -1.544  0.1225

zeit = 4:
 contrast                       estimate    SE  df z.ratio p.value
 Private Home - Animal Shelter    -0.935 0.527 Inf  -1.774  0.0760

zeit = 5:
 contrast                       estimate    SE  df z.ratio p.value
 Private Home - Animal Shelter    -0.697 0.491 Inf  -1.419  0.1560

Results are given on the log (not the response) scale.

pairs(emmeans(schnurren_glmm_znb1, "zeit", by="umfeld") , adjust="dunnet")

umfeld = Private Home:
 contrast      estimate    SE  df z.ratio p.value
 zeit1 - zeit2   0.1881 0.195 Inf   0.967  0.8814
 zeit1 - zeit3   0.3846 0.207 Inf   1.860  0.3530
 zeit1 - zeit4   0.7104 0.231 Inf   3.075  0.0182
 zeit1 - zeit5   0.0832 0.191 Inf   0.436  0.9932
 zeit2 - zeit3   0.1965 0.215 Inf   0.914  0.9013
 zeit2 - zeit4   0.5223 0.238 Inf   2.191  0.1896
 zeit2 - zeit5  -0.1049 0.199 Inf  -0.526  0.9862
 zeit3 - zeit4   0.3258 0.248 Inf   1.314  0.7021
 zeit3 - zeit5  -0.3014 0.211 Inf  -1.428  0.6298
 zeit4 - zeit5  -0.6273 0.235 Inf  -2.671  0.0598

umfeld = Animal Shelter:
 contrast      estimate    SE  df z.ratio p.value
 zeit1 - zeit2   0.0904 0.217 Inf   0.416  0.9943
 zeit1 - zeit3   0.8058 0.268 Inf   3.004  0.0228
 zeit1 - zeit4   0.9802 0.287 Inf   3.411  0.0059
 zeit1 - zeit5   0.5911 0.249 Inf   2.376  0.1257
 zeit2 - zeit3   0.7155 0.273 Inf   2.618  0.0690
 zeit2 - zeit4   0.8898 0.292 Inf   3.047  0.0199
 zeit2 - zeit5   0.5007 0.254 Inf   1.970  0.2922
 zeit3 - zeit4   0.1744 0.332 Inf   0.525  0.9863
 zeit3 - zeit5  -0.2147 0.299 Inf  -0.718  0.9571
 zeit4 - zeit5  -0.3891 0.316 Inf  -1.230  0.7518

Results are given on the log (not the response) scale.
P value adjustment: dunnettx method for 10 tests

confint(pairs(emmeans(schnurren_glmm_znb1,~umfeld)))

 contrast                       estimate    SE  df asymp.LCL asymp.UCL
 Private Home - Animal Shelter    -0.985 0.442 Inf     -1.85    -0.119

Results are averaged over the levels of: zeit
Results are given on the log (not the response) scale.
Confidence level used: 0.95

confint(pairs(emmeans(schnurren_glmm_znb1,~zeit)))

 contrast      estimate    SE  df asymp.LCL asymp.UCL
 zeit1 - zeit2    0.139 0.146 Inf   -0.2583    0.5367
 zeit1 - zeit3    0.595 0.169 Inf    0.1332    1.0572
 zeit1 - zeit4    0.845 0.184 Inf    0.3422    1.3484
 zeit1 - zeit5    0.337 0.157 Inf   -0.0902    0.7644
 zeit2 - zeit3    0.456 0.174 Inf   -0.0181    0.9301
 zeit2 - zeit4    0.706 0.188 Inf    0.1920    1.2202
 zeit2 - zeit5    0.198 0.161 Inf   -0.2425    0.6383
 zeit3 - zeit4    0.250 0.207 Inf   -0.3153    0.8155
 zeit3 - zeit5   -0.258 0.183 Inf   -0.7575    0.2413
 zeit4 - zeit5   -0.508 0.197 Inf   -1.0456    0.0292

Results are averaged over the levels of: umfeld
Results are given on the log (not the response) scale.
Confidence level used: 0.95
Conf-level adjustment: tukey method for comparing a family of 5 estimates
```

Vorraussetzungen

```
# 1. Poisson o. neg Binomial

# 2. Struktur der zufälligen Effekte auf Normalverteilung prüfen

# DHARMa - prüft automatisch Residuen auf Normalverteilung & Abweichungen
q_unit <- DHARMa::simulateResiduals(schnurren_glmm_nb1)
plot(q_unit)



#Ausreißer
testOutliers(q_unit)



    DHARMa bootstrapped outlier test

data:  q_unit
outliers at both margin(s) = 0, observations = 410, p-value = 0.68
alternative hypothesis: two.sided
 percent confidence interval:
 0.00000000 0.02195122
sample estimates:
outlier frequency (expected: 0.00590243902439024 )
                                                 0
```

Tabelle Ergebnisse - Analyse Subset 2

```
tab_model(ini_glmm_nb1, show.se = T, collapse.se = TRUE, show.r2 = F,  show.ci=F, p.style = "stars")
```

	kontaktini
Predictors	*Incidence Rate Ratios*
(Intercept)	6.10 *** (0.76)
umfeld [Animal Shelter]	1.39 (0.37)
zeit [2]	0.94 (0.11)
zeit [3]	0.73 * (0.09)
zeit [4]	0.49 *** (0.07)
zeit [5]	0.57 *** (0.08)
umfeld [Animal Shelter] × zeit [2]	0.94 (0.23)
umfeld [Animal Shelter] × zeit [3]	0.72 (0.20)
umfeld [Animal Shelter] × zeit [4]	0.67 (0.22)
umfeld [Animal Shelter] × zeit [5]	1.03 (0.28)
**Random Effects**	
σ^2	0.41
$\tau_{00\ versuch}$	0.48
ICC	0.54
$N_{versuch}$	82
Observations	409

* p<0.05 ** p<0.01 *** p<0.001

```
tab_model(ini_glmm_nb1, ver_glmm_nb1, miauen_glmm_znb1, schnurren_glmm_nb1, show.se = T, collapse.se = TRUE, show.r2 = F, show.ci=F, p.style = "stars", transform="exp")
```

	kontaktini	kontaktver	miauen	schnurren
Predictors	*Incidence Rate Ratios*	*Incidence Rate Ratios*	*Incidence Rate Ratios*	*Incidence Rate Ratios*
(Intercept)	6.10 *** (0.76)	0.63 * (0.14)	0.57 (0.24)	0.55 * (0.14)
(Intercept)	6.10 *** (0.76)	0.63 * (0.14)	155.56 (NA)	0.55 * (0.14)
umfeld [Animal Shelter]	1.39 (0.37)	1.98 (0.87)	0.20 (0.19)	3.34 * (1.58)
zeit [2]	0.94 (0.11)	1.21 (0.26)	0.72 (0.20)	0.83 (0.16)
zeit [3]	0.73 * (0.09)	1.13 (0.24)	0.78 (0.21)	0.68 (0.14)
zeit [4]	0.49 *** (0.07)	0.88 (0.20)	0.52 * (0.17)	0.49 ** (0.11)
zeit [5]	0.57 *** (0.08)	0.81 (0.19)	0.61 (0.17)	0.92 (0.18)
umfeld [Animal Shelter] × zeit [2]	0.94 (0.23)	0.47 (0.20)	1.40 (1.02)	1.10 (0.32)
umfeld [Animal Shelter] × zeit [3]	0.72 (0.20)	0.65 (0.27)	2.45 (1.88)	0.66 (0.22)
umfeld [Animal Shelter] × zeit [4]	0.67 (0.22)	0.39 (0.20)	2.23 (1.59)	0.76 (0.28)
umfeld [Animal Shelter] × zeit [5]	1.03 (0.28)	0.77 (0.33)	0.90 (0.72)	0.60 (0.19)
**Zero-Inflated Model**				
(Intercept)			0.17 ** (0.11)	
**Random Effects**				
σ^2	0.41	1.15	0.34	1.41
τ_{00}	0.48 versuch	1.16 versuch	4.36 versuch	1.93 versuch
ICC	0.54	0.50	0.93	0.58
N	82 versuch	82 versuch	82 versuch	82 versuch
Observations	409	409	410	410

* p<0.05 ** p<0.01 *** p<0.001

```
## Incidence Rate ratios sind standartmößig bei neg Binomial ausgegeben -
#Die IRR ist die Exponentiation des geschätzten Koeffizienten (β)
#mit tranform = NULL wird Estimates ausgegeben


tab_model(ini_glmm_nb1, show.se = TRUE, collapse.se = TRUE, show.r2 = FALSE, show.ci = FALSE, p.style = "stars", transform = NULL)
```

	kontaktini
Predictors	*Log-Mean*
(Intercept)	1.81 *** (0.12)
umfeld [Animal Shelter]	0.33 (0.27)
zeit [2]	-0.06 (0.12)
zeit [3]	-0.32 * (0.13)
zeit [4]	-0.71 *** (0.15)
zeit [5]	-0.56 *** (0.14)
umfeld [Animal Shelter] × zeit [2]	-0.06 (0.24)
umfeld [Animal Shelter] × zeit [3]	-0.33 (0.27)
umfeld [Animal Shelter] × zeit [4]	-0.41 (0.34)
umfeld [Animal Shelter] × zeit [5]	0.03 (0.27)
**Random Effects**	
σ^2	0.41
$\tau_{00\ versuch}$	0.48
ICC	0.54
$N_{versuch}$	82
Observations	409

* p<0.05 ** p<0.01 *** p<0.001

```
tab_model(ini_glmm_nb1, ver_glmm_nb1, miauen_glmm_znb1, schnurren_glmm_nb1,
          show.se = TRUE, collapse.se = TRUE, show.r2 = FALSE, show.ci = FALSE,
          p.style = "stars", transform = NULL)
```

	kontaktini	kontaktver	miauen	schnurren
Predictors	*Log-Mean*	*Log-Mean*	*Log-Mean*	*Log-Mean*
(Intercept)	1.81 *** (0.12)	-0.47 * (0.22)	-0.56 (0.43)	-0.60 * (0.25)
(Intercept)	1.81 *** (0.12)	-0.47 * (0.22)	5.05 (NA)	-0.60 * (0.25)
umfeld [Animal Shelter]	0.33 (0.27)	0.68 (0.44)	-1.60 (0.93)	1.21 * (0.47)
zeit [2]	-0.06 (0.12)	0.19 (0.21)	-0.33 (0.28)	-0.19 (0.19)
zeit [3]	-0.32 * (0.13)	0.12 (0.21)	-0.24 (0.27)	-0.38 (0.21)
zeit [4]	-0.71 *** (0.15)	-0.13 (0.23)	-0.66 * (0.33)	-0.71 ** (0.23)
zeit [5]	-0.56 *** (0.14)	-0.21 (0.23)	-0.50 (0.28)	-0.08 (0.19)
umfeld [Animal Shelter] × zeit [2]	-0.06 (0.24)	-0.76 (0.44)	0.34 (0.73)	0.10 (0.29)
umfeld [Animal Shelter] × zeit [3]	-0.33 (0.27)	-0.43 (0.41)	0.90 (0.76)	-0.42 (0.34)
umfeld [Animal Shelter] × zeit [4]	-0.41 (0.34)	-0.94 (0.52)	0.80 (0.71)	-0.27 (0.37)
umfeld [Animal Shelter] × zeit [5]	0.03 (0.27)	-0.26 (0.43)	-0.11 (0.80)	-0.51 (0.31)
**Zero-Inflated Model**				
(Intercept)			-1.77 ** (0.62)	
**Random Effects**				
σ^2	0.41	1.15	0.34	1.41
τ_{00}	0.48 versuch	1.16 versuch	4.36 versuch	1.93 versuch
ICC	0.54	0.50	0.93	0.58
N	82 versuch	82 versuch	82 versuch	82 versuch
Observations	409	409	410	410

* p<0.05 ** p<0.01 *** p<0.001

Grafiken Ergebnisse

```
ini_g1 + ver_g1 + miauen_g1 + schnurren_g1



#alle em_df Datensätze zusammengeben und rbind, davor muss man Variable einfügen mit dem Namen der Variable "exp", "ply"
#facet_wrap(~verhalten) zum Grafik basteln

# Angenommene Datensätze em_df_exp_m1, em_df_ply_m1, etc.
# Füge eine zusätzliche Variable 'verhalten' für jedes Dataset hinzu

# Erstelle Beispiel-Datensätze, wie im Beispiel beschrieben
em_df_ini_m1$verhalten <- "Initialisation"
em_df_ver_m1$verhalten <- "Avoidance"
em_df_miauen_m1$verhalten <- "Meow"
em_df_schnurren_m1$verhalten <- "Purr"



# Verbinde alle Datensätze zu einem großen DataFrame
combined_df2 <- rbind(em_df_ini_m1, em_df_ver_m1, em_df_miauen_m1, em_df_schnurren_m1)
View(combined_df2)

# Erstelle die ggplot-Grafik mit facet_wrap
ggplot(combined_df2    , aes(x = zeit, y = emmean_trans, group = umfeld, color = umfeld)) +
  geom_line() +
  geom_errorbar(aes(ymin = asymp.LCL_trans, ymax = asymp.UCL_trans), width = .4,
                position = position_dodge(0.01)) +
  geom_point(aes(shape = umfeld)) +
  scale_colour_manual(values = c("#D700FF", "#00C0FA")) +
  scale_y_continuous(limits = c(0, 14), breaks = seq(0, 14, 2)) +
  theme_bw() +
  labs(x = "Video Sequence (No)", y = "Behaviour (sec)", color = "Living Environment", shape = "Living Environment") +
  facet_wrap(~verhalten)
```
